# A Computational Approach for Designing a Peptide-Based Acetyl-CoA Synthetase 2 Inhibitor: A New Horizon for Anticancer Development

**DOI:** 10.1007/s12013-025-01729-y

**Published:** 2025-04-27

**Authors:** Musab Ali, Ernest Oduro-Kwateng, Ibrahim Oluwatobi Kehinde, Narasimham L. Parinandi, Mahmoud E. S. Soliman

**Affiliations:** 1 Molecular Bio-Computation and Drug Design Research Group, School of Health Sciences, University of KwaZulu Natal, Westville Campus, Durban, South Africa; 2 Division of Pulmonary, Critical Care and Sleep Medicine Department of Medicine, Davis Heart and Lung Research Institute, The Ohio State University, Weber Medical Center, Columbus, OH USA

**Keywords:** Peptide inhibitor, ACSS2, Anticancer agents, Molecular docking, Molecular dynamics simulations

## Abstract

Acetyl-CoA Synthetase 2 (ACSS2) has emerged as a new target for anticancer development owing to its high expression in various tumours and its enhancement of malignancy. Stressing the growing interest in peptide-derived drugs featuring better selectivity and efficacy, a computational protocol was applied to design a peptide inhibitor for ACSS2. Herein, 3600 peptide sequences derived from ACSS2 nucleotide motif were generated by classifying the 20 amino acids into six physiochemical groups. De novo modeling maintained essential binding interactions, and a refined library of 16 peptides was derived using Support Vector Machine filters to ensure proper bioavailability, toxicity, and therapeutic relevance. Structural and folding predictions, along with molecular docking, identified the top candidate, Pep16, which demonstrated significantly higher binding affinity (91.1 ± 1.6 kcal/mol) compared to a known inhibitor (53.7 ± 0.7 kcal/mol). Further molecular dynamics simulations and binding free energy calculations revealed that Pep16 enhances ACSS2 conformational variability, occupies a larger binding interface, and achieved firm binding. MM/GBSA analysis highlighted key electrostatic interactions with specific ACSS2 residues, including ARG 373, ARG 526, ARG 628, ARG 631, and LYS 632. Overall, Pep16 appears to lock the ACSS2 nucleotide pocket into a compact, rigid conformation, potentially blocking ATP binding and catalytic activity, and may serve as a novel specific ACSS2 inhibitor. Though, we urge further research to confirm and compare its therapeutic potential to existing inhibitors. We also believe that this systematic methodology would represent an indispensable tool for prospective peptide-based drug discovery.

## Introduction

Acetyl-CoA synthetase 2 (ACSS2) is an important member of the acetyl-CoA synthetase (ACSS) family which converts acetate into acetyl-CoA; the known intermediate metabolite in the metabolism of energy substrates [1, 2]. The ACSS family currently comprises three isoenzymes: ACSS1, ACSS2, and ACSS3. The primary mechanism governing the enzymatic activity of ACSS members is the acetylation and deacetylation of particular lysine sites. While Sirtuin 1 (SIRT1) activates ACSS2 in the nucleus and cytoplasm via deacetylating lysine 661, Sirtuin 3 (SIRT3) activates ACSS1 in the mitochondrial matrix by deacetylating lysine 635 [3]. Primarily ACSS2 is a cytosolic enzyme, however, it can be translocated to the nucleus [4, 5]. ACSS2 is the only enzyme that has been found to use free acetate within the nucleus to produce local acetyl-CoA [6–8]. In addition to its critical function in energy metabolism, ACSS2 also regulates the activity of acyltransferases that catalyse histone acetylation; thereby it plays a crucial role in cell division, bioenergetics, and gene expression regulation [9, 10]. Research on tumours has demonstrated that, in response to metabolic stress, cancer cells activate or upregulate the expression of ACSS2 to adapt to the growth conditions in the tumour microenvironment (TME) [11]. During stressful metabolic conditions, the amount of acetyl-CoA produced by glucose will decrease, while acetyl-CoA from acetate will increase significantly [6, 12, 13]. Numerous tumours, such as glioblastoma, breast cancer, liver cancer, prostate cancer, and others, have high expression levels of ACSS2 [6]. It has been connected to the tumour stage and the overall patient survival rate. A growing body of research indicates that hypoxia causes ACSS2 expression, which aids tumour cells in surviving in this nutrient-poor environment and promotes tumour growth, proliferation, invasion, and metastasis [6].

**Fig. 1:**
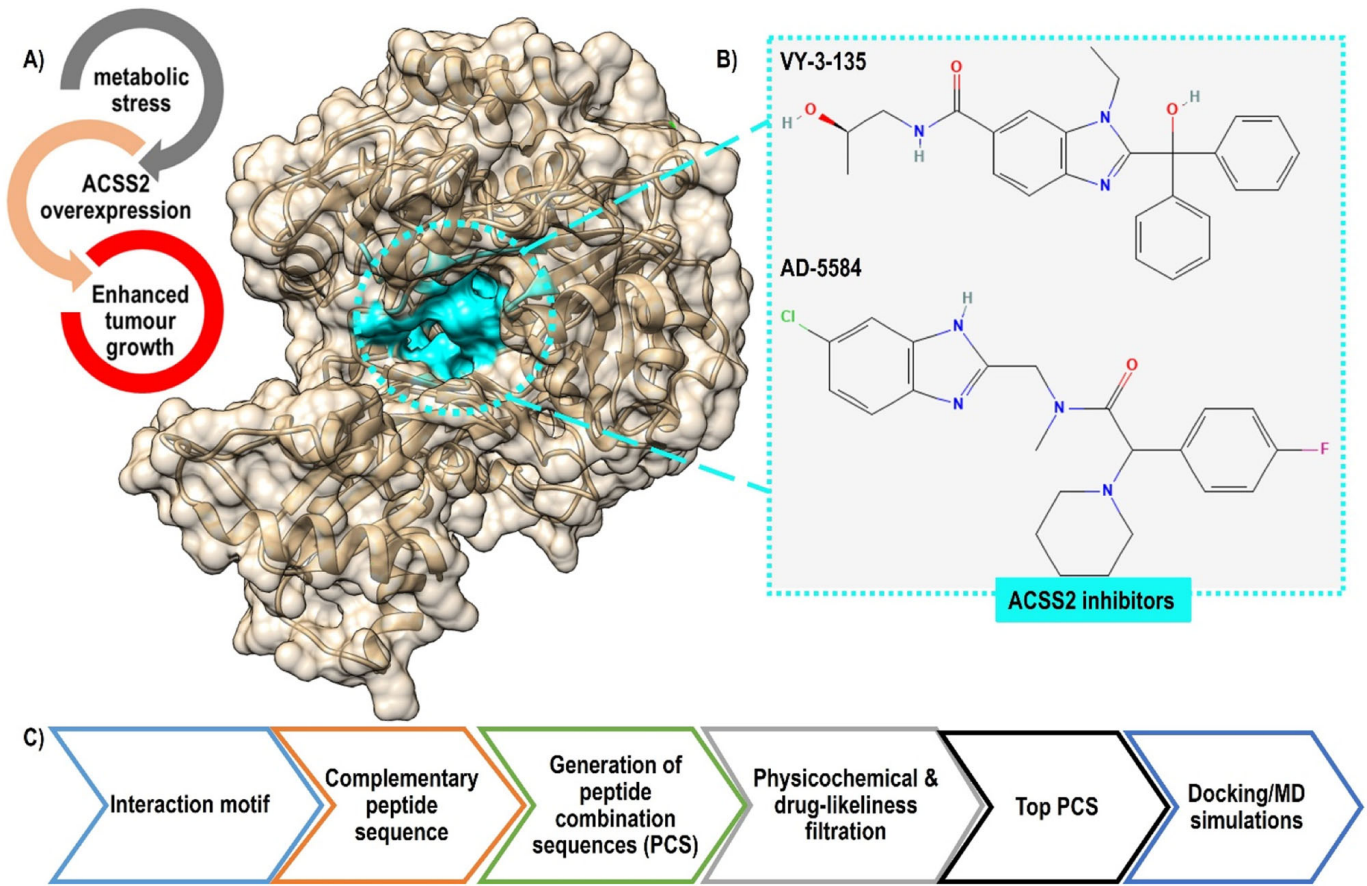
Overview of ACSS2 role in cancer and the previous computational pipeline used for designing peptide-based inhibitors. **A** The 3D structure of ACSS2 (PDBID:8W0D), highlighted in cyan is the nucleotide site. **B** Representative examples of some ACSS2 inhibitors that bind to nucleotide binding site. **C** Main steps involved in designing peptide-based inhibitors

The capacity of ACSS2 to stimulate lipid nuclear synthesis, acetylate histones in the nucleus, and promote the expression of autophagy genes are thought to be the probable mechanisms [14, 15]. Additionally, it contributes to cytoplasmic lipid synthesis and produces phospholipid membranes that promote fast tumour growth [16]. A schematic summary of ACSS2-mediated cancer progression alongside the 2D structure of representative inhibitors is given in Fig. 1.

Numerous investigations have demonstrated that ACSS2 knockdown can prevent the growth of a range of malignancies because ACSS2 is necessary for the development of tumour cells in hypoxia and energy stress [17]. Consequently, focusing on ACSS2 may offer cutting-edge methods for treating tumours and ACSS2 inhibitors can be effective in halting cancer growth and can be combined with other antineoplastic drugs to reduce drug resistance [6]. Zachary T. Schug and his team synthesized VY-3-135, a small molecule that demonstrated good specificity for ACSS2 with tumour growth suppression both in vitro and in vivo experiments conducted on mice and human breast models [18, 19]. Moreover, some pyridine and novel substituted tetrazoles were reported to inhibit ACSS2 invitro as proposed by Sabnis et al. [20, 21]. Despite some ACSS2 have reached the clinical trials (MTB-9655, Phase I trials), ACSS2 inhibitors are being investigated for their pharmacokinetic, specificity and podetial side-effects.

In silico computational tools have revolutionized drug discovery by reducing time and costs compared to traditional experimental methods [22]. These approaches, including molecular docking, molecular dynamics simulations, and QSAR modeling, enable rapid virtual screening of chemical libraries, facilitating efficient identification of potential drug candidates [23]. Additionally, they predict pharmacokinetic and pharmacodynamic properties crucial for assessing ADME-Tox profiles [24]. One key advantage of in silico methods is their ability to optimize lead compounds before synthesis, minimizing costly lab experiments [25]. These computational approaches play a vital role in various drug development stages, from target identification to lead optimization and clinical candidate selection [26]. Expanding on their relevance to drug discovery, in-silico approaches enable the rapid identification of critical molecular interactions that influence drug efficacy. For instance, predicting how mutations impact protein structure and function, computational tools assist in the rational design of small-molecule inhibitors or targeted therapies that specifically address oncogenic alterations [27–30]. These in‐silico methods not only provide detailed insights into the structural perturbations induced by deleterious mutations but also offer a cost-effective and high-throughput strategy for identifying potential therapeutic targets in oncogenesis [29, 31]. Collectively, these advances streamline the drug discovery pipeline and reduces the dependency on trial-and-error methodologies, saving both time and resources.

De novo peptide modeling has enabled the design of novel peptides with high affinity and specificity, offering promising applications in biosensing, therapeutic targeting, and synthetic biology. As example, Torres et al. developed an integrated computational protocol that combines parametric backbone generation, deep learning‐based methods, and RFdiffusion to design binder proteins targeting bioactive helical peptides [32]. Their approach, which includes iterative optimization strategies such as partial diffusion, yielded binders with picomolar affinities as validated by molecular dynamics simulations, fluorescence polarization assays, and high-resolution X-ray crystallography. In a complementary study, Wu et al. employed a strategy based on superhelical matching and geometric hashing to design modular peptide-binding proteins with repeating units [33]. Their de novo designs were rigorously validated using biophysical techniques such as small-angle X-ray scattering, circular dichroism, bio-layer interferometry, and yeast display, confirming both the structural accuracy and binding efficiency of the designed interfaces.

Targeting specific proteins with small molecules is common practice; nevertheless, some targets are challenging because of their size, flatness, and lack of natural substrates. Peptides are a common strategy for targeting unreachable or undruggable targets because of their large surface area and ability to interact with “hot spots” on the target’s binding surface. Target selectivity, biological affinity, and safety are all quite high for these peptides [34–36]. Certain peptide pharmacokinetic features, like bioavailability, permeability, and in vivo stability, are typically not in line with Lipinski’s rule of five. However, it has been demonstrated to be improved by current chemical modifications like N-methylation, cyclization, and the addition of D-amino acids [37–39].

To date, no crystal structure for human ACSS2 has been released; and available studies have largely relied on the *Salmonella enterica* crystallized ACSS2 [18, 40]. More recently, a crystal structure of ACSS 2 from *Candida* albicans has been uploaded to RCSB (PDBID:8W0D); which used for our design. In this study, we proposed a peptide-based ACSS2 inhibitor according to our previous computational protocol (Fig. 1C); in which we designed and optimised a novel and potentially disruptive peptide drug for p25-inducing-cyclin-dependent kinase (Cdk5) hyperregulation, a protein-protein interaction that has been linked to neuroinflammatory conditions like Alzheimer’s and Parkinsonism [41]. The process involved mapping a targeted protein’s interaction motif and generating a library of complementary peptide sequences that undergo strict physicochemical filtration, toxicity and folding patterns. Herein, we mapped the nucleotide-binding site, formerly identified as the binding site of known ACSS2 inhibitors [18, 40], details on the inhibitor design are described in the following sections.

## Computational Methods

### Design of Peptide Inhibitor (Pep16)

Acetyl-CoA, Adenosine monophosphate (AMP), and pyrophosphate (PP) are produced through the catalytic conversion of acetate, coenzyme A (CoA), and ATP by ACSS2. In the first stage, pyrophosphate is released while acylAMP is created, and in the second stage, acetylAMP is formed. The final product, acetyl-CoA, is then produced when Coenzyme A (CoA) takes the place of AMP [42]. Computational research on known inhibitors identified the nucleotide site as their primary binding site. They inhibit ACSS2 activity by competing with ATP and prevent CoA binding and hence subsequent catalytic steps [18, 20, 40]. et al. developed the prototype ACSS2 inhibitor, VY-3-135, and identified the essential residues composing the nucleotide-binding pocket of ACSS2 (GEPDTYWQ).

They also demonstrated that VY-3-135 occupies part of the CoA site in addition to preferring the protein’s acetyl-AMP binding site [18]. Recently, Alexej Dick and his team discovered AD-5584, a brain-penetrative inhibitor that showed improved ACSS2 inhibition and efficacy against breast cancer brain metastases [40]. Here, we used the nucleotide binding motif (GEPDTYWQ) as a starting point to construct a complementary peptide combination sequence (PCS), strictly screened for favorable drug-like properties to finally design the Pep16 molecule.

In accordance with Schug et al. sequence alignment of ACSS2 across different organisms; the nucleotide pocket is strongly conserved region consisting of eight amino acids, and only one amino acid change has been noted across studied organisms. To ensure enough similarity to human ACSS2, we compared our selected PDB (PDBID:8W0D) to an AlphaFold modelled human ACSS2 crystal structure (AF-Q9NR19-F1), furthermore, we calculated RMSD between these two models to assess their overall 3D patterns. Results of these comparisons are shown in Fig. 3 under Section 3.1.

#### Generation of peptide combination sequences (PCS)

The Peptide Combination Generator (https://pepcogen.bicfri.in/), uses the biological characteristics of amino acids to produce a wide range of peptide sequences with distinct physicochemical qualities. Amino acids are classified into six types depending on their properties: acidic (‘D’, ‘E’), basic (‘R’, ‘H’, ‘K’), hydrophobic and aliphatic (‘A’, ‘I’, ‘L’, ‘M’, ‘V’), aromatic (‘F’, ‘W’, ‘Y’), polar but uncharged (‘N’, ‘C’, ‘Q’, ‘S’, ‘T’), and unique (‘G’, ‘P’) [43]. These amino acids are flexible and can be positioned at various points in the peptide sequence to create a variety of peptide combinations that have potential drug-like properties [41, 43]. To achieve our goal, we generated every conceivable peptide combination sequence (PCS) using the recognized ACSS2 nucleotide pocket sequence (GEPDTYWQ). Following our previous pipeline [41], 3600 PCS were produced as a result, and they underwent thorough physicochemical screening to evaluate their toxicity profile (SVM greater >−0.30), molecular weight (< = 900 DA), amphipathicity (>0.30), hydrophobicity (> = 0.2), and tendency for aggregation in vivo (Na4vSS > −57). This extensive screening procedure intended to find peptide candidates with good drug-like properties and minimal off-target effects, leading to the selection of 16 peptide hits (Table 1).

**Table 1:** Physiochemical properties of the top-hit PCS showing their predictive values for toxicity, hydrophobicity, amphipathicity, molecular weights, in vivo aggregation, and folding energies

PeptideID	Peptide Name	Peptide Sequence	SVM Score	Prediction	Hydrophobicity	Amphipathicity	Mol wt	Na4vSS	sOPEP energy
2606	Pep1	PDPECYYS	−0.12	Non-Toxin	−0.21	0.16	973.11	−26.8	−2.80309
2607	Pep2	PDPECYYT	−0.18	Non-Toxin	−0.2	0.16	987.14	−25.8	−2.36648
3284	Pep3	PEPDCYYN	−0.29	Non-Toxin	−0.26	0.16	1000.14	−36.5	−7.09487
359	Pep4	GDGECYYN	−0.17	Non-Toxin	−0.2	0.16	920.02	−38.4	−1.26908
2385	Pep5	PDPDCYYQ	−0.25	Non-Toxin	−0.27	0.16	1000.14	−40.2	−2.49303
658	Pep6	GDPDQWYC	−0.18	Non-Toxin	−0.2	0.16	983.12	−57	−3.63035
2159	Pep7	PDGECYYN	−0.25	Non-Toxin	−0.23	0.16	960.08	−36.9	−1.71421
2594	Pep8	PDPECWYN	−0.15	Non-Toxin	−0.21	0.16	1023.18	−35.9	−2.46027
135	Pep9	GDGDCYYQ	−0.28	Non-Toxin	−0.22	0.16	920.02	−44.2	−1.9197
668	Pep10	GDPDQYWC	−0.22	Non-Toxin	−0.2	0.16	983.12	−56.7	−3.73611
1034	Pep11	GEGDCYYN	−0.23	Non-Toxin	−0.2	0.16	920.02	−40.5	−2.2124
2834	Pep12	PEGDCYYN	−0.18	Non-Toxin	−0.23	0.16	960.08	−39	−2.70096
580	Pep13	GDPDCYWQ	−0.15	Non-Toxin	−0.2	0.16	983.12	−42.9	−2.40548
2370	Pep14	PDPDCWYQ	−0.27	Non-Toxin	−0.23	0.16	1023.18	−41.7	−2.45375
2609	Pep15	PDPECYYN	−0.11	Non-Toxin	−0.26	0.16	1000.14	−34.4	−2.36291
1930	Pep16	PDGDCYWQ	−0.22	Non-Toxin	−0.2	0.16	983.12	−44	−2.9538

#### Peptide toxicity and physiochemical properties prediction

A crucial component of peptide design and development, especially in the areas of therapeutic applications and drug discovery, i the prediction of peptide toxicity. To examine the possible adverse effects of peptides on biological systems, we used the toxicity prediction program ToxinPred (https://webs.iiitd.edu.in/raghava/toxinpred/), which uses the Support Vector Machine (SVM) machine learning technique [44]. Quantitative matrix (QM) models, presence of motifs, dipeptide composition (DPC), and amino acid composition (AAC) are some of the properties of peptide sequences that the SVM models in ToxinPred are built on. While lower scores imply a reduced chance of the peptide being categorized as a toxin, higher scores show a higher confidence in the prediction that the peptide is a toxin [44, 45]. Moreover, since ToxinPred trains its SVM algorithm on several amino acid sequence properties to encode information about the physicochemical properties of the peptides [46], we applied it to predict the physiochemical properties of the PCS. In this work, we used strict filters to assess the in vivo propensity for aggregation, hydrophobicity, amphipathicity, and isoelectric point. Thereby, ranking peptides according to their favourable pharmacological characteristics, which increased their therapeutic potential.

#### Peptide aggregation propensity prediction

One of the essential components of the sensible design of bioactive peptide inhibitors is the ability to predict the tendency of peptide aggregation profiles [47]. Using physiochemical properties as our basis for final PCS prediction after filtration, we utilized the webserver-based tool AGGRESCAN [48]. The polypeptide aggregation features of AGGRESCAN (http://bioinf.uab.es/aggrescan/) are predicted through multiple critical steps using the aggregation-propensity value (a4v) per amino acid calculation that was previously derived from experimental data. Using an aggregation propensity scale developed from in vivo research, aggregation-prone regions inside polypeptides were first discovered. Second, the program effectively identified known aggregation-promoting sites in a variety of proteins by validating its predictions against experimental data, demonstrating the predictive dependability of the system. Following this, aggregation-prone segments were ranked according to peak area or normalized peak area by AGGRESCAN, which yielded a quantitative evaluation of their contribution to the behaviour of polypeptide aggregation. In summary, the program provided insights into the features of protein aggregation by predicting the rate of aggregation and identifying portions within polypeptide sequences that are prone to aggregation. The normalized a4v Sum Score (Na4vSS), which is another name for the aggregation propensity score, was computed using the aggregation-propensity scale for natural amino acids obtained from in vivo experiments [48]. Sixteen peptides (Pep1–Pep16) were determined to have good aggregation propensity scores and were selected for structure prediction to ensure appropriate folding. The greater the score, the higher the tendency of the corresponding amino acid to promote aggregation.

#### Prediction of peptide folding

Accurate peptide structure prediction aids in identifying bioactive compounds, understanding binding interactions with target proteins, and optimizing peptide sequences for efficacy and specificity. To precisely model the spatial arrangement of peptides based on their amino acid sequences and physicochemical properties, we employed PEP-FOLD4, an advanced de novo peptide structure prediction server that incorporates the most recent advancements to the sOPEP force field, in our peptide drug design approach [49]. Using a fragment-based methodology, PEP-FOLD4 (available at https://bioserv.rpbs.univ-paris-diderot.fr/services/PEP-FOLD4/) views a peptide as a collection of four amino acid fragments that overlap by three amino acids. PEP-FOLD4 includes a Debye-Hueckel formalism to account for fluctuations in salt concentration and pH levels, as well as a new formalism to address non-bonded interactions. Based on a two-state process, the greedy algorithm technique is used. A PSI-BLAST search against a database of sequences was used to create an amino acid profile, which was then used to forecast the likelihood of each structural alphabet (SA) state at each place in the peptide sequence. To predict the SA profile from the amino acid profile, a support vector machine (SVM) is employed. 3D model generation from the SA profile was the main goal of the second step. To sample the conformational space given by the SA profile, algorithms based on the Hidden Markov Model (HMM) paradigm were employed, such as the forward-backtrack technique.

**Fig. 2:**
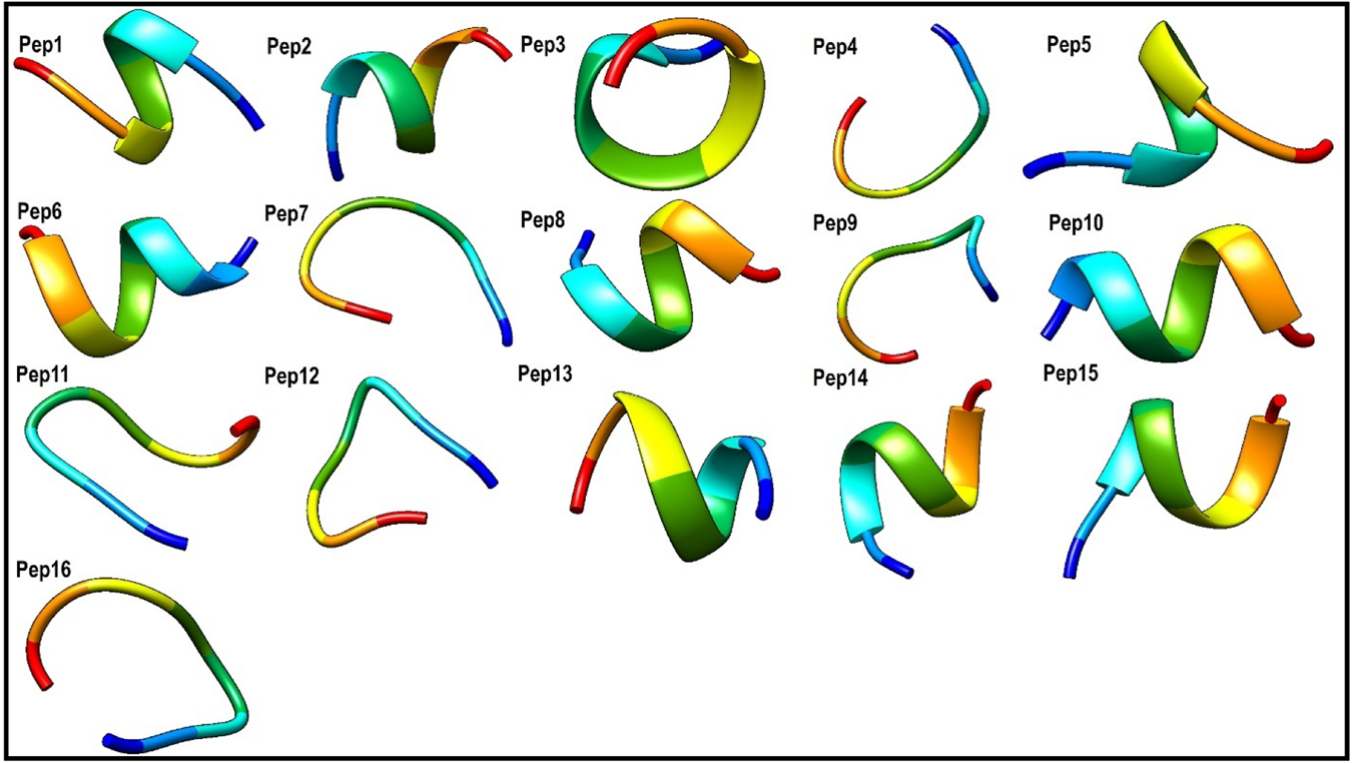
Predicted 3D structures of Pep1-Pep16. The residues are coloured using rainbow gradient, side chains are not shown

This process generates a set of states or trajectories that reflect prospective structures. Given a trajectory, prototype segments associated with each SA state were tightly joined to gradually construct the entire peptide structure (Fig. 2). After generating a complete structure, it is refined using Monte Carlo methods. During this revision, fragments were randomly swapped to improve the structure. The greedy algorithm was driven by the implicit solvent sOPEP coarse-grained force field, which provided energy terms that controlled conformation selection [49].

In comparison to atomistic models, this coarse-grained force field can decrease the number of degrees of freedom, improving computing efficiency and facilitating faster conformational space exploration. It also catches the crucial interactions, such as side-chain and backbone hydrogen bonding, that control peptide folding. Its precision and level of information, however, are limited.

#### Peptide-protein docking

To clarify the molecular interactions and provide insight into the binding mode, peptide docking to ACSS2 was conducted. For this reason, the docking investigations employed High Ambiguity Driven protein-protein DOCKing (HADDOCK), a flexible and adaptable docking technique that combines experimental data with computer modelling to account for the flexibility of both the peptide and the protein-protein complex [50]. It’s especially helpful for researching protein-peptide interactions since it can adapt to conformational changes that happen during binding. Before docking, the 3D structure of ACSS2 (PDB: 8W0D) was prepared using UCSF Chimera tools [51]. This involves the removal of all nonstandard residues, including water molecules and co-crystallized CoA, as well as redundant chains. Ambiguous interaction restraint (AIR) is the technique used by the HADDOCK docking server (https://wenmr.science.uu.nl/haddock2.4/) to make binding interface interactions easier [52]. The ACSS2 residues (ILE 321, THR 322, SER 396, VAL 397, GLY 398, GLU 399, PRO 400, ILE 401, ASP 422, THR 423, TYR 424, TRP 425, GLN 426, THR 427, THR 511, ASP 513, ILE 525, GLY 527, ARG 528, ASN 534, GLY 537, and ARG 539) were defined as the active binding site. First, orientations were randomly generated during the docking process. Next, energy was minimized through rigid-body optimization, producing a total of 1000 complexes. Once binding interactions were optimized, 200 solutions were chosen and refined in the torsion angle space with partial flexibility.

Finally, they were clustered using pairwise backbone root mean square deviation (RMSD), using a 1.5 Å threshold. At each level, the structures were sorted and scored using the weighted scoring system; the best structures were kept for further refinement, and the cluster with the lowest HADDOCK score was chosen as the most likely answer to the final approved docked complex. A weighted sum of several energy terms, such as the van der Waals energy, electrostatic energy, desolvation energy, restraint violation energy, and buried surface area, makes up the HADDOCK docking scoring function. Docked against AD5583, Pep16 achieved the highest docking score among PCS and subsequently prepared for molecular dynamics simulations.

### Molecular Dynamics (MD) Simulation

MD simulations were conducted on unbound ACSS2, AD5584-ACSS2, and Pep16-ACSS2 using Particle Mesh Ewald MD (PMEMD) Compute Unified Device Architecture (PMEMD. CUDA) dual graphic processor unit (GPU) featured in AMBER 18 package [53]. Utilizing the FF14SB AMBER force field for parameterization, the proteins were solvated and neutralized by adding hydrogen atoms, sodium ions, and counteracting chloride ions through the use of the LEAP module. Atomic solvation was achieved using a TIP3P box with 12 Å water molecules for each system. ACSS2 topologies were altered with the pdb4amber command before the LEAP protocol.

Partial minimization (2500 steps with 500 kcal/mol restraint potential) and full minimization (200 steps without conjugate restraint potential) were conducted [54]. Each system was then heated in a canonical ensemble (NVT) for 50 ps from 0 to 300 K using a Langevin thermostat that had a 1 ps random collision and a 10 kcal/mol Å harmonic potential restriction. Then, utilizing the SHAKE algorithm for the hydrogen bond constraint and the Barendsen-Barostat’s 1 bar pressure supply, equilibration was carried out. MD simulations were run in an isothermal-isobaric (NPT) ensemble at 300 K and a constant pressure of 1 bar for 300 ns with a 2-fs time interval [41, 54, 55]. Trajectories were saved every 1 ps and analysed using the CPTRAJ and PTRAJ modules of AMBER 18 GPU [56]. Schrödinger Maestro [57] and UCSF Chimera packages were used for visualization, structural and interaction analyses. Plots and statistical values were produced with the Origin program [54].

### Binding free energy (BFE) calculations

Using the generalized Born surface area/molecular mechanics (MM/GBSA) technique, the free energy of binding (BFE) between Pep16 and the AD5584 reference was calculated [26]. The MM/GBSA model predicts and evaluates the binding energies of molecular interactions in biological systems. MM/GBSA combines molecular mechanics computations with classical force fields to characterize molecular interactions, the generalized Born (GB) dielectric continuum solvent model, and surface area (SA) parameters to estimate the BFE. Internal energy, van der Waals interactions, electrostatic interactions, and other molecular forces were all included in the calculations of molecular mechanics.

The GB model took into account the atoms’ pairwise interactions and Born radii to predict the polar solvation-free energy. The surface tension constant (γ) was set at 0.0072 kcal/mol Å^2^, and the water probe radius of 1.4 Å was used to correlate the nonpolar solvation energy with the peptide-protein-protein interface surface area. This allowed the SA method to quantify the changes in hydrophobic interactions upon binding by calculating the buried surface area (BSA) during complex formation. The estimation of BFE was done with 100,000 MD trajectory frames [41, 54, 58]. The formula for BFE (∆G) is as follows:

$$\begin{array}{c}\Delta {G}_{{bind}}={G}_{{complex}}-{G}_{{receptor}}-{G}_{{ligand}}\\ \Delta {G}_{{bind}}={E}_{{gas}}+{G}_{{sol}}-T\Delta S\\ {E}_{{gas}}={E}_{\mathrm{int}}+{E}_{{vdw}}+{E}_{{ele}}\\ {G}_{{sol}}={G}_{{GB}}+{G}_{{SA}}\\ {G}_{{SA}}=\gamma {SASA}\end{array}$$

where ΔG_bind_ signifies the gas-phase summation, E_gas_ is the gas-phase energy, G_sol_ is the free solvation energy, TΔS is the total interaction entropy, E_int_ is the internal energy, E_ele_ is the Coulomb energy, and E_vdw_ is the van der Waals energy. E_gas_ was calculated from the AMBER FF14SB force field, and G_sol_ was calculated from the energy contributions of polar and non-polar states.

The MM/GBSA method presents several inherent limitations in estimating binding free energies. Notably, it neglects the conformational reorganization of both the receptor and the ligand upon binding and does not explicitly account for the contribution of water molecules in the binding site, thereby omitting relevant entropic and energetic effects. Furthermore, entropy is typically estimated via normal-mode analysis, which captures only the local vibrational properties of a fixed binding conformation and fails to include the full conformational entropy, resulting in high statistical uncertainty and necessitating numerous independent simulations for reliable estimates. In addition, the calculated binding free energies are highly sensitive to the parameters of the continuum solvation model—such as the specific GB variant, atomic radii, and dielectric constant—with even minor variations leading to significant differences in computed energies. Finally, the method’s reliance on classical force fields introduces limitations in accurately describing electrostatic and van der Waals interactions. Collectively, these factors suggest that, although MM/GBSA may provide qualitative insights and help rationalize observed binding trends, it may not be sufficiently precise for quantitative predictions in drug design.

## Results and Discussion

### Computational Screening and Optimization for Peptide-based ACSS2 Inhibitor

**Fig. 3:**
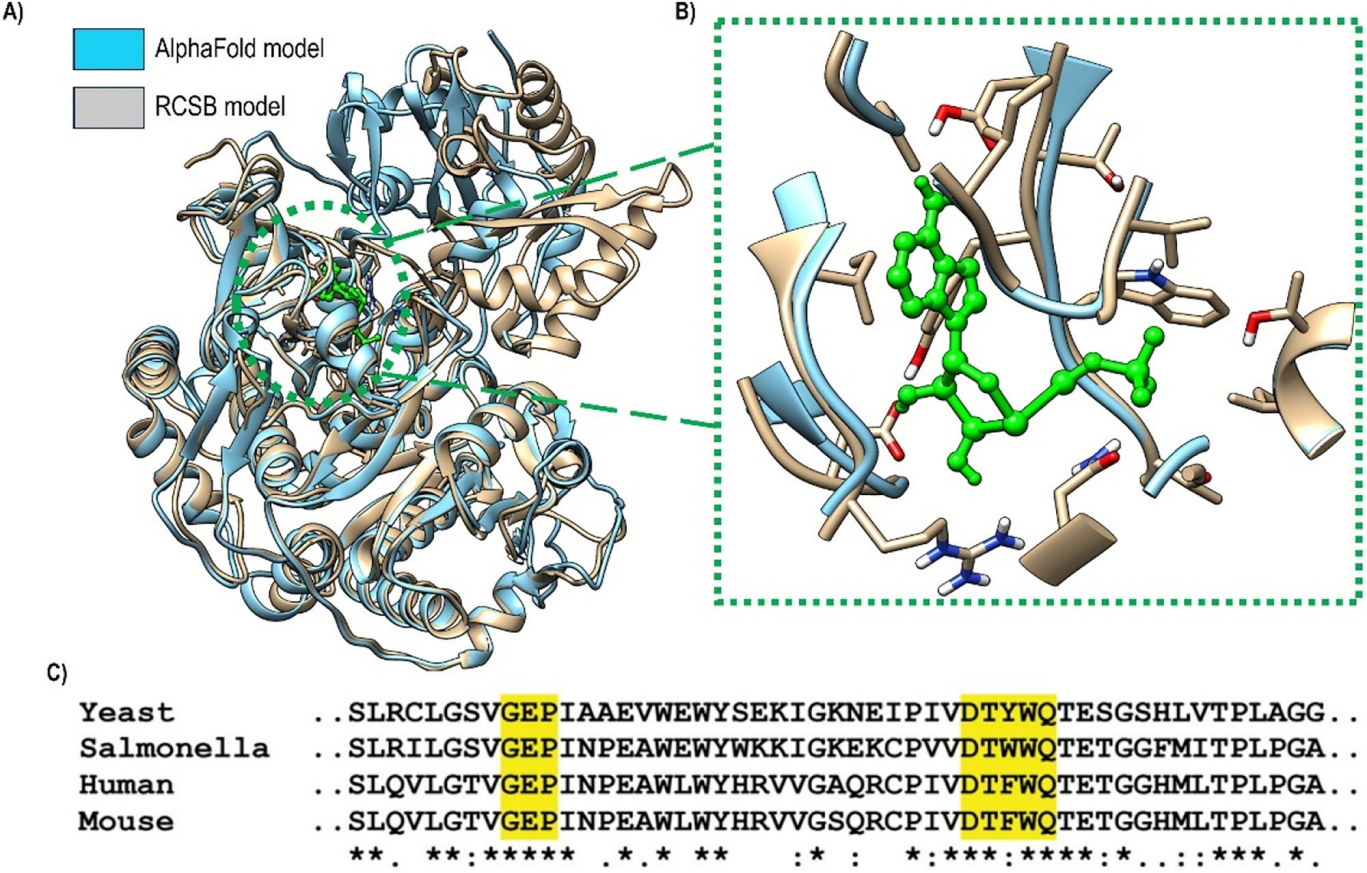
Structural relevance of selected protein to human ACSS2. **A** Superimposed structures for 8W0D and AF-Q9NR19-F1. **B** The nucleotide pocket showing ATP in green ball and stick representation. **C** Amino acid sequence alignment adapted from Schug et al. [18, 19]. Key residues that create the nucleotide binding pocket of ACSS2 are highlighted in yellow

Prior to the design, we made superimposed the Candida and AlphaFold predicted model of human ACSS2 (8W0D and AF-Q9NR19-F1, respectively) and calculated the RMSD between the 3D structures. Furthermore, we compared the nucleotide forming pocket residues previously reported. As can be depicted under Fig. 3, the two proteins show very similar 3D pattern (Fig. 3(A)), having an RMSD value of 0.78 Å. Most importantly, the near-identical overlapping of the nucleotide pocket (Fig. 3(B)). The latter turned to have the amino acid phenylalanine (F) in human, rather than tyrosine (Y) in yeast (Fig. 3(C)); thereby we implemented the (GEPDTYWQ) motif for our design.

A different amino acid with the same physicochemical properties—basic, hydrophobic and aliphatic, aromatic, polar but uncharged, and unique—was substituted for each amino acid position. We were able to build peptide sequences with certain features including charge, amphipathicithy, hydrophobicity, and molecular weight by classifying amino acid residues according to these physical attributes [41]. With ensured structural diversity and enhanced structural integrity, we finally reached a peptide library comprising 3600 PCS (Table S5), for which dedicated physicochemical and drug-likeliness screening was employed.

Employing the Support Vector Machine (SVM) algorithm (Via the toxinPred server) to assess the toxicity and physicochemical properties of the peptide combination sequences (PCS). SVM’s ability to handle complex data, model non-linear relationships, and produce robust and reliable predictions made it an appropriate choice for our investigation [46]. Prioritizing the evaluation of safety and toxicity over properties including hydrophobicity, isoelectric point, and amphipathicity during PCS screening. By ensuring that potentially hazardous peptides were found and eliminated early in the screening procedure, this method decreased the possibility of choosing peptides for additional applications that would have unfavourable toxic effects (cytotoxicity, immunogenicity, or off-target effects. Considering only PCS having SVM greater than −0.30 for the initial phase of the screening process, yielded 172 PCS (Table S1). Next, we examined the PCS for amphipathicity, taking into account moderate to high degrees of amphipathicity. Peptides with amphipathicity greater than 0.30 were filtered out, giving rise to 88 PCS (Table S2). To make it easier to interact with the target proteins’ hydrophobic areas, we then screened for hydrophobicity by taking moderate to high values into account. Peptide inhibitors with moderate to high hydrophobicity can engage in favourable hydrophobic interactions with hydrophobic surface regions of target proteins, promoting binding and stabilization of the protein-peptide complex, and contributing to the binding affinity and molecular recognition of the peptides [59–61]. To prevent aggregation, poor solubility, and non-specific and/or poor interactions that could compromise the effectiveness of peptides, PCS with too low hydrophobicity were excluded. A hydrophobicity greater or equal to −0.20 was used, resulting in 19 PCS (Table S3).

Up to this point, all PCS shared a charge of −2.0 and comparable molecular weights averaging around 900 DA. Notably, peptides with higher molecular weights are therapeutically attractive in many ways. First, they could potentially provide a wider binding interface with target proteins achieving enhanced selectivity and affinity. Secondly, they offer much structural stability; preserving activity and conformation. This is explainable by the existence of more amino acids and the potential for secondary structures. Lastly, they feature prolonged biological activity looking to their longer half-life in biological systems and lower clearance rates [62], enhance enhanced proteolytic resistance upon modification and their inherit balance between solubility and membrane permeability [63]. As a result, every PCS was kept and put through additional testing to determine whether they were likely to aggregate in vivo.

**Fig. 4:**
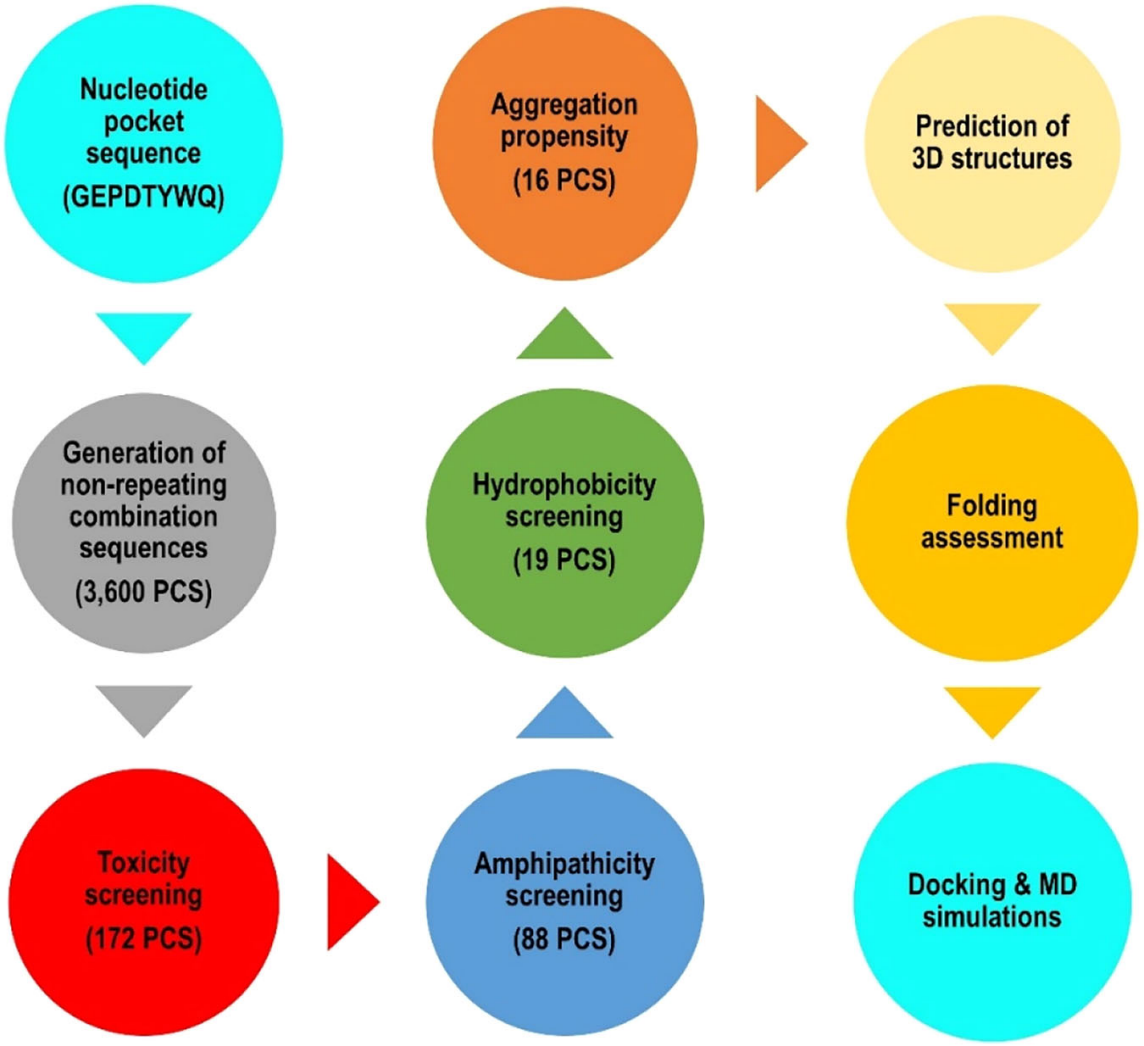
Schematic flowchart of the computational screening and optimization protocol used in the development and optimization of the 16 PCS inhibitors derived from the nucleotide pocket binding motif

The potential of aggregation in vivo was assessed using using the AGGRESCAN algorithm. PCS with a Normalized a4v Sum Score (Na4vSS) less than −57 were excluded, resulting in the top 16 PCS (Table S4). Therefore, in comparison to the primary results (Table S5), the strict screening protocol we implemented produced PCS with predicted favourable toxicological profiles, increased bioavailability, longer half-life, decreased risk of in vivo aggregation, and overall enhanced specificity towards ACSS2. Additionally, the solvent sOPEP coarse-grained force field was employed to further optimize the technique. Were able to predict the 3D peptide structural conformations of the top-16 PCS with accuracy (Fig. 2). The sOPEP force field is calculated as the sum of the local (E_local_), nonbonded (E_nonbonded_), and hydrogen bonding (E_h-bond_) energy terms. The E_local_ reflects the local interactions inside a peptide, including bond lengths, bond angles, and dihedral angles. Non-covalent interactions like electrostatic and van der Waals forces are accounted for by E_nonbonded_ interactions, whereas the energy involved in hydrogen bonding interactions between the peptide’s backbone atoms is reflected by E_h-bond_ interactions [64]. Because these conformations correspond to states that are favourable in terms of energy, peptides tend to adopt lower energy conditions. Lower energy values, according to sOPEP energy, imply a more stable conformation that is physiologically relevant and functionally active. Concerning molecular modelling investigations, it can be deduced that all 16 of the PCS showed energetically favourable states, indicating correct peptide folding and supporting their heat stability and increased therapeutic potential. A summary of the design and optimization process is given in Fig. 4.

### Peptide Sequences Showed Greater Affinity for ACSS2

Peptides typically have vast interaction surfaces, which allow them to interact with target proteins in a targeted and comprehensive manner. Their capacity to adopt diverse secondary structures enables them to adhere to target proteins’ specific shape and charge distribution, hence increasing selectivity by perfectly fitting the binding site. Furthermore, peptide inhibitors can be modified to improve their cellular uptake and distribution, increasing the likelihood that they will reach their targeted targets and decreasing the possibility of off-target effects [36, 41, 65]. Molecular docking indicated that PCS exhibited more preferential binding than the reference inhibitor and ATP. Binding affinities and interacting residues are given in Table 2.

**Table 2:** Molecular docking scores of Pep1-Pep16, compared to the reference inhibitor, AD5584

Peptide	Docking Score	Interacting Residues (within 5 Å)
Pep1	−66.2+/− 3.7	**ARG 373**, GLU 399, **GLY 446**, ARG 526, **ARG 528**, ASN 534, ASP 565, GLN 570, GLU 637, GLY 514, GLY 527, GLY 562, GLY 569, HIS 564, ILE 401, ILE 405, ILE 525, ILE 563, **LYS 632**^**S**^, PRO 369, PRO 400, PRO 403, SER 402, TRP 524, VAL 529, VAL 532, VAL 533.
Pep2	−69.7+/− 1.5	ARG 373, ARG 526, **ARG 528**, ASN 534, ASP 565, GLN 570, GLU 399, GLU 637, GLY 446, GLY 514, GLY 527, GLY 562, GLY 569, HIS 564, ILE 401, ILE 405, ILE 525, ILE 563, **LYS 632**^S^, PRO 369, PRO 400, PRO 403, SER 402, SER 402, TRP 524, VAL 529, VAL 532, VAL 533.
Pep3	−70.4+/− 1.5	ARG 373, ARG 526, **ARG 528**, ASN 534, ASP 565, GLN 570, GLU 399, GLU 637, GLY 446, GLY 514, GLY 527, GLY 562, GLY 569, HIS 564, ILE 401, ILE 405, ILE 525, ILE 563, **LYS 632**^S^, PRO 369, PRO 400, PRO 403, SER 402, TRP 524, VAL 529, VAL 532, VAL 533.
Pep4	−71.1+/− 2.5	ARG 373, ARG 526, **ARG 528**, ASN 534, ASP 565, GLN 570, GLU 399, GLU 637, GLU 8, GLY 446, GLY 514, GLY 527, GLY 562, GLY 569, HIS 564, ILE 401, ILE 405, ILE 525, ILE 563**, LYS 632**^**S**^, PRO 369, PRO 400, PRO 403, SER 402, TRP 524, VAL 529, VAL 532, VAL 533.
Pep5	−72.6+/− 5.2	ARG 373, ARG 526, **ARG 528**, ASN 534, ASP 565, GLN 570, GLU 399, GLU 637, GLU 8, GLY 446, GLY 514, GLY 527, GLY 562, GLY 569, HIS 564, ILE 401, ILE 405, ILE 525, ILE 563**, LYS 632**^**S**^, PRO 369, PRO 400, PRO 403, SER 402, TRP 524, VAL 529, VAL 532, VAL 533.
Pep6	−74.4+/− 0.6	ARG 373, ARG 526, **ARG 528**, ASN 534, ASP 565, GLN 570, GLU 399, GLU 637, GLU 8, GLY 446, GLY 514, GLY 527, GLY 562, GLY 569, HIS 564, ILE 401, ILE 405, ILE 525, ILE 563**, LYS 632**^**S**^, PRO 369, PRO 400, PRO 403, SER 402, TRP 524, VAL 529, VAL 532, VAL 533.
Pep7	−76.4+/− 1.2	ARG 373, ARG 526, ARG 528, ARG 628, ARG 631, ASN 534, ASP 404, ASP 513, ASP 565, ASP 566, **GLN 570**, GLU 399, GLU 637, GLY 398, GLY 514, **GLY 527**, GLY 562, GLY 569, HIS 564, ILE 401, ILE 405, ILE 563, LYS 632, PRO 400, SER 402, **SER 635**, VAL 529.
Pep8	−79.2+/− 1.6	ARG 373, ARG 526, ARG 528, ARG 628, ARG 631, ASN 534, ASP 404, ASP 513, ASP 565, ASP 566, **GLN 570**, GLU 399, GLU 637, **GLY 398**, GLY 514, **GLY 527**, GLY 562, GLY 569, HIS 564, ILE 401, ILE 405, ILE 563, LYS 632, PRO 400, SER 402, **SER 635**, VAL 529.
Pep9	−80.7+/− 4.4	ARG 373, **ARG 526,** **ARG 528**, ASN 534, ASP 531, ASP 565, **ASP 566,** **GLN 570**, GLU 399, GLY 398, GLY 514, GLY 527, **GLY 537**, GLY 562, GLY 569, HIS 564, ILE 563, PRO 400, SER 402, SER 536, THR 370, THR 568, VAL 529, **VAL 532**, VAL 533, VAL 535.
Pep10	−82.4+/− 2.2	ARG 373, **ARG 526,** **ARG 528**, ASN 534, ASP 531, ASP 565, **ASP 566,** **GLN 570**, GLU 399, GLY 398, GLY 514, GLY 527, **GLY 537**, GLY 562, GLY 569, HIS 564, ILE 563, PRO 400, SER 402, SER 536, THR 370, THR 568, VAL 529, **VAL 532**, VAL 533, VAL 535.
Pep11	−83.9+/− 3.4	ARG 373, **ARG 526,** **ARG 528**, ASN 534, ASP 531, ASP 565, **ASP 566,** **GLN 570**, GLU 399, GLY 398, GLY 514, GLY 527, **GLY 537**, GLY 562, GLY 569, HIS 564, ILE 563, PRO 400, SER 402, SER 536, THR 370, THR 568, VAL 529, **VAL 532**, VAL 533, VAL 535.
Pep12	−87.2+/− 2.7	ARG 373, **ARG 526,** **ARG 528**, ASN 534, ASP 531, ASP 565, **ASP 566,** **GLN 570**, GLU 399, GLY 398, GLY 514, GLY 527, **GLY 537**, GLY 562, GLY 569, HIS 564, ILE 563, PRO 400, SER 402, SER 536, THR 370, THR 568, VAL 529, **VAL 532**, VAL 533, VAL 535.
Pep13	−87.3+/− 0.3	ARG 373, **ARG 526,** **ARG 528**, ASN 534, ASP 531, ASP 565, **ASP 566,** **GLN 570**, GLU 399, GLY 398, GLY 514, GLY 527, **GLY 537**, GLY 562, GLY 569, HIS 564, ILE 563, PRO 400, SER 402, SER 536, THR 370, THR 568, VAL 529, **VAL 532**, VAL 533, VAL 535.
Pep14	−87.7+/− 2.7	ARG 373, **ARG 526,** **ARG 528**, ASN 534, ASP 531, ASP 565, **ASP 566,** **GLN 570**, GLU 399, GLY 398, GLY 514, GLY 527, **GLY 537**, GLY 562, GLY 569, HIS 564, ILE 563, PRO 400, SER 402, SER 536, THR 370, THR 568, VAL 529, **VAL 532**, VAL 533, VAL 535.
Pep15	−89.0+/− 0.3	ARG 373, **ARG 526,** **ARG 528**, ASN 534, ASP 531, ASP 565, **ASP 566,** **GLN 570**, GLU 399, GLY 398, GLY 514, GLY 527, **GLY 537**, GLY 562, GLY 569, HIS 564, ILE 563, PRO 400, SER 402, SER 536, THR 370, THR 568, VAL 529, **VAL 532**, VAL 533, VAL 535.
Pep16	−91.1+/− 1.6	ARG 373, **ARG 526,** **ARG 528**, ASN 534, ASP 531, ASP 565, **ASP 566,** **GLN 570**, GLU 399, GLY 398, GLY 514, GLY 527, **GLY 537**, GLY 562, GLY 569, HIS 564, ILE 563, PRO 400, SER 402, SER 536, THR 370, THR 568, VAL 529, **VAL 532**, VAL 533, VAL 535.
AD-5584	−53.7+/− 0.7	TRP 320, ILE 321, THR 422, GLU 399, TYR 424, TRP 425, GLN 426, THR 427, VAL 397, GLY 398, ASP 513, VAL 532, VAL 533, ASN 534, SER 536, GLY 537, HIS 538, ARG 539, GLN 570, GLY 569, THR 568, IE 525, ARG 526, ARG 528, PRO 400.
ATP	−35.5+/− 1.4	VAL 397, GLN 426, TYR 424, **THR 423,** **ASP 422**, GLY 398, ALA 448, **THR 427**, TRP 425, GLU 399, PRO 400, **ARG 539**, ILE 525, **ASP 513**, ILE 321, ASN 534, **ARG 528**, GLY 527.

Bold: H-Bond, Superscript (^S^): Salt bridge. Other interactions are not shown

**Fig. 5:**
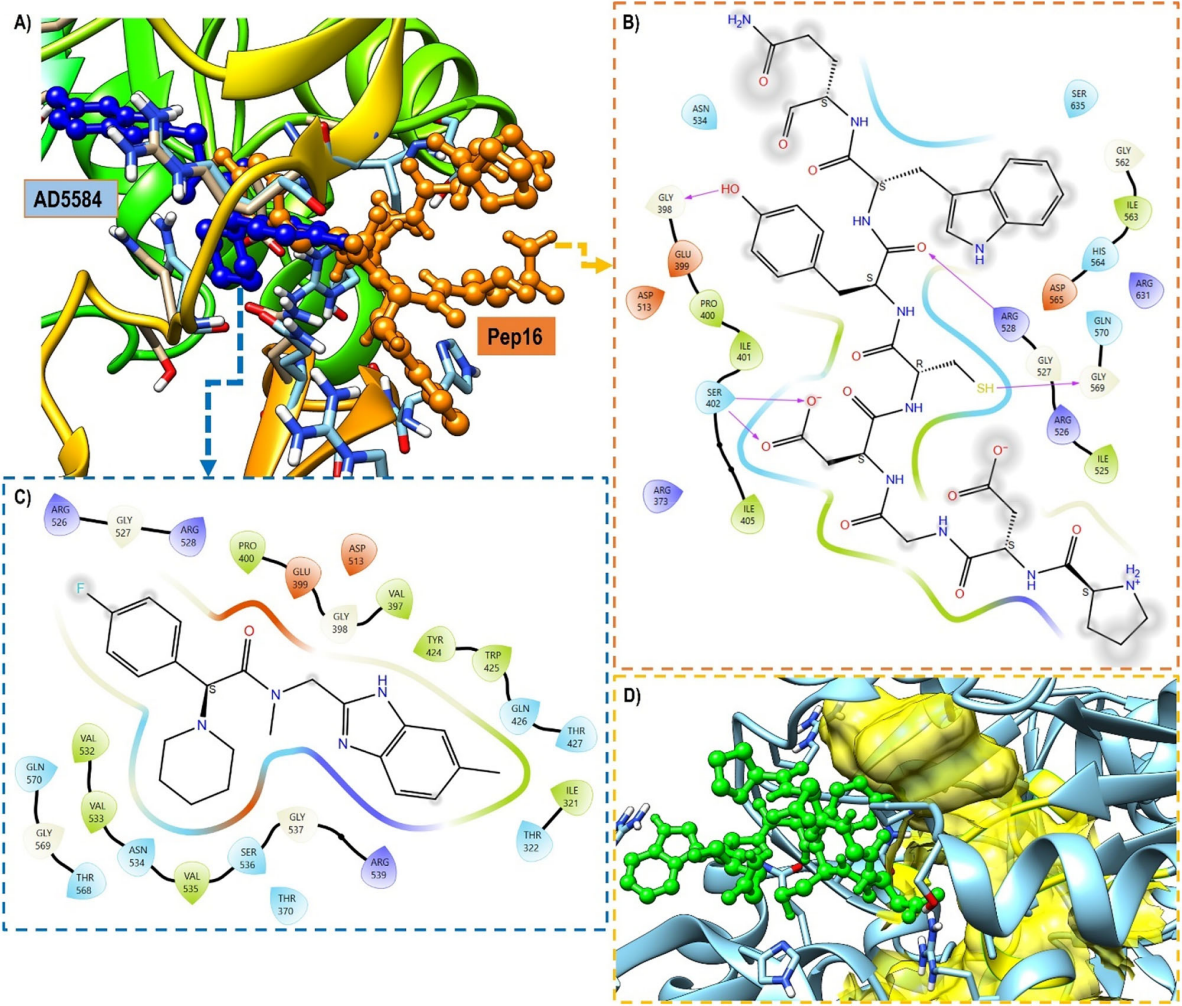
The binding mode of Pep16 and AD5584 to ACSS2. **A** The 3D structure of ACSS2 showing Pep16 (orange) and AD5584 (blue) in ball and sticks repreparation. Pep16 and AD5584 2d pose are shown in (**B**) and (**C**), respectively. Extenstion of Pep16 to some CoA pocket (**D**). Pep16 is shown in green ball and sticks, CoA bidng site as yellow surface

The HADDOCK scores ranged from −66.5 ± 3.7 to −91.1 ± 1.6 Kcal/mol, with an average value of −79.3. In comparison to the AD5584 (−53.7 ± 0.7), all top-16 PCS exhibited higher binding scores, with Pep16 almost double affinity (−91 versus −53 Kcal/mol) suggesting its potential as a new ACSS2 inhibitor. Furthermore, Pep16 scored more than a double compared to ATP (−91 versus −35 Kcal/mol), with contacts to residues that extend beyond ATP binding pocket. The 3D binding poses for Pep16 and AD5584 to ACSS2 is given in Fig. 5.

Having these findings, we speculate that Pep16 could possibly disrupts ACSS2 through completive effects. We also noted that PCS demonstrated some interaction with CoA site. Pep16’s docked pose in Fig. 8D illustrated an example for this observation.

### Structural and Conformational Analysis Revealed Enhanced ACSS2 Flexibility by Pep16

The catalytic conversion of acetate to acetyl-CoA is a two-step process that begins with ATP binding to ACSS2, hence current inhibitors have higher preference for the ATP pocket and completely block subsequent catalytic events. To understand and explain the observed affinity of Pep16 to ACSS2, we ran a 300 ns MD simulation and looked up the molecular events that occurred within ACSS2.

**Fig. 6:**
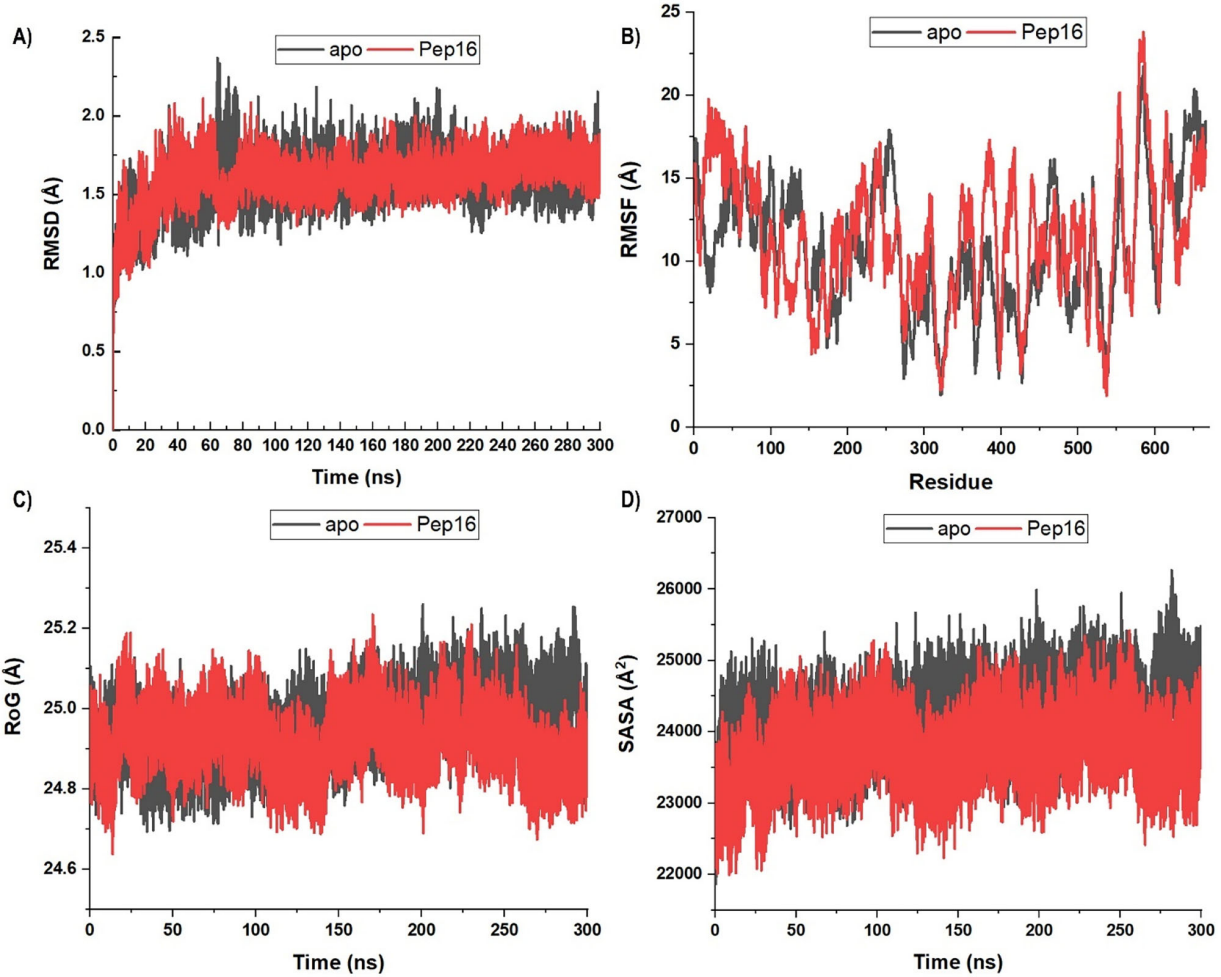
The C-α RMSD (**A**), RMSF (**B**), RoG (**C**) and SASA (**D**) of unbound ACSS2 (black) and Pep16 bound system (red) over 300 ns simulation. The graphs indicated overall lower stability, improved flexibility, decreased compactness and increased folding of the bound system

In doing so, we initially assessed ACSS2 structural stability, flexibility, and compactness by measuring the Root Mean Square Deviation (RMSD), Root Mean Square Fluctuation (RMSF), and Radius of Gyration (RoG). While RMSD primarily focuses on the stability of the protein backbone, RMSF and RoG are helpful in identifying dynamic events in proteins because they denote atomistic motions (flexibility) and structural compactness, respectively. The overall folding/unfolding behaviour during the simulation was estimated using the Solvent Accessible Surface Area (SASA). Figure 6 summarises the results of these metrics for unbound ACSS2 (apo), and Pep16-ACSS2.

The backbone RMSD trajectories for both apo and Pep16 systems (Fig. 5A) plateau after ~30–40 ns, stabilizing within a narrow range ( ± 0.3 Å) for the remaining simulation time. The absence of systematic drift and the limited fluctuations in RMSD after 40 ns indicate structural equilibration. The RMSD ranged from 0 to 2.36 Å with a mean value of 1.58 Å. Both systems exhibited almost similar RMSD values across the time. Overall, Pep16-ACSS2 had a slightly higher average RMSD of 1.60 Å compared to 1.57 Å for free ACSS2. This can suggest increased atomistic fluctuation and lower stability of ACSS2 due to Pep16 binding.

Overall protein flexibility was estimated using the RMSF metric, which ranged between 1.91 and 23.8 Å with an average of 13.49 Å. The Pep16 bound system experienced a higher residue flexibility and reduced rigidity (RMSF of 11.44 versus 10.82 Å). Furthermore, structural compactness was almost similar across the simulation averaging around 25 Å as denoted by RoG. Nevertheless, from 200 ns onward, the Pep16 bound system witnessed reduced compactness and adopted a loose conformation than unbound ACSS2. These data pinpoint reduced stability and increased conformational flexibility of ACSS2 due to Pep16. Continuing to probe the binding dynamics of Pep16, a study of SASA has been conducted. The SASA values match the folding and unfolding of the target system. As illustrated, the apo system steadily had higher SASA values throughout the simulation’s interval, with an average of 24,223.66 Å^2^. The Pep16 bound ACSS2, on the other hand, exhibited lower SASA averaging around 23,744.97 Å^2^; indicative of increased folding behaviour of of Pep16 bound system.

**Fig. 7:**
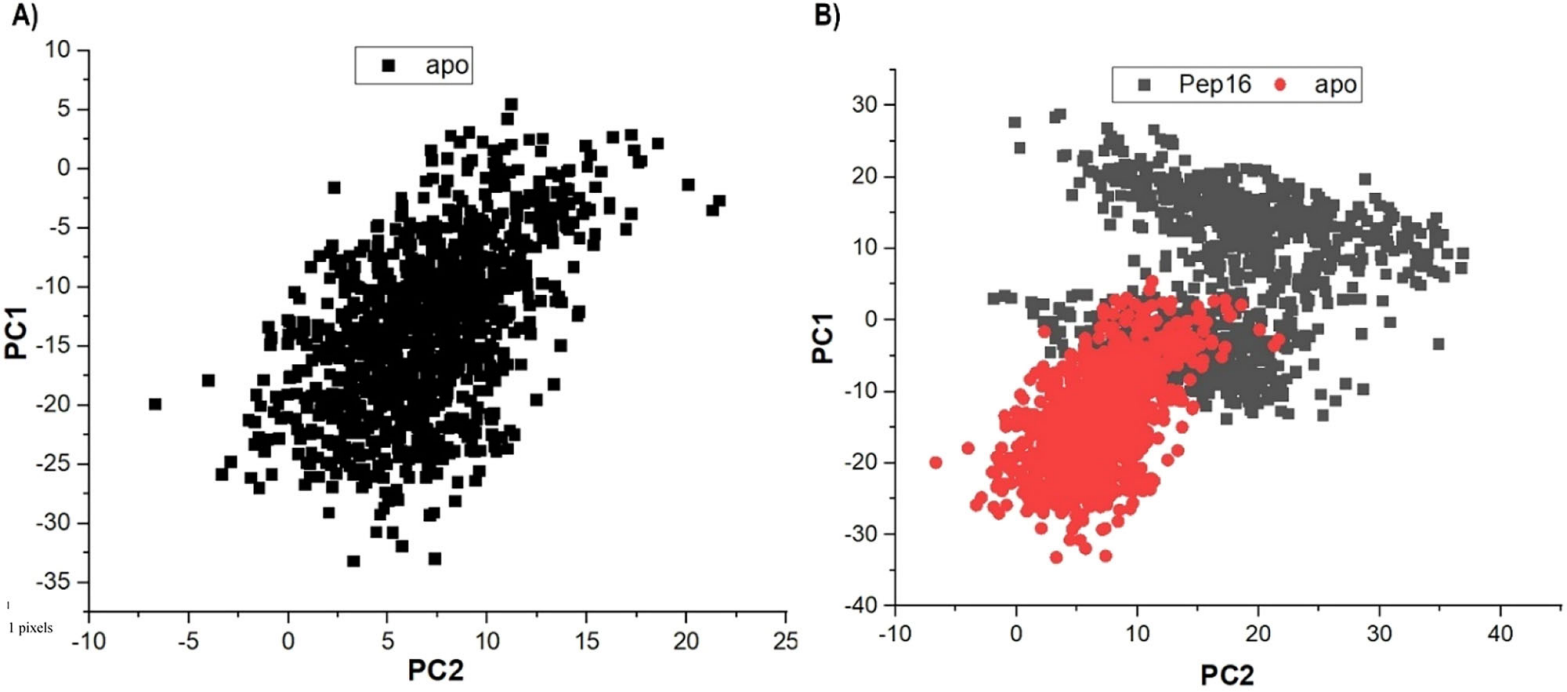
Graph of PCA showing the degree of C‐α atomic conformational changes in free ACSS2 (**A**) and Pep16 bound system (**B**). PCA results show restricted residue displacements in apo compared to Pep16-ACSS2

Finally, the degree of C‐α atom displacements of ACSS2 across the simulation was assessed using the principal component analysis (PCA). The two principal components (PC1 and PC2) represent the conformational dynamics of the MD trajectories computed from the covariance matrix concerning the first two eigenvectors (ev1 and ev2) [58, 66]. Substantial conformational fluctuations were observed for Pep16 bound ACSS2 versus the unbound ACSS2, as a measure of their computed eigenvectors (Fig. 7).

### Pep16-ACSS2 Thermodynamic Profiling and Binding Pattern Analysis: Larger Interaction Interface and Tighter Binding

**Fig. 8:**
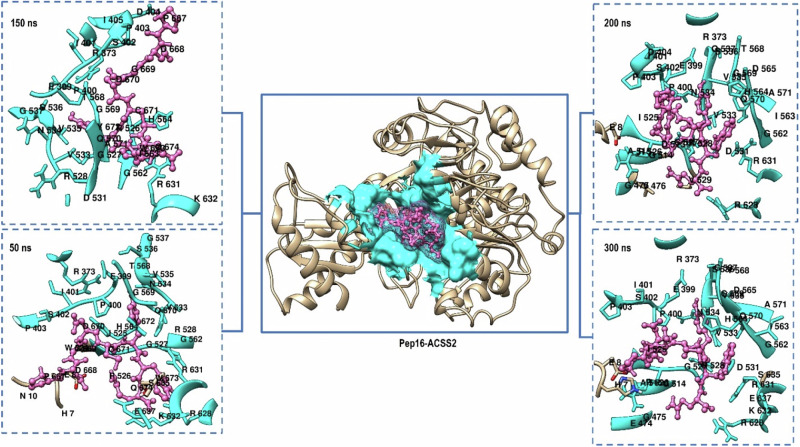
3D binding poses of Pep16-ACSS2 visualized at 50 ns, 150 ns, 200 ns, and 300 ns MD trajectory snapshots. The binding pocket is shown in cyan, and Pep16 in pink (ball and stick)

To affirm improved binding affinity and understand the Pep16 binding pattern to ACSS2, we thoroughly examined several MD trajectories across the simulation. We also calculated the binding free energy of Pep16 and compared it to AD5585, followed by an estimation of the residual energy contributions of the binding residues to the stabilization of Pep16 (PRED analysis). Snapshot analysis revealed that Pep16 occupies a larger binding site consisting of 34 residues (Table 3); thereby maintaining tighter binding to ACSS2. Figure 8 illustrates the 3D binding modes of Pep15 alongside its binding pocket residues.

**Table 3:** Residue interacting framework of Pep16 with ACSS2 at different MD time steps

MD snapshot	Interacting Residues (within 5 Å)
50 ns	373, 399, 400, 401, 402, 403, 525, 526, 527, 528, 533, 534, 535,536, 537, 562, 564, 568, 569, 570, 628, 631, 632, 635, 637.
150 ns	373, 401, 402, 403, 404, 405, 399, 400, 528, 526, 527, 531, 533, 534, 535, 536, 537, 562, 564, 563, 568, 569, 570, 571, 631, 632.
200 ns	8, 373, 399, 400, 401, 402, 403, 404, 475, 476, 513, 514, 515, 525, 526, 527, 528, 529, 531, 533, 534, 535, 536, 537, 562, 563, 564, 565, 568, 569, 570, 571, 626, 631.
250 ns	8, 10, 373, 399, 400, 401, 402, 403, 404, 405, 446, 474, 514, 524, 526, 527, 528, 531, 533, 534, 536, 537, 42, 562, 563, 564, 565, 568, 569, 570, 571, 572, 628, 631, 637.
300 ns	373, 399, 400, 401, 402, 403, 474, 475, 514, 515, 525, 526, 527, 528, 531, 533, 534, 536, 537, 562, 563, 564, 565, 568, 569, 570, 571, 628, 631, 632, 636, 637.
Conserved residues	373, 399, 400, 401, 402, 403, 404, 405, 474, 475, 514, 515, 525, 526, 527, 528, 531, 533, 534, 535, 536, 537, 562, 563, 564, 565, 568, 569, 570, 571, 628, 631, 632, 637.

Using the generalized Born surface area/molecular mechanics (MM/GBSA) approach, the Binding Free Energy (BFE) profiles of the molecular connections between Pep16 and ACSS2 were assessed. Analyzing 250,000 frames from stabilized MD trajectories allowed for this to be accomplished [67, 68]. BFE gave excellent insights into the contribution of the interaction energy components for Pep16-ACSS2, providing a measure of the expected binding affinity. A Generalized Born (GB) implicit solvent model and a Solvent Accessible (SA) model were used to compute the solvation energy in MM/GBSA. The solute-continuum solvent electrostatic interactions are taken into account in the GB model to mimic the solvent effect. To take into consideration the nonpolar solvation effects, the solvation energy was also computed by the SA model. As shown in Table 4, the Pep16-ACSS2 complex demonstrated more favourable total binding free energy compared to the AD5584-ACSS2. These findings highlight the potential of Pep16 as a novel peptide-based inhibitor for ACSS2, justifying additional experimental validation and optimization.

**Table 4:** Binding free energy terms of Pep16-ACSS2 and AD5584-ACSS2 complexes expressed in kcal/mol with the standard error of mean

Energy components (kcal/mol)							
System	ΔE_vdw_	Δ_Eele_	ΔG_GB_	ΔG_SA_	ΔG_gas_	ΔG_sol_	ΔG_bind_
Pep16-ACSS2	−60.14 ± 0.12	248.63 ± 114	−212.08 ± 1.00	−7.99 ± 0.01	188.58 ± 1.09	−220.07 ± 1.00	−31.48 ± 0.16
AD5584-ACSS2	−39.51 ± 0.10	−246.36 ± 0.46	264.97 ± 0.01	−5.0 ± 0.48	−285.58 ± 0.52	259.94 ± 0.47	−25.94 ± 0.10

∆E_elec_ (electrostatic energy), ∆E_vdW_ (van der Waals energy), ∆G_GB_ (polar solvation energy), ∆G_SA_ (non-polar solvation energy), ∆G_gas_ (gas-phase energy), ∆G_solv_ (Total solvation free energy of polar and non-polar states), and ∆G_bind_ (total free binding energy)

Added to the thermodynamic profiling of molecular interactions by the MM/GBSA technique, PRED was used to estimate the individual residue energy contributions of the Pep16-ACSS2 to the overall BFE (Fig. 8). PRED estimates are useful for determining the impact of electrostatic and van der Waals energies [41, 54, 58, 69].

Energy decomposition revealed that the electrostatic interaction energy was for more significant and higher than the van der Waals energy. As illustrated, Pep16 had a larger binding landscape. In the Pep16-ACSS2 complex, residues ARG 628, ARG 631, LYS 632, and ARG 526 were the most constitutors to ∆E_ele_ with −49.686, −47.586 and −44.579 Kcal/mol respectively, while ARG 526, ARG 373, and ARG 528 had lower ∆E_ele_ values of −34.86, −33.149 and −22.995 Kcal/mol respectively. Additionally, multiple conventional hydrogen bonds were established (SER 402, HIS 564, and ARG 628). Added to these fundamental interactions, van der Waals and other non-bonded interactions formed the Pep16 residue interaction network and maintained its stabilization and binding to ACSS2.

### Pep16 Conformationally Locks ACSS2 Nucleotide Binding Motif: The Potential for Firm Inhibition

**Fig. 9:**
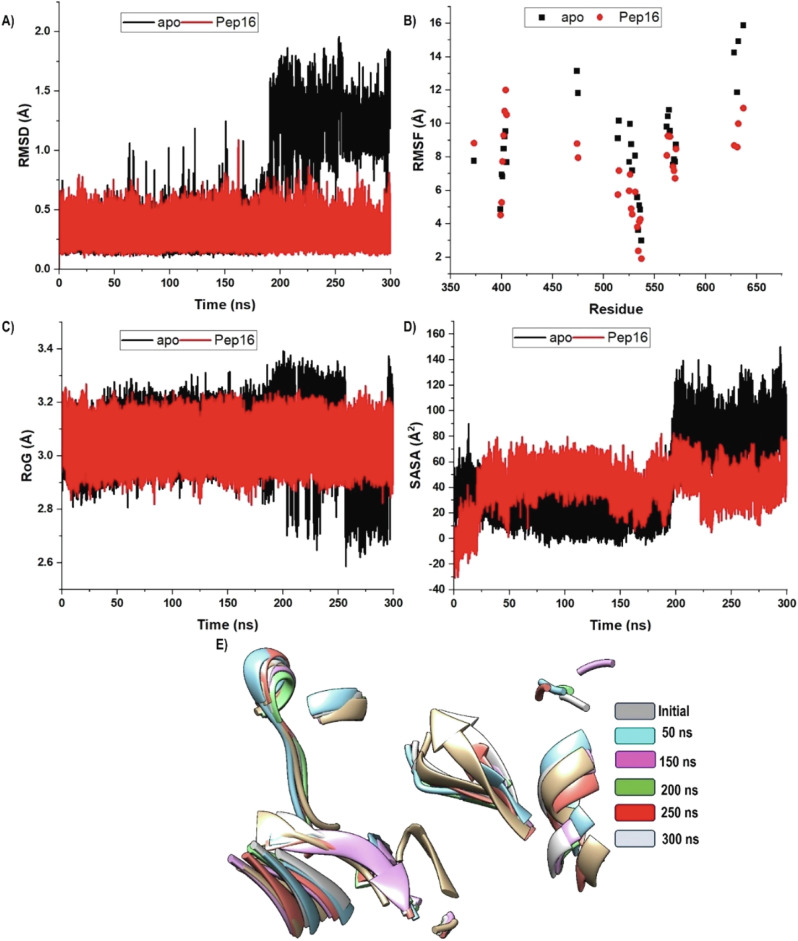
The C-α RMSD (**A**), RMSF (**B**), RoG (**C**), SASA (**D**), as well as the conformational poses (**E**) of the Pep16 binding pocket. The graphs indicated overall lower stability, improved flexibility, decreased compactness and increased folding of the bound system

As demonstrated, Pep16 exhibited good binding affinity to ACSS2 and occupied a large binding landscape of ACSS2. To understand the binding pattern and subsequent blockade of ACSS2 catalytic functionality, we aimed to study conformational dynamics and structural disparities of the binding pocket. Overall stability, flexibility and compactness of binding residues were assessed following RMSD, RMSF, RoG and SASA metrics (Fig. 9).

While maintaining similar RMSD from the start of the simulation, unbound protein spiked at 180 ns forward compared to Pep16-ACSS2 which held lower RMSD values (Fig. 9A). Overall, the apo system achieved twice-fold RMSD values compared to the Pep16-ACSS2 complex with a mean of 0.63 and 0.30 Å respectively. These results symbolise the improved structural stability and lower fluctuations experienced by binding pocket residues.

**Fig. 10:**
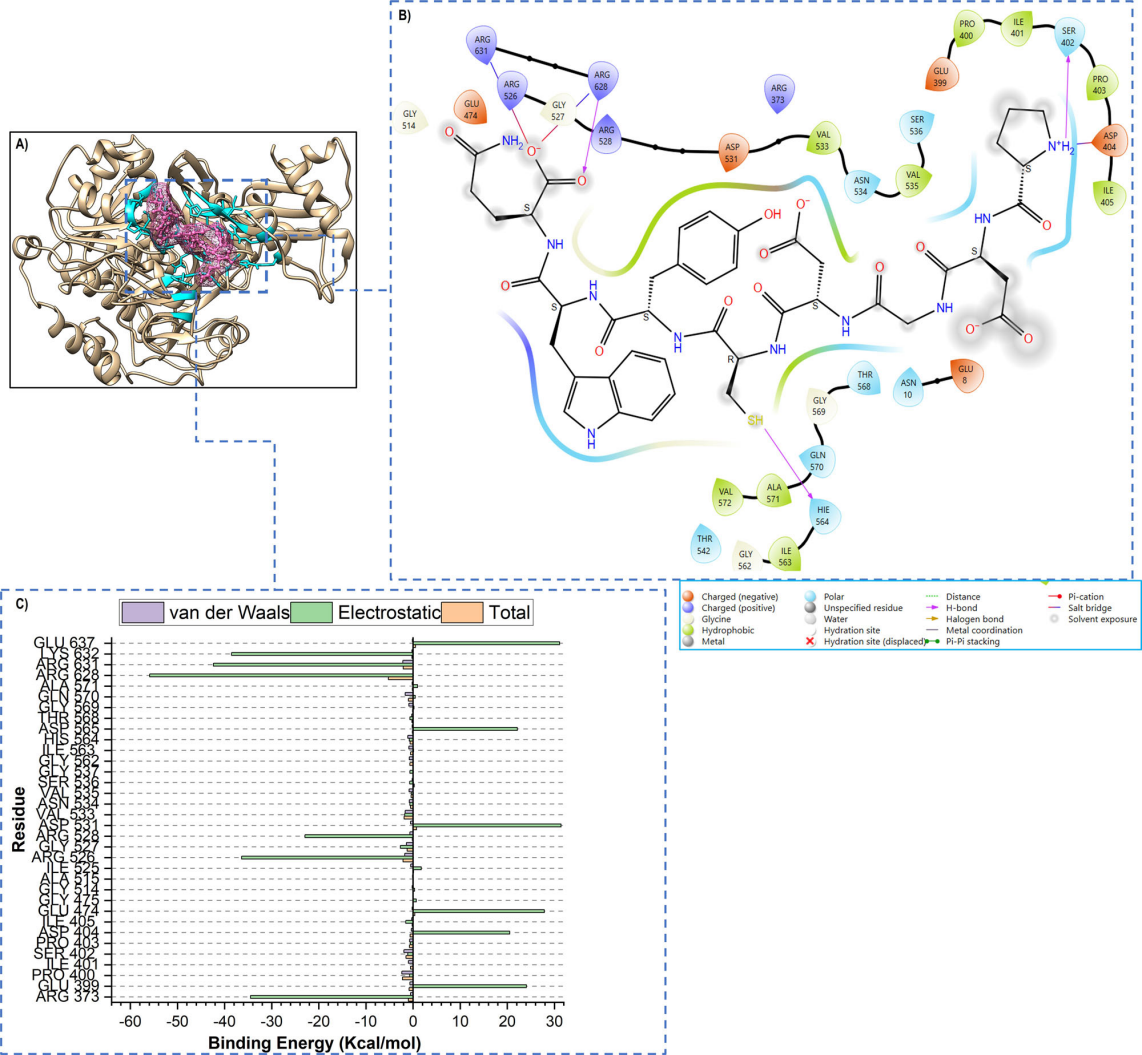
Representation of the (**A**) 3D binding poses of Pep16-ACSS2, (**B**) 2D Pep16-ACSS2 interactions and (**C**) the PRED analysis of interacting residues framework at 250 ns MD trajectory snapshots

As shown in Fig. 9B, the binding pocket in Pep16-ACSS2 scored lower RMSF values compared to its apo counterpart. These findings are suggestive of reduced flexibility and higher rigidity; reiterating the RMSD results of potentiated structural stability. Furthermore, the binding pocket adopted a more tight and compact conformation in Pep16-ACSS2 as depicted by RoG (Fig. 9C) and SASA (Fig. 9D) measures. Time-scale conformational analysis of the binding pocket (Fig. 10E) further highlighted a substantial conformational shift; confirming the overall induced dynamical perturbation by Pep16.

**Fig. 11:**
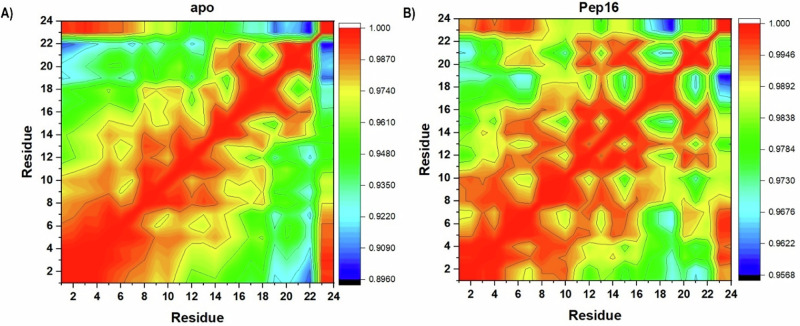
DCCM graph illustrating the degree of relative inter‐C‐α residue motions within Pep16 binding residues of (**A**) apo and (**B**) Pep16-ACSS2 complex. Legends on the right panel of each graph show the degree of correlated motions

Furthermore, we constructed the Dynamic Cross-Correlation Matrix (DCCM) which is useful in measuring the strength and relative position of the C‐α atoms of the binding pocket in free ACSS2 and Pep16‐ACSS2 across the simulations. Measuring conformational dynamics and inter-residue movements during ligand binding can help determine the effectiveness of ligand inhibition [54, 58]. DCCM scales from -1 (complete negative), 0 (no correlation), or +1 (complete positive) correlation of residue movement, represented by cyan to black, light green to green, and red to yellow contours, respectively (Fig. 11). According to DCCM, binding residue frameworks experience more positively correlated motions upon Pep16 binding, implicating their adoption of conformationally more compact status.

**Fig. 12:**
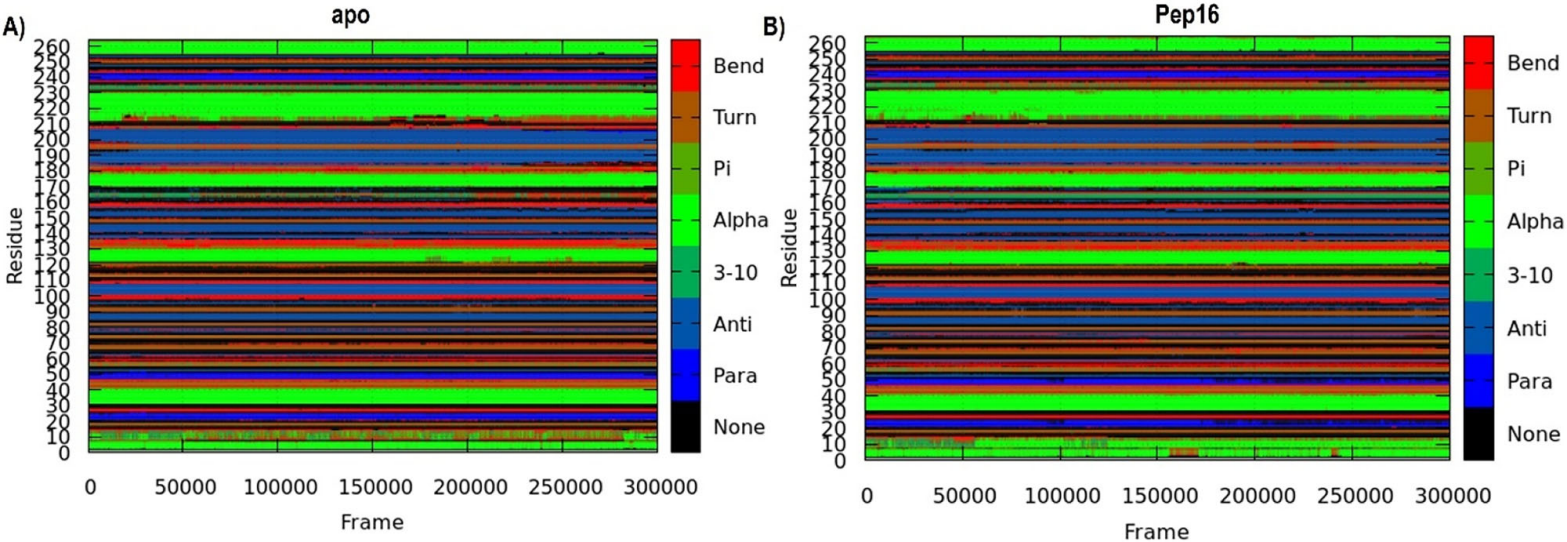
Graphical plots of DSSP presentation of the secondary structure changes of the C-α residues of (**A**) ACSS2, and Pep16-ACSS2 complex (**B**). The coloured legends on the right panel of each graph depict the secondary structure assignments

To gain more insights into the potential inhibitory effects of Pep16, ACSS2 secondary structure perturbation was examined using the Dictionary of Protein Secondary Structure (DSSP) analysis (Fig. 12). Using integers (from 0 to 7) to indicate None, Para, Anti, 3–10, Alpha, Pi, Turn, and Bend, respectively, each residue is categorized according to its main secondary structure in the DSSP approach, as shown in the image legend [41, 54, 58]. This description is dependent on estimates of the hydrogen bond interactions and atomic coordinates [70]. The conversion into turn, bend, and pi-helix structures suggest a potential reduction in hydrogen bonds compared with transitions into beta sheets, alpha helices, and 3–10 helices. Both free and bound systems shared some significant similarities and distinctions in their secondary structure while conserving the architectural integrity of the ACSS2 secondary structure. Residues 1–10 transitioned from bend states in the apo into more stable alpha states just after the first 50 ns of the simulation forward. A similar transition was observed for residues 120–130 and 160–170 after 150 and 50 ns respectively, maintaining a more stable secondary structure across the simulation. Nevertheless, residues 230–240 shifted from Pi and 3–10 helices in unbound ACSS2 to the turn structures.

Compared to free ACSS2, the Pep16-bound system had more residues adopting the alpha states with enhanced formation of intermolecular hydrogen bonds rationalizing the structurally more compact and rigid conformation of the pocket residues throughout the simulation.

Current Acetyl-CoA Synthetase 2 (ACSS2) inhibitors have limited pharmacokinetic data [12, 71]. Additionally, specificity challenges persist due to structural similarities among ACSS isoforms (ACSS1, ACSS2, ACSS3), raising risks of cross-reactivity and off-target side effects despite claims of selectivity for ACSS2. Metabolic dysregulation [11, 12]. These findings underscore the need for rigorous validation of isoform selectivity, tumor-specific efficacy, and long-term safety in clinical translation. Jointly, our findings outline that Pep16 possibly lock the nucleotide site in a compact and rigid state, preventing ATP binding and subsequent catalytic events, yet, further investigations and comparison to existing inhibitors are to be made. The built-in peptides limitations could also be avoided/reduced in many ways. The C-terminal functionalization enables selective modifications to enhance solubility and resistance to proteolytic degradation [72], leveraging pharmacokinetics and bioavailability profiles. Formulation strategies such as encapsulation within polymeric nanoparticles (e.g., PLGA or PEGylated liposomes) can further enhance tumor targeting via the enhanced permeability and retention (EPR) [73].

Above all, predicted therapeutic utility of Pep16 can be expanded by synergy with existing therapies though rational combination strategies that can even sensitize tumors to chemotherapy [73, 74]. Moreover, integrating peptides with antibody-drug conjugates (ADCs) or nanoparticle-based co-delivery systems could amplify therapeutic efficacy while minimizing off-target effects [75].

## Conclusion

Studies have demonstrated the link between metabolic stress and ACSS2 overexpression in many cancers; hence, it has attracted attention as a novel anticancer therapeutic. In this regard, significant research efforts have been allocated to inhibiting ACSS2-mediated acetate production, ultimately potentiating tumour growth and survival. Despite lacking full pharmacokinetics characterization, known selective ACSS2 inhibitors act primarily through the interference of ATP binding to the nucleoside region within ACSS2 and subsequently block its catalytic activity. Our study’s objective was to apply our established methodical approach for developing a peptide-based ACSS2 inhibitor endowed with peptide drugs attractiveness of improved selectivity, efficacy and safety prospectives.

Following our protocol, we first mapped the nucleoside motif of ACSS2 and produced a sizable collection of 3600 non-repeating peptide combination sequences (PCS). The PCS were screened for toxicity and filtered based on amphipathicity, hydrophobicity, molecular weight, and in vivo aggregation. We identified 16 promising PCS and optimized them to achieve the most energy-efficient folding conformations. We started our strategy to block ACSS2 by performing docking studies, which showed that all 16 PCS had higher binding scores than AD5584, the reference molecule. These higher binding scores were linked to a larger interaction landscape.

Next, we explored the conformational dynamics of ACSS2 upon Pep16 binding, the top PCS. Interestingly, our analysis indicated that Pep16 induced noticeable conformational events, mainly increased atomistic fluctuations and flexibility. Furthermore, the structural analysis revealed that the Pep16-ACSS2 complex had a more compact structure with a potentially buried surface, indicating enhanced structural stability and reduced conformational variability compared to free protein. Furthermore, we looked at Pep16’s interaction patterns and found that it continuously interacted with a significant number of residues during the MD simulation, outpacing the reference inhibitor’s interaction frequency and providing an explanation for its increased binding. Based on the MM/GBSA approach, the electrostatic energy term principally contributed to the stabilization of Pep16, which expectedly had a higher free energy score than the AD5584. Complemented by PRED analysis, ARG 373, ARG 526, ARG 628, ARG 631, and LYS 632 residues of ACSS2 were the dominant contributors to Pep16 binding.

To obtain insights on Pep16’s potential for ACSS2 inhibition, we further conducted a conformational and structural analysis of Pep16 the binding pocket. While Pep16 seems to potentiate ACSS2 flexibility and reduce its stability, the binding pocket underwent a reverse trend; experiencing more stable, rigid and compact conformations. Based on these findings, we advocate that Pep16 can block ATP binding and interfere with the catalytic events that follow.

Collectively, our data underline the potential of Pep16 inhibitory efficacy against ACSS2 and we urge further research to confirm these results through clinical analysis. Advances in peptides functionalization, formulation strategies and implantation of rational drug combination could further its probable therapeutics potential.

## Supplementary information

Supplementary Tables

## Data Availability

All data can be made available upon request to the Corresponding Author.

## References

[1] Xu, H., Luo, J., Ma, G., Zhang, X., Yao, D., Li, M., & Loor, J. J. (2018). Acyl-CoA synthetase short-chain family member 2 (ACSS2) is regulated by SREBP-1 and plays a role in fatty acid synthesis in caprine mammary epithelial cells. *Journal of Cellular Physiology*, *233*(2), 1005–1016. 28407230 10.1002/jcp.25954

[2] Brown, M. S., & Goldstein, J. L. (1997). The SREBP pathway: regulation of cholesterol metabolism by proteolysis of a membrane-bound transcription factor. *Cell*, *89*(3), 331–340. 9150132 10.1016/s0092-8674(00)80213-5

[3] Moffett, J. R., Arun, P., Ariyannur, P. S., & Namboodiri, A. M. A. (2013). N-Acetylaspartate reductions in brain injury: Impact on post-injury neuroenergetics, lipid synthesis, and protein acetylation. *Frontiers in Neuroenergetics*, *5*, 11. 24421768 10.3389/fnene.2013.00011 PMC3872778

[4] Chen, R., Xu, M., Nagati, J., & Garcia, J. A. (2017). Coordinate regulation of stress signaling and epigenetic events by Acss2 and HIF-2 in cancer cells. *PLoS ONE*, *12*(12), e0190241. 29281714 10.1371/journal.pone.0190241 PMC5744998

[5] Huang, Z., Zhang, M., Plec, A. A., Estill, S. J., Cai, L., Repa, J. J., McKnight, S. L., & Tu, B. P. (2018). ACSS2 promotes systemic fat storage and utilization through selective regulation of genes involved in lipid metabolism. *Proceedings of the National Academy of Sciences of the United States of America*, *115*(40), E9499–E9506. 30228117 10.1073/pnas.1806635115 PMC6176566

[6] Schug, Z. T., Peck, B., Jones, D. T., Zhang, Q., Grosskurth, S., Alam, I. S., Goodwin, L. M., Smethurst, E., Mason, S., Blyth, K., McGarry, L., James, D., Shanks, E., Kalna, G., Saunders, R. E., Jiang, M., Howell, M., Lassailly, F., Thin, M. Z., & Gottlieb, E. (2015). Acetyl-CoA synthetase 2 promotes acetate utilization and maintains cancer cell growth under metabolic stress. *Cancer Cell*, *27*(1), 57–71. 25584894 10.1016/j.ccell.2014.12.002 PMC4297291

[7] Moffett, J. R., Puthillathu, N., Vengilote, R., Jaworski, D. M., & Namboodiri, A. M. (2020). Acetate revisited: A key biomolecule at the nexus of metabolism, epigenetics, and oncogenesis - part 2: Acetate and ACSS2 in health and disease. *Frontiers in Physiology*, *11*, 580171. 33304273 10.3389/fphys.2020.580171 PMC7693462

[8] Yoshii, Y., Waki, A., Furukawa, T., Kiyono, Y., Mori, T., Yoshii, H., Kudo, T., Okazawa, H., Welch, M. J., & Fujibayashi, Y. (2009). Tumor uptake of radiolabeled acetate reflects the expression of cytosolic acetyl-CoA synthetase: Implications for the mechanism of acetate PET. *Nuclear Medicine and Biology*, *36*(7), 771–777. 19720289 10.1016/j.nucmedbio.2009.05.006

[9] Gan, S., Mao, J., Pan, Y., Tang, J., & Qiu, Z. (2021). hsa-miR-15b-5p regulates the proliferation and apoptosis of human vascular smooth muscle cells by targeting the ACSS2/PTGS2 axis. *Experimental and Therapeutic Medicine*, *22*(5), 1208. 34584553 10.3892/etm.2021.10642 PMC8422401

[10] Mews, P., Donahue, G., Drake, A. M., Luczak, V., Abel, T., & Berger, S. L. (2017). Acetyl-CoA synthetase regulates histone acetylation and hippocampal memory. *Nature*, *546*(7658), 381–386. 28562591 10.1038/nature22405 PMC5505514

[11] Ling, R., Chen, G., Tang, X., Liu, N., Zhou, Y., & Chen, D. (2022). Acetyl-CoA synthetase 2(ACSS2): a review with a focus on metabolism and tumor development. *Discover Oncology*, *13*(1), 58. 35798917 10.1007/s12672-022-00521-1 PMC9263018

[12] Liu, M., Liu, N., Wang, J., Fu, S., Wang, X., & Chen, D. (2022). Acetyl-CoA synthetase 2 as a therapeutic target in tumor metabolism. *Cancers (Basel)*, *14*(12), 2896. 35740562 10.3390/cancers14122896 PMC9221533

[13] Kamphorst, J. J., Chung, M. K., Fan, J., & Rabinowitz, J. D. (2014). Quantitative analysis of acetyl-CoA production in hypoxic cancer cells reveals substantial contribution from acetate. *Cancer & Metabolism*, *2*(1), 23. 25671109 10.1186/2049-3002-2-23 PMC4322440

[14] Yang, X., Shao, F., Shi, S., Feng, X., Wang, W., Wang, Y., Guo, W., Wang, J., Gao, S., Gao, Y., Lu, Z., & He, J. (2019). Prognostic impact of metabolism reprogramming markers Acetyl-CoA synthetase 2 phosphorylation and ketohexokinase-a expression in non-small-cell lung carcinoma. *Frontiers in Oncology*, *9*, 486002. 10.3389/fonc.2019.01123 PMC6848158 31750240

[15] Li, X., Yu, W., Qian, X., Xia, Y., Zheng, Y., Lee, J. H., Li, W., Lyu, J., Rao, G., Zhang, X., Qian, C. N., Rozen, S. G., Jiang, T., & Lu, Z. (2017). Nucleus-translocated ACSS2 promotes gene transcription for lysosomal biogenesis and autophagy. *Molecular Cell*, *66*(5), 684–697.e9. 28552616 10.1016/j.molcel.2017.04.026 PMC5521213

[16] Gao, X., Lin, S. H., Ren, F., Li, J. T., Chen, J. J., Yao, C. B., Yang, H. B., Jiang, S. X., Yan, G. Q., Wang, D., Wang, Y., Liu, Y., Cai, Z., Xu, Y. Y., Chen, J., Yu, W., Yang, P. Y., & Lei, Q. Y. (2016). Acetate functions as an epigenetic metabolite to promote lipid synthesis under hypoxia. *Nature Communications*, *7*, 11960. 27357947 10.1038/ncomms11960 PMC4931325

[17] Comerford, S. A., Huang, Z., Du, X., Wang, Y., Cai, L., Witkiewicz, A. K., Walters, H., Tantawy, M. N., Fu, A., Manning, H. C., Horton, J. D., Hammer, R. E., McKnight, S. L., & Tu, B. P. (2014). Acetate dependence of tumors. *Cell*, *159*(7), 1591–1602. 25525877 10.1016/j.cell.2014.11.020 PMC4272450

[18] Miller, K. D., Pniewski, K., Perry, C. E., Papp, S. B., Shaffer, J. D., Velasco-Silva, J. N., Casciano, J. C., Aramburu, T. M., Srikanth, Y., Cassel, J., Skordalakes, E., Kossenkov, A. V., Salvino, J. M., & Schug, Z. T. (2021). Targeting ACSS2 with a transition-state mimetic inhibits triple-negative breast cancer growth. *Cancer Research*, *81*(5), 1252–1264. 33414169 10.1158/0008-5472.CAN-20-1847 PMC8026699

[19] Stine, Z. E., Schug, Z. T., Salvino, J. M., & Dang, C. V. (2022). Targeting cancer metabolism in the era of precision oncology. *Nature Reviews. Drug Discovery*, *21*(2), 141–162. 34862480 10.1038/s41573-021-00339-6 PMC8641543

[20] Sabnis, R. W. (2021). Amide-substituted condensed pyridine derivatives as ACSS2 inhibitors for treating cancer. *ACS Medicinal Chemistry Letters*, *12*(12), 1870–1871. 34917239 10.1021/acsmedchemlett.1c00571 PMC8667058

[21] Sabnis, R. W. (2021). Novel Substituted Tetrazoles as ACSS2 Inhibitors for Treating Cancer. *ACS Medicinal Chemistry Letters*, *12*(12), 1894–1895. 34917250 10.1021/acsmedchemlett.1c00621 PMC8667299

[22] Cherkasov, A., Muratov, E. N., Fourches, D., Varnek, A., Baskin, I. I., Cronin, M., Dearden, J., Gramatica, P., Martin, Y. C., Todeschini, R., Consonni, V., Kuz’min, V. E., Cramer, R., Benigni, R., Yang, C., Rathman, J., Terfloth, L., Gasteiger, J., Richard, A., & Tropsha, A. (2014). QSAR modeling: where have you been? Where are you going to? *Journal of Medicinal Chemistry*, *57*(12), 4977–5010. 24351051 10.1021/jm4004285 PMC4074254

[23] Meng, X. Y., Zhang, H. X., Mezei, M., & Cui, M. (2011). Molecular docking: a powerful approach for structure-based drug discovery. *Current Computer-Aided Drug Design*, *7*(2), 146–157. 21534921 10.2174/157340911795677602 PMC3151162

[24] Wager, T. T., Hou, X., Verhoest, P. R., & Villalobos, A. (2010). Moving beyond rules: the development of a central nervous system multiparameter optimization (CNS MPO) approach to enable alignment of druglike properties. *ACS Chemical Neuroscience*, *1*(6), 435–449. 22778837 10.1021/cn100008c PMC3368654

[25] Sliwoski, G., Kothiwale, S., Meiler, J., & Lowe, E. W. (2013). Computational methods in drug discovery. *Pharmacological Reviews*, *66*(1), 334–395. 24381236 10.1124/pr.112.007336 PMC3880464

[26] Genheden, S., & Ryde, U. (2015). The MM/PBSA and MM/GBSA methods to estimate ligand-binding affinities. *Expert Opinion on Drug Discovery*, *10*(5), 449–461. 25835573 10.1517/17460441.2015.1032936 PMC4487606

[27] Kumar, A., Rajendran, V., Sethumadhavan, R., & Purohit, R. (2014). Relationship between a point mutation S97C in CK1δ protein and its affect on ATP-binding affinity. *Journal of Biomolecular Structure & Dynamics*, *32*(3), 394–405. 23527964 10.1080/07391102.2013.770373

[28] Gopalakrishnan, C., Kamaraj, B., & Purohit, R. (2014). Mutations in microRNA binding sites of CEP genes involved in cancer. *Cell Biochemistry and Biophysics*, *70*(3), 1933–1942. 25115610 10.1007/s12013-014-0153-8

[29] Kamaraj, B., Rajendran, V., Sethumadhavan, R., & Purohit, R. (2013). In-silico screening of cancer associated mutation on PLK1 protein and its structural consequences. *Journal of Molecular Modeling*, *19*(12), 5587–5599. 24271645 10.1007/s00894-013-2044-0

[30] Kalsi, N., Gopalakrishnan, C., Rajendran, V., & Purohit, R. (2016). Biophysical aspect of phosphatidylinositol 3-kinase and role of oncogenic mutants (E542K & E545K). *Journal of Biomolecular Structure & Dynamics*, *34*(12), 2711–2721. 26646651 10.1080/07391102.2015.1127774

[31] Sharma, B., Bhattacherjee, D., Zyryanov, G. V., & Purohit, R. (2023). An insight from computational approach to explore novel, high-affinity phosphodiesterase 10A inhibitors for neurological disorders. *Journal of Biomolecular Structure & Dynamics*, *41*(19), 9424–9436. 36336960 10.1080/07391102.2022.2141895

[32] Wu, K., Bai, H., Chang, Y. T., Redler, R., McNally, K. E., Sheffler, W., Brunette, T. J., Hicks, D. R., Morgan, T. E., Stevens, T. J., Broerman, A., Goreshnik, I., DeWitt, M., Chow, C. M., Shen, Y., Stewart, L., Derivery, E., Silva, D. A., Bhabha, G., & Baker, D. (2023). De novo design of modular peptide-binding proteins by superhelical matching. *Nature*, *616*(7957), 581–589. 37020023 10.1038/s41586-023-05909-9 PMC10115654

[33] Vázquez Torres, S., Leung, P. J. Y., Venkatesh, P., Lutz, I. D., Hink, F., Huynh, H. H., Becker, J., Yeh, A. H., Juergens, D., Bennett, N. R., Hoofnagle, A. N., Huang, E., MacCoss, M. J., Expòsit, M., Lee, G. R., Bera, A. K., Kang, A., De La Cruz, J., Levine, P. M., & Baker, D. (2024). De novo design of high-affinity binders of bioactive helical peptides. *Nature*, *626*(7998), 435–442. 38109936 10.1038/s41586-023-06953-1 PMC10849960

[34] London, N., Movshovitz-Attias, D., & Schueler-Furman, O. (2010). The structural basis of peptide-protein binding strategies. *Structure*, *18*(2), 188–199. 20159464 10.1016/j.str.2009.11.012

[35] Clackson, T., & Wells, J. A. (1995). A hot spot of binding energy in a hormone-receptor interface. *Science*, *267*(5196), 383–386. 7529940 10.1126/science.7529940

[36] Wang, L., Wang, N., Zhang, W., Cheng, X., Yan, Z., Shao, G., Wang, X., Wang, R., & Fu, C. (2022). Therapeutic peptides: current applications and future directions. *Signal Transduction and Targeted Therapy*, *7*(1), 48. 35165272 10.1038/s41392-022-00904-4 PMC8844085

[37] Zorzi, A., Deyle, K., & Heinis, C. (2017). Cyclic peptide therapeutics: past, present and future. *Current Opinion in Chemical Biology*, *38*, 24–29. 28249193 10.1016/j.cbpa.2017.02.006

[38] Liu, M., Li, X., Xie, Z., Xie, C., Zhan, C., Hu, X., Shen, Q., Wei, X., Su, B., Wang, J., & Lu, W. (2016). D-peptides as recognition molecules and therapeutic agents. *Chemical Record (New York, N.Y.)*, *16*(4), 1772–1786. 27255896 10.1002/tcr.201600005

[39] Culf, A. S. (2019). Peptoids as tools and sensors. *Biopolymers*, *110*(6), e23285. 31070792 10.1002/bip.23285

[40] Esquea, E. M., Ciraku, L., Young, R. G., Merzy, J., Talarico, A. N., Ahmed, N. N., Karuppiah, M., Ramesh, A., Chatoff, A., Crispim, C. V., Rashad, A. A., Cocklin, S., Snyder, N. W., Beld, J., Simone, N. L., Reginato, M. J., & Dick, A. (2024). Selective and brain-penetrant ACSS2 inhibitors target breast cancer brain metastatic cells. *Frontiers in Pharmacology*, *15*, 1394685. 38818373 10.3389/fphar.2024.1394685 PMC11137182

[41] Oduro-Kwateng, E., Ali, M., Kehinde, I. O., Zhang, Z., & Soliman, M. E. S. (2024). De novo rational design of peptide-based protein-protein inhibitors (Pep-PPIs) approach by mapping the interaction motifs of the PP interface and physicochemical filtration: A case on p25-Cdk5-mediated neurodegenerative diseases. *Journal of Cellular Biochemistry*, *125*(9), e30633. 39148280 10.1002/jcb.30633

[42] Gulick, A. M., Starai, V. J., Horswill, A. R., Homick, K. M., & Escalante-Semerena, J. C. (2003). The 1.75 Å Crystal structure of Acetyl-CoA synthetase bound to adenosine-5′-propylphosphate and Coenzyme A†. *Biochemistry*, *42*(10), 2866–2873. 12627952 10.1021/bi0271603

[43] Ali, N., Shamoon, A., Yadav, N., & Sharma, T. (2020). Peptide combination generator: A tool for generating peptide combinations. *ACS Omega*, *5*(11), 5781–5783. 32226857 10.1021/acsomega.9b03848 PMC7097909

[44] Sharma, N., Naorem, L. D., Jain, S., & Raghava, G. P. S. (2022). ToxinPred2: An improved method for predicting toxicity of proteins. *Briefings in Bioinformatics*, *23*(5), bbac174. 35595541 10.1093/bib/bbac174

[45] Gupta, S., Kapoor, P., Chaudhary, K., Gautam, A., Kumar, R., & Raghava, G. P. S. (2015). Peptide toxicity prediction. *Methods in Molecular Biology*, *1268*, 143–157. 25555724 10.1007/978-1-4939-2285-7_7

[46] Gupta, S., Kapoor, P., Chaudhary, K., Gautam, A., Kumar, R., & Raghava, G. P. S. (2013). In Silico approach for predicting toxicity of peptides and proteins. *PLoS ONE*, *8*(9), e73957. 24058508 10.1371/journal.pone.0073957 PMC3772798

[47] Pallarés, I., & Ventura, S. (2019). Advances in the prediction of protein aggregation propensity. *Current Medicinal Chemistry*, *26*(21), 3911–3920. 28685682 10.2174/0929867324666170705121754

[48] Conchillo-Solé, O., de Groot, N. S., Avilés, F. X., Vendrell, J., Daura, X., & Ventura, S. (2007). AGGRESCAN: A server for the prediction and evaluation of “hot spots” of aggregation in polypeptides. *BMC Bioinformatics*, *8*(1), 1–17. 17324296 10.1186/1471-2105-8-65 PMC1828741

[49] Rey, J., Murail, S., De Vries, S., Derreumaux, P., & Tuffery, P. (2023). PEP-FOLD4: a pH-dependent force field for peptide structure prediction in aqueous solution. *Nucleic Acids Research*, *51*(W1), W432–W437. 37166962 10.1093/nar/gkad376 PMC10320157

[50] Dominguez, C., Boelens, R., & Bonvin, A. M. J. J. (2003). HADDOCK: A protein-protein docking approach based on biochemical or biophysical information. *Journal of the American Chemical Society*, *125*(7), 1731–1737. 12580598 10.1021/ja026939x

[51] Pettersen, E. F., Goddard, T. D., Huang, C. C., Couch, G. S., Greenblatt, D. M., Meng, E. C., & Ferrin, T. E. (2004). UCSF Chimera–a visualization system for exploratory research and analysis. *Journal of Computational Chemistry*, *25*(13), 1605–1612. 15264254 10.1002/jcc.20084

[52] Honorato, R. V., Trellet, M. E., Jiménez-García, B., Schaarschmidt, J. J., Giulini, M., Reys, V., Koukos, P. I., Rodrigues, J., Karaca, E., van Zundert, G., Roel-Touris, J., van Noort, C. W., Jandová, Z., Melquiond, A., & Bonvin, A. (2024). The HADDOCK2.4 web server for integrative modeling of biomolecular complexes. *Nature Protocols*, *19*(11), 3219–3241. 38886530 10.1038/s41596-024-01011-0

[53] Salomon-Ferrer, R., Götz, A. W., Poole, D., Le Grand, S., & Walker, R. C. (2013). Routine microsecond molecular dynamics simulations with AMBER on GPUs. 2. Explicit solvent particle mesh ewald. *Journal of Chemical Theory and Computation*, *9*(9), 3878–3888. 26592383 10.1021/ct400314y

[54] Ali, M., Zhang, Z., Ibrahim, M. A. A., & Soliman, M. E. S. (2024). Heat shock protein (Hsp27)-ceramide synthase (Cers1) protein-protein interactions provide a new avenue for unexplored anti-cancer mechanism and therapy. *Journal of Receptor and Signal Transduction Research*, *44*(2), 41–53. 39189140 10.1080/10799893.2024.2392711

[55] Ali, M., Rabbad, A. H., & Soliman, M. E. (2024). Monastrol disrupts KIFC1-ATP dynamics: Towards newer anticancer mechanism. *Chemical Physics Impact*, *8*, 100480.

[56] Case, D. A., Cheatham, T. E., Darden, T., Gohlke, H., Luo, R., Merz, Jr, K. M., Onufriev, A., Simmerling, C., Wang, B., & Woods, R. J. (2005). The Amber biomolecular simulation programs. *Journal of Computational Chemistry*, *26*(16), 1668–1688. 16200636 10.1002/jcc.20290 PMC1989667

[57] Maestro, Schrödinger, LLC, New York, NY, 2025.

[58] Oduro-Kwateng, E., & Soliman, M. E. (2024). DON/DRP-104 as potent serine protease inhibitors implicated in SARS-CoV-2 infection: Comparative binding modes with human TMPRSS2 and novel therapeutic approach. *Journal of Cellular Biochemistry*, *125*(10), e30528. 38284235 10.1002/jcb.30528

[59] Nagano, Y., Arafiles, J., Kuwata, K., Kawaguchi, Y., Imanishi, M., Hirose, H., & Futaki, S. (2022). Grafting hydrophobic amino acids critical for inhibition of protein-protein interactions on a cell-penetrating peptide scaffold. *Molecular Pharmaceutics*, *19*(2), 558–567. 34958576 10.1021/acs.molpharmaceut.1c00671

[60] Arabi-Jeshvaghani, F., Javadi-Zarnaghi, F. & Ganjalikhany, M. R. (2023). Analysis of critical protein-protein interactions of SARS-CoV-2 capping and proofreading molecular machineries towards designing dual target inhibitory peptides. *Scientific Reports*, *13*(1), 1–17. 36611052 10.1038/s41598-022-26778-8 PMC9825083

[61] Chen, Y., Guarnieri, M. T., Vasil, A. I., Vasil, M. L., Mant, C. T., & Hodges, R. S. (2007). Role of peptide hydrophobicity in the mechanism of action of α-helical antimicrobial peptides. *Antimicrobial Agents and Chemotherapy*, *51*(4), 1398–1406. 17158938 10.1128/AAC.00925-06 PMC1855469

[62] Sharma, A., Singla, D., Rashid, M., & Raghava, G. P. S. (2014). Designing of peptides with desired half-life in intestine-like environment. *BMC Bioinformatics*, *15*(1), 1–8. 25141912 10.1186/1471-2105-15-282 PMC4150950

[63] Macyszyn, J., Chyży, P., Burmistrz, M., Lobka, M., Miszkiewicz, J., Wojciechowska, M., & Trylska, J. (2023). Structural dynamics influences the antibacterial activity of a cell-penetrating peptide (KFF)3K. *Scientific Reports*, *13*(1), 1–13. 37684254 10.1038/s41598-023-38745-y PMC10491836

[64] Maupetit, J., Tuffery, P., & Derreumaux, P. (2007). A coarse-grained protein force field for folding and structure prediction. *Proteins*, *69*(2), 394–408. 17600832 10.1002/prot.21505

[65] Wang, X., Ni, D., Liu, Y., & Lu, S. (2021). Rational design of peptide-based inhibitors disrupting protein-protein interactions. *Frontiers in Chemistry*, *9*, 682675. 34017824 10.3389/fchem.2021.682675 PMC8128998

[66] Adewumi, A. T., Elrashedy, A., Soremekun, O. S., Ajadi, M. B., & Soliman, M. E. S. (2022). Weak spots inhibition in the Mycobacterium tuberculosis antigen 85C target for antitubercular drug design through selective irreversible covalent inhibitor-SER124. *Journal of Biomolecular Structure & Dynamics*, *40*(7), 2934–2954. 33155529 10.1080/07391102.2020.1844061

[67] Chen, J., Zhang, S., Wang, W., Pang, L., Zhang, Q., & Liu, X. (2021). Mutation-induced impacts on the switch transformations of the GDP- and GTP-bound K-Ras: Insights from multiple replica gaussian accelerated molecular dynamics and free energy analysis. *Journal of Chemical Information and Modeling*, *61*(4), 1954–1969. 33739090 10.1021/acs.jcim.0c01470

[68] Kazmirchuk, T. D. D., Bradbury-Jost, C., Withey, T. A., Gessese, T., Azad, T., Samanfar, B., Dehne, F., & Golshani, A. (2023). Peptides of a feather: How computation is taking peptide therapeutics under its wing. *Genes*, *14*(6), 1194. 37372372 10.3390/genes14061194 PMC10298604

[69] Miller, B. R., McGee, T. D., Swails, J. M., Homeyer, N., Gohlke, H., & Roitberg, A. E. (2012). MMPBSA.py: An efficient program for end-state free energy calculations. *Journal of Chemical Theory and Computation*, *8*(9), 3314–3321. 26605738 10.1021/ct300418h

[70] Kabsch, W., & Sander, C. (1983). Dictionary of protein secondary structure: Pattern recognition of hydrogen-bonded and geometrical features. *Biopolymers*, *22*(12), 2577–2637. 6667333 10.1002/bip.360221211

[71] Li, Z., Liu, H., He, J., Wang, Z., Yin, Z., You, G., Wang, Z., Davis, R. E., Lin, P., Bergsagel, P. L., Manasanch, E. E., Wong, S., Esnaola, N. F., Chang, J. C., Orlowski, R. Z., Yi, Q., & Yang, J. (2021). Acetyl-CoA synthetase 2: A critical linkage in obesity-induced tumorigenesis in myeloma. *Cell metabolism*, *33*(1), 78–93.e7. 33406405 10.1016/j.cmet.2020.12.011 PMC7799390

[72] Wu, B., Wijma, H. J., Song, L., Rozeboom, H. J., Poloni, C., Tian, Y., Arif, M. I., Nuijens, T., Quaedflieg, P. J. L. M., Szymanski, W., Feringa, B. L., & Janssen, D. B. (2016). Versatile peptide C-terminal functionalization via a computationally engineered peptide amidase. *ACS Catalysis*, *6*(8), 5405–5414.

[73] Levit, S. L., & Tang, C. (2021). Polymeric nanoparticle delivery of combination therapy with synergistic effects in ovarian cancer. *Nanomaterials*, *11*(4), 1048. 33923947 10.3390/nano11041048 PMC8072532

[74] Zhang, J. Z., Nguyen, W. H., Greenwood, N., Rose, J. C., Ong, S. E., Maly, D. J., & Baker, D. (2024). Computationally designed sensors detect endogenous Ras activity and signaling effectors at subcellular resolution. *Nature Biotechnology*, *42*(12), 1888–1898. 38273065 10.1038/s41587-023-02107-w PMC11631767

[75] Gupta, N., Ansari, A., Dhoke, G. V., Chilamari, M., Sivaccumar, J., Kumari, S., Chatterjee, S., Goyal, R., Dutta, P. K., Samarla, M., Mukherjee, M., Sarkar, A., Mandal, S. K., Rai, V., Biswas, G., Sengupta, A., Roy, S., Roy, M., & Sengupta, S. (2019). Computationally designed antibody–drug conjugates self-assembled via affinity ligands. *Nature Biomedical Engineering*, *3*(11), 917–929. 31686001 10.1038/s41551-019-0470-8

