# Supplementary material for: A Computational Approach for Designing a Peptide-Based Acetyl-CoA Synthetase 2 Inhibitor: A New Horizon for Anticancer Development
Source: Cell Biochem Biophys. 2025 Apr 27;83(3):3465–85. doi: 10.1007/s12013-025-01729-y (PMC12414092; doi:10.1007/s12013-025-01729-y)
Supplement: Supplementary file 1 — Supplementary Tables [file 12013_2025_1729_MOESM1_ESM.docx]

**Table S1:** Physiochemical properties of PCS after screening for toxicity

| Peptide Sequence | SVM Score | Prediction | Hydrophobicity | Amphipathicity | Mol wt |
| --- | --- | --- | --- | --- | --- |
| GDGDCWFC | -0.29 | Non-Toxin | -0.01 | 0 | 902.06 |
| GDGDCWYQ | -0.29 | Non-Toxin | -0.17 | 0.16 | 943.06 |
| GDGECWFN | -0.29 | Non-Toxin | -0.08 | 0.16 | 927.06 |
| GEGDCYWQ | -0.29 | Non-Toxin | -0.16 | 0.32 | 957.09 |
| GEPDQWWC | -0.29 | Non-Toxin | -0.14 | 0.32 | 1020.19 |
| PEPDCYYN | -0.29 | Non-Toxin | -0.26 | 0.16 | 1000.14 |
| GDGDCYYQ | -0.28 | Non-Toxin | -0.22 | 0.16 | 920.02 |
| GDPDCWWS | -0.28 | Non-Toxin | -0.1 | 0 | 965.1 |
| GDPDCWWT | -0.28 | Non-Toxin | -0.09 | 0 | 979.13 |
| GEGDCWYT | -0.28 | Non-Toxin | -0.1 | 0.16 | 930.06 |
| GEGDCWYN | -0.28 | Non-Toxin | -0.15 | 0.16 | 943.06 |
| GEGDCYFT | -0.28 | Non-Toxin | -0.07 | 0.16 | 891.02 |
| GEGDCYFN | -0.28 | Non-Toxin | -0.12 | 0.16 | 904.02 |
| GEGECYWC | -0.28 | Non-Toxin | -0.06 | 0.32 | 946.12 |
| PDGDQFFC | -0.28 | Non-Toxin | -0.1 | 0.16 | 928.08 |
| PDPDCWYC | -0.28 | Non-Toxin | -0.14 | 0 | 998.18 |
| PDPDQWWC | -0.28 | Non-Toxin | -0.19 | 0.16 | 1046.22 |
| PDPECWWQ | -0.28 | Non-Toxin | -0.17 | 0.32 | 1060.25 |
| PEGDCWYN | -0.28 | Non-Toxin | -0.18 | 0.16 | 983.12 |
| PEPECWWC | -0.28 | Non-Toxin | -0.07 | 0.32 | 1049.28 |
| GDGDCFFC | -0.27 | Non-Toxin | 0.02 | 0 | 863.02 |
| GDGECWWT | -0.27 | Non-Toxin | -0.05 | 0.16 | 953.1 |
| GDGECYWT | -0.27 | Non-Toxin | -0.1 | 0.16 | 930.06 |
| GDPDCFFN | -0.27 | Non-Toxin | -0.09 | 0 | 914.05 |
| GDPDCWFT | -0.27 | Non-Toxin | -0.06 | 0 | 940.09 |
| GDPDCYWN | -0.27 | Non-Toxin | -0.19 | 0 | 969.09 |
| GDPECFFN | -0.27 | Non-Toxin | -0.08 | 0.16 | 928.08 |
| GDPECWFT | -0.27 | Non-Toxin | -0.05 | 0.16 | 954.12 |
| GDPECWFN | -0.27 | Non-Toxin | -0.11 | 0.16 | 967.12 |
| GDPECWWS | -0.27 | Non-Toxin | -0.09 | 0.16 | 979.13 |
| GDPECYFS | -0.27 | Non-Toxin | -0.1 | 0.16 | 917.05 |
| PDGDCWYS | -0.27 | Non-Toxin | -0.15 | 0 | 942.06 |
| PDGDCWYN | -0.27 | Non-Toxin | -0.2 | 0 | 969.09 |
| PDPDCWYQ | -0.27 | Non-Toxin | -0.23 | 0.16 | 1023.18 |
| PDPECYWT | -0.27 | Non-Toxin | -0.15 | 0.16 | 1010.18 |
| PEGDCWYT | -0.27 | Non-Toxin | -0.12 | 0.16 | 970.12 |
| PEGDQWWC | -0.27 | Non-Toxin | -0.14 | 0.32 | 1020.19 |
| PEPDCFWC | -0.27 | Non-Toxin | -0.05 | 0.16 | 996.21 |
| PEPECYWC | -0.27 | Non-Toxin | -0.11 | 0.32 | 1026.24 |
| GDGECYFN | -0.26 | Non-Toxin | -0.12 | 0.16 | 904.02 |
| GDPDCWFN | -0.26 | Non-Toxin | -0.12 | 0 | 953.09 |
| GDPDQWFC | -0.26 | Non-Toxin | -0.13 | 0.16 | 967.12 |
| GDPDQWYQ | -0.26 | Non-Toxin | -0.29 | 0.31 | 1008.12 |
| GDPECWWT | -0.26 | Non-Toxin | -0.08 | 0.16 | 993.16 |
| GEGDCYYS | -0.26 | Non-Toxin | -0.15 | 0.16 | 892.99 |
| GEGDQWWC | -0.26 | Non-Toxin | -0.12 | 0.32 | 980.13 |
| GEPDCWYC | -0.26 | Non-Toxin | -0.1 | 0.16 | 972.15 |
| PDGDCYWS | -0.26 | Non-Toxin | -0.15 | 0 | 942.06 |
| PDGECYYS | -0.26 | Non-Toxin | -0.18 | 0.16 | 933.05 |
| PDPECYWS | -0.26 | Non-Toxin | -0.16 | 0.16 | 996.15 |
| PEGDCYWS | -0.26 | Non-Toxin | -0.14 | 0.16 | 956.09 |
| PEGDCYWT | -0.26 | Non-Toxin | -0.12 | 0.16 | 970.12 |
| GDGECYWQ | -0.25 | Non-Toxin | -0.16 | 0.32 | 957.09 |
| GDPDTYFC | -0.25 | Non-Toxin | -0.11 | 0 | 917.05 |
| GDPDCFFT | -0.25 | Non-Toxin | -0.03 | 0 | 901.05 |
| GDPDCWWQ | -0.25 | Non-Toxin | -0.16 | 0.16 | 1006.16 |
| GDPDCYFQ | -0.25 | Non-Toxin | -0.17 | 0.16 | 944.08 |
| GDPECFFT | -0.25 | Non-Toxin | -0.02 | 0.16 | 915.08 |
| PDGDCWYT | -0.25 | Non-Toxin | -0.14 | 0 | 956.09 |
| PDGDCYFN | -0.25 | Non-Toxin | -0.17 | 0 | 930.05 |
| PDGDCYWT | -0.25 | Non-Toxin | -0.14 | 0 | 956.09 |
| PDGDCYYT | -0.25 | Non-Toxin | -0.18 | 0 | 933.05 |
| PDGECWYS | -0.25 | Non-Toxin | -0.14 | 0.16 | 956.09 |
| PDGECWYT | -0.25 | Non-Toxin | -0.12 | 0.16 | 970.12 |
| PDGECWYN | -0.25 | Non-Toxin | -0.18 | 0.16 | 983.12 |
| PDGECYYN | -0.25 | Non-Toxin | -0.23 | 0.16 | 960.08 |
| PDPDCYYQ | -0.25 | Non-Toxin | -0.27 | 0.16 | 1000.14 |
| PDPECYFN | -0.25 | Non-Toxin | -0.18 | 0.16 | 984.14 |
| PEGDCYWQ | -0.25 | Non-Toxin | -0.19 | 0.32 | 997.15 |
| PEGECFFC | -0.25 | Non-Toxin | 0.02 | 0.32 | 931.14 |
| PEPDQWWC | -0.25 | Non-Toxin | -0.17 | 0.32 | 1060.25 |
| GDGDCWYC | -0.24 | Non-Toxin | -0.08 | 0 | 918.06 |
| GDGECWWQ | -0.24 | Non-Toxin | -0.12 | 0.32 | 980.13 |
| GDPDCYFS | -0.24 | Non-Toxin | -0.12 | 0 | 903.02 |
| GDPECYFQ | -0.24 | Non-Toxin | -0.16 | 0.32 | 958.11 |
| GDPECYWS | -0.24 | Non-Toxin | -0.14 | 0.16 | 956.09 |
| PDPECYWQ | -0.24 | Non-Toxin | -0.22 | 0.32 | 1037.21 |
| GDGECYFT | -0.23 | Non-Toxin | -0.07 | 0.16 | 891.02 |
| GDPDNYWC | -0.23 | Non-Toxin | -0.19 | 0 | 969.09 |
| GDPECYWT | -0.23 | Non-Toxin | -0.12 | 0.16 | 970.12 |
| GEGDCYYN | -0.23 | Non-Toxin | -0.2 | 0.16 | 920.02 |
| GEPDCYYC | -0.23 | Non-Toxin | -0.14 | 0.16 | 949.11 |
| PDGDTWWC | -0.23 | Non-Toxin | -0.09 | 0 | 979.13 |
| PDGDTYWC | -0.23 | Non-Toxin | -0.14 | 0 | 956.09 |
| PDGDQFWC | -0.23 | Non-Toxin | -0.13 | 0.16 | 967.12 |
| PDPDCYYC | -0.23 | Non-Toxin | -0.18 | 0 | 975.14 |
| PEGDCYFN | -0.23 | Non-Toxin | -0.15 | 0.16 | 944.08 |
| PEGDCYYS | -0.23 | Non-Toxin | -0.18 | 0.16 | 933.05 |
| PEGDCYYT | -0.23 | Non-Toxin | -0.17 | 0.16 | 947.08 |
| PEGECWFC | -0.23 | Non-Toxin | -0.01 | 0.32 | 970.18 |
| GDGECYYT | -0.22 | Non-Toxin | -0.14 | 0.16 | 907.02 |
| GDPDQYWC | -0.22 | Non-Toxin | -0.2 | 0.16 | 983.12 |
| GDPECWWQ | -0.22 | Non-Toxin | -0.14 | 0.32 | 1020.19 |
| GEGECWWC | -0.22 | Non-Toxin | -0.01 | 0.32 | 969.16 |
| PDGDCYWQ | -0.22 | Non-Toxin | -0.2 | 0.16 | 983.12 |
| PDPDCWFC | -0.22 | Non-Toxin | -0.06 | 0 | 982.18 |
| PEGDCYFT | -0.22 | Non-Toxin | -0.1 | 0.16 | 931.08 |
| GDGDCYYC | -0.21 | Non-Toxin | -0.12 | 0 | 895.02 |
| GEGDCFWC | -0.21 | Non-Toxin | 0.01 | 0.16 | 916.09 |
| GEPDCFFC | -0.21 | Non-Toxin | 0.01 | 0.16 | 917.11 |
| PDGDCYFT | -0.21 | Non-Toxin | -0.11 | 0 | 917.05 |
| PDPECYFT | -0.21 | Non-Toxin | -0.12 | 0.16 | 971.14 |
| GDGDCYFC | -0.2 | Non-Toxin | -0.05 | 0 | 879.02 |
| GDGECYYS | -0.2 | Non-Toxin | -0.15 | 0.16 | 892.99 |
| GDPDTFWC | -0.2 | Non-Toxin | -0.06 | 0 | 940.09 |
| GDPDNWWC | -0.2 | Non-Toxin | -0.15 | 0 | 992.13 |
| GDPECYWQ | -0.2 | Non-Toxin | -0.19 | 0.32 | 997.15 |
| PDGDCYYS | -0.2 | Non-Toxin | -0.19 | 0 | 919.02 |
| PDGDCYYN | -0.2 | Non-Toxin | -0.24 | 0 | 946.05 |
| PDPDCFFC | -0.2 | Non-Toxin | -0.04 | 0 | 943.14 |
| PEGECYFC | -0.2 | Non-Toxin | -0.05 | 0.32 | 947.14 |
| GDPDTFFC | -0.19 | Non-Toxin | -0.03 | 0 | 901.05 |
| GDPECYFN | -0.19 | Non-Toxin | -0.15 | 0.16 | 944.08 |
| GEPDCWFC | -0.19 | Non-Toxin | -0.02 | 0.16 | 956.15 |
| PDGECFWC | -0.19 | Non-Toxin | -0.02 | 0.16 | 956.15 |
| PEGDCFWC | -0.19 | Non-Toxin | -0.02 | 0.16 | 956.15 |
| PEPDCWYC | -0.19 | Non-Toxin | -0.13 | 0.16 | 1012.21 |
| GDGECWYS | -0.18 | Non-Toxin | -0.11 | 0.16 | 916.03 |
| GDPDCYWT | -0.18 | Non-Toxin | -0.14 | 0 | 956.09 |
| GDPDQWYC | -0.18 | Non-Toxin | -0.2 | 0.16 | 983.12 |
| GEPDCWYQ | -0.18 | Non-Toxin | -0.19 | 0.32 | 997.15 |
| GEPDCYYQ | -0.18 | Non-Toxin | -0.23 | 0.32 | 974.11 |
| GEPECWFC | -0.18 | Non-Toxin | -0.01 | 0.32 | 970.18 |
| GEPECWYC | -0.18 | Non-Toxin | -0.08 | 0.32 | 986.18 |
| GEPECYFC | -0.18 | Non-Toxin | -0.05 | 0.32 | 947.14 |
| GEPECYYC | -0.18 | Non-Toxin | -0.13 | 0.32 | 963.14 |
| PDPECYYT | -0.18 | Non-Toxin | -0.2 | 0.16 | 987.14 |
| PEGDCYYN | -0.18 | Non-Toxin | -0.23 | 0.16 | 960.08 |
| GDGECYYN | -0.17 | Non-Toxin | -0.2 | 0.16 | 920.02 |
| GDPDCYWS | -0.17 | Non-Toxin | -0.15 | 0 | 942.06 |
| GEPDCYFC | -0.17 | Non-Toxin | -0.07 | 0.16 | 933.11 |
| GEPECFFC | -0.17 | Non-Toxin | 0.02 | 0.32 | 931.14 |
| PDPDCYFC | -0.17 | Non-Toxin | -0.11 | 0 | 959.14 |
| GDGECWYN | -0.16 | Non-Toxin | -0.15 | 0.16 | 943.06 |
| GDPDCYFN | -0.16 | Non-Toxin | -0.16 | 0 | 930.05 |
| PDGDCFWC | -0.16 | Non-Toxin | -0.04 | 0 | 942.12 |
| PDPECWYS | -0.16 | Non-Toxin | -0.16 | 0.16 | 996.15 |
| PDPECWYT | -0.16 | Non-Toxin | -0.15 | 0.16 | 1010.18 |
| PEGECWYC | -0.16 | Non-Toxin | -0.08 | 0.32 | 986.18 |
| PEPDCWYQ | -0.16 | Non-Toxin | -0.22 | 0.32 | 1037.21 |
| GDPDTYWC | -0.15 | Non-Toxin | -0.14 | 0 | 956.09 |
| GDPDCWYT | -0.15 | Non-Toxin | -0.14 | 0 | 956.09 |
| GDPDCWYN | -0.15 | Non-Toxin | -0.19 | 0 | 969.09 |
| GDPDCYWQ | -0.15 | Non-Toxin | -0.2 | 0.16 | 983.12 |
| GDPDCYYT | -0.15 | Non-Toxin | -0.18 | 0 | 933.05 |
| GDPECYFT | -0.15 | Non-Toxin | -0.1 | 0.16 | 931.08 |
| PDGDQWWC | -0.15 | Non-Toxin | -0.16 | 0.16 | 1006.16 |
| PDPECWYN | -0.15 | Non-Toxin | -0.21 | 0.16 | 1023.18 |
| GDGECWYT | -0.14 | Non-Toxin | -0.1 | 0.16 | 930.06 |
| GEPECYYQ | -0.14 | Non-Toxin | -0.22 | 0.47 | 988.14 |
| PEGECYYC | -0.14 | Non-Toxin | -0.13 | 0.32 | 963.14 |
| GDGDCWWC | -0.13 | Non-Toxin | -0.04 | 0 | 941.1 |
| GDPDTWWC | -0.13 | Non-Toxin | -0.09 | 0 | 979.13 |
| GDPDCYFT | -0.13 | Non-Toxin | -0.11 | 0 | 917.05 |
| GDPECWYS | -0.13 | Non-Toxin | -0.14 | 0.16 | 956.09 |
| GDPDCWYS | -0.12 | Non-Toxin | -0.15 | 0 | 942.06 |
| GDPDQFFC | -0.12 | Non-Toxin | -0.1 | 0.16 | 928.08 |
| PDPECYYS | -0.12 | Non-Toxin | -0.21 | 0.16 | 973.11 |
| PEGECYYQ | -0.12 | Non-Toxin | -0.22 | 0.47 | 988.14 |
| GDPECWYN | -0.11 | Non-Toxin | -0.18 | 0.16 | 983.12 |
| GDPECYYT | -0.11 | Non-Toxin | -0.17 | 0.16 | 947.08 |
| GEGDCFFC | -0.11 | Non-Toxin | 0.03 | 0.16 | 877.05 |
| GEPECWYQ | -0.11 | Non-Toxin | -0.18 | 0.47 | 1011.18 |
| PDPECYYN | -0.11 | Non-Toxin | -0.26 | 0.16 | 1000.14 |
| PEGECWYQ | -0.11 | Non-Toxin | -0.18 | 0.47 | 1011.18 |
| PEPDCYYC | -0.11 | Non-Toxin | -0.17 | 0.16 | 989.17 |
| GDPDQFWC | -0.1 | Non-Toxin | -0.13 | 0.16 | 967.12 |
| PDPDCWWC | -0.1 | Non-Toxin | -0.09 | 0 | 1021.22 |
| PEPDCYYQ | -0.1 | Non-Toxin | -0.26 | 0.32 | 1014.17 |
| GDPECYYN | -0.07 | Non-Toxin | -0.23 | 0.16 | 960.08 |
| PDGDCWYQ | -0.05 | Non-Toxin | -0.2 | 0.16 | 983.12 |
| PDGDCYYQ | -0.02 | Non-Toxin | -0.24 | 0.16 | 960.08 |

**Table S2.** Physiochemical properties of PCS after screening for amphipathicity

| Peptide Sequence | SVM Score | Prediction | Hydrophobicity | Amphipathicity | Mol wt |
| --- | --- | --- | --- | --- | --- |
| GDGDCWYQ | -0.29 | Non-Toxin | -0.17 | 0.16 | 943.06 |
| GDGECWFN | -0.29 | Non-Toxin | -0.08 | 0.16 | 927.06 |
| PEPDCYYN | -0.29 | Non-Toxin | -0.26 | 0.16 | 1000.14 |
| GDGDCYYQ | -0.28 | Non-Toxin | -0.22 | 0.16 | 920.02 |
| GEGDCWYT | -0.28 | Non-Toxin | -0.1 | 0.16 | 930.06 |
| GEGDCWYN | -0.28 | Non-Toxin | -0.15 | 0.16 | 943.06 |
| GEGDCYFT | -0.28 | Non-Toxin | -0.07 | 0.16 | 891.02 |
| GEGDCYFN | -0.28 | Non-Toxin | -0.12 | 0.16 | 904.02 |
| PDGDQFFC | -0.28 | Non-Toxin | -0.1 | 0.16 | 928.08 |
| PDPDQWWC | -0.28 | Non-Toxin | -0.19 | 0.16 | 1046.22 |
| PEGDCWYN | -0.28 | Non-Toxin | -0.18 | 0.16 | 983.12 |
| GDGECWWT | -0.27 | Non-Toxin | -0.05 | 0.16 | 953.1 |
| GDGECYWT | -0.27 | Non-Toxin | -0.1 | 0.16 | 930.06 |
| GDPECFFN | -0.27 | Non-Toxin | -0.08 | 0.16 | 928.08 |
| GDPECWFT | -0.27 | Non-Toxin | -0.05 | 0.16 | 954.12 |
| GDPECWFN | -0.27 | Non-Toxin | -0.11 | 0.16 | 967.12 |
| GDPECWWS | -0.27 | Non-Toxin | -0.09 | 0.16 | 979.13 |
| GDPECYFS | -0.27 | Non-Toxin | -0.1 | 0.16 | 917.05 |
| PDPDCWYQ | -0.27 | Non-Toxin | -0.23 | 0.16 | 1023.18 |
| PDPECYWT | -0.27 | Non-Toxin | -0.15 | 0.16 | 1010.18 |
| PEGDCWYT | -0.27 | Non-Toxin | -0.12 | 0.16 | 970.12 |
| PEPDCFWC | -0.27 | Non-Toxin | -0.05 | 0.16 | 996.21 |
| GDGECYFN | -0.26 | Non-Toxin | -0.12 | 0.16 | 904.02 |
| GDPDQWFC | -0.26 | Non-Toxin | -0.13 | 0.16 | 967.12 |
| GDPECWWT | -0.26 | Non-Toxin | -0.08 | 0.16 | 993.16 |
| GEGDCYYS | -0.26 | Non-Toxin | -0.15 | 0.16 | 892.99 |
| GEPDCWYC | -0.26 | Non-Toxin | -0.1 | 0.16 | 972.15 |
| PDGECYYS | -0.26 | Non-Toxin | -0.18 | 0.16 | 933.05 |
| PDPECYWS | -0.26 | Non-Toxin | -0.16 | 0.16 | 996.15 |
| PEGDCYWS | -0.26 | Non-Toxin | -0.14 | 0.16 | 956.09 |
| PEGDCYWT | -0.26 | Non-Toxin | -0.12 | 0.16 | 970.12 |
| GDPDCWWQ | -0.25 | Non-Toxin | -0.16 | 0.16 | 1006.16 |
| GDPDCYFQ | -0.25 | Non-Toxin | -0.17 | 0.16 | 944.08 |
| GDPECFFT | -0.25 | Non-Toxin | -0.02 | 0.16 | 915.08 |
| PDGECWYS | -0.25 | Non-Toxin | -0.14 | 0.16 | 956.09 |
| PDGECWYT | -0.25 | Non-Toxin | -0.12 | 0.16 | 970.12 |
| PDGECWYN | -0.25 | Non-Toxin | -0.18 | 0.16 | 983.12 |
| PDGECYYN | -0.25 | Non-Toxin | -0.23 | 0.16 | 960.08 |
| PDPDCYYQ | -0.25 | Non-Toxin | -0.27 | 0.16 | 1000.14 |
| PDPECYFN | -0.25 | Non-Toxin | -0.18 | 0.16 | 984.14 |
| GDPECYWS | -0.24 | Non-Toxin | -0.14 | 0.16 | 956.09 |
| GDGECYFT | -0.23 | Non-Toxin | -0.07 | 0.16 | 891.02 |
| GDPECYWT | -0.23 | Non-Toxin | -0.12 | 0.16 | 970.12 |
| GEGDCYYN | -0.23 | Non-Toxin | -0.2 | 0.16 | 920.02 |
| GEPDCYYC | -0.23 | Non-Toxin | -0.14 | 0.16 | 949.11 |
| PDGDQFWC | -0.23 | Non-Toxin | -0.13 | 0.16 | 967.12 |
| PEGDCYFN | -0.23 | Non-Toxin | -0.15 | 0.16 | 944.08 |
| PEGDCYYS | -0.23 | Non-Toxin | -0.18 | 0.16 | 933.05 |
| PEGDCYYT | -0.23 | Non-Toxin | -0.17 | 0.16 | 947.08 |
| GDGECYYT | -0.22 | Non-Toxin | -0.14 | 0.16 | 907.02 |
| GDPDQYWC | -0.22 | Non-Toxin | -0.2 | 0.16 | 983.12 |
| PDGDCYWQ | -0.22 | Non-Toxin | -0.2 | 0.16 | 983.12 |
| PEGDCYFT | -0.22 | Non-Toxin | -0.1 | 0.16 | 931.08 |
| GEGDCFWC | -0.21 | Non-Toxin | 0.01 | 0.16 | 916.09 |
| GEPDCFFC | -0.21 | Non-Toxin | 0.01 | 0.16 | 917.11 |
| PDPECYFT | -0.21 | Non-Toxin | -0.12 | 0.16 | 971.14 |
| GDGECYYS | -0.2 | Non-Toxin | -0.15 | 0.16 | 892.99 |
| GDPECYFN | -0.19 | Non-Toxin | -0.15 | 0.16 | 944.08 |
| GEPDCWFC | -0.19 | Non-Toxin | -0.02 | 0.16 | 956.15 |
| PDGECFWC | -0.19 | Non-Toxin | -0.02 | 0.16 | 956.15 |
| PEGDCFWC | -0.19 | Non-Toxin | -0.02 | 0.16 | 956.15 |
| PEPDCWYC | -0.19 | Non-Toxin | -0.13 | 0.16 | 1012.21 |
| GDGECWYS | -0.18 | Non-Toxin | -0.11 | 0.16 | 916.03 |
| GDPDQWYC | -0.18 | Non-Toxin | -0.2 | 0.16 | 983.12 |
| PDPECYYT | -0.18 | Non-Toxin | -0.2 | 0.16 | 987.14 |
| PEGDCYYN | -0.18 | Non-Toxin | -0.23 | 0.16 | 960.08 |
| GDGECYYN | -0.17 | Non-Toxin | -0.2 | 0.16 | 920.02 |
| GEPDCYFC | -0.17 | Non-Toxin | -0.07 | 0.16 | 933.11 |
| GDGECWYN | -0.16 | Non-Toxin | -0.15 | 0.16 | 943.06 |
| PDPECWYS | -0.16 | Non-Toxin | -0.16 | 0.16 | 996.15 |
| PDPECWYT | -0.16 | Non-Toxin | -0.15 | 0.16 | 1010.18 |
| GDPDCYWQ | -0.15 | Non-Toxin | -0.2 | 0.16 | 983.12 |
| GDPECYFT | -0.15 | Non-Toxin | -0.1 | 0.16 | 931.08 |
| PDGDQWWC | -0.15 | Non-Toxin | -0.16 | 0.16 | 1006.16 |
| PDPECWYN | -0.15 | Non-Toxin | -0.21 | 0.16 | 1023.18 |
| GDGECWYT | -0.14 | Non-Toxin | -0.1 | 0.16 | 930.06 |
| GDPECWYS | -0.13 | Non-Toxin | -0.14 | 0.16 | 956.09 |
| GDPDQFFC | -0.12 | Non-Toxin | -0.1 | 0.16 | 928.08 |
| PDPECYYS | -0.12 | Non-Toxin | -0.21 | 0.16 | 973.11 |
| GDPECWYN | -0.11 | Non-Toxin | -0.18 | 0.16 | 983.12 |
| GDPECYYT | -0.11 | Non-Toxin | -0.17 | 0.16 | 947.08 |
| GEGDCFFC | -0.11 | Non-Toxin | 0.03 | 0.16 | 877.05 |
| PDPECYYN | -0.11 | Non-Toxin | -0.26 | 0.16 | 1000.14 |
| PEPDCYYC | -0.11 | Non-Toxin | -0.17 | 0.16 | 989.17 |
| GDPDQFWC | -0.1 | Non-Toxin | -0.13 | 0.16 | 967.12 |
| GDPECYYN | -0.07 | Non-Toxin | -0.23 | 0.16 | 960.08 |
| PDGDCWYQ | -0.05 | Non-Toxin | -0.2 | 0.16 | 983.12 |
| PDGDCYYQ | -0.02 | Non-Toxin | -0.24 | 0.16 | 960.08 |

**Table S3.** Physiochemical properties of PCS after screening for hydrophobicity

| Peptide Sequence | SVM Score | Prediction | Hydrophobicity | Amphipathicity | Mol wt |
| --- | --- | --- | --- | --- | --- |
| PDPDCYYQ | -0.25 | Non-Toxin | -0.27 | 0.16 | 1000.14 |
| PEPDCYYN | -0.29 | Non-Toxin | -0.26 | 0.16 | 1000.14 |
| PDPECYYN | -0.11 | Non-Toxin | -0.26 | 0.16 | 1000.14 |
| PDPDCWYQ | -0.27 | Non-Toxin | -0.23 | 0.16 | 1023.18 |
| PDGECYYN | -0.25 | Non-Toxin | -0.23 | 0.16 | 960.08 |
| PEGDCYYN | -0.18 | Non-Toxin | -0.23 | 0.16 | 960.08 |
| GDGDCYYQ | -0.28 | Non-Toxin | -0.22 | 0.16 | 920.02 |
| PDPECWYN | -0.15 | Non-Toxin | -0.21 | 0.16 | 1023.18 |
| PDPECYYS | -0.12 | Non-Toxin | -0.21 | 0.16 | 973.11 |
| GEGDCYYN | -0.23 | Non-Toxin | -0.2 | 0.16 | 920.02 |
| GDPDQYWC | -0.22 | Non-Toxin | -0.2 | 0.16 | 983.12 |
| PDGDCYWQ | -0.22 | Non-Toxin | -0.2 | 0.16 | 983.12 |
| GDPDQWYC | -0.18 | Non-Toxin | -0.2 | 0.16 | 983.12 |
| PDPECYYT | -0.18 | Non-Toxin | -0.2 | 0.16 | 987.14 |
| GDGECYYN | -0.17 | Non-Toxin | -0.2 | 0.16 | 920.02 |
| GDPDCYWQ | -0.15 | Non-Toxin | -0.2 | 0.16 | 983.12 |
| GDPECYYN | -0.07 | Non-Toxin | -0.23 | 0.16 | 960.08 |
| PDGDCWYQ | -0.05 | Non-Toxin | -0.2 | 0.16 | 983.12 |
| PDGDCYYQ | -0.02 | Non-Toxin | -0.24 | 0.16 | 960.08 |

**Table S4.** Physiochemical Properties of PCS after screening for Propensity for in vivo Aggregation

| Sequence No. | Peptide Sequence | SVM Score | Prediction | Hydrophobicity | Amphipathicity | Mol wt | Na4vSS | sOPEP energy |
| --- | --- | --- | --- | --- | --- | --- | --- | --- |
| 10 | GDPDQWYC | -0.18 | Non-Toxin | -0.2 | 0.16 | 983.12 | -57 | -3.63035 |
| 7 | GDPDQYWC | -0.22 | Non-Toxin | -0.2 | 0.16 | 983.12 | -56.7 | -3.73611 |
| 2 | GDGDCYYQ | -0.28 | Non-Toxin | -0.22 | 0.16 | 920.02 | -44.2 | -1.9197 |
| 8 | PDGDCYWQ | -0.22 | Non-Toxin | -0.2 | 0.16 | 983.12 | -44 | -2.9538 |
| 13 | GDPDCYWQ | -0.15 | Non-Toxin | -0.2 | 0.16 | 983.12 | -42.9 | -2.40548 |
| 3 | PDPDCWYQ | -0.27 | Non-Toxin | -0.23 | 0.16 | 1023.18 | -41.7 | -2.45375 |
| 6 | GEGDCYYN | -0.23 | Non-Toxin | -0.2 | 0.16 | 920.02 | -40.5 | -2.2124 |
| 5 | PDPDCYYQ | -0.25 | Non-Toxin | -0.27 | 0.16 | 1000.14 | -40.2 | -2.49303 |
| 9 | PEGDCYYN | -0.18 | Non-Toxin | -0.23 | 0.16 | 960.08 | -39 | -2.70096 |
| 12 | GDGECYYN | -0.17 | Non-Toxin | -0.2 | 0.16 | 920.02 | -38.4 | -1.26908 |
| 4 | PDGECYYN | -0.25 | Non-Toxin | -0.23 | 0.16 | 960.08 | -36.9 | -1.71421 |
| 1 | PEPDCYYN | -0.29 | Non-Toxin | -0.26 | 0.16 | 1000.14 | -36.5 | -7.09487 |
| 14 | PDPECWYN | -0.15 | Non-Toxin | -0.21 | 0.16 | 1023.18 | -35.9 | -2.46027 |
| 16 | PDPECYYN | -0.11 | Non-Toxin | -0.26 | 0.16 | 1000.14 | -34.4 | -2.36291 |
| 15 | PDPECYYS | -0.12 | Non-Toxin | -0.21 | 0.16 | 973.11 | -26.8 | -2.80309 |
| 11 | PDPECYYT | -0.18 | Non-Toxin | -0.2 | 0.16 | 987.14 | -25.8 | -2.36648 |

**Table S5.** Physiochemical properties of all PCS.

| Peptide  ID | Peptide Sequence | SVM Score | Prediction | Hydrophobicity | Amphipathicity | Mol wt |
| --- | --- | --- | --- | --- | --- | --- |
| 1 | GDGDSFFS | -0.88 | Non-Toxin | -0.05 | 0 | 830.9 |
| 2 | GDGDSFFT | -0.83 | Non-Toxin | -0.04 | 0 | 844.93 |
| 3 | GDGDSFFC | -0.65 | Non-Toxin | -0.01 | 0 | 846.96 |
| 4 | GDGDSFFN | -0.85 | Non-Toxin | -0.1 | 0 | 857.93 |
| 5 | GDGDSFFQ | -0.87 | Non-Toxin | -0.11 | 0.16 | 871.96 |
| 6 | GDGDSFWS | -1 | Non-Toxin | -0.08 | 0 | 869.94 |
| 7 | GDGDSFWT | -0.92 | Non-Toxin | -0.07 | 0 | 883.97 |
| 8 | GDGDSFWC | -0.55 | Non-Toxin | -0.04 | 0 | 886 |
| 9 | GDGDSFWN | -0.98 | Non-Toxin | -0.13 | 0 | 896.97 |
| 10 | GDGDSFWQ | -0.91 | Non-Toxin | -0.14 | 0.16 | 911 |
| 11 | GDGDSFYS | -1.05 | Non-Toxin | -0.13 | 0 | 846.9 |
| 12 | GDGDSFYT | -1 | Non-Toxin | -0.12 | 0 | 860.93 |
| 13 | GDGDSFYC | -0.9 | Non-Toxin | -0.09 | 0 | 862.96 |
| 14 | GDGDSFYN | -1.02 | Non-Toxin | -0.17 | 0 | 873.93 |
| 15 | GDGDSFYQ | -0.9 | Non-Toxin | -0.18 | 0.16 | 887.96 |
| 16 | GDGDSWFS | -1.07 | Non-Toxin | -0.08 | 0 | 869.94 |
| 17 | GDGDSWFT | -1.02 | Non-Toxin | -0.07 | 0 | 883.97 |
| 18 | GDGDSWFC | -0.83 | Non-Toxin | -0.04 | 0 | 886 |
| 19 | GDGDSWFN | -1.02 | Non-Toxin | -0.13 | 0 | 896.97 |
| 20 | GDGDSWFQ | -1.05 | Non-Toxin | -0.14 | 0.16 | 911 |
| 21 | GDGDSWWS | -1.03 | Non-Toxin | -0.11 | 0 | 908.98 |
| 22 | GDGDSWWT | -0.98 | Non-Toxin | -0.1 | 0 | 923.01 |
| 23 | GDGDSWWC | -0.62 | Non-Toxin | -0.07 | 0 | 925.04 |
| 24 | GDGDSWWN | -1.03 | Non-Toxin | -0.16 | 0 | 936.01 |
| 25 | GDGDSWWQ | -0.96 | Non-Toxin | -0.17 | 0.16 | 950.04 |
| 26 | GDGDSWYS | -0.91 | Non-Toxin | -0.16 | 0 | 885.94 |
| 27 | GDGDSWYT | -0.88 | Non-Toxin | -0.15 | 0 | 899.97 |
| 28 | GDGDSWYC | -0.72 | Non-Toxin | -0.12 | 0 | 902 |
| 29 | GDGDSWYN | -0.88 | Non-Toxin | -0.2 | 0 | 912.97 |
| 30 | GDGDSWYQ | -0.75 | Non-Toxin | -0.21 | 0.16 | 927 |
| 31 | GDGDSYFS | -0.94 | Non-Toxin | -0.13 | 0 | 846.9 |
| 32 | GDGDSYFT | -0.88 | Non-Toxin | -0.12 | 0 | 860.93 |
| 33 | GDGDSYFC | -0.71 | Non-Toxin | -0.09 | 0 | 862.96 |
| 34 | GDGDSYFN | -0.91 | Non-Toxin | -0.17 | 0 | 873.93 |
| 35 | GDGDSYFQ | -0.94 | Non-Toxin | -0.18 | 0.16 | 887.96 |
| 36 | GDGDSYWS | -1.01 | Non-Toxin | -0.16 | 0 | 885.94 |
| 37 | GDGDSYWT | -0.96 | Non-Toxin | -0.15 | 0 | 899.97 |
| 38 | GDGDSYWC | -0.6 | Non-Toxin | -0.12 | 0 | 902 |
| 39 | GDGDSYWN | -1.02 | Non-Toxin | -0.2 | 0 | 912.97 |
| 40 | GDGDSYWQ | -0.94 | Non-Toxin | -0.21 | 0.16 | 927 |
| 41 | GDGDSYYS | -0.89 | Non-Toxin | -0.2 | 0 | 862.9 |
| 42 | GDGDSYYT | -0.86 | Non-Toxin | -0.19 | 0 | 876.93 |
| 43 | GDGDSYYC | -0.66 | Non-Toxin | -0.16 | 0 | 878.96 |
| 44 | GDGDSYYN | -0.87 | Non-Toxin | -0.25 | 0 | 889.93 |
| 45 | GDGDSYYQ | -0.73 | Non-Toxin | -0.25 | 0.16 | 903.96 |
| 46 | GDGDTFFS | -0.82 | Non-Toxin | -0.04 | 0 | 844.93 |
| 47 | GDGDTFFT | -0.75 | Non-Toxin | -0.03 | 0 | 858.96 |
| 48 | GDGDTFFC | -0.53 | Non-Toxin | 0 | 0 | 860.99 |
| 49 | GDGDTFFN | -0.74 | Non-Toxin | -0.09 | 0 | 871.96 |
| 50 | GDGDTFFQ | -0.81 | Non-Toxin | -0.1 | 0.16 | 885.99 |
| 51 | GDGDTFWS | -0.89 | Non-Toxin | -0.07 | 0 | 883.97 |
| 52 | GDGDTFWT | -0.83 | Non-Toxin | -0.06 | 0 | 898 |
| 53 | GDGDTFWC | -0.49 | Non-Toxin | -0.03 | 0 | 900.03 |
| 54 | GDGDTFWN | -0.9 | Non-Toxin | -0.12 | 0 | 911 |
| 55 | GDGDTFWQ | -0.83 | Non-Toxin | -0.13 | 0.16 | 925.03 |
| 56 | GDGDTFYS | -0.92 | Non-Toxin | -0.12 | 0 | 860.93 |
| 57 | GDGDTFYT | -0.9 | Non-Toxin | -0.11 | 0 | 874.96 |
| 58 | GDGDTFYC | -0.74 | Non-Toxin | -0.08 | 0 | 876.99 |
| 59 | GDGDTFYN | -0.92 | Non-Toxin | -0.16 | 0 | 887.96 |
| 60 | GDGDTFYQ | -0.8 | Non-Toxin | -0.17 | 0.16 | 901.99 |
| 61 | GDGDTWFS | -0.87 | Non-Toxin | -0.07 | 0 | 883.97 |
| 62 | GDGDTWFT | -0.81 | Non-Toxin | -0.06 | 0 | 898 |
| 63 | GDGDTWFC | -0.6 | Non-Toxin | -0.03 | 0 | 900.03 |
| 64 | GDGDTWFN | -0.82 | Non-Toxin | -0.12 | 0 | 911 |
| 65 | GDGDTWFQ | -0.86 | Non-Toxin | -0.13 | 0.16 | 925.03 |
| 66 | GDGDTWWS | -0.84 | Non-Toxin | -0.1 | 0 | 923.01 |
| 67 | GDGDTWWT | -0.8 | Non-Toxin | -0.09 | 0 | 937.04 |
| 68 | GDGDTWWC | -0.43 | Non-Toxin | -0.06 | 0 | 939.07 |
| 69 | GDGDTWWN | -0.85 | Non-Toxin | -0.15 | 0 | 950.04 |
| 70 | GDGDTWWQ | -0.78 | Non-Toxin | -0.16 | 0.16 | 964.07 |
| 71 | GDGDTWYS | -0.74 | Non-Toxin | -0.15 | 0 | 899.97 |
| 72 | GDGDTWYT | -0.71 | Non-Toxin | -0.14 | 0 | 914 |
| 73 | GDGDTWYC | -0.52 | Non-Toxin | -0.11 | 0 | 916.03 |
| 74 | GDGDTWYN | -0.73 | Non-Toxin | -0.19 | 0 | 927 |
| 75 | GDGDTWYQ | -0.6 | Non-Toxin | -0.2 | 0.16 | 941.03 |
| 76 | GDGDTYFS | -0.77 | Non-Toxin | -0.12 | 0 | 860.93 |
| 77 | GDGDTYFT | -0.69 | Non-Toxin | -0.11 | 0 | 874.96 |
| 78 | GDGDTYFC | -0.51 | Non-Toxin | -0.08 | 0 | 876.99 |
| 79 | GDGDTYFN | -0.75 | Non-Toxin | -0.16 | 0 | 887.96 |
| 80 | GDGDTYFQ | -0.77 | Non-Toxin | -0.17 | 0.16 | 901.99 |
| 81 | GDGDTYWS | -0.83 | Non-Toxin | -0.15 | 0 | 899.97 |
| 82 | GDGDTYWT | -0.8 | Non-Toxin | -0.14 | 0 | 914 |
| 83 | GDGDTYWC | -0.44 | Non-Toxin | -0.11 | 0 | 916.03 |
| 84 | GDGDTYWN | -0.86 | Non-Toxin | -0.19 | 0 | 927 |
| 85 | GDGDTYWQ | -0.78 | Non-Toxin | -0.2 | 0.16 | 941.03 |
| 86 | GDGDTYYS | -0.77 | Non-Toxin | -0.19 | 0 | 876.93 |
| 87 | GDGDTYYT | -0.72 | Non-Toxin | -0.18 | 0 | 890.96 |
| 88 | GDGDTYYC | -0.52 | Non-Toxin | -0.15 | 0 | 892.99 |
| 89 | GDGDTYYN | -0.76 | Non-Toxin | -0.24 | 0 | 903.96 |
| 90 | GDGDTYYQ | -0.62 | Non-Toxin | -0.24 | 0.16 | 917.99 |
| 91 | GDGDCFFS | -0.58 | Non-Toxin | -0.01 | 0 | 846.96 |
| 92 | GDGDCFFT | -0.52 | Non-Toxin | 0 | 0 | 860.99 |
| 93 | GDGDCFFC | -0.27 | Non-Toxin | 0.02 | 0 | 863.02 |
| 94 | GDGDCFFN | -0.53 | Non-Toxin | -0.06 | 0 | 873.99 |
| 95 | GDGDCFFQ | -0.58 | Non-Toxin | -0.07 | 0.16 | 888.02 |
| 96 | GDGDCFWS | -0.75 | Non-Toxin | -0.04 | 0 | 886 |
| 97 | GDGDCFWT | -0.71 | Non-Toxin | -0.03 | 0 | 900.03 |
| 98 | GDGDCFWC | -0.35 | Non-Toxin | -0.01 | 0 | 902.06 |
| 99 | GDGDCFWN | -0.76 | Non-Toxin | -0.09 | 0 | 913.03 |
| 100 | GDGDCFWQ | -0.69 | Non-Toxin | -0.1 | 0.16 | 927.06 |
| 101 | GDGDCFYS | -0.74 | Non-Toxin | -0.09 | 0 | 862.96 |
| 102 | GDGDCFYT | -0.72 | Non-Toxin | -0.08 | 0 | 876.99 |
| 103 | GDGDCFYC | -0.62 | Non-Toxin | -0.05 | 0 | 879.02 |
| 104 | GDGDCFYN | -0.74 | Non-Toxin | -0.14 | 0 | 889.99 |
| 105 | GDGDCFYQ | -0.61 | Non-Toxin | -0.14 | 0.16 | 904.02 |
| 106 | GDGDCWFS | -0.57 | Non-Toxin | -0.04 | 0 | 886 |
| 107 | GDGDCWFT | -0.51 | Non-Toxin | -0.03 | 0 | 900.03 |
| 108 | GDGDCWFC | -0.29 | Non-Toxin | -0.01 | 0 | 902.06 |
| 109 | GDGDCWFN | -0.5 | Non-Toxin | -0.09 | 0 | 913.03 |
| 110 | GDGDCWFQ | -0.56 | Non-Toxin | -0.1 | 0.16 | 927.06 |
| 111 | GDGDCWWS | -0.53 | Non-Toxin | -0.07 | 0 | 925.04 |
| 112 | GDGDCWWT | -0.51 | Non-Toxin | -0.06 | 0 | 939.07 |
| 113 | GDGDCWWC | -0.13 | Non-Toxin | -0.04 | 0 | 941.1 |
| 114 | GDGDCWWN | -0.55 | Non-Toxin | -0.12 | 0 | 952.07 |
| 115 | GDGDCWWQ | -0.48 | Non-Toxin | -0.13 | 0.16 | 966.1 |
| 116 | GDGDCWYS | -0.45 | Non-Toxin | -0.12 | 0 | 902 |
| 117 | GDGDCWYT | -0.41 | Non-Toxin | -0.11 | 0 | 916.03 |
| 118 | GDGDCWYC | -0.24 | Non-Toxin | -0.08 | 0 | 918.06 |
| 119 | GDGDCWYN | -0.44 | Non-Toxin | -0.17 | 0 | 929.03 |
| 120 | GDGDCWYQ | -0.29 | Non-Toxin | -0.17 | 0.16 | 943.06 |
| 121 | GDGDCYFS | -0.47 | Non-Toxin | -0.09 | 0 | 862.96 |
| 122 | GDGDCYFT | -0.4 | Non-Toxin | -0.08 | 0 | 876.99 |
| 123 | GDGDCYFC | -0.2 | Non-Toxin | -0.05 | 0 | 879.02 |
| 124 | GDGDCYFN | -0.42 | Non-Toxin | -0.14 | 0 | 889.99 |
| 125 | GDGDCYFQ | -0.47 | Non-Toxin | -0.14 | 0.16 | 904.02 |
| 126 | GDGDCYWS | -0.46 | Non-Toxin | -0.12 | 0 | 902 |
| 127 | GDGDCYWT | -0.43 | Non-Toxin | -0.11 | 0 | 916.03 |
| 128 | GDGDCYWC | -0.09 | Non-Toxin | -0.08 | 0 | 918.06 |
| 129 | GDGDCYWN | -0.5 | Non-Toxin | -0.17 | 0 | 929.03 |
| 130 | GDGDCYWQ | -0.42 | Non-Toxin | -0.17 | 0.16 | 943.06 |
| 131 | GDGDCYYS | -0.41 | Non-Toxin | -0.16 | 0 | 878.96 |
| 132 | GDGDCYYT | -0.42 | Non-Toxin | -0.15 | 0 | 892.99 |
| 133 | GDGDCYYC | -0.21 | Non-Toxin | -0.12 | 0 | 895.02 |
| 134 | GDGDCYYN | -0.4 | Non-Toxin | -0.21 | 0 | 905.99 |
| 135 | GDGDCYYQ | -0.28 | Non-Toxin | -0.22 | 0.16 | 920.02 |
| 136 | GDGDNFFS | -1.05 | Non-Toxin | -0.1 | 0 | 857.93 |
| 137 | GDGDNFFT | -0.99 | Non-Toxin | -0.09 | 0 | 871.96 |
| 138 | GDGDNFFC | -0.78 | Non-Toxin | -0.06 | 0 | 873.99 |
| 139 | GDGDNFFN | -1 | Non-Toxin | -0.15 | 0 | 884.96 |
| 140 | GDGDNFFQ | -1.05 | Non-Toxin | -0.15 | 0.16 | 898.99 |
| 141 | GDGDNFWS | -1.14 | Non-Toxin | -0.13 | 0 | 896.97 |
| 142 | GDGDNFWT | -1.1 | Non-Toxin | -0.12 | 0 | 911 |
| 143 | GDGDNFWC | -0.76 | Non-Toxin | -0.09 | 0 | 913.03 |
| 144 | GDGDNFWN | -1.16 | Non-Toxin | -0.18 | 0 | 924 |
| 145 | GDGDNFWQ | -1.09 | Non-Toxin | -0.18 | 0.16 | 938.03 |
| 146 | GDGDNFYS | -1.19 | Non-Toxin | -0.17 | 0 | 873.93 |
| 147 | GDGDNFYT | -1.14 | Non-Toxin | -0.16 | 0 | 887.96 |
| 148 | GDGDNFYC | -1 | Non-Toxin | -0.14 | 0 | 889.99 |
| 149 | GDGDNFYN | -1.16 | Non-Toxin | -0.22 | 0 | 900.96 |
| 150 | GDGDNFYQ | -1.05 | Non-Toxin | -0.23 | 0.16 | 914.99 |
| 151 | GDGDNWFS | -0.98 | Non-Toxin | -0.13 | 0 | 896.97 |
| 152 | GDGDNWFT | -0.93 | Non-Toxin | -0.12 | 0 | 911 |
| 153 | GDGDNWFC | -0.73 | Non-Toxin | -0.09 | 0 | 913.03 |
| 154 | GDGDNWFN | -0.94 | Non-Toxin | -0.18 | 0 | 924 |
| 155 | GDGDNWFQ | -0.98 | Non-Toxin | -0.18 | 0.16 | 938.03 |
| 156 | GDGDNWWS | -0.95 | Non-Toxin | -0.16 | 0 | 936.01 |
| 157 | GDGDNWWT | -0.91 | Non-Toxin | -0.15 | 0 | 950.04 |
| 158 | GDGDNWWC | -0.56 | Non-Toxin | -0.12 | 0 | 952.07 |
| 159 | GDGDNWWN | -0.97 | Non-Toxin | -0.21 | 0 | 963.04 |
| 160 | GDGDNWWQ | -0.9 | Non-Toxin | -0.21 | 0.16 | 977.07 |
| 161 | GDGDNWYS | -0.87 | Non-Toxin | -0.2 | 0 | 912.97 |
| 162 | GDGDNWYT | -0.84 | Non-Toxin | -0.19 | 0 | 927 |
| 163 | GDGDNWYC | -0.65 | Non-Toxin | -0.17 | 0 | 929.03 |
| 164 | GDGDNWYN | -0.84 | Non-Toxin | -0.25 | 0 | 940 |
| 165 | GDGDNWYQ | -0.71 | Non-Toxin | -0.26 | 0.16 | 954.03 |
| 166 | GDGDNYFS | -0.9 | Non-Toxin | -0.17 | 0 | 873.93 |
| 167 | GDGDNYFT | -0.86 | Non-Toxin | -0.16 | 0 | 887.96 |
| 168 | GDGDNYFC | -0.66 | Non-Toxin | -0.14 | 0 | 889.99 |
| 169 | GDGDNYFN | -0.89 | Non-Toxin | -0.22 | 0 | 900.96 |
| 170 | GDGDNYFQ | -0.92 | Non-Toxin | -0.23 | 0.16 | 914.99 |
| 171 | GDGDNYWS | -0.98 | Non-Toxin | -0.2 | 0 | 912.97 |
| 172 | GDGDNYWT | -0.95 | Non-Toxin | -0.19 | 0 | 927 |
| 173 | GDGDNYWC | -0.59 | Non-Toxin | -0.17 | 0 | 929.03 |
| 174 | GDGDNYWN | -1.01 | Non-Toxin | -0.25 | 0 | 940 |
| 175 | GDGDNYWQ | -0.93 | Non-Toxin | -0.26 | 0.16 | 954.03 |
| 176 | GDGDNYYS | -0.93 | Non-Toxin | -0.25 | 0 | 889.93 |
| 177 | GDGDNYYT | -0.86 | Non-Toxin | -0.24 | 0 | 903.96 |
| 178 | GDGDNYYC | -0.62 | Non-Toxin | -0.21 | 0 | 905.99 |
| 179 | GDGDNYYN | -0.9 | Non-Toxin | -0.29 | 0 | 916.96 |
| 180 | GDGDNYYQ | -0.76 | Non-Toxin | -0.3 | 0.16 | 930.99 |
| 181 | GDGDQFFS | -0.77 | Non-Toxin | -0.11 | 0.16 | 871.96 |
| 182 | GDGDQFFT | -0.71 | Non-Toxin | -0.1 | 0.16 | 885.99 |
| 183 | GDGDQFFC | -0.49 | Non-Toxin | -0.07 | 0.16 | 888.02 |
| 184 | GDGDQFFN | -0.7 | Non-Toxin | -0.15 | 0.16 | 898.99 |
| 185 | GDGDQFFQ | -0.76 | Non-Toxin | -0.16 | 0.31 | 913.02 |
| 186 | GDGDQFWS | -0.84 | Non-Toxin | -0.14 | 0.16 | 911 |
| 187 | GDGDQFWT | -0.79 | Non-Toxin | -0.13 | 0.16 | 925.03 |
| 188 | GDGDQFWC | -0.44 | Non-Toxin | -0.1 | 0.16 | 927.06 |
| 189 | GDGDQFWN | -0.85 | Non-Toxin | -0.18 | 0.16 | 938.03 |
| 190 | GDGDQFWQ | -0.79 | Non-Toxin | -0.19 | 0.31 | 952.06 |
| 191 | GDGDQFYS | -0.86 | Non-Toxin | -0.18 | 0.16 | 887.96 |
| 192 | GDGDQFYT | -0.82 | Non-Toxin | -0.17 | 0.16 | 901.99 |
| 193 | GDGDQFYC | -0.67 | Non-Toxin | -0.14 | 0.16 | 904.02 |
| 194 | GDGDQFYN | -0.86 | Non-Toxin | -0.23 | 0.16 | 914.99 |
| 195 | GDGDQFYQ | -0.72 | Non-Toxin | -0.23 | 0.31 | 929.02 |
| 196 | GDGDQWFS | -0.83 | Non-Toxin | -0.14 | 0.16 | 911 |
| 197 | GDGDQWFT | -0.78 | Non-Toxin | -0.13 | 0.16 | 925.03 |
| 198 | GDGDQWFC | -0.57 | Non-Toxin | -0.1 | 0.16 | 927.06 |
| 199 | GDGDQWFN | -0.78 | Non-Toxin | -0.18 | 0.16 | 938.03 |
| 200 | GDGDQWFQ | -0.81 | Non-Toxin | -0.19 | 0.31 | 952.06 |
| 201 | GDGDQWWS | -0.79 | Non-Toxin | -0.17 | 0.16 | 950.04 |
| 202 | GDGDQWWT | -0.75 | Non-Toxin | -0.16 | 0.16 | 964.07 |
| 203 | GDGDQWWC | -0.4 | Non-Toxin | -0.13 | 0.16 | 966.1 |
| 204 | GDGDQWWN | -0.81 | Non-Toxin | -0.21 | 0.16 | 977.07 |
| 205 | GDGDQWWQ | -0.74 | Non-Toxin | -0.22 | 0.31 | 991.1 |
| 206 | GDGDQWYS | -0.68 | Non-Toxin | -0.21 | 0.16 | 927 |
| 207 | GDGDQWYT | -0.66 | Non-Toxin | -0.2 | 0.16 | 941.03 |
| 208 | GDGDQWYC | -0.46 | Non-Toxin | -0.17 | 0.16 | 943.06 |
| 209 | GDGDQWYN | -0.68 | Non-Toxin | -0.26 | 0.16 | 954.03 |
| 210 | GDGDQWYQ | -0.54 | Non-Toxin | -0.26 | 0.31 | 968.06 |
| 211 | GDGDQYFS | -0.86 | Non-Toxin | -0.18 | 0.16 | 887.96 |
| 212 | GDGDQYFT | -0.79 | Non-Toxin | -0.17 | 0.16 | 901.99 |
| 213 | GDGDQYFC | -0.61 | Non-Toxin | -0.14 | 0.16 | 904.02 |
| 214 | GDGDQYFN | -0.82 | Non-Toxin | -0.23 | 0.16 | 914.99 |
| 215 | GDGDQYFQ | -0.86 | Non-Toxin | -0.23 | 0.31 | 929.02 |
| 216 | GDGDQYWS | -0.92 | Non-Toxin | -0.21 | 0.16 | 927 |
| 217 | GDGDQYWT | -0.87 | Non-Toxin | -0.2 | 0.16 | 941.03 |
| 218 | GDGDQYWC | -0.52 | Non-Toxin | -0.17 | 0.16 | 943.06 |
| 219 | GDGDQYWN | -0.93 | Non-Toxin | -0.26 | 0.16 | 954.03 |
| 220 | GDGDQYWQ | -0.86 | Non-Toxin | -0.26 | 0.31 | 968.06 |
| 221 | GDGDQYYS | -0.84 | Non-Toxin | -0.25 | 0.16 | 903.96 |
| 222 | GDGDQYYT | -0.8 | Non-Toxin | -0.24 | 0.16 | 917.99 |
| 223 | GDGDQYYC | -0.58 | Non-Toxin | -0.22 | 0.16 | 920.02 |
| 224 | GDGDQYYN | -0.83 | Non-Toxin | -0.3 | 0.16 | 930.99 |
| 225 | GDGDQYYQ | -0.7 | Non-Toxin | -0.31 | 0.31 | 945.02 |
| 226 | GDGESFFS | -0.9 | Non-Toxin | -0.04 | 0.16 | 844.93 |
| 227 | GDGESFFT | -0.88 | Non-Toxin | -0.03 | 0.16 | 858.96 |
| 228 | GDGESFFC | -0.58 | Non-Toxin | 0 | 0.16 | 860.99 |
| 229 | GDGESFFN | -0.9 | Non-Toxin | -0.09 | 0.16 | 871.96 |
| 230 | GDGESFFQ | -0.9 | Non-Toxin | -0.09 | 0.32 | 885.99 |
| 231 | GDGESFWS | -1.12 | Non-Toxin | -0.07 | 0.16 | 883.97 |
| 232 | GDGESFWT | -0.99 | Non-Toxin | -0.06 | 0.16 | 898 |
| 233 | GDGESFWC | -0.46 | Non-Toxin | -0.03 | 0.16 | 900.03 |
| 234 | GDGESFWN | -1.11 | Non-Toxin | -0.12 | 0.16 | 911 |
| 235 | GDGESFWQ | -1 | Non-Toxin | -0.12 | 0.32 | 925.03 |
| 236 | GDGESFYS | -1.21 | Non-Toxin | -0.11 | 0.16 | 860.93 |
| 237 | GDGESFYT | -1.19 | Non-Toxin | -0.1 | 0.16 | 874.96 |
| 238 | GDGESFYC | -0.98 | Non-Toxin | -0.08 | 0.16 | 876.99 |
| 239 | GDGESFYN | -1.19 | Non-Toxin | -0.16 | 0.16 | 887.96 |
| 240 | GDGESFYQ | -0.94 | Non-Toxin | -0.17 | 0.32 | 901.99 |
| 241 | GDGESWFS | -1.2 | Non-Toxin | -0.07 | 0.16 | 883.97 |
| 242 | GDGESWFT | -1.16 | Non-Toxin | -0.06 | 0.16 | 898 |
| 243 | GDGESWFC | -0.86 | Non-Toxin | -0.03 | 0.16 | 900.03 |
| 244 | GDGESWFN | -1.16 | Non-Toxin | -0.12 | 0.16 | 911 |
| 245 | GDGESWFQ | -1.2 | Non-Toxin | -0.12 | 0.32 | 925.03 |
| 246 | GDGESWWS | -1.18 | Non-Toxin | -0.1 | 0.16 | 923.01 |
| 247 | GDGESWWT | -1.1 | Non-Toxin | -0.09 | 0.16 | 937.04 |
| 248 | GDGESWWC | -0.58 | Non-Toxin | -0.06 | 0.16 | 939.07 |
| 249 | GDGESWWN | -1.2 | Non-Toxin | -0.15 | 0.16 | 950.04 |
| 250 | GDGESWWQ | -1.09 | Non-Toxin | -0.15 | 0.32 | 964.07 |
| 251 | GDGESWYS | -1.01 | Non-Toxin | -0.14 | 0.16 | 899.97 |
| 252 | GDGESWYT | -1.02 | Non-Toxin | -0.13 | 0.16 | 914 |
| 253 | GDGESWYC | -0.73 | Non-Toxin | -0.11 | 0.16 | 916.03 |
| 254 | GDGESWYN | -1 | Non-Toxin | -0.19 | 0.16 | 927 |
| 255 | GDGESWYQ | -0.74 | Non-Toxin | -0.2 | 0.32 | 941.03 |
| 256 | GDGESYFS | -1.07 | Non-Toxin | -0.11 | 0.16 | 860.93 |
| 257 | GDGESYFT | -1.03 | Non-Toxin | -0.1 | 0.16 | 874.96 |
| 258 | GDGESYFC | -0.74 | Non-Toxin | -0.08 | 0.16 | 876.99 |
| 259 | GDGESYFN | -1.07 | Non-Toxin | -0.16 | 0.16 | 887.96 |
| 260 | GDGESYFQ | -1.1 | Non-Toxin | -0.17 | 0.32 | 901.99 |
| 261 | GDGESYWS | -1.18 | Non-Toxin | -0.14 | 0.16 | 899.97 |
| 262 | GDGESYWT | -1.11 | Non-Toxin | -0.13 | 0.16 | 914 |
| 263 | GDGESYWC | -0.58 | Non-Toxin | -0.11 | 0.16 | 916.03 |
| 264 | GDGESYWN | -1.21 | Non-Toxin | -0.19 | 0.16 | 927 |
| 265 | GDGESYWQ | -1.09 | Non-Toxin | -0.2 | 0.32 | 941.03 |
| 266 | GDGESYYS | -1.02 | Non-Toxin | -0.19 | 0.16 | 876.93 |
| 267 | GDGESYYT | -1.04 | Non-Toxin | -0.18 | 0.16 | 890.96 |
| 268 | GDGESYYC | -0.68 | Non-Toxin | -0.15 | 0.16 | 892.99 |
| 269 | GDGESYYN | -1.02 | Non-Toxin | -0.23 | 0.16 | 903.96 |
| 270 | GDGESYYQ | -0.74 | Non-Toxin | -0.24 | 0.32 | 917.99 |
| 271 | GDGETFFS | -1.18 | Non-Toxin | -0.03 | 0.16 | 858.96 |
| 272 | GDGETFFT | -1.19 | Non-Toxin | -0.02 | 0.16 | 872.99 |
| 273 | GDGETFFC | -0.85 | Non-Toxin | 0.01 | 0.16 | 875.02 |
| 274 | GDGETFFN | -1.11 | Non-Toxin | -0.08 | 0.16 | 885.99 |
| 275 | GDGETFFQ | -1.21 | Non-Toxin | -0.08 | 0.32 | 900.02 |
| 276 | GDGETFWS | -1.32 | Non-Toxin | -0.06 | 0.16 | 898 |
| 277 | GDGETFWT | -1.23 | Non-Toxin | -0.05 | 0.16 | 912.03 |
| 278 | GDGETFWC | -0.72 | Non-Toxin | -0.02 | 0.16 | 914.06 |
| 279 | GDGETFWN | -1.35 | Non-Toxin | -0.11 | 0.16 | 925.03 |
| 280 | GDGETFWQ | -1.24 | Non-Toxin | -0.11 | 0.32 | 939.06 |
| 281 | GDGETFYS | -1.42 | Non-Toxin | -0.1 | 0.16 | 874.96 |
| 282 | GDGETFYT | -1.41 | Non-Toxin | -0.09 | 0.16 | 888.99 |
| 283 | GDGETFYC | -1.2 | Non-Toxin | -0.07 | 0.16 | 891.02 |
| 284 | GDGETFYN | -1.41 | Non-Toxin | -0.15 | 0.16 | 901.99 |
| 285 | GDGETFYQ | -1.18 | Non-Toxin | -0.16 | 0.32 | 916.02 |
| 286 | GDGETWFS | -1.18 | Non-Toxin | -0.06 | 0.16 | 898 |
| 287 | GDGETWFT | -1.18 | Non-Toxin | -0.05 | 0.16 | 912.03 |
| 288 | GDGETWFC | -0.87 | Non-Toxin | -0.02 | 0.16 | 914.06 |
| 289 | GDGETWFN | -1.14 | Non-Toxin | -0.11 | 0.16 | 925.03 |
| 290 | GDGETWFQ | -1.2 | Non-Toxin | -0.11 | 0.32 | 939.06 |
| 291 | GDGETWWS | -1.17 | Non-Toxin | -0.09 | 0.16 | 937.04 |
| 292 | GDGETWWT | -1.11 | Non-Toxin | -0.08 | 0.16 | 951.07 |
| 293 | GDGETWWC | -0.55 | Non-Toxin | -0.05 | 0.16 | 953.1 |
| 294 | GDGETWWN | -1.2 | Non-Toxin | -0.14 | 0.16 | 964.07 |
| 295 | GDGETWWQ | -1.09 | Non-Toxin | -0.14 | 0.32 | 978.1 |
| 296 | GDGETWYS | -1.04 | Non-Toxin | -0.13 | 0.16 | 914 |
| 297 | GDGETWYT | -1.02 | Non-Toxin | -0.12 | 0.16 | 928.03 |
| 298 | GDGETWYC | -0.76 | Non-Toxin | -0.1 | 0.16 | 930.06 |
| 299 | GDGETWYN | -1.02 | Non-Toxin | -0.18 | 0.16 | 941.03 |
| 300 | GDGETWYQ | -0.77 | Non-Toxin | -0.19 | 0.32 | 955.06 |
| 301 | GDGETYFS | -1.03 | Non-Toxin | -0.1 | 0.16 | 874.96 |
| 302 | GDGETYFT | -1.01 | Non-Toxin | -0.09 | 0.16 | 888.99 |
| 303 | GDGETYFC | -0.73 | Non-Toxin | -0.07 | 0.16 | 891.02 |
| 304 | GDGETYFN | -1.04 | Non-Toxin | -0.15 | 0.16 | 901.99 |
| 305 | GDGETYFQ | -1.08 | Non-Toxin | -0.16 | 0.32 | 916.02 |
| 306 | GDGETYWS | -1.14 | Non-Toxin | -0.13 | 0.16 | 914 |
| 307 | GDGETYWT | -1.09 | Non-Toxin | -0.12 | 0.16 | 928.03 |
| 308 | GDGETYWC | -0.54 | Non-Toxin | -0.1 | 0.16 | 930.06 |
| 309 | GDGETYWN | -1.19 | Non-Toxin | -0.18 | 0.16 | 941.03 |
| 310 | GDGETYWQ | -1.07 | Non-Toxin | -0.19 | 0.32 | 955.06 |
| 311 | GDGETYYS | -1.05 | Non-Toxin | -0.18 | 0.16 | 890.96 |
| 312 | GDGETYYT | -1.01 | Non-Toxin | -0.17 | 0.16 | 904.99 |
| 313 | GDGETYYC | -0.72 | Non-Toxin | -0.14 | 0.16 | 907.02 |
| 314 | GDGETYYN | -1.03 | Non-Toxin | -0.22 | 0.16 | 917.99 |
| 315 | GDGETYYQ | -0.77 | Non-Toxin | -0.23 | 0.32 | 932.02 |
| 316 | GDGECFFS | -0.41 | Non-Toxin | 0 | 0.16 | 860.99 |
| 317 | GDGECFFT | -0.34 | Non-Toxin | 0.01 | 0.16 | 875.02 |
| 318 | GDGECFFC | 0.01 | Toxin | 0.03 | 0.16 | 877.05 |
| 319 | GDGECFFN | -0.34 | Non-Toxin | -0.05 | 0.16 | 888.02 |
| 320 | GDGECFFQ | -0.43 | Non-Toxin | -0.06 | 0.32 | 902.05 |
| 321 | GDGECFWS | -0.69 | Non-Toxin | -0.03 | 0.16 | 900.03 |
| 322 | GDGECFWT | -0.63 | Non-Toxin | -0.02 | 0.16 | 914.06 |
| 323 | GDGECFWC | -0.09 | Non-Toxin | 0.01 | 0.16 | 916.09 |
| 324 | GDGECFWN | -0.72 | Non-Toxin | -0.08 | 0.16 | 927.06 |
| 325 | GDGECFWQ | -0.6 | Non-Toxin | -0.09 | 0.32 | 941.09 |
| 326 | GDGECFYS | -0.68 | Non-Toxin | -0.08 | 0.16 | 876.99 |
| 327 | GDGECFYT | -0.66 | Non-Toxin | -0.07 | 0.16 | 891.02 |
| 328 | GDGECFYC | -0.54 | Non-Toxin | -0.04 | 0.16 | 893.05 |
| 329 | GDGECFYN | -0.67 | Non-Toxin | -0.12 | 0.16 | 904.02 |
| 330 | GDGECFYQ | -0.44 | Non-Toxin | -0.13 | 0.32 | 918.05 |
| 331 | GDGECWFS | -0.37 | Non-Toxin | -0.03 | 0.16 | 900.03 |
| 332 | GDGECWFT | -0.3 | Non-Toxin | -0.02 | 0.16 | 914.06 |
| 333 | GDGECWFC | 0 | Toxin | 0.01 | 0.16 | 916.09 |
| 334 | GDGECWFN | -0.29 | Non-Toxin | -0.08 | 0.16 | 927.06 |
| 335 | GDGECWFQ | -0.37 | Non-Toxin | -0.09 | 0.32 | 941.09 |
| 336 | GDGECWWS | -0.31 | Non-Toxin | -0.06 | 0.16 | 939.07 |
| 337 | GDGECWWT | -0.27 | Non-Toxin | -0.05 | 0.16 | 953.1 |
| 338 | GDGECWWC | 0.29 | Toxin | -0.02 | 0.16 | 955.13 |
| 339 | GDGECWWN | -0.35 | Non-Toxin | -0.11 | 0.16 | 966.1 |
| 340 | GDGECWWQ | -0.24 | Non-Toxin | -0.12 | 0.32 | 980.13 |
| 341 | GDGECWYS | -0.18 | Non-Toxin | -0.11 | 0.16 | 916.03 |
| 342 | GDGECWYT | -0.14 | Non-Toxin | -0.1 | 0.16 | 930.06 |
| 343 | GDGECWYC | 0.09 | Toxin | -0.07 | 0.16 | 932.09 |
| 344 | GDGECWYN | -0.16 | Non-Toxin | -0.15 | 0.16 | 943.06 |
| 345 | GDGECWYQ | 0.1 | Toxin | -0.16 | 0.32 | 957.09 |
| 346 | GDGECYFS | -0.32 | Non-Toxin | -0.08 | 0.16 | 876.99 |
| 347 | GDGECYFT | -0.23 | Non-Toxin | -0.07 | 0.16 | 891.02 |
| 348 | GDGECYFC | 0.03 | Toxin | -0.04 | 0.16 | 893.05 |
| 349 | GDGECYFN | -0.26 | Non-Toxin | -0.12 | 0.16 | 904.02 |
| 350 | GDGECYFQ | -0.34 | Non-Toxin | -0.13 | 0.32 | 918.05 |
| 351 | GDGECYWS | -0.31 | Non-Toxin | -0.11 | 0.16 | 916.03 |
| 352 | GDGECYWT | -0.27 | Non-Toxin | -0.1 | 0.16 | 930.06 |
| 353 | GDGECYWC | 0.24 | Toxin | -0.07 | 0.16 | 932.09 |
| 354 | GDGECYWN | -0.38 | Non-Toxin | -0.15 | 0.16 | 943.06 |
| 355 | GDGECYWQ | -0.25 | Non-Toxin | -0.16 | 0.32 | 957.09 |
| 356 | GDGECYYS | -0.2 | Non-Toxin | -0.15 | 0.16 | 892.99 |
| 357 | GDGECYYT | -0.22 | Non-Toxin | -0.14 | 0.16 | 907.02 |
| 358 | GDGECYYC | 0.07 | Toxin | -0.11 | 0.16 | 909.05 |
| 359 | GDGECYYN | -0.17 | Non-Toxin | -0.2 | 0.16 | 920.02 |
| 360 | GDGECYYQ | 0.05 | Toxin | -0.2 | 0.32 | 934.05 |
| 361 | GDGENFFS | -1.16 | Non-Toxin | -0.09 | 0.16 | 871.96 |
| 362 | GDGENFFT | -1.08 | Non-Toxin | -0.08 | 0.16 | 885.99 |
| 363 | GDGENFFC | -0.77 | Non-Toxin | -0.05 | 0.16 | 888.02 |
| 364 | GDGENFFN | -1.07 | Non-Toxin | -0.14 | 0.16 | 898.99 |
| 365 | GDGENFFQ | -1.14 | Non-Toxin | -0.14 | 0.32 | 913.02 |
| 366 | GDGENFWS | -1.28 | Non-Toxin | -0.12 | 0.16 | 911 |
| 367 | GDGENFWT | -1.22 | Non-Toxin | -0.11 | 0.16 | 925.03 |
| 368 | GDGENFWC | -0.71 | Non-Toxin | -0.08 | 0.16 | 927.06 |
| 369 | GDGENFWN | -1.32 | Non-Toxin | -0.17 | 0.16 | 938.03 |
| 370 | GDGENFWQ | -1.21 | Non-Toxin | -0.17 | 0.32 | 952.06 |
| 371 | GDGENFYS | -1.34 | Non-Toxin | -0.16 | 0.16 | 887.96 |
| 372 | GDGENFYT | -1.31 | Non-Toxin | -0.15 | 0.16 | 901.99 |
| 373 | GDGENFYC | -1.11 | Non-Toxin | -0.12 | 0.16 | 904.02 |
| 374 | GDGENFYN | -1.34 | Non-Toxin | -0.21 | 0.16 | 914.99 |
| 375 | GDGENFYQ | -1.12 | Non-Toxin | -0.22 | 0.32 | 929.02 |
| 376 | GDGENWFS | -1.03 | Non-Toxin | -0.12 | 0.16 | 911 |
| 377 | GDGENWFT | -0.98 | Non-Toxin | -0.11 | 0.16 | 925.03 |
| 378 | GDGENWFC | -0.68 | Non-Toxin | -0.08 | 0.16 | 927.06 |
| 379 | GDGENWFN | -0.96 | Non-Toxin | -0.17 | 0.16 | 938.03 |
| 380 | GDGENWFQ | -1.02 | Non-Toxin | -0.17 | 0.32 | 952.06 |
| 381 | GDGENWWS | -0.99 | Non-Toxin | -0.15 | 0.16 | 950.04 |
| 382 | GDGENWWT | -0.93 | Non-Toxin | -0.14 | 0.16 | 964.07 |
| 383 | GDGENWWC | -0.41 | Non-Toxin | -0.11 | 0.16 | 966.1 |
| 384 | GDGENWWN | -1.02 | Non-Toxin | -0.2 | 0.16 | 977.07 |
| 385 | GDGENWWQ | -0.92 | Non-Toxin | -0.2 | 0.32 | 991.1 |
| 386 | GDGENWYS | -0.84 | Non-Toxin | -0.19 | 0.16 | 927 |
| 387 | GDGENWYT | -0.84 | Non-Toxin | -0.18 | 0.16 | 941.03 |
| 388 | GDGENWYC | -0.57 | Non-Toxin | -0.15 | 0.16 | 943.06 |
| 389 | GDGENWYN | -0.84 | Non-Toxin | -0.24 | 0.16 | 954.03 |
| 390 | GDGENWYQ | -0.59 | Non-Toxin | -0.24 | 0.32 | 968.06 |
| 391 | GDGENYFS | -0.94 | Non-Toxin | -0.16 | 0.16 | 887.96 |
| 392 | GDGENYFT | -0.89 | Non-Toxin | -0.15 | 0.16 | 901.99 |
| 393 | GDGENYFC | -0.6 | Non-Toxin | -0.12 | 0.16 | 904.02 |
| 394 | GDGENYFN | -0.91 | Non-Toxin | -0.21 | 0.16 | 914.99 |
| 395 | GDGENYFQ | -0.95 | Non-Toxin | -0.22 | 0.32 | 929.02 |
| 396 | GDGENYWS | -1.04 | Non-Toxin | -0.19 | 0.16 | 927 |
| 397 | GDGENYWT | -0.98 | Non-Toxin | -0.18 | 0.16 | 941.03 |
| 398 | GDGENYWC | -0.46 | Non-Toxin | -0.15 | 0.16 | 943.06 |
| 399 | GDGENYWN | -1.09 | Non-Toxin | -0.24 | 0.16 | 954.03 |
| 400 | GDGENYWQ | -0.97 | Non-Toxin | -0.24 | 0.32 | 968.06 |
| 401 | GDGENYYS | -0.9 | Non-Toxin | -0.24 | 0.16 | 903.96 |
| 402 | GDGENYYT | -0.86 | Non-Toxin | -0.23 | 0.16 | 917.99 |
| 403 | GDGENYYC | -0.5 | Non-Toxin | -0.2 | 0.16 | 920.02 |
| 404 | GDGENYYN | -0.92 | Non-Toxin | -0.28 | 0.16 | 930.99 |
| 405 | GDGENYYQ | -0.65 | Non-Toxin | -0.29 | 0.32 | 945.02 |
| 406 | GDGEQFFS | -0.92 | Non-Toxin | -0.09 | 0.32 | 885.99 |
| 407 | GDGEQFFT | -0.84 | Non-Toxin | -0.08 | 0.32 | 900.02 |
| 408 | GDGEQFFC | -0.54 | Non-Toxin | -0.06 | 0.32 | 902.05 |
| 409 | GDGEQFFN | -0.83 | Non-Toxin | -0.14 | 0.32 | 913.02 |
| 410 | GDGEQFFQ | -0.92 | Non-Toxin | -0.15 | 0.47 | 927.05 |
| 411 | GDGEQFWS | -1.07 | Non-Toxin | -0.12 | 0.32 | 925.03 |
| 412 | GDGEQFWT | -0.98 | Non-Toxin | -0.11 | 0.32 | 939.06 |
| 413 | GDGEQFWC | -0.47 | Non-Toxin | -0.09 | 0.32 | 941.09 |
| 414 | GDGEQFWN | -1.09 | Non-Toxin | -0.17 | 0.32 | 952.06 |
| 415 | GDGEQFWQ | -0.98 | Non-Toxin | -0.18 | 0.47 | 966.09 |
| 416 | GDGEQFYS | -1.14 | Non-Toxin | -0.17 | 0.32 | 901.99 |
| 417 | GDGEQFYT | -1.1 | Non-Toxin | -0.16 | 0.32 | 916.02 |
| 418 | GDGEQFYC | -0.9 | Non-Toxin | -0.13 | 0.32 | 918.05 |
| 419 | GDGEQFYN | -1.13 | Non-Toxin | -0.22 | 0.32 | 929.02 |
| 420 | GDGEQFYQ | -0.88 | Non-Toxin | -0.22 | 0.47 | 943.05 |
| 421 | GDGEQWFS | -1.01 | Non-Toxin | -0.12 | 0.32 | 925.03 |
| 422 | GDGEQWFT | -0.95 | Non-Toxin | -0.11 | 0.32 | 939.06 |
| 423 | GDGEQWFC | -0.66 | Non-Toxin | -0.09 | 0.32 | 941.09 |
| 424 | GDGEQWFN | -0.95 | Non-Toxin | -0.17 | 0.32 | 952.06 |
| 425 | GDGEQWFQ | -1 | Non-Toxin | -0.18 | 0.47 | 966.09 |
| 426 | GDGEQWWS | -1 | Non-Toxin | -0.15 | 0.32 | 964.07 |
| 427 | GDGEQWWT | -0.92 | Non-Toxin | -0.14 | 0.32 | 978.1 |
| 428 | GDGEQWWC | -0.4 | Non-Toxin | -0.12 | 0.32 | 980.13 |
| 429 | GDGEQWWN | -1.02 | Non-Toxin | -0.2 | 0.32 | 991.1 |
| 430 | GDGEQWWQ | -0.91 | Non-Toxin | -0.21 | 0.47 | 1005.13 |
| 431 | GDGEQWYS | -0.83 | Non-Toxin | -0.2 | 0.32 | 941.03 |
| 432 | GDGEQWYT | -0.82 | Non-Toxin | -0.19 | 0.32 | 955.06 |
| 433 | GDGEQWYC | -0.55 | Non-Toxin | -0.16 | 0.32 | 957.09 |
| 434 | GDGEQWYN | -0.82 | Non-Toxin | -0.24 | 0.32 | 968.06 |
| 435 | GDGEQWYQ | -0.59 | Non-Toxin | -0.25 | 0.47 | 982.09 |
| 436 | GDGEQYFS | -1.11 | Non-Toxin | -0.17 | 0.32 | 901.99 |
| 437 | GDGEQYFT | -1.02 | Non-Toxin | -0.16 | 0.32 | 916.02 |
| 438 | GDGEQYFC | -0.76 | Non-Toxin | -0.13 | 0.32 | 918.05 |
| 439 | GDGEQYFN | -1.06 | Non-Toxin | -0.22 | 0.32 | 929.02 |
| 440 | GDGEQYFQ | -1.12 | Non-Toxin | -0.22 | 0.47 | 943.05 |
| 441 | GDGEQYWS | -1.19 | Non-Toxin | -0.2 | 0.32 | 941.03 |
| 442 | GDGEQYWT | -1.11 | Non-Toxin | -0.19 | 0.32 | 955.06 |
| 443 | GDGEQYWC | -0.59 | Non-Toxin | -0.16 | 0.32 | 957.09 |
| 444 | GDGEQYWN | -1.22 | Non-Toxin | -0.24 | 0.32 | 968.06 |
| 445 | GDGEQYWQ | -1.09 | Non-Toxin | -0.25 | 0.47 | 982.09 |
| 446 | GDGEQYYS | -1.08 | Non-Toxin | -0.24 | 0.32 | 917.99 |
| 447 | GDGEQYYT | -1.04 | Non-Toxin | -0.23 | 0.32 | 932.02 |
| 448 | GDGEQYYC | -0.74 | Non-Toxin | -0.2 | 0.32 | 934.05 |
| 449 | GDGEQYYN | -1.05 | Non-Toxin | -0.29 | 0.32 | 945.02 |
| 450 | GDGEQYYQ | -0.83 | Non-Toxin | -0.29 | 0.47 | 959.05 |
| 451 | GDPDSFFS | -0.78 | Non-Toxin | -0.08 | 0 | 870.96 |
| 452 | GDPDSFFT | -0.72 | Non-Toxin | -0.07 | 0 | 884.99 |
| 453 | GDPDSFFC | -0.43 | Non-Toxin | -0.04 | 0 | 887.02 |
| 454 | GDPDSFFN | -0.75 | Non-Toxin | -0.13 | 0 | 897.99 |
| 455 | GDPDSFFQ | -0.77 | Non-Toxin | -0.13 | 0.16 | 912.02 |
| 456 | GDPDSFWS | -0.98 | Non-Toxin | -0.11 | 0 | 910 |
| 457 | GDPDSFWT | -0.89 | Non-Toxin | -0.1 | 0 | 924.03 |
| 458 | GDPDSFWC | -0.33 | Non-Toxin | -0.07 | 0 | 926.06 |
| 459 | GDPDSFWN | -0.99 | Non-Toxin | -0.16 | 0 | 937.03 |
| 460 | GDPDSFWQ | -0.89 | Non-Toxin | -0.16 | 0.16 | 951.06 |
| 461 | GDPDSFYS | -1.07 | Non-Toxin | -0.15 | 0 | 886.96 |
| 462 | GDPDSFYT | -1.07 | Non-Toxin | -0.14 | 0 | 900.99 |
| 463 | GDPDSFYC | -0.95 | Non-Toxin | -0.12 | 0 | 903.02 |
| 464 | GDPDSFYN | -1.07 | Non-Toxin | -0.2 | 0 | 913.99 |
| 465 | GDPDSFYQ | -0.92 | Non-Toxin | -0.21 | 0.16 | 928.02 |
| 466 | GDPDSWFS | -1.09 | Non-Toxin | -0.11 | 0 | 910 |
| 467 | GDPDSWFT | -1.02 | Non-Toxin | -0.1 | 0 | 924.03 |
| 468 | GDPDSWFC | -0.72 | Non-Toxin | -0.07 | 0 | 926.06 |
| 469 | GDPDSWFN | -1.02 | Non-Toxin | -0.16 | 0 | 937.03 |
| 470 | GDPDSWFQ | -1.08 | Non-Toxin | -0.16 | 0.16 | 951.06 |
| 471 | GDPDSWWS | -1.01 | Non-Toxin | -0.14 | 0 | 949.04 |
| 472 | GDPDSWWT | -0.98 | Non-Toxin | -0.13 | 0 | 963.07 |
| 473 | GDPDSWWC | -0.42 | Non-Toxin | -0.1 | 0 | 965.1 |
| 474 | GDPDSWWN | -1.05 | Non-Toxin | -0.19 | 0 | 976.07 |
| 475 | GDPDSWWQ | -0.95 | Non-Toxin | -0.19 | 0.16 | 990.1 |
| 476 | GDPDSWYS | -0.79 | Non-Toxin | -0.18 | 0 | 926 |
| 477 | GDPDSWYT | -0.83 | Non-Toxin | -0.17 | 0 | 940.03 |
| 478 | GDPDSWYC | -0.63 | Non-Toxin | -0.15 | 0 | 942.06 |
| 479 | GDPDSWYN | -0.81 | Non-Toxin | -0.23 | 0 | 953.03 |
| 480 | GDPDSWYQ | -0.64 | Non-Toxin | -0.24 | 0.16 | 967.06 |
| 481 | GDPDSYFS | -0.92 | Non-Toxin | -0.15 | 0 | 886.96 |
| 482 | GDPDSYFT | -0.84 | Non-Toxin | -0.14 | 0 | 900.99 |
| 483 | GDPDSYFC | -0.56 | Non-Toxin | -0.12 | 0 | 903.02 |
| 484 | GDPDSYFN | -0.89 | Non-Toxin | -0.2 | 0 | 913.99 |
| 485 | GDPDSYFQ | -0.94 | Non-Toxin | -0.21 | 0.16 | 928.02 |
| 486 | GDPDSYWS | -0.98 | Non-Toxin | -0.18 | 0 | 926 |
| 487 | GDPDSYWT | -0.96 | Non-Toxin | -0.17 | 0 | 940.03 |
| 488 | GDPDSYWC | -0.39 | Non-Toxin | -0.15 | 0 | 942.06 |
| 489 | GDPDSYWN | -1.03 | Non-Toxin | -0.23 | 0 | 953.03 |
| 490 | GDPDSYWQ | -0.92 | Non-Toxin | -0.24 | 0.16 | 967.06 |
| 491 | GDPDSYYS | -0.76 | Non-Toxin | -0.23 | 0 | 902.96 |
| 492 | GDPDSYYT | -0.8 | Non-Toxin | -0.22 | 0 | 916.99 |
| 493 | GDPDSYYC | -0.53 | Non-Toxin | -0.19 | 0 | 919.02 |
| 494 | GDPDSYYN | -0.78 | Non-Toxin | -0.28 | 0 | 929.99 |
| 495 | GDPDSYYQ | -0.6 | Non-Toxin | -0.28 | 0.16 | 944.02 |
| 496 | GDPDTFFS | -0.63 | Non-Toxin | -0.07 | 0 | 884.99 |
| 497 | GDPDTFFT | -0.54 | Non-Toxin | -0.06 | 0 | 899.02 |
| 498 | GDPDTFFC | -0.19 | Non-Toxin | -0.03 | 0 | 901.05 |
| 499 | GDPDTFFN | -0.53 | Non-Toxin | -0.12 | 0 | 912.02 |
| 500 | GDPDTFFQ | -0.63 | Non-Toxin | -0.12 | 0.16 | 926.05 |
| 501 | GDPDTFWS | -0.77 | Non-Toxin | -0.1 | 0 | 924.03 |
| 502 | GDPDTFWT | -0.73 | Non-Toxin | -0.09 | 0 | 938.06 |
| 503 | GDPDTFWC | -0.2 | Non-Toxin | -0.06 | 0 | 940.09 |
| 504 | GDPDTFWN | -0.83 | Non-Toxin | -0.15 | 0 | 951.06 |
| 505 | GDPDTFWQ | -0.72 | Non-Toxin | -0.15 | 0.16 | 965.09 |
| 506 | GDPDTFYS | -0.82 | Non-Toxin | -0.14 | 0 | 900.99 |
| 507 | GDPDTFYT | -0.88 | Non-Toxin | -0.13 | 0 | 915.02 |
| 508 | GDPDTFYC | -0.67 | Non-Toxin | -0.11 | 0 | 917.05 |
| 509 | GDPDTFYN | -0.88 | Non-Toxin | -0.19 | 0 | 928.02 |
| 510 | GDPDTFYQ | -0.72 | Non-Toxin | -0.2 | 0.16 | 942.05 |
| 511 | GDPDTWFS | -0.79 | Non-Toxin | -0.1 | 0 | 924.03 |
| 512 | GDPDTWFT | -0.7 | Non-Toxin | -0.09 | 0 | 938.06 |
| 513 | GDPDTWFC | -0.37 | Non-Toxin | -0.06 | 0 | 940.09 |
| 514 | GDPDTWFN | -0.71 | Non-Toxin | -0.15 | 0 | 951.06 |
| 515 | GDPDTWFQ | -0.79 | Non-Toxin | -0.15 | 0.16 | 965.09 |
| 516 | GDPDTWWS | -0.72 | Non-Toxin | -0.13 | 0 | 963.07 |
| 517 | GDPDTWWT | -0.71 | Non-Toxin | -0.12 | 0 | 977.1 |
| 518 | GDPDTWWC | -0.13 | Non-Toxin | -0.09 | 0 | 979.13 |
| 519 | GDPDTWWN | -0.77 | Non-Toxin | -0.18 | 0 | 990.1 |
| 520 | GDPDTWWQ | -0.67 | Non-Toxin | -0.18 | 0.16 | 1004.13 |
| 521 | GDPDTWYS | -0.54 | Non-Toxin | -0.17 | 0 | 940.03 |
| 522 | GDPDTWYT | -0.58 | Non-Toxin | -0.16 | 0 | 954.06 |
| 523 | GDPDTWYC | -0.32 | Non-Toxin | -0.14 | 0 | 956.09 |
| 524 | GDPDTWYN | -0.57 | Non-Toxin | -0.22 | 0 | 967.06 |
| 525 | GDPDTWYQ | -0.39 | Non-Toxin | -0.23 | 0.16 | 981.09 |
| 526 | GDPDTYFS | -0.65 | Non-Toxin | -0.14 | 0 | 900.99 |
| 527 | GDPDTYFT | -0.54 | Non-Toxin | -0.13 | 0 | 915.02 |
| 528 | GDPDTYFC | -0.25 | Non-Toxin | -0.11 | 0 | 917.05 |
| 529 | GDPDTYFN | -0.63 | Non-Toxin | -0.19 | 0 | 928.02 |
| 530 | GDPDTYFQ | -0.67 | Non-Toxin | -0.2 | 0.16 | 942.05 |
| 531 | GDPDTYWS | -0.71 | Non-Toxin | -0.17 | 0 | 940.03 |
| 532 | GDPDTYWT | -0.71 | Non-Toxin | -0.16 | 0 | 954.06 |
| 533 | GDPDTYWC | -0.15 | Non-Toxin | -0.14 | 0 | 956.09 |
| 534 | GDPDTYWN | -0.78 | Non-Toxin | -0.22 | 0 | 967.06 |
| 535 | GDPDTYWQ | -0.67 | Non-Toxin | -0.23 | 0.16 | 981.09 |
| 536 | GDPDTYYS | -0.57 | Non-Toxin | -0.22 | 0 | 916.99 |
| 537 | GDPDTYYT | -0.58 | Non-Toxin | -0.21 | 0 | 931.02 |
| 538 | GDPDTYYC | -0.3 | Non-Toxin | -0.18 | 0 | 933.05 |
| 539 | GDPDTYYN | -0.6 | Non-Toxin | -0.27 | 0 | 944.02 |
| 540 | GDPDTYYQ | -0.41 | Non-Toxin | -0.27 | 0.16 | 958.05 |
| 541 | GDPDCFFS | -0.34 | Non-Toxin | -0.04 | 0 | 887.02 |
| 542 | GDPDCFFT | -0.25 | Non-Toxin | -0.03 | 0 | 901.05 |
| 543 | GDPDCFFC | 0.14 | Toxin | -0.01 | 0 | 903.08 |
| 544 | GDPDCFFN | -0.27 | Non-Toxin | -0.09 | 0 | 914.05 |
| 545 | GDPDCFFQ | -0.36 | Non-Toxin | -0.1 | 0.16 | 928.08 |
| 546 | GDPDCFWS | -0.61 | Non-Toxin | -0.07 | 0 | 926.06 |
| 547 | GDPDCFWT | -0.6 | Non-Toxin | -0.06 | 0 | 940.09 |
| 548 | GDPDCFWC | -0.04 | Non-Toxin | -0.04 | 0 | 942.12 |
| 549 | GDPDCFWN | -0.67 | Non-Toxin | -0.12 | 0 | 953.09 |
| 550 | GDPDCFWQ | -0.57 | Non-Toxin | -0.13 | 0.16 | 967.12 |
| 551 | GDPDCFYS | -0.62 | Non-Toxin | -0.12 | 0 | 903.02 |
| 552 | GDPDCFYT | -0.67 | Non-Toxin | -0.11 | 0 | 917.05 |
| 553 | GDPDCFYC | -0.55 | Non-Toxin | -0.08 | 0 | 919.08 |
| 554 | GDPDCFYN | -0.66 | Non-Toxin | -0.16 | 0 | 930.05 |
| 555 | GDPDCFYQ | -0.5 | Non-Toxin | -0.17 | 0.16 | 944.08 |
| 556 | GDPDCWFS | -0.36 | Non-Toxin | -0.07 | 0 | 926.06 |
| 557 | GDPDCWFT | -0.27 | Non-Toxin | -0.06 | 0 | 940.09 |
| 558 | GDPDCWFC | 0.09 | Toxin | -0.04 | 0 | 942.12 |
| 559 | GDPDCWFN | -0.26 | Non-Toxin | -0.12 | 0 | 953.09 |
| 560 | GDPDCWFQ | -0.35 | Non-Toxin | -0.13 | 0.16 | 967.12 |
| 561 | GDPDCWWS | -0.28 | Non-Toxin | -0.1 | 0 | 965.1 |
| 562 | GDPDCWWT | -0.28 | Non-Toxin | -0.09 | 0 | 979.13 |
| 563 | GDPDCWWC | 0.29 | Toxin | -0.07 | 0 | 981.16 |
| 564 | GDPDCWWN | -0.34 | Non-Toxin | -0.15 | 0 | 992.13 |
| 565 | GDPDCWWQ | -0.25 | Non-Toxin | -0.16 | 0.16 | 1006.16 |
| 566 | GDPDCWYS | -0.12 | Non-Toxin | -0.15 | 0 | 942.06 |
| 567 | GDPDCWYT | -0.15 | Non-Toxin | -0.14 | 0 | 956.09 |
| 568 | GDPDCWYC | 0.08 | Toxin | -0.11 | 0 | 958.12 |
| 569 | GDPDCWYN | -0.15 | Non-Toxin | -0.19 | 0 | 969.09 |
| 570 | GDPDCWYQ | 0.04 | Toxin | -0.2 | 0.16 | 983.12 |
| 571 | GDPDCYFS | -0.24 | Non-Toxin | -0.12 | 0 | 903.02 |
| 572 | GDPDCYFT | -0.13 | Non-Toxin | -0.11 | 0 | 917.05 |
| 573 | GDPDCYFC | 0.18 | Toxin | -0.08 | 0 | 919.08 |
| 574 | GDPDCYFN | -0.16 | Non-Toxin | -0.16 | 0 | 930.05 |
| 575 | GDPDCYFQ | -0.25 | Non-Toxin | -0.17 | 0.16 | 944.08 |
| 576 | GDPDCYWS | -0.17 | Non-Toxin | -0.15 | 0 | 942.06 |
| 577 | GDPDCYWT | -0.18 | Non-Toxin | -0.14 | 0 | 956.09 |
| 578 | GDPDCYWC | 0.35 | Toxin | -0.11 | 0 | 958.12 |
| 579 | GDPDCYWN | -0.27 | Non-Toxin | -0.19 | 0 | 969.09 |
| 580 | GDPDCYWQ | -0.15 | Non-Toxin | -0.2 | 0.16 | 983.12 |
| 581 | GDPDCYYS | -0.06 | Non-Toxin | -0.19 | 0 | 919.02 |
| 582 | GDPDCYYT | -0.15 | Non-Toxin | -0.18 | 0 | 933.05 |
| 583 | GDPDCYYC | 0.14 | Toxin | -0.15 | 0 | 935.08 |
| 584 | GDPDCYYN | -0.08 | Non-Toxin | -0.24 | 0 | 946.05 |
| 585 | GDPDCYYQ | 0.06 | Toxin | -0.24 | 0.16 | 960.08 |
| 586 | GDPDNFFS | -0.9 | Non-Toxin | -0.13 | 0 | 897.99 |
| 587 | GDPDNFFT | -0.81 | Non-Toxin | -0.12 | 0 | 912.02 |
| 588 | GDPDNFFC | -0.47 | Non-Toxin | -0.09 | 0 | 914.05 |
| 589 | GDPDNFFN | -0.82 | Non-Toxin | -0.18 | 0 | 925.02 |
| 590 | GDPDNFFQ | -0.9 | Non-Toxin | -0.18 | 0.16 | 939.05 |
| 591 | GDPDNFWS | -1.05 | Non-Toxin | -0.16 | 0 | 937.03 |
| 592 | GDPDNFWT | -1.02 | Non-Toxin | -0.15 | 0 | 951.06 |
| 593 | GDPDNFWC | -0.49 | Non-Toxin | -0.12 | 0 | 953.09 |
| 594 | GDPDNFWN | -1.1 | Non-Toxin | -0.21 | 0 | 964.06 |
| 595 | GDPDNFWQ | -1 | Non-Toxin | -0.21 | 0.16 | 978.09 |
| 596 | GDPDNFYS | -1.13 | Non-Toxin | -0.2 | 0 | 913.99 |
| 597 | GDPDNFYT | -1.14 | Non-Toxin | -0.19 | 0 | 928.02 |
| 598 | GDPDNFYC | -0.96 | Non-Toxin | -0.16 | 0 | 930.05 |
| 599 | GDPDNFYN | -1.14 | Non-Toxin | -0.25 | 0 | 941.02 |
| 600 | GDPDNFYQ | -1 | Non-Toxin | -0.26 | 0.16 | 955.05 |
| 601 | GDPDNWFS | -0.83 | Non-Toxin | -0.16 | 0 | 937.03 |
| 602 | GDPDNWFT | -0.76 | Non-Toxin | -0.15 | 0 | 951.06 |
| 603 | GDPDNWFC | -0.43 | Non-Toxin | -0.12 | 0 | 953.09 |
| 604 | GDPDNWFN | -0.77 | Non-Toxin | -0.21 | 0 | 964.06 |
| 605 | GDPDNWFQ | -0.84 | Non-Toxin | -0.21 | 0.16 | 978.09 |
| 606 | GDPDNWWS | -0.76 | Non-Toxin | -0.19 | 0 | 976.07 |
| 607 | GDPDNWWT | -0.75 | Non-Toxin | -0.18 | 0 | 990.1 |
| 608 | GDPDNWWC | -0.2 | Non-Toxin | -0.15 | 0 | 992.13 |
| 609 | GDPDNWWN | -0.82 | Non-Toxin | -0.24 | 0 | 1003.1 |
| 610 | GDPDNWWQ | -0.72 | Non-Toxin | -0.24 | 0.16 | 1017.13 |
| 611 | GDPDNWYS | -0.61 | Non-Toxin | -0.23 | 0 | 953.03 |
| 612 | GDPDNWYT | -0.64 | Non-Toxin | -0.22 | 0 | 967.06 |
| 613 | GDPDNWYC | -0.39 | Non-Toxin | -0.19 | 0 | 969.09 |
| 614 | GDPDNWYN | -0.61 | Non-Toxin | -0.28 | 0 | 980.06 |
| 615 | GDPDNWYQ | -0.45 | Non-Toxin | -0.29 | 0.16 | 994.09 |
| 616 | GDPDNYFS | -0.71 | Non-Toxin | -0.2 | 0 | 913.99 |
| 617 | GDPDNYFT | -0.65 | Non-Toxin | -0.19 | 0 | 928.02 |
| 618 | GDPDNYFC | -0.33 | Non-Toxin | -0.16 | 0 | 930.05 |
| 619 | GDPDNYFN | -0.69 | Non-Toxin | -0.25 | 0 | 941.02 |
| 620 | GDPDNYFQ | -0.75 | Non-Toxin | -0.26 | 0.16 | 955.05 |
| 621 | GDPDNYWS | -0.79 | Non-Toxin | -0.23 | 0 | 953.03 |
| 622 | GDPDNYWT | -0.78 | Non-Toxin | -0.22 | 0 | 967.06 |
| 623 | GDPDNYWC | -0.23 | Non-Toxin | -0.19 | 0 | 969.09 |
| 624 | GDPDNYWN | -0.87 | Non-Toxin | -0.28 | 0 | 980.06 |
| 625 | GDPDNYWQ | -0.75 | Non-Toxin | -0.29 | 0.16 | 994.09 |
| 626 | GDPDNYYS | -0.65 | Non-Toxin | -0.28 | 0 | 929.99 |
| 627 | GDPDNYYT | -0.65 | Non-Toxin | -0.27 | 0 | 944.02 |
| 628 | GDPDNYYC | -0.3 | Non-Toxin | -0.24 | 0 | 946.05 |
| 629 | GDPDNYYN | -0.67 | Non-Toxin | -0.32 | 0 | 957.02 |
| 630 | GDPDNYYQ | -0.48 | Non-Toxin | -0.33 | 0.16 | 971.05 |
| 631 | GDPDQFFS | -0.55 | Non-Toxin | -0.14 | 0.16 | 912.02 |
| 632 | GDPDQFFT | -0.46 | Non-Toxin | -0.13 | 0.16 | 926.05 |
| 633 | GDPDQFFC | -0.12 | Non-Toxin | -0.1 | 0.16 | 928.08 |
| 634 | GDPDQFFN | -0.45 | Non-Toxin | -0.18 | 0.16 | 939.05 |
| 635 | GDPDQFFQ | -0.55 | Non-Toxin | -0.19 | 0.31 | 953.08 |
| 636 | GDPDQFWS | -0.68 | Non-Toxin | -0.17 | 0.16 | 951.06 |
| 637 | GDPDQFWT | -0.64 | Non-Toxin | -0.15 | 0.16 | 965.09 |
| 638 | GDPDQFWC | -0.1 | Non-Toxin | -0.13 | 0.16 | 967.12 |
| 639 | GDPDQFWN | -0.73 | Non-Toxin | -0.21 | 0.16 | 978.09 |
| 640 | GDPDQFWQ | -0.63 | Non-Toxin | -0.22 | 0.31 | 992.12 |
| 641 | GDPDQFYS | -0.72 | Non-Toxin | -0.21 | 0.16 | 928.02 |
| 642 | GDPDQFYT | -0.75 | Non-Toxin | -0.2 | 0.16 | 942.05 |
| 643 | GDPDQFYC | -0.54 | Non-Toxin | -0.17 | 0.16 | 944.08 |
| 644 | GDPDQFYN | -0.77 | Non-Toxin | -0.26 | 0.16 | 955.05 |
| 645 | GDPDQFYQ | -0.57 | Non-Toxin | -0.26 | 0.31 | 969.08 |
| 646 | GDPDQWFS | -0.66 | Non-Toxin | -0.17 | 0.16 | 951.06 |
| 647 | GDPDQWFT | -0.59 | Non-Toxin | -0.15 | 0.16 | 965.09 |
| 648 | GDPDQWFC | -0.26 | Non-Toxin | -0.13 | 0.16 | 967.12 |
| 649 | GDPDQWFN | -0.59 | Non-Toxin | -0.21 | 0.16 | 978.09 |
| 650 | GDPDQWFQ | -0.65 | Non-Toxin | -0.22 | 0.31 | 992.12 |
| 651 | GDPDQWWS | -0.59 | Non-Toxin | -0.19 | 0.16 | 990.1 |
| 652 | GDPDQWWT | -0.57 | Non-Toxin | -0.18 | 0.16 | 1004.13 |
| 653 | GDPDQWWC | -0.02 | Non-Toxin | -0.16 | 0.16 | 1006.16 |
| 654 | GDPDQWWN | -0.65 | Non-Toxin | -0.24 | 0.16 | 1017.13 |
| 655 | GDPDQWWQ | -0.55 | Non-Toxin | -0.25 | 0.31 | 1031.16 |
| 656 | GDPDQWYS | -0.39 | Non-Toxin | -0.24 | 0.16 | 967.06 |
| 657 | GDPDQWYT | -0.44 | Non-Toxin | -0.23 | 0.16 | 981.09 |
| 658 | GDPDQWYC | -0.18 | Non-Toxin | -0.2 | 0.16 | 983.12 |
| 659 | GDPDQWYN | -0.43 | Non-Toxin | -0.29 | 0.16 | 994.09 |
| 660 | GDPDQWYQ | -0.26 | Non-Toxin | -0.29 | 0.31 | 1008.12 |
| 661 | GDPDQYFS | -0.76 | Non-Toxin | -0.21 | 0.16 | 928.02 |
| 662 | GDPDQYFT | -0.66 | Non-Toxin | -0.2 | 0.16 | 942.05 |
| 663 | GDPDQYFC | -0.36 | Non-Toxin | -0.17 | 0.16 | 944.08 |
| 664 | GDPDQYFN | -0.71 | Non-Toxin | -0.26 | 0.16 | 955.05 |
| 665 | GDPDQYFQ | -0.77 | Non-Toxin | -0.26 | 0.31 | 969.08 |
| 666 | GDPDQYWS | -0.8 | Non-Toxin | -0.24 | 0.16 | 967.06 |
| 667 | GDPDQYWT | -0.77 | Non-Toxin | -0.23 | 0.16 | 981.09 |
| 668 | GDPDQYWC | -0.22 | Non-Toxin | -0.2 | 0.16 | 983.12 |
| 669 | GDPDQYWN | -0.86 | Non-Toxin | -0.29 | 0.16 | 994.09 |
| 670 | GDPDQYWQ | -0.75 | Non-Toxin | -0.29 | 0.31 | 1008.12 |
| 671 | GDPDQYYS | -0.63 | Non-Toxin | -0.28 | 0.16 | 944.02 |
| 672 | GDPDQYYT | -0.66 | Non-Toxin | -0.27 | 0.16 | 958.05 |
| 673 | GDPDQYYC | -0.36 | Non-Toxin | -0.24 | 0.16 | 960.08 |
| 674 | GDPDQYYN | -0.67 | Non-Toxin | -0.33 | 0.16 | 971.05 |
| 675 | GDPDQYYQ | -0.5 | Non-Toxin | -0.34 | 0.31 | 985.08 |
| 676 | GDPESFFS | -0.78 | Non-Toxin | -0.07 | 0.16 | 884.99 |
| 677 | GDPESFFT | -0.71 | Non-Toxin | -0.06 | 0.16 | 899.02 |
| 678 | GDPESFFC | -0.36 | Non-Toxin | -0.03 | 0.16 | 901.05 |
| 679 | GDPESFFN | -0.75 | Non-Toxin | -0.12 | 0.16 | 912.02 |
| 680 | GDPESFFQ | -0.72 | Non-Toxin | -0.12 | 0.32 | 926.05 |
| 681 | GDPESFWS | -0.97 | Non-Toxin | -0.1 | 0.16 | 924.03 |
| 682 | GDPESFWT | -0.87 | Non-Toxin | -0.09 | 0.16 | 938.06 |
| 683 | GDPESFWC | -0.33 | Non-Toxin | -0.06 | 0.16 | 940.09 |
| 684 | GDPESFWN | -0.97 | Non-Toxin | -0.15 | 0.16 | 951.06 |
| 685 | GDPESFWQ | -0.87 | Non-Toxin | -0.15 | 0.32 | 965.09 |
| 686 | GDPESFYS | -1.05 | Non-Toxin | -0.14 | 0.16 | 900.99 |
| 687 | GDPESFYT | -1.04 | Non-Toxin | -0.13 | 0.16 | 915.02 |
| 688 | GDPESFYC | -0.83 | Non-Toxin | -0.1 | 0.16 | 917.05 |
| 689 | GDPESFYN | -1.03 | Non-Toxin | -0.19 | 0.16 | 928.02 |
| 690 | GDPESFYQ | -0.86 | Non-Toxin | -0.2 | 0.32 | 942.05 |
| 691 | GDPESWFS | -1.07 | Non-Toxin | -0.1 | 0.16 | 924.03 |
| 692 | GDPESWFT | -1.01 | Non-Toxin | -0.09 | 0.16 | 938.06 |
| 693 | GDPESWFC | -0.65 | Non-Toxin | -0.06 | 0.16 | 940.09 |
| 694 | GDPESWFN | -1.01 | Non-Toxin | -0.15 | 0.16 | 951.06 |
| 695 | GDPESWFQ | -1.02 | Non-Toxin | -0.15 | 0.32 | 965.09 |
| 696 | GDPESWWS | -1.01 | Non-Toxin | -0.13 | 0.16 | 963.07 |
| 697 | GDPESWWT | -0.97 | Non-Toxin | -0.12 | 0.16 | 977.1 |
| 698 | GDPESWWC | -0.44 | Non-Toxin | -0.09 | 0.16 | 979.13 |
| 699 | GDPESWWN | -1.05 | Non-Toxin | -0.18 | 0.16 | 990.1 |
| 700 | GDPESWWQ | -0.95 | Non-Toxin | -0.18 | 0.32 | 1004.13 |
| 701 | GDPESWYS | -0.83 | Non-Toxin | -0.17 | 0.16 | 940.03 |
| 702 | GDPESWYT | -0.85 | Non-Toxin | -0.16 | 0.16 | 954.06 |
| 703 | GDPESWYC | -0.56 | Non-Toxin | -0.14 | 0.16 | 956.09 |
| 704 | GDPESWYN | -0.82 | Non-Toxin | -0.22 | 0.16 | 967.06 |
| 705 | GDPESWYQ | -0.63 | Non-Toxin | -0.23 | 0.32 | 981.09 |
| 706 | GDPESYFS | -0.92 | Non-Toxin | -0.14 | 0.16 | 900.99 |
| 707 | GDPESYFT | -0.84 | Non-Toxin | -0.13 | 0.16 | 915.02 |
| 708 | GDPESYFC | -0.5 | Non-Toxin | -0.1 | 0.16 | 917.05 |
| 709 | GDPESYFN | -0.9 | Non-Toxin | -0.19 | 0.16 | 928.02 |
| 710 | GDPESYFQ | -0.89 | Non-Toxin | -0.2 | 0.32 | 942.05 |
| 711 | GDPESYWS | -1 | Non-Toxin | -0.17 | 0.16 | 940.03 |
| 712 | GDPESYWT | -0.97 | Non-Toxin | -0.16 | 0.16 | 954.06 |
| 713 | GDPESYWC | -0.43 | Non-Toxin | -0.14 | 0.16 | 956.09 |
| 714 | GDPESYWN | -1.05 | Non-Toxin | -0.22 | 0.16 | 967.06 |
| 715 | GDPESYWQ | -0.94 | Non-Toxin | -0.23 | 0.32 | 981.09 |
| 716 | GDPESYYS | -0.81 | Non-Toxin | -0.22 | 0.16 | 916.99 |
| 717 | GDPESYYT | -0.83 | Non-Toxin | -0.21 | 0.16 | 931.02 |
| 718 | GDPESYYC | -0.47 | Non-Toxin | -0.18 | 0.16 | 933.05 |
| 719 | GDPESYYN | -0.81 | Non-Toxin | -0.26 | 0.16 | 944.02 |
| 720 | GDPESYYQ | -0.59 | Non-Toxin | -0.27 | 0.32 | 958.05 |
| 721 | GDPETFFS | -0.93 | Non-Toxin | -0.06 | 0.16 | 899.02 |
| 722 | GDPETFFT | -0.9 | Non-Toxin | -0.05 | 0.16 | 913.05 |
| 723 | GDPETFFC | -0.51 | Non-Toxin | -0.02 | 0.16 | 915.08 |
| 724 | GDPETFFN | -0.84 | Non-Toxin | -0.11 | 0.16 | 926.05 |
| 725 | GDPETFFQ | -0.91 | Non-Toxin | -0.11 | 0.32 | 940.08 |
| 726 | GDPETFWS | -1.06 | Non-Toxin | -0.09 | 0.16 | 938.06 |
| 727 | GDPETFWT | -0.99 | Non-Toxin | -0.08 | 0.16 | 952.09 |
| 728 | GDPETFWC | -0.47 | Non-Toxin | -0.05 | 0.16 | 954.12 |
| 729 | GDPETFWN | -1.1 | Non-Toxin | -0.14 | 0.16 | 965.09 |
| 730 | GDPETFWQ | -1 | Non-Toxin | -0.14 | 0.32 | 979.12 |
| 731 | GDPETFYS | -1.14 | Non-Toxin | -0.13 | 0.16 | 915.02 |
| 732 | GDPETFYT | -1.14 | Non-Toxin | -0.12 | 0.16 | 929.05 |
| 733 | GDPETFYC | -0.93 | Non-Toxin | -0.09 | 0.16 | 931.08 |
| 734 | GDPETFYN | -1.14 | Non-Toxin | -0.18 | 0.16 | 942.05 |
| 735 | GDPETFYQ | -0.97 | Non-Toxin | -0.19 | 0.32 | 956.08 |
| 736 | GDPETWFS | -1.03 | Non-Toxin | -0.09 | 0.16 | 938.06 |
| 737 | GDPETWFT | -1 | Non-Toxin | -0.08 | 0.16 | 952.09 |
| 738 | GDPETWFC | -0.63 | Non-Toxin | -0.05 | 0.16 | 954.12 |
| 739 | GDPETWFN | -0.97 | Non-Toxin | -0.14 | 0.16 | 965.09 |
| 740 | GDPETWFQ | -1.01 | Non-Toxin | -0.14 | 0.32 | 979.12 |
| 741 | GDPETWWS | -0.98 | Non-Toxin | -0.12 | 0.16 | 977.1 |
| 742 | GDPETWWT | -0.95 | Non-Toxin | -0.11 | 0.16 | 991.13 |
| 743 | GDPETWWC | -0.38 | Non-Toxin | -0.08 | 0.16 | 993.16 |
| 744 | GDPETWWN | -1.03 | Non-Toxin | -0.17 | 0.16 | 1004.13 |
| 745 | GDPETWWQ | -0.92 | Non-Toxin | -0.17 | 0.32 | 1018.16 |
| 746 | GDPETWYS | -0.84 | Non-Toxin | -0.16 | 0.16 | 954.06 |
| 747 | GDPETWYT | -0.82 | Non-Toxin | -0.15 | 0.16 | 968.09 |
| 748 | GDPETWYC | -0.56 | Non-Toxin | -0.12 | 0.16 | 970.12 |
| 749 | GDPETWYN | -0.82 | Non-Toxin | -0.21 | 0.16 | 981.09 |
| 750 | GDPETWYQ | -0.63 | Non-Toxin | -0.22 | 0.32 | 995.12 |
| 751 | GDPETYFS | -0.87 | Non-Toxin | -0.13 | 0.16 | 915.02 |
| 752 | GDPETYFT | -0.82 | Non-Toxin | -0.12 | 0.16 | 929.05 |
| 753 | GDPETYFC | -0.49 | Non-Toxin | -0.09 | 0.16 | 931.08 |
| 754 | GDPETYFN | -0.86 | Non-Toxin | -0.18 | 0.16 | 942.05 |
| 755 | GDPETYFQ | -0.87 | Non-Toxin | -0.19 | 0.32 | 956.08 |
| 756 | GDPETYWS | -0.96 | Non-Toxin | -0.16 | 0.16 | 954.06 |
| 757 | GDPETYWT | -0.94 | Non-Toxin | -0.15 | 0.16 | 968.09 |
| 758 | GDPETYWC | -0.38 | Non-Toxin | -0.12 | 0.16 | 970.12 |
| 759 | GDPETYWN | -1.02 | Non-Toxin | -0.21 | 0.16 | 981.09 |
| 760 | GDPETYWQ | -0.91 | Non-Toxin | -0.22 | 0.32 | 995.12 |
| 761 | GDPETYYS | -0.84 | Non-Toxin | -0.21 | 0.16 | 931.02 |
| 762 | GDPETYYT | -0.79 | Non-Toxin | -0.2 | 0.16 | 945.05 |
| 763 | GDPETYYC | -0.51 | Non-Toxin | -0.17 | 0.16 | 947.08 |
| 764 | GDPETYYN | -0.82 | Non-Toxin | -0.25 | 0.16 | 958.05 |
| 765 | GDPETYYQ | -0.62 | Non-Toxin | -0.26 | 0.32 | 972.08 |
| 766 | GDPECFFS | -0.35 | Non-Toxin | -0.03 | 0.16 | 901.05 |
| 767 | GDPECFFT | -0.25 | Non-Toxin | -0.02 | 0.16 | 915.08 |
| 768 | GDPECFFC | 0.15 | Toxin | 0.01 | 0.16 | 917.11 |
| 769 | GDPECFFN | -0.27 | Non-Toxin | -0.08 | 0.16 | 928.08 |
| 770 | GDPECFFQ | -0.32 | Non-Toxin | -0.08 | 0.32 | 942.11 |
| 771 | GDPECFWS | -0.6 | Non-Toxin | -0.06 | 0.16 | 940.09 |
| 772 | GDPECFWT | -0.58 | Non-Toxin | -0.05 | 0.16 | 954.12 |
| 773 | GDPECFWC | -0.03 | Non-Toxin | -0.02 | 0.16 | 956.15 |
| 774 | GDPECFWN | -0.65 | Non-Toxin | -0.11 | 0.16 | 967.12 |
| 775 | GDPECFWQ | -0.54 | Non-Toxin | -0.11 | 0.32 | 981.15 |
| 776 | GDPECFYS | -0.59 | Non-Toxin | -0.1 | 0.16 | 917.05 |
| 777 | GDPECFYT | -0.58 | Non-Toxin | -0.1 | 0.16 | 931.08 |
| 778 | GDPECFYC | -0.46 | Non-Toxin | -0.07 | 0.16 | 933.11 |
| 779 | GDPECFYN | -0.58 | Non-Toxin | -0.15 | 0.16 | 944.08 |
| 780 | GDPECFYQ | -0.42 | Non-Toxin | -0.16 | 0.32 | 958.11 |
| 781 | GDPECWFS | -0.38 | Non-Toxin | -0.06 | 0.16 | 940.09 |
| 782 | GDPECWFT | -0.27 | Non-Toxin | -0.05 | 0.16 | 954.12 |
| 783 | GDPECWFC | 0.09 | Toxin | -0.02 | 0.16 | 956.15 |
| 784 | GDPECWFN | -0.27 | Non-Toxin | -0.11 | 0.16 | 967.12 |
| 785 | GDPECWFQ | -0.33 | Non-Toxin | -0.11 | 0.32 | 981.15 |
| 786 | GDPECWWS | -0.27 | Non-Toxin | -0.09 | 0.16 | 979.13 |
| 787 | GDPECWWT | -0.26 | Non-Toxin | -0.08 | 0.16 | 993.16 |
| 788 | GDPECWWC | 0.31 | Toxin | -0.05 | 0.16 | 995.19 |
| 789 | GDPECWWN | -0.32 | Non-Toxin | -0.14 | 0.16 | 1006.16 |
| 790 | GDPECWWQ | -0.22 | Non-Toxin | -0.14 | 0.32 | 1020.19 |
| 791 | GDPECWYS | -0.13 | Non-Toxin | -0.14 | 0.16 | 956.09 |
| 792 | GDPECWYT | -0.09 | Non-Toxin | -0.12 | 0.16 | 970.12 |
| 793 | GDPECWYC | 0.13 | Toxin | -0.1 | 0.16 | 972.15 |
| 794 | GDPECWYN | -0.11 | Non-Toxin | -0.18 | 0.16 | 983.12 |
| 795 | GDPECWYQ | 0.08 | Toxin | -0.19 | 0.32 | 997.15 |
| 796 | GDPECYFS | -0.27 | Non-Toxin | -0.1 | 0.16 | 917.05 |
| 797 | GDPECYFT | -0.15 | Non-Toxin | -0.1 | 0.16 | 931.08 |
| 798 | GDPECYFC | 0.16 | Toxin | -0.07 | 0.16 | 933.11 |
| 799 | GDPECYFN | -0.19 | Non-Toxin | -0.15 | 0.16 | 944.08 |
| 800 | GDPECYFQ | -0.24 | Non-Toxin | -0.16 | 0.32 | 958.11 |
| 801 | GDPECYWS | -0.24 | Non-Toxin | -0.14 | 0.16 | 956.09 |
| 802 | GDPECYWT | -0.23 | Non-Toxin | -0.12 | 0.16 | 970.12 |
| 803 | GDPECYWC | 0.29 | Toxin | -0.1 | 0.16 | 972.15 |
| 804 | GDPECYWN | -0.33 | Non-Toxin | -0.18 | 0.16 | 983.12 |
| 805 | GDPECYWQ | -0.2 | Non-Toxin | -0.19 | 0.32 | 997.15 |
| 806 | GDPECYYS | -0.09 | Non-Toxin | -0.18 | 0.16 | 933.05 |
| 807 | GDPECYYT | -0.11 | Non-Toxin | -0.17 | 0.16 | 947.08 |
| 808 | GDPECYYC | 0.17 | Toxin | -0.14 | 0.16 | 949.11 |
| 809 | GDPECYYN | -0.07 | Non-Toxin | -0.23 | 0.16 | 960.08 |
| 810 | GDPECYYQ | 0.08 | Toxin | -0.23 | 0.32 | 974.11 |
| 811 | GDPENFFS | -1.05 | Non-Toxin | -0.12 | 0.16 | 912.02 |
| 812 | GDPENFFT | -0.94 | Non-Toxin | -0.11 | 0.16 | 926.05 |
| 813 | GDPENFFC | -0.58 | Non-Toxin | -0.08 | 0.16 | 928.08 |
| 814 | GDPENFFN | -0.94 | Non-Toxin | -0.16 | 0.16 | 939.05 |
| 815 | GDPENFFQ | -0.98 | Non-Toxin | -0.17 | 0.32 | 953.08 |
| 816 | GDPENFWS | -1.16 | Non-Toxin | -0.15 | 0.16 | 951.06 |
| 817 | GDPENFWT | -1.12 | Non-Toxin | -0.14 | 0.16 | 965.09 |
| 818 | GDPENFWC | -0.6 | Non-Toxin | -0.11 | 0.16 | 967.12 |
| 819 | GDPENFWN | -1.2 | Non-Toxin | -0.19 | 0.16 | 978.09 |
| 820 | GDPENFWQ | -1.11 | Non-Toxin | -0.2 | 0.32 | 992.12 |
| 821 | GDPENFYS | -1.21 | Non-Toxin | -0.19 | 0.16 | 928.02 |
| 822 | GDPENFYT | -1.19 | Non-Toxin | -0.18 | 0.16 | 942.05 |
| 823 | GDPENFYC | -0.99 | Non-Toxin | -0.15 | 0.16 | 944.08 |
| 824 | GDPENFYN | -1.22 | Non-Toxin | -0.24 | 0.16 | 955.05 |
| 825 | GDPENFYQ | -1.06 | Non-Toxin | -0.24 | 0.32 | 969.08 |
| 826 | GDPENWFS | -0.96 | Non-Toxin | -0.15 | 0.16 | 951.06 |
| 827 | GDPENWFT | -0.87 | Non-Toxin | -0.14 | 0.16 | 965.09 |
| 828 | GDPENWFC | -0.52 | Non-Toxin | -0.11 | 0.16 | 967.12 |
| 829 | GDPENWFN | -0.87 | Non-Toxin | -0.19 | 0.16 | 978.09 |
| 830 | GDPENWFQ | -0.9 | Non-Toxin | -0.2 | 0.32 | 992.12 |
| 831 | GDPENWWS | -0.87 | Non-Toxin | -0.18 | 0.16 | 990.1 |
| 832 | GDPENWWT | -0.85 | Non-Toxin | -0.17 | 0.16 | 1004.13 |
| 833 | GDPENWWC | -0.32 | Non-Toxin | -0.14 | 0.16 | 1006.16 |
| 834 | GDPENWWN | -0.93 | Non-Toxin | -0.22 | 0.16 | 1017.13 |
| 835 | GDPENWWQ | -0.83 | Non-Toxin | -0.23 | 0.32 | 1031.16 |
| 836 | GDPENWYS | -0.71 | Non-Toxin | -0.22 | 0.16 | 967.06 |
| 837 | GDPENWYT | -0.72 | Non-Toxin | -0.21 | 0.16 | 981.09 |
| 838 | GDPENWYC | -0.44 | Non-Toxin | -0.18 | 0.16 | 983.12 |
| 839 | GDPENWYN | -0.71 | Non-Toxin | -0.27 | 0.16 | 994.09 |
| 840 | GDPENWYQ | -0.53 | Non-Toxin | -0.27 | 0.32 | 1008.12 |
| 841 | GDPENYFS | -0.82 | Non-Toxin | -0.19 | 0.16 | 928.02 |
| 842 | GDPENYFT | -0.74 | Non-Toxin | -0.18 | 0.16 | 942.05 |
| 843 | GDPENYFC | -0.4 | Non-Toxin | -0.15 | 0.16 | 944.08 |
| 844 | GDPENYFN | -0.77 | Non-Toxin | -0.24 | 0.16 | 955.05 |
| 845 | GDPENYFQ | -0.78 | Non-Toxin | -0.24 | 0.32 | 969.08 |
| 846 | GDPENYWS | -0.9 | Non-Toxin | -0.22 | 0.16 | 967.06 |
| 847 | GDPENYWT | -0.87 | Non-Toxin | -0.21 | 0.16 | 981.09 |
| 848 | GDPENYWC | -0.34 | Non-Toxin | -0.18 | 0.16 | 983.12 |
| 849 | GDPENYWN | -0.96 | Non-Toxin | -0.27 | 0.16 | 994.09 |
| 850 | GDPENYWQ | -0.85 | Non-Toxin | -0.27 | 0.32 | 1008.12 |
| 851 | GDPENYYS | -0.72 | Non-Toxin | -0.26 | 0.16 | 944.02 |
| 852 | GDPENYYT | -0.69 | Non-Toxin | -0.25 | 0.16 | 958.05 |
| 853 | GDPENYYC | -0.33 | Non-Toxin | -0.23 | 0.16 | 960.08 |
| 854 | GDPENYYN | -0.74 | Non-Toxin | -0.31 | 0.16 | 971.05 |
| 855 | GDPENYYQ | -0.54 | Non-Toxin | -0.32 | 0.32 | 985.08 |
| 856 | GDPEQFFS | -0.83 | Non-Toxin | -0.12 | 0.32 | 926.05 |
| 857 | GDPEQFFT | -0.72 | Non-Toxin | -0.11 | 0.32 | 940.08 |
| 858 | GDPEQFFC | -0.36 | Non-Toxin | -0.09 | 0.32 | 942.11 |
| 859 | GDPEQFFN | -0.72 | Non-Toxin | -0.17 | 0.32 | 953.08 |
| 860 | GDPEQFFQ | -0.78 | Non-Toxin | -0.18 | 0.47 | 967.11 |
| 861 | GDPEQFWS | -0.96 | Non-Toxin | -0.15 | 0.32 | 965.09 |
| 862 | GDPEQFWT | -0.9 | Non-Toxin | -0.14 | 0.32 | 979.12 |
| 863 | GDPEQFWC | -0.38 | Non-Toxin | -0.12 | 0.32 | 981.15 |
| 864 | GDPEQFWN | -0.99 | Non-Toxin | -0.2 | 0.32 | 992.12 |
| 865 | GDPEQFWQ | -0.9 | Non-Toxin | -0.21 | 0.47 | 1006.15 |
| 866 | GDPEQFYS | -1.03 | Non-Toxin | -0.2 | 0.32 | 942.05 |
| 867 | GDPEQFYT | -1 | Non-Toxin | -0.19 | 0.32 | 956.08 |
| 868 | GDPEQFYC | -0.79 | Non-Toxin | -0.16 | 0.32 | 958.11 |
| 869 | GDPEQFYN | -1.02 | Non-Toxin | -0.24 | 0.32 | 969.08 |
| 870 | GDPEQFYQ | -0.84 | Non-Toxin | -0.25 | 0.47 | 983.11 |
| 871 | GDPEQWFS | -0.97 | Non-Toxin | -0.15 | 0.32 | 965.09 |
| 872 | GDPEQWFT | -0.88 | Non-Toxin | -0.14 | 0.32 | 979.12 |
| 873 | GDPEQWFC | -0.53 | Non-Toxin | -0.11 | 0.32 | 981.15 |
| 874 | GDPEQWFN | -0.88 | Non-Toxin | -0.2 | 0.32 | 992.12 |
| 875 | GDPEQWFQ | -0.91 | Non-Toxin | -0.21 | 0.47 | 1006.15 |
| 876 | GDPEQWWS | -0.91 | Non-Toxin | -0.18 | 0.32 | 1004.13 |
| 877 | GDPEQWWT | -0.86 | Non-Toxin | -0.17 | 0.32 | 1018.16 |
| 878 | GDPEQWWC | -0.33 | Non-Toxin | -0.14 | 0.32 | 1020.19 |
| 879 | GDPEQWWN | -0.95 | Non-Toxin | -0.23 | 0.32 | 1031.16 |
| 880 | GDPEQWWQ | -0.84 | Non-Toxin | -0.24 | 0.47 | 1045.19 |
| 881 | GDPEQWYS | -0.73 | Non-Toxin | -0.23 | 0.32 | 981.09 |
| 882 | GDPEQWYT | -0.72 | Non-Toxin | -0.22 | 0.32 | 995.12 |
| 883 | GDPEQWYC | -0.45 | Non-Toxin | -0.19 | 0.32 | 997.15 |
| 884 | GDPEQWYN | -0.71 | Non-Toxin | -0.27 | 0.32 | 1008.12 |
| 885 | GDPEQWYQ | -0.55 | Non-Toxin | -0.28 | 0.47 | 1022.15 |
| 886 | GDPEQYFS | -1.05 | Non-Toxin | -0.2 | 0.32 | 942.05 |
| 887 | GDPEQYFT | -0.93 | Non-Toxin | -0.19 | 0.32 | 956.08 |
| 888 | GDPEQYFC | -0.62 | Non-Toxin | -0.16 | 0.32 | 958.11 |
| 889 | GDPEQYFN | -0.98 | Non-Toxin | -0.24 | 0.32 | 969.08 |
| 890 | GDPEQYFQ | -1.02 | Non-Toxin | -0.25 | 0.47 | 983.11 |
| 891 | GDPEQYWS | -1.11 | Non-Toxin | -0.23 | 0.32 | 981.09 |
| 892 | GDPEQYWT | -1.06 | Non-Toxin | -0.22 | 0.32 | 995.12 |
| 893 | GDPEQYWC | -0.53 | Non-Toxin | -0.19 | 0.32 | 997.15 |
| 894 | GDPEQYWN | -1.15 | Non-Toxin | -0.27 | 0.32 | 1008.12 |
| 895 | GDPEQYWQ | -1.04 | Non-Toxin | -0.28 | 0.47 | 1022.15 |
| 896 | GDPEQYYS | -0.96 | Non-Toxin | -0.27 | 0.32 | 958.05 |
| 897 | GDPEQYYT | -0.93 | Non-Toxin | -0.26 | 0.32 | 972.08 |
| 898 | GDPEQYYC | -0.63 | Non-Toxin | -0.23 | 0.32 | 974.11 |
| 899 | GDPEQYYN | -0.93 | Non-Toxin | -0.32 | 0.32 | 985.08 |
| 900 | GDPEQYYQ | -0.78 | Non-Toxin | -0.32 | 0.47 | 999.11 |
| 901 | GEGDSFFS | -0.97 | Non-Toxin | -0.04 | 0.16 | 844.93 |
| 902 | GEGDSFFT | -0.93 | Non-Toxin | -0.03 | 0.16 | 858.96 |
| 903 | GEGDSFFC | -0.69 | Non-Toxin | 0 | 0.16 | 860.99 |
| 904 | GEGDSFFN | -0.92 | Non-Toxin | -0.09 | 0.16 | 871.96 |
| 905 | GEGDSFFQ | -0.97 | Non-Toxin | -0.09 | 0.32 | 885.99 |
| 906 | GEGDSFWS | -1.15 | Non-Toxin | -0.07 | 0.16 | 883.97 |
| 907 | GEGDSFWT | -1.05 | Non-Toxin | -0.06 | 0.16 | 898 |
| 908 | GEGDSFWC | -0.51 | Non-Toxin | -0.03 | 0.16 | 900.03 |
| 909 | GEGDSFWN | -1.15 | Non-Toxin | -0.12 | 0.16 | 911 |
| 910 | GEGDSFWQ | -1.06 | Non-Toxin | -0.12 | 0.32 | 925.03 |
| 911 | GEGDSFYS | -1.29 | Non-Toxin | -0.11 | 0.16 | 860.93 |
| 912 | GEGDSFYT | -1.24 | Non-Toxin | -0.1 | 0.16 | 874.96 |
| 913 | GEGDSFYC | -1.1 | Non-Toxin | -0.08 | 0.16 | 876.99 |
| 914 | GEGDSFYN | -1.24 | Non-Toxin | -0.16 | 0.16 | 887.96 |
| 915 | GEGDSFYQ | -1.01 | Non-Toxin | -0.17 | 0.32 | 901.99 |
| 916 | GEGDSWFS | -1.25 | Non-Toxin | -0.07 | 0.16 | 883.97 |
| 917 | GEGDSWFT | -1.2 | Non-Toxin | -0.06 | 0.16 | 898 |
| 918 | GEGDSWFC | -0.96 | Non-Toxin | -0.03 | 0.16 | 900.03 |
| 919 | GEGDSWFN | -1.16 | Non-Toxin | -0.12 | 0.16 | 911 |
| 920 | GEGDSWFQ | -1.25 | Non-Toxin | -0.12 | 0.32 | 925.03 |
| 921 | GEGDSWWS | -1.19 | Non-Toxin | -0.1 | 0.16 | 923.01 |
| 922 | GEGDSWWT | -1.14 | Non-Toxin | -0.09 | 0.16 | 937.04 |
| 923 | GEGDSWWC | -0.62 | Non-Toxin | -0.06 | 0.16 | 939.07 |
| 924 | GEGDSWWN | -1.23 | Non-Toxin | -0.15 | 0.16 | 950.04 |
| 925 | GEGDSWWQ | -1.13 | Non-Toxin | -0.15 | 0.32 | 964.07 |
| 926 | GEGDSWYS | -1.04 | Non-Toxin | -0.14 | 0.16 | 899.97 |
| 927 | GEGDSWYT | -1.03 | Non-Toxin | -0.13 | 0.16 | 914 |
| 928 | GEGDSWYC | -0.8 | Non-Toxin | -0.11 | 0.16 | 916.03 |
| 929 | GEGDSWYN | -1 | Non-Toxin | -0.19 | 0.16 | 927 |
| 930 | GEGDSWYQ | -0.76 | Non-Toxin | -0.2 | 0.32 | 941.03 |
| 931 | GEGDSYFS | -1.1 | Non-Toxin | -0.11 | 0.16 | 860.93 |
| 932 | GEGDSYFT | -1.05 | Non-Toxin | -0.1 | 0.16 | 874.96 |
| 933 | GEGDSYFC | -0.82 | Non-Toxin | -0.08 | 0.16 | 876.99 |
| 934 | GEGDSYFN | -1.06 | Non-Toxin | -0.16 | 0.16 | 887.96 |
| 935 | GEGDSYFQ | -1.13 | Non-Toxin | -0.17 | 0.32 | 901.99 |
| 936 | GEGDSYWS | -1.17 | Non-Toxin | -0.14 | 0.16 | 899.97 |
| 937 | GEGDSYWT | -1.13 | Non-Toxin | -0.13 | 0.16 | 914 |
| 938 | GEGDSYWC | -0.59 | Non-Toxin | -0.11 | 0.16 | 916.03 |
| 939 | GEGDSYWN | -1.22 | Non-Toxin | -0.19 | 0.16 | 927 |
| 940 | GEGDSYWQ | -1.11 | Non-Toxin | -0.2 | 0.32 | 941.03 |
| 941 | GEGDSYYS | -1.02 | Non-Toxin | -0.19 | 0.16 | 876.93 |
| 942 | GEGDSYYT | -1 | Non-Toxin | -0.18 | 0.16 | 890.96 |
| 943 | GEGDSYYC | -0.7 | Non-Toxin | -0.15 | 0.16 | 892.99 |
| 944 | GEGDSYYN | -0.98 | Non-Toxin | -0.23 | 0.16 | 903.96 |
| 945 | GEGDSYYQ | -0.72 | Non-Toxin | -0.24 | 0.32 | 917.99 |
| 946 | GEGDTFFS | -0.91 | Non-Toxin | -0.03 | 0.16 | 858.96 |
| 947 | GEGDTFFT | -0.84 | Non-Toxin | -0.02 | 0.16 | 872.99 |
| 948 | GEGDTFFC | -0.54 | Non-Toxin | 0.01 | 0.16 | 875.02 |
| 949 | GEGDTFFN | -0.78 | Non-Toxin | -0.08 | 0.16 | 885.99 |
| 950 | GEGDTFFQ | -0.91 | Non-Toxin | -0.08 | 0.32 | 900.02 |
| 951 | GEGDTFWS | -1.02 | Non-Toxin | -0.06 | 0.16 | 898 |
| 952 | GEGDTFWT | -0.96 | Non-Toxin | -0.05 | 0.16 | 912.03 |
| 953 | GEGDTFWC | -0.46 | Non-Toxin | -0.02 | 0.16 | 914.06 |
| 954 | GEGDTFWN | -1.07 | Non-Toxin | -0.11 | 0.16 | 925.03 |
| 955 | GEGDTFWQ | -0.97 | Non-Toxin | -0.11 | 0.32 | 939.06 |
| 956 | GEGDTFYS | -1.13 | Non-Toxin | -0.1 | 0.16 | 874.96 |
| 957 | GEGDTFYT | -1.12 | Non-Toxin | -0.09 | 0.16 | 888.99 |
| 958 | GEGDTFYC | -0.9 | Non-Toxin | -0.07 | 0.16 | 891.02 |
| 959 | GEGDTFYN | -1.12 | Non-Toxin | -0.15 | 0.16 | 901.99 |
| 960 | GEGDTFYQ | -0.89 | Non-Toxin | -0.16 | 0.32 | 916.02 |
| 961 | GEGDTWFS | -0.94 | Non-Toxin | -0.06 | 0.16 | 898 |
| 962 | GEGDTWFT | -0.87 | Non-Toxin | -0.05 | 0.16 | 912.03 |
| 963 | GEGDTWFC | -0.59 | Non-Toxin | -0.02 | 0.16 | 914.06 |
| 964 | GEGDTWFN | -0.85 | Non-Toxin | -0.11 | 0.16 | 925.03 |
| 965 | GEGDTWFQ | -0.94 | Non-Toxin | -0.11 | 0.32 | 939.06 |
| 966 | GEGDTWWS | -0.89 | Non-Toxin | -0.09 | 0.16 | 937.04 |
| 967 | GEGDTWWT | -0.86 | Non-Toxin | -0.08 | 0.16 | 951.07 |
| 968 | GEGDTWWC | -0.31 | Non-Toxin | -0.05 | 0.16 | 953.1 |
| 969 | GEGDTWWN | -0.94 | Non-Toxin | -0.14 | 0.16 | 964.07 |
| 970 | GEGDTWWQ | -0.84 | Non-Toxin | -0.14 | 0.32 | 978.1 |
| 971 | GEGDTWYS | -0.78 | Non-Toxin | -0.13 | 0.16 | 914 |
| 972 | GEGDTWYT | -0.76 | Non-Toxin | -0.12 | 0.16 | 928.03 |
| 973 | GEGDTWYC | -0.48 | Non-Toxin | -0.1 | 0.16 | 930.06 |
| 974 | GEGDTWYN | -0.76 | Non-Toxin | -0.18 | 0.16 | 941.03 |
| 975 | GEGDTWYQ | -0.5 | Non-Toxin | -0.19 | 0.32 | 955.06 |
| 976 | GEGDTYFS | -0.83 | Non-Toxin | -0.1 | 0.16 | 874.96 |
| 977 | GEGDTYFT | -0.74 | Non-Toxin | -0.09 | 0.16 | 888.99 |
| 978 | GEGDTYFC | -0.51 | Non-Toxin | -0.07 | 0.16 | 891.02 |
| 979 | GEGDTYFN | -0.79 | Non-Toxin | -0.15 | 0.16 | 901.99 |
| 980 | GEGDTYFQ | -0.86 | Non-Toxin | -0.16 | 0.32 | 916.02 |
| 981 | GEGDTYWS | -0.89 | Non-Toxin | -0.13 | 0.16 | 914 |
| 982 | GEGDTYWT | -0.88 | Non-Toxin | -0.12 | 0.16 | 928.03 |
| 983 | GEGDTYWC | -0.34 | Non-Toxin | -0.1 | 0.16 | 930.06 |
| 984 | GEGDTYWN | -0.96 | Non-Toxin | -0.18 | 0.16 | 941.03 |
| 985 | GEGDTYWQ | -0.85 | Non-Toxin | -0.19 | 0.32 | 955.06 |
| 986 | GEGDTYYS | -0.82 | Non-Toxin | -0.18 | 0.16 | 890.96 |
| 987 | GEGDTYYT | -0.77 | Non-Toxin | -0.17 | 0.16 | 904.99 |
| 988 | GEGDTYYC | -0.47 | Non-Toxin | -0.14 | 0.16 | 907.02 |
| 989 | GEGDTYYN | -0.79 | Non-Toxin | -0.22 | 0.16 | 917.99 |
| 990 | GEGDTYYQ | -0.53 | Non-Toxin | -0.23 | 0.32 | 932.02 |
| 991 | GEGDCFFS | -0.51 | Non-Toxin | 0 | 0.16 | 860.99 |
| 992 | GEGDCFFT | -0.45 | Non-Toxin | 0.01 | 0.16 | 875.02 |
| 993 | GEGDCFFC | -0.11 | Non-Toxin | 0.03 | 0.16 | 877.05 |
| 994 | GEGDCFFN | -0.42 | Non-Toxin | -0.05 | 0.16 | 888.02 |
| 995 | GEGDCFFQ | -0.54 | Non-Toxin | -0.06 | 0.32 | 902.05 |
| 996 | GEGDCFWS | -0.77 | Non-Toxin | -0.03 | 0.16 | 900.03 |
| 997 | GEGDCFWT | -0.74 | Non-Toxin | -0.02 | 0.16 | 914.06 |
| 998 | GEGDCFWC | -0.21 | Non-Toxin | 0.01 | 0.16 | 916.09 |
| 999 | GEGDCFWN | -0.83 | Non-Toxin | -0.08 | 0.16 | 927.06 |
| 1000 | GEGDCFWQ | -0.73 | Non-Toxin | -0.09 | 0.32 | 941.09 |
| 1001 | GEGDCFYS | -0.83 | Non-Toxin | -0.08 | 0.16 | 876.99 |
| 1002 | GEGDCFYT | -0.83 | Non-Toxin | -0.07 | 0.16 | 891.02 |
| 1003 | GEGDCFYC | -0.69 | Non-Toxin | -0.04 | 0.16 | 893.05 |
| 1004 | GEGDCFYN | -0.82 | Non-Toxin | -0.12 | 0.16 | 904.02 |
| 1005 | GEGDCFYQ | -0.58 | Non-Toxin | -0.13 | 0.32 | 918.05 |
| 1006 | GEGDCWFS | -0.45 | Non-Toxin | -0.03 | 0.16 | 900.03 |
| 1007 | GEGDCWFT | -0.39 | Non-Toxin | -0.02 | 0.16 | 914.06 |
| 1008 | GEGDCWFC | -0.09 | Non-Toxin | 0.01 | 0.16 | 916.09 |
| 1009 | GEGDCWFN | -0.34 | Non-Toxin | -0.08 | 0.16 | 927.06 |
| 1010 | GEGDCWFQ | -0.46 | Non-Toxin | -0.09 | 0.32 | 941.09 |
| 1011 | GEGDCWWS | -0.39 | Non-Toxin | -0.06 | 0.16 | 939.07 |
| 1012 | GEGDCWWT | -0.39 | Non-Toxin | -0.05 | 0.16 | 953.1 |
| 1013 | GEGDCWWC | 0.16 | Toxin | -0.02 | 0.16 | 955.13 |
| 1014 | GEGDCWWN | -0.46 | Non-Toxin | -0.11 | 0.16 | 966.1 |
| 1015 | GEGDCWWQ | -0.37 | Non-Toxin | -0.12 | 0.32 | 980.13 |
| 1016 | GEGDCWYS | -0.3 | Non-Toxin | -0.11 | 0.16 | 916.03 |
| 1017 | GEGDCWYT | -0.28 | Non-Toxin | -0.1 | 0.16 | 930.06 |
| 1018 | GEGDCWYC | -0.03 | Non-Toxin | -0.07 | 0.16 | 932.09 |
| 1019 | GEGDCWYN | -0.28 | Non-Toxin | -0.15 | 0.16 | 943.06 |
| 1020 | GEGDCWYQ | -0.02 | Non-Toxin | -0.16 | 0.32 | 957.09 |
| 1021 | GEGDCYFS | -0.37 | Non-Toxin | -0.08 | 0.16 | 876.99 |
| 1022 | GEGDCYFT | -0.28 | Non-Toxin | -0.07 | 0.16 | 891.02 |
| 1023 | GEGDCYFC | -0.02 | Non-Toxin | -0.04 | 0.16 | 893.05 |
| 1024 | GEGDCYFN | -0.28 | Non-Toxin | -0.12 | 0.16 | 904.02 |
| 1025 | GEGDCYFQ | -0.39 | Non-Toxin | -0.13 | 0.32 | 918.05 |
| 1026 | GEGDCYWS | -0.3 | Non-Toxin | -0.11 | 0.16 | 916.03 |
| 1027 | GEGDCYWT | -0.3 | Non-Toxin | -0.1 | 0.16 | 930.06 |
| 1028 | GEGDCYWC | 0.2 | Toxin | -0.07 | 0.16 | 932.09 |
| 1029 | GEGDCYWN | -0.41 | Non-Toxin | -0.15 | 0.16 | 943.06 |
| 1030 | GEGDCYWQ | -0.29 | Non-Toxin | -0.16 | 0.32 | 957.09 |
| 1031 | GEGDCYYS | -0.26 | Non-Toxin | -0.15 | 0.16 | 892.99 |
| 1032 | GEGDCYYT | -0.3 | Non-Toxin | -0.14 | 0.16 | 907.02 |
| 1033 | GEGDCYYC | 0.01 | Toxin | -0.11 | 0.16 | 909.05 |
| 1034 | GEGDCYYN | -0.23 | Non-Toxin | -0.2 | 0.16 | 920.02 |
| 1035 | GEGDCYYQ | -0.01 | Non-Toxin | -0.2 | 0.32 | 934.05 |
| 1036 | GEGDNFFS | -1.11 | Non-Toxin | -0.09 | 0.16 | 871.96 |
| 1037 | GEGDNFFT | -1.05 | Non-Toxin | -0.08 | 0.16 | 885.99 |
| 1038 | GEGDNFFC | -0.76 | Non-Toxin | -0.05 | 0.16 | 888.02 |
| 1039 | GEGDNFFN | -1.02 | Non-Toxin | -0.14 | 0.16 | 898.99 |
| 1040 | GEGDNFFQ | -1.12 | Non-Toxin | -0.14 | 0.32 | 913.02 |
| 1041 | GEGDNFWS | -1.24 | Non-Toxin | -0.12 | 0.16 | 911 |
| 1042 | GEGDNFWT | -1.2 | Non-Toxin | -0.11 | 0.16 | 925.03 |
| 1043 | GEGDNFWC | -0.7 | Non-Toxin | -0.08 | 0.16 | 927.06 |
| 1044 | GEGDNFWN | -1.3 | Non-Toxin | -0.17 | 0.16 | 938.03 |
| 1045 | GEGDNFWQ | -1.2 | Non-Toxin | -0.17 | 0.32 | 952.06 |
| 1046 | GEGDNFYS | -1.38 | Non-Toxin | -0.16 | 0.16 | 887.96 |
| 1047 | GEGDNFYT | -1.34 | Non-Toxin | -0.15 | 0.16 | 901.99 |
| 1048 | GEGDNFYC | -1.13 | Non-Toxin | -0.12 | 0.16 | 904.02 |
| 1049 | GEGDNFYN | -1.33 | Non-Toxin | -0.21 | 0.16 | 914.99 |
| 1050 | GEGDNFYQ | -1.12 | Non-Toxin | -0.22 | 0.32 | 929.02 |
| 1051 | GEGDNWFS | -1.02 | Non-Toxin | -0.12 | 0.16 | 911 |
| 1052 | GEGDNWFT | -0.98 | Non-Toxin | -0.11 | 0.16 | 925.03 |
| 1053 | GEGDNWFC | -0.7 | Non-Toxin | -0.08 | 0.16 | 927.06 |
| 1054 | GEGDNWFN | -0.95 | Non-Toxin | -0.17 | 0.16 | 938.03 |
| 1055 | GEGDNWFQ | -1.04 | Non-Toxin | -0.17 | 0.32 | 952.06 |
| 1056 | GEGDNWWS | -0.98 | Non-Toxin | -0.15 | 0.16 | 950.04 |
| 1057 | GEGDNWWT | -0.95 | Non-Toxin | -0.14 | 0.16 | 964.07 |
| 1058 | GEGDNWWC | -0.43 | Non-Toxin | -0.11 | 0.16 | 966.1 |
| 1059 | GEGDNWWN | -1.04 | Non-Toxin | -0.2 | 0.16 | 977.07 |
| 1060 | GEGDNWWQ | -0.94 | Non-Toxin | -0.2 | 0.32 | 991.1 |
| 1061 | GEGDNWYS | -0.89 | Non-Toxin | -0.19 | 0.16 | 927 |
| 1062 | GEGDNWYT | -0.87 | Non-Toxin | -0.18 | 0.16 | 941.03 |
| 1063 | GEGDNWYC | -0.59 | Non-Toxin | -0.15 | 0.16 | 943.06 |
| 1064 | GEGDNWYN | -0.84 | Non-Toxin | -0.24 | 0.16 | 954.03 |
| 1065 | GEGDNWYQ | -0.6 | Non-Toxin | -0.24 | 0.32 | 968.06 |
| 1066 | GEGDNYFS | -0.91 | Non-Toxin | -0.16 | 0.16 | 887.96 |
| 1067 | GEGDNYFT | -0.87 | Non-Toxin | -0.15 | 0.16 | 901.99 |
| 1068 | GEGDNYFC | -0.61 | Non-Toxin | -0.12 | 0.16 | 904.02 |
| 1069 | GEGDNYFN | -0.88 | Non-Toxin | -0.21 | 0.16 | 914.99 |
| 1070 | GEGDNYFQ | -0.96 | Non-Toxin | -0.22 | 0.32 | 929.02 |
| 1071 | GEGDNYWS | -0.99 | Non-Toxin | -0.19 | 0.16 | 927 |
| 1072 | GEGDNYWT | -0.97 | Non-Toxin | -0.18 | 0.16 | 941.03 |
| 1073 | GEGDNYWC | -0.45 | Non-Toxin | -0.15 | 0.16 | 943.06 |
| 1074 | GEGDNYWN | -1.07 | Non-Toxin | -0.24 | 0.16 | 954.03 |
| 1075 | GEGDNYWQ | -0.96 | Non-Toxin | -0.24 | 0.32 | 968.06 |
| 1076 | GEGDNYYS | -0.93 | Non-Toxin | -0.24 | 0.16 | 903.96 |
| 1077 | GEGDNYYT | -0.86 | Non-Toxin | -0.23 | 0.16 | 917.99 |
| 1078 | GEGDNYYC | -0.5 | Non-Toxin | -0.2 | 0.16 | 920.02 |
| 1079 | GEGDNYYN | -0.89 | Non-Toxin | -0.28 | 0.16 | 930.99 |
| 1080 | GEGDNYYQ | -0.63 | Non-Toxin | -0.29 | 0.32 | 945.02 |
| 1081 | GEGDQFFS | -0.79 | Non-Toxin | -0.09 | 0.32 | 885.99 |
| 1082 | GEGDQFFT | -0.72 | Non-Toxin | -0.08 | 0.32 | 900.02 |
| 1083 | GEGDQFFC | -0.44 | Non-Toxin | -0.06 | 0.32 | 902.05 |
| 1084 | GEGDQFFN | -0.68 | Non-Toxin | -0.14 | 0.32 | 913.02 |
| 1085 | GEGDQFFQ | -0.8 | Non-Toxin | -0.15 | 0.47 | 927.05 |
| 1086 | GEGDQFWS | -0.89 | Non-Toxin | -0.12 | 0.32 | 925.03 |
| 1087 | GEGDQFWT | -0.84 | Non-Toxin | -0.11 | 0.32 | 939.06 |
| 1088 | GEGDQFWC | -0.33 | Non-Toxin | -0.09 | 0.32 | 941.09 |
| 1089 | GEGDQFWN | -0.94 | Non-Toxin | -0.17 | 0.32 | 952.06 |
| 1090 | GEGDQFWQ | -0.85 | Non-Toxin | -0.18 | 0.47 | 966.09 |
| 1091 | GEGDQFYS | -0.99 | Non-Toxin | -0.17 | 0.32 | 901.99 |
| 1092 | GEGDQFYT | -0.96 | Non-Toxin | -0.16 | 0.32 | 916.02 |
| 1093 | GEGDQFYC | -0.73 | Non-Toxin | -0.13 | 0.32 | 918.05 |
| 1094 | GEGDQFYN | -0.98 | Non-Toxin | -0.22 | 0.32 | 929.02 |
| 1095 | GEGDQFYQ | -0.72 | Non-Toxin | -0.22 | 0.47 | 943.05 |
| 1096 | GEGDQWFS | -0.86 | Non-Toxin | -0.12 | 0.32 | 925.03 |
| 1097 | GEGDQWFT | -0.82 | Non-Toxin | -0.11 | 0.32 | 939.06 |
| 1098 | GEGDQWFC | -0.54 | Non-Toxin | -0.09 | 0.32 | 941.09 |
| 1099 | GEGDQWFN | -0.78 | Non-Toxin | -0.17 | 0.32 | 952.06 |
| 1100 | GEGDQWFQ | -0.86 | Non-Toxin | -0.18 | 0.47 | 966.09 |
| 1101 | GEGDQWWS | -0.81 | Non-Toxin | -0.15 | 0.32 | 964.07 |
| 1102 | GEGDQWWT | -0.78 | Non-Toxin | -0.14 | 0.32 | 978.1 |
| 1103 | GEGDQWWC | -0.26 | Non-Toxin | -0.12 | 0.32 | 980.13 |
| 1104 | GEGDQWWN | -0.87 | Non-Toxin | -0.2 | 0.32 | 991.1 |
| 1105 | GEGDQWWQ | -0.77 | Non-Toxin | -0.21 | 0.47 | 1005.13 |
| 1106 | GEGDQWYS | -0.68 | Non-Toxin | -0.2 | 0.32 | 941.03 |
| 1107 | GEGDQWYT | -0.67 | Non-Toxin | -0.19 | 0.32 | 955.06 |
| 1108 | GEGDQWYC | -0.38 | Non-Toxin | -0.16 | 0.32 | 957.09 |
| 1109 | GEGDQWYN | -0.67 | Non-Toxin | -0.24 | 0.32 | 968.06 |
| 1110 | GEGDQWYQ | -0.42 | Non-Toxin | -0.25 | 0.47 | 982.09 |
| 1111 | GEGDQYFS | -0.93 | Non-Toxin | -0.17 | 0.32 | 901.99 |
| 1112 | GEGDQYFT | -0.86 | Non-Toxin | -0.16 | 0.32 | 916.02 |
| 1113 | GEGDQYFC | -0.62 | Non-Toxin | -0.13 | 0.32 | 918.05 |
| 1114 | GEGDQYFN | -0.87 | Non-Toxin | -0.22 | 0.32 | 929.02 |
| 1115 | GEGDQYFQ | -0.95 | Non-Toxin | -0.22 | 0.47 | 943.05 |
| 1116 | GEGDQYWS | -0.98 | Non-Toxin | -0.2 | 0.32 | 941.03 |
| 1117 | GEGDQYWT | -0.94 | Non-Toxin | -0.19 | 0.32 | 955.06 |
| 1118 | GEGDQYWC | -0.42 | Non-Toxin | -0.16 | 0.32 | 957.09 |
| 1119 | GEGDQYWN | -1.04 | Non-Toxin | -0.24 | 0.32 | 968.06 |
| 1120 | GEGDQYWQ | -0.93 | Non-Toxin | -0.25 | 0.47 | 982.09 |
| 1121 | GEGDQYYS | -0.88 | Non-Toxin | -0.24 | 0.32 | 917.99 |
| 1122 | GEGDQYYT | -0.85 | Non-Toxin | -0.23 | 0.32 | 932.02 |
| 1123 | GEGDQYYC | -0.53 | Non-Toxin | -0.2 | 0.32 | 934.05 |
| 1124 | GEGDQYYN | -0.86 | Non-Toxin | -0.29 | 0.32 | 945.02 |
| 1125 | GEGDQYYQ | -0.62 | Non-Toxin | -0.29 | 0.47 | 959.05 |
| 1126 | GEGESFFS | -0.96 | Non-Toxin | -0.03 | 0.32 | 858.96 |
| 1127 | GEGESFFT | -0.96 | Non-Toxin | -0.02 | 0.32 | 872.99 |
| 1128 | GEGESFFC | -0.79 | Non-Toxin | 0.01 | 0.32 | 875.02 |
| 1129 | GEGESFFN | -0.95 | Non-Toxin | -0.07 | 0.32 | 885.99 |
| 1130 | GEGESFFQ | -0.97 | Non-Toxin | -0.08 | 0.47 | 900.02 |
| 1131 | GEGESFWS | -1.1 | Non-Toxin | -0.06 | 0.32 | 898 |
| 1132 | GEGESFWT | -1.04 | Non-Toxin | -0.05 | 0.32 | 912.03 |
| 1133 | GEGESFWC | -0.69 | Non-Toxin | -0.02 | 0.32 | 914.06 |
| 1134 | GEGESFWN | -1.11 | Non-Toxin | -0.1 | 0.32 | 925.03 |
| 1135 | GEGESFWQ | -1.05 | Non-Toxin | -0.11 | 0.47 | 939.06 |
| 1136 | GEGESFYS | -1.2 | Non-Toxin | -0.1 | 0.32 | 874.96 |
| 1137 | GEGESFYT | -1.22 | Non-Toxin | -0.09 | 0.32 | 888.99 |
| 1138 | GEGESFYC | -1.08 | Non-Toxin | -0.06 | 0.32 | 891.02 |
| 1139 | GEGESFYN | -1.19 | Non-Toxin | -0.15 | 0.32 | 901.99 |
| 1140 | GEGESFYQ | -0.99 | Non-Toxin | -0.15 | 0.47 | 916.02 |
| 1141 | GEGESWFS | -1.15 | Non-Toxin | -0.06 | 0.32 | 898 |
| 1142 | GEGESWFT | -1.15 | Non-Toxin | -0.05 | 0.32 | 912.03 |
| 1143 | GEGESWFC | -0.97 | Non-Toxin | -0.02 | 0.32 | 914.06 |
| 1144 | GEGESWFN | -1.12 | Non-Toxin | -0.1 | 0.32 | 925.03 |
| 1145 | GEGESWFQ | -1.16 | Non-Toxin | -0.11 | 0.47 | 939.06 |
| 1146 | GEGESWWS | -1.14 | Non-Toxin | -0.09 | 0.32 | 937.04 |
| 1147 | GEGESWWT | -1.11 | Non-Toxin | -0.08 | 0.32 | 951.07 |
| 1148 | GEGESWWC | -0.76 | Non-Toxin | -0.05 | 0.32 | 953.1 |
| 1149 | GEGESWWN | -1.18 | Non-Toxin | -0.14 | 0.32 | 964.07 |
| 1150 | GEGESWWQ | -1.11 | Non-Toxin | -0.14 | 0.47 | 978.1 |
| 1151 | GEGESWYS | -1.05 | Non-Toxin | -0.13 | 0.32 | 914 |
| 1152 | GEGESWYT | -1.08 | Non-Toxin | -0.12 | 0.32 | 928.03 |
| 1153 | GEGESWYC | -0.89 | Non-Toxin | -0.09 | 0.32 | 930.06 |
| 1154 | GEGESWYN | -1.04 | Non-Toxin | -0.18 | 0.32 | 941.03 |
| 1155 | GEGESWYQ | -0.83 | Non-Toxin | -0.18 | 0.47 | 955.06 |
| 1156 | GEGESYFS | -1.1 | Non-Toxin | -0.1 | 0.32 | 874.96 |
| 1157 | GEGESYFT | -1.1 | Non-Toxin | -0.09 | 0.32 | 888.99 |
| 1158 | GEGESYFC | -0.93 | Non-Toxin | -0.06 | 0.32 | 891.02 |
| 1159 | GEGESYFN | -1.09 | Non-Toxin | -0.15 | 0.32 | 901.99 |
| 1160 | GEGESYFQ | -1.13 | Non-Toxin | -0.15 | 0.47 | 916.02 |
| 1161 | GEGESYWS | -1.15 | Non-Toxin | -0.13 | 0.32 | 914 |
| 1162 | GEGESYWT | -1.12 | Non-Toxin | -0.12 | 0.32 | 928.03 |
| 1163 | GEGESYWC | -0.77 | Non-Toxin | -0.09 | 0.32 | 930.06 |
| 1164 | GEGESYWN | -1.19 | Non-Toxin | -0.18 | 0.32 | 941.03 |
| 1165 | GEGESYWQ | -1.11 | Non-Toxin | -0.18 | 0.47 | 955.06 |
| 1166 | GEGESYYS | -1.06 | Non-Toxin | -0.17 | 0.32 | 890.96 |
| 1167 | GEGESYYT | -1.09 | Non-Toxin | -0.16 | 0.32 | 904.99 |
| 1168 | GEGESYYC | -0.85 | Non-Toxin | -0.14 | 0.32 | 907.02 |
| 1169 | GEGESYYN | -1.05 | Non-Toxin | -0.22 | 0.32 | 917.99 |
| 1170 | GEGESYYQ | -0.83 | Non-Toxin | -0.23 | 0.47 | 932.02 |
| 1171 | GEGETFFS | -1.18 | Non-Toxin | -0.02 | 0.32 | 872.99 |
| 1172 | GEGETFFT | -1.21 | Non-Toxin | -0.01 | 0.32 | 887.02 |
| 1173 | GEGETFFC | -1.01 | Non-Toxin | 0.02 | 0.32 | 889.05 |
| 1174 | GEGETFFN | -1.13 | Non-Toxin | -0.06 | 0.32 | 900.02 |
| 1175 | GEGETFFQ | -1.21 | Non-Toxin | -0.07 | 0.47 | 914.05 |
| 1176 | GEGETFWS | -1.28 | Non-Toxin | -0.05 | 0.32 | 912.03 |
| 1177 | GEGETFWT | -1.24 | Non-Toxin | -0.04 | 0.32 | 926.06 |
| 1178 | GEGETFWC | -0.9 | Non-Toxin | -0.01 | 0.32 | 928.09 |
| 1179 | GEGETFWN | -1.32 | Non-Toxin | -0.09 | 0.32 | 939.06 |
| 1180 | GEGETFWQ | -1.25 | Non-Toxin | -0.1 | 0.47 | 953.09 |
| 1181 | GEGETFYS | -1.39 | Non-Toxin | -0.09 | 0.32 | 888.99 |
| 1182 | GEGETFYT | -1.4 | Non-Toxin | -0.08 | 0.32 | 903.02 |
| 1183 | GEGETFYC | -1.27 | Non-Toxin | -0.05 | 0.32 | 905.05 |
| 1184 | GEGETFYN | -1.38 | Non-Toxin | -0.14 | 0.32 | 916.02 |
| 1185 | GEGETFYQ | -1.19 | Non-Toxin | -0.14 | 0.47 | 930.05 |
| 1186 | GEGETWFS | -1.15 | Non-Toxin | -0.05 | 0.32 | 912.03 |
| 1187 | GEGETWFT | -1.17 | Non-Toxin | -0.04 | 0.32 | 926.06 |
| 1188 | GEGETWFC | -0.99 | Non-Toxin | -0.01 | 0.32 | 928.09 |
| 1189 | GEGETWFN | -1.11 | Non-Toxin | -0.09 | 0.32 | 939.06 |
| 1190 | GEGETWFQ | -1.18 | Non-Toxin | -0.1 | 0.47 | 953.09 |
| 1191 | GEGETWWS | -1.14 | Non-Toxin | -0.08 | 0.32 | 951.07 |
| 1192 | GEGETWWT | -1.12 | Non-Toxin | -0.07 | 0.32 | 965.1 |
| 1193 | GEGETWWC | -0.75 | Non-Toxin | -0.04 | 0.32 | 967.13 |
| 1194 | GEGETWWN | -1.19 | Non-Toxin | -0.12 | 0.32 | 978.1 |
| 1195 | GEGETWWQ | -1.12 | Non-Toxin | -0.13 | 0.47 | 992.13 |
| 1196 | GEGETWYS | -1.08 | Non-Toxin | -0.12 | 0.32 | 928.03 |
| 1197 | GEGETWYT | -1.09 | Non-Toxin | -0.11 | 0.32 | 942.06 |
| 1198 | GEGETWYC | -0.91 | Non-Toxin | -0.08 | 0.32 | 944.09 |
| 1199 | GEGETWYN | -1.06 | Non-Toxin | -0.17 | 0.32 | 955.06 |
| 1200 | GEGETWYQ | -0.86 | Non-Toxin | -0.17 | 0.47 | 969.09 |
| 1201 | GEGETYFS | -1.08 | Non-Toxin | -0.09 | 0.32 | 888.99 |
| 1202 | GEGETYFT | -1.09 | Non-Toxin | -0.08 | 0.32 | 903.02 |
| 1203 | GEGETYFC | -0.93 | Non-Toxin | -0.05 | 0.32 | 905.05 |
| 1204 | GEGETYFN | -1.08 | Non-Toxin | -0.14 | 0.32 | 916.02 |
| 1205 | GEGETYFQ | -1.13 | Non-Toxin | -0.14 | 0.47 | 930.05 |
| 1206 | GEGETYWS | -1.13 | Non-Toxin | -0.12 | 0.32 | 928.03 |
| 1207 | GEGETYWT | -1.12 | Non-Toxin | -0.11 | 0.32 | 942.06 |
| 1208 | GEGETYWC | -0.76 | Non-Toxin | -0.08 | 0.32 | 944.09 |
| 1209 | GEGETYWN | -1.19 | Non-Toxin | -0.17 | 0.32 | 955.06 |
| 1210 | GEGETYWQ | -1.11 | Non-Toxin | -0.17 | 0.47 | 969.09 |
| 1211 | GEGETYYS | -1.09 | Non-Toxin | -0.16 | 0.32 | 904.99 |
| 1212 | GEGETYYT | -1.07 | Non-Toxin | -0.15 | 0.32 | 919.02 |
| 1213 | GEGETYYC | -0.89 | Non-Toxin | -0.13 | 0.32 | 921.05 |
| 1214 | GEGETYYN | -1.07 | Non-Toxin | -0.21 | 0.32 | 932.02 |
| 1215 | GEGETYYQ | -0.86 | Non-Toxin | -0.22 | 0.47 | 946.05 |
| 1216 | GEGECFFS | -0.7 | Non-Toxin | 0.01 | 0.32 | 875.02 |
| 1217 | GEGECFFT | -0.68 | Non-Toxin | 0.02 | 0.32 | 889.05 |
| 1218 | GEGECFFC | -0.48 | Non-Toxin | 0.05 | 0.32 | 891.08 |
| 1219 | GEGECFFN | -0.65 | Non-Toxin | -0.04 | 0.32 | 902.05 |
| 1220 | GEGECFFQ | -0.73 | Non-Toxin | -0.04 | 0.47 | 916.08 |
| 1221 | GEGECFWS | -0.89 | Non-Toxin | -0.02 | 0.32 | 914.06 |
| 1222 | GEGECFWT | -0.87 | Non-Toxin | -0.01 | 0.32 | 928.09 |
| 1223 | GEGECFWC | -0.52 | Non-Toxin | 0.02 | 0.32 | 930.12 |
| 1224 | GEGECFWN | -0.93 | Non-Toxin | -0.07 | 0.32 | 941.09 |
| 1225 | GEGECFWQ | -0.86 | Non-Toxin | -0.07 | 0.47 | 955.12 |
| 1226 | GEGECFYS | -0.93 | Non-Toxin | -0.06 | 0.32 | 891.02 |
| 1227 | GEGECFYT | -0.94 | Non-Toxin | -0.05 | 0.32 | 905.05 |
| 1228 | GEGECFYC | -0.87 | Non-Toxin | -0.03 | 0.32 | 907.08 |
| 1229 | GEGECFYN | -0.92 | Non-Toxin | -0.11 | 0.32 | 918.05 |
| 1230 | GEGECFYQ | -0.73 | Non-Toxin | -0.12 | 0.47 | 932.08 |
| 1231 | GEGECWFS | -0.64 | Non-Toxin | -0.02 | 0.32 | 914.06 |
| 1232 | GEGECWFT | -0.62 | Non-Toxin | -0.01 | 0.32 | 928.09 |
| 1233 | GEGECWFC | -0.44 | Non-Toxin | 0.02 | 0.32 | 930.12 |
| 1234 | GEGECWFN | -0.58 | Non-Toxin | -0.07 | 0.32 | 941.09 |
| 1235 | GEGECWFQ | -0.65 | Non-Toxin | -0.07 | 0.47 | 955.12 |
| 1236 | GEGECWWS | -0.6 | Non-Toxin | -0.05 | 0.32 | 953.1 |
| 1237 | GEGECWWT | -0.59 | Non-Toxin | -0.04 | 0.32 | 967.13 |
| 1238 | GEGECWWC | -0.22 | Non-Toxin | -0.01 | 0.32 | 969.16 |
| 1239 | GEGECWWN | -0.65 | Non-Toxin | -0.1 | 0.32 | 980.13 |
| 1240 | GEGECWWQ | -0.58 | Non-Toxin | -0.1 | 0.47 | 994.16 |
| 1241 | GEGECWYS | -0.54 | Non-Toxin | -0.09 | 0.32 | 930.06 |
| 1242 | GEGECWYT | -0.53 | Non-Toxin | -0.08 | 0.32 | 944.09 |
| 1243 | GEGECWYC | -0.38 | Non-Toxin | -0.06 | 0.32 | 946.12 |
| 1244 | GEGECWYN | -0.52 | Non-Toxin | -0.14 | 0.32 | 957.09 |
| 1245 | GEGECWYQ | -0.31 | Non-Toxin | -0.15 | 0.47 | 971.12 |
| 1246 | GEGECYFS | -0.66 | Non-Toxin | -0.06 | 0.32 | 891.02 |
| 1247 | GEGECYFT | -0.62 | Non-Toxin | -0.05 | 0.32 | 905.05 |
| 1248 | GEGECYFC | -0.48 | Non-Toxin | -0.03 | 0.32 | 907.08 |
| 1249 | GEGECYFN | -0.61 | Non-Toxin | -0.11 | 0.32 | 918.05 |
| 1250 | GEGECYFQ | -0.68 | Non-Toxin | -0.12 | 0.47 | 932.08 |
| 1251 | GEGECYWS | -0.63 | Non-Toxin | -0.09 | 0.32 | 930.06 |
| 1252 | GEGECYWT | -0.62 | Non-Toxin | -0.08 | 0.32 | 944.09 |
| 1253 | GEGECYWC | -0.28 | Non-Toxin | -0.06 | 0.32 | 946.12 |
| 1254 | GEGECYWN | -0.7 | Non-Toxin | -0.14 | 0.32 | 957.09 |
| 1255 | GEGECYWQ | -0.61 | Non-Toxin | -0.15 | 0.47 | 971.12 |
| 1256 | GEGECYYS | -0.57 | Non-Toxin | -0.14 | 0.32 | 907.02 |
| 1257 | GEGECYYT | -0.6 | Non-Toxin | -0.13 | 0.32 | 921.05 |
| 1258 | GEGECYYC | -0.41 | Non-Toxin | -0.1 | 0.32 | 923.08 |
| 1259 | GEGECYYN | -0.54 | Non-Toxin | -0.18 | 0.32 | 934.05 |
| 1260 | GEGECYYQ | -0.36 | Non-Toxin | -0.19 | 0.47 | 948.08 |
| 1261 | GEGENFFS | -1.1 | Non-Toxin | -0.08 | 0.32 | 885.99 |
| 1262 | GEGENFFT | -1.07 | Non-Toxin | -0.07 | 0.32 | 900.02 |
| 1263 | GEGENFFC | -0.89 | Non-Toxin | -0.04 | 0.32 | 902.05 |
| 1264 | GEGENFFN | -1.03 | Non-Toxin | -0.12 | 0.32 | 913.02 |
| 1265 | GEGENFFQ | -1.1 | Non-Toxin | -0.13 | 0.47 | 927.05 |
| 1266 | GEGENFWS | -1.18 | Non-Toxin | -0.11 | 0.32 | 925.03 |
| 1267 | GEGENFWT | -1.16 | Non-Toxin | -0.1 | 0.32 | 939.06 |
| 1268 | GEGENFWC | -0.82 | Non-Toxin | -0.07 | 0.32 | 941.09 |
| 1269 | GEGENFWN | -1.23 | Non-Toxin | -0.15 | 0.32 | 952.06 |
| 1270 | GEGENFWQ | -1.16 | Non-Toxin | -0.16 | 0.47 | 966.09 |
| 1271 | GEGENFYS | -1.27 | Non-Toxin | -0.15 | 0.32 | 901.99 |
| 1272 | GEGENFYT | -1.27 | Non-Toxin | -0.14 | 0.32 | 916.02 |
| 1273 | GEGENFYC | -1.14 | Non-Toxin | -0.11 | 0.32 | 918.05 |
| 1274 | GEGENFYN | -1.27 | Non-Toxin | -0.2 | 0.32 | 929.02 |
| 1275 | GEGENFYQ | -1.08 | Non-Toxin | -0.2 | 0.47 | 943.05 |
| 1276 | GEGENWFS | -1.03 | Non-Toxin | -0.1 | 0.32 | 925.03 |
| 1277 | GEGENWFT | -1.02 | Non-Toxin | -0.1 | 0.32 | 939.06 |
| 1278 | GEGENWFC | -0.84 | Non-Toxin | -0.07 | 0.32 | 941.09 |
| 1279 | GEGENWFN | -0.98 | Non-Toxin | -0.15 | 0.32 | 952.06 |
| 1280 | GEGENWFQ | -1.04 | Non-Toxin | -0.16 | 0.47 | 966.09 |
| 1281 | GEGENWWS | -1 | Non-Toxin | -0.14 | 0.32 | 964.07 |
| 1282 | GEGENWWT | -0.99 | Non-Toxin | -0.12 | 0.32 | 978.1 |
| 1283 | GEGENWWC | -0.64 | Non-Toxin | -0.1 | 0.32 | 980.13 |
| 1284 | GEGENWWN | -1.05 | Non-Toxin | -0.18 | 0.32 | 991.1 |
| 1285 | GEGENWWQ | -0.98 | Non-Toxin | -0.19 | 0.47 | 1005.13 |
| 1286 | GEGENWYS | -0.93 | Non-Toxin | -0.18 | 0.32 | 941.03 |
| 1287 | GEGENWYT | -0.95 | Non-Toxin | -0.17 | 0.32 | 955.06 |
| 1288 | GEGENWYC | -0.77 | Non-Toxin | -0.14 | 0.32 | 957.09 |
| 1289 | GEGENWYN | -0.93 | Non-Toxin | -0.23 | 0.32 | 968.06 |
| 1290 | GEGENWYQ | -0.72 | Non-Toxin | -0.23 | 0.47 | 982.09 |
| 1291 | GEGENYFS | -0.98 | Non-Toxin | -0.15 | 0.32 | 901.99 |
| 1292 | GEGENYFT | -0.96 | Non-Toxin | -0.14 | 0.32 | 916.02 |
| 1293 | GEGENYFC | -0.8 | Non-Toxin | -0.11 | 0.32 | 918.05 |
| 1294 | GEGENYFN | -0.95 | Non-Toxin | -0.2 | 0.32 | 929.02 |
| 1295 | GEGENYFQ | -1 | Non-Toxin | -0.2 | 0.47 | 943.05 |
| 1296 | GEGENYWS | -1.02 | Non-Toxin | -0.18 | 0.32 | 941.03 |
| 1297 | GEGENYWT | -1 | Non-Toxin | -0.17 | 0.32 | 955.06 |
| 1298 | GEGENYWC | -0.65 | Non-Toxin | -0.14 | 0.32 | 957.09 |
| 1299 | GEGENYWN | -1.07 | Non-Toxin | -0.23 | 0.32 | 968.06 |
| 1300 | GEGENYWQ | -1 | Non-Toxin | -0.23 | 0.47 | 982.09 |
| 1301 | GEGENYYS | -0.94 | Non-Toxin | -0.22 | 0.32 | 917.99 |
| 1302 | GEGENYYT | -0.93 | Non-Toxin | -0.21 | 0.32 | 932.02 |
| 1303 | GEGENYYC | -0.7 | Non-Toxin | -0.18 | 0.32 | 934.05 |
| 1304 | GEGENYYN | -0.94 | Non-Toxin | -0.27 | 0.32 | 945.02 |
| 1305 | GEGENYYQ | -0.73 | Non-Toxin | -0.28 | 0.47 | 959.05 |
| 1306 | GEGEQFFS | -0.94 | Non-Toxin | -0.08 | 0.47 | 900.02 |
| 1307 | GEGEQFFT | -0.91 | Non-Toxin | -0.07 | 0.47 | 914.05 |
| 1308 | GEGEQFFC | -0.73 | Non-Toxin | -0.04 | 0.47 | 916.08 |
| 1309 | GEGEQFFN | -0.87 | Non-Toxin | -0.13 | 0.47 | 927.05 |
| 1310 | GEGEQFFQ | -0.95 | Non-Toxin | -0.13 | 0.63 | 941.08 |
| 1311 | GEGEQFWS | -1.04 | Non-Toxin | -0.11 | 0.47 | 939.06 |
| 1312 | GEGEQFWT | -1 | Non-Toxin | -0.1 | 0.47 | 953.09 |
| 1313 | GEGEQFWC | -0.67 | Non-Toxin | -0.07 | 0.47 | 955.12 |
| 1314 | GEGEQFWN | -1.08 | Non-Toxin | -0.16 | 0.47 | 966.09 |
| 1315 | GEGEQFWQ | -1.01 | Non-Toxin | -0.16 | 0.63 | 980.12 |
| 1316 | GEGEQFYS | -1.14 | Non-Toxin | -0.15 | 0.47 | 916.02 |
| 1317 | GEGEQFYT | -1.13 | Non-Toxin | -0.14 | 0.47 | 930.05 |
| 1318 | GEGEQFYC | -1 | Non-Toxin | -0.12 | 0.47 | 932.08 |
| 1319 | GEGEQFYN | -1.12 | Non-Toxin | -0.2 | 0.47 | 943.05 |
| 1320 | GEGEQFYQ | -0.92 | Non-Toxin | -0.21 | 0.63 | 957.08 |
| 1321 | GEGEQWFS | -1 | Non-Toxin | -0.11 | 0.47 | 939.06 |
| 1322 | GEGEQWFT | -0.98 | Non-Toxin | -0.1 | 0.47 | 953.09 |
| 1323 | GEGEQWFC | -0.8 | Non-Toxin | -0.07 | 0.47 | 955.12 |
| 1324 | GEGEQWFN | -0.95 | Non-Toxin | -0.16 | 0.47 | 966.09 |
| 1325 | GEGEQWFQ | -1 | Non-Toxin | -0.16 | 0.63 | 980.12 |
| 1326 | GEGEQWWS | -0.99 | Non-Toxin | -0.14 | 0.47 | 978.1 |
| 1327 | GEGEQWWT | -0.96 | Non-Toxin | -0.13 | 0.47 | 992.13 |
| 1328 | GEGEQWWC | -0.61 | Non-Toxin | -0.1 | 0.47 | 994.16 |
| 1329 | GEGEQWWN | -1.02 | Non-Toxin | -0.19 | 0.47 | 1005.13 |
| 1330 | GEGEQWWQ | -0.95 | Non-Toxin | -0.19 | 0.63 | 1019.16 |
| 1331 | GEGEQWYS | -0.9 | Non-Toxin | -0.18 | 0.47 | 955.06 |
| 1332 | GEGEQWYT | -0.91 | Non-Toxin | -0.17 | 0.47 | 969.09 |
| 1333 | GEGEQWYC | -0.74 | Non-Toxin | -0.15 | 0.47 | 971.12 |
| 1334 | GEGEQWYN | -0.89 | Non-Toxin | -0.23 | 0.47 | 982.09 |
| 1335 | GEGEQWYQ | -0.7 | Non-Toxin | -0.24 | 0.63 | 996.12 |
| 1336 | GEGEQYFS | -1.07 | Non-Toxin | -0.15 | 0.47 | 916.02 |
| 1337 | GEGEQYFT | -1.04 | Non-Toxin | -0.14 | 0.47 | 930.05 |
| 1338 | GEGEQYFC | -0.89 | Non-Toxin | -0.12 | 0.47 | 932.08 |
| 1339 | GEGEQYFN | -1.04 | Non-Toxin | -0.2 | 0.47 | 943.05 |
| 1340 | GEGEQYFQ | -1.09 | Non-Toxin | -0.21 | 0.63 | 957.08 |
| 1341 | GEGEQYWS | -1.1 | Non-Toxin | -0.18 | 0.47 | 955.06 |
| 1342 | GEGEQYWT | -1.07 | Non-Toxin | -0.17 | 0.47 | 969.09 |
| 1343 | GEGEQYWC | -0.72 | Non-Toxin | -0.15 | 0.47 | 971.12 |
| 1344 | GEGEQYWN | -1.14 | Non-Toxin | -0.23 | 0.47 | 982.09 |
| 1345 | GEGEQYWQ | -1.07 | Non-Toxin | -0.24 | 0.63 | 996.12 |
| 1346 | GEGEQYYS | -1.04 | Non-Toxin | -0.23 | 0.47 | 932.02 |
| 1347 | GEGEQYYT | -1.03 | Non-Toxin | -0.22 | 0.47 | 946.05 |
| 1348 | GEGEQYYC | -0.84 | Non-Toxin | -0.19 | 0.47 | 948.08 |
| 1349 | GEGEQYYN | -1.02 | Non-Toxin | -0.28 | 0.47 | 959.05 |
| 1350 | GEGEQYYQ | -0.83 | Non-Toxin | -0.28 | 0.63 | 973.08 |
| 1351 | GEPDSFFS | -1 | Non-Toxin | -0.07 | 0.16 | 884.99 |
| 1352 | GEPDSFFT | -0.97 | Non-Toxin | -0.06 | 0.16 | 899.02 |
| 1353 | GEPDSFFC | -0.68 | Non-Toxin | -0.03 | 0.16 | 901.05 |
| 1354 | GEPDSFFN | -0.98 | Non-Toxin | -0.12 | 0.16 | 912.02 |
| 1355 | GEPDSFFQ | -0.98 | Non-Toxin | -0.12 | 0.32 | 926.05 |
| 1356 | GEPDSFWS | -1.2 | Non-Toxin | -0.1 | 0.16 | 924.03 |
| 1357 | GEPDSFWT | -1.09 | Non-Toxin | -0.09 | 0.16 | 938.06 |
| 1358 | GEPDSFWC | -0.53 | Non-Toxin | -0.06 | 0.16 | 940.09 |
| 1359 | GEPDSFWN | -1.21 | Non-Toxin | -0.15 | 0.16 | 951.06 |
| 1360 | GEPDSFWQ | -1.1 | Non-Toxin | -0.15 | 0.32 | 965.09 |
| 1361 | GEPDSFYS | -1.32 | Non-Toxin | -0.14 | 0.16 | 900.99 |
| 1362 | GEPDSFYT | -1.32 | Non-Toxin | -0.13 | 0.16 | 915.02 |
| 1363 | GEPDSFYC | -1.23 | Non-Toxin | -0.1 | 0.16 | 917.05 |
| 1364 | GEPDSFYN | -1.29 | Non-Toxin | -0.19 | 0.16 | 928.02 |
| 1365 | GEPDSFYQ | -1.07 | Non-Toxin | -0.2 | 0.32 | 942.05 |
| 1366 | GEPDSWFS | -1.32 | Non-Toxin | -0.1 | 0.16 | 924.03 |
| 1367 | GEPDSWFT | -1.28 | Non-Toxin | -0.09 | 0.16 | 938.06 |
| 1368 | GEPDSWFC | -0.99 | Non-Toxin | -0.06 | 0.16 | 940.09 |
| 1369 | GEPDSWFN | -1.27 | Non-Toxin | -0.15 | 0.16 | 951.06 |
| 1370 | GEPDSWFQ | -1.31 | Non-Toxin | -0.15 | 0.32 | 965.09 |
| 1371 | GEPDSWWS | -1.28 | Non-Toxin | -0.13 | 0.16 | 963.07 |
| 1372 | GEPDSWWT | -1.23 | Non-Toxin | -0.12 | 0.16 | 977.1 |
| 1373 | GEPDSWWC | -0.67 | Non-Toxin | -0.09 | 0.16 | 979.13 |
| 1374 | GEPDSWWN | -1.33 | Non-Toxin | -0.18 | 0.16 | 990.1 |
| 1375 | GEPDSWWQ | -1.22 | Non-Toxin | -0.18 | 0.32 | 1004.13 |
| 1376 | GEPDSWYS | -1.1 | Non-Toxin | -0.17 | 0.16 | 940.03 |
| 1377 | GEPDSWYT | -1.13 | Non-Toxin | -0.16 | 0.16 | 954.06 |
| 1378 | GEPDSWYC | -0.95 | Non-Toxin | -0.14 | 0.16 | 956.09 |
| 1379 | GEPDSWYN | -1.08 | Non-Toxin | -0.22 | 0.16 | 967.06 |
| 1380 | GEPDSWYQ | -0.84 | Non-Toxin | -0.23 | 0.32 | 981.09 |
| 1381 | GEPDSYFS | -1.2 | Non-Toxin | -0.14 | 0.16 | 900.99 |
| 1382 | GEPDSYFT | -1.15 | Non-Toxin | -0.13 | 0.16 | 915.02 |
| 1383 | GEPDSYFC | -0.87 | Non-Toxin | -0.1 | 0.16 | 917.05 |
| 1384 | GEPDSYFN | -1.18 | Non-Toxin | -0.19 | 0.16 | 928.02 |
| 1385 | GEPDSYFQ | -1.21 | Non-Toxin | -0.2 | 0.32 | 942.05 |
| 1386 | GEPDSYWS | -1.25 | Non-Toxin | -0.17 | 0.16 | 940.03 |
| 1387 | GEPDSYWT | -1.2 | Non-Toxin | -0.16 | 0.16 | 954.06 |
| 1388 | GEPDSYWC | -0.64 | Non-Toxin | -0.14 | 0.16 | 956.09 |
| 1389 | GEPDSYWN | -1.3 | Non-Toxin | -0.22 | 0.16 | 967.06 |
| 1390 | GEPDSYWQ | -1.18 | Non-Toxin | -0.23 | 0.32 | 981.09 |
| 1391 | GEPDSYYS | -1.07 | Non-Toxin | -0.22 | 0.16 | 916.99 |
| 1392 | GEPDSYYT | -1.11 | Non-Toxin | -0.21 | 0.16 | 931.02 |
| 1393 | GEPDSYYC | -0.86 | Non-Toxin | -0.18 | 0.16 | 933.05 |
| 1394 | GEPDSYYN | -1.06 | Non-Toxin | -0.26 | 0.16 | 944.02 |
| 1395 | GEPDSYYQ | -0.8 | Non-Toxin | -0.27 | 0.32 | 958.05 |
| 1396 | GEPDTFFS | -0.98 | Non-Toxin | -0.06 | 0.16 | 899.02 |
| 1397 | GEPDTFFT | -0.93 | Non-Toxin | -0.05 | 0.16 | 913.05 |
| 1398 | GEPDTFFC | -0.57 | Non-Toxin | -0.02 | 0.16 | 915.08 |
| 1399 | GEPDTFFN | -0.89 | Non-Toxin | -0.11 | 0.16 | 926.05 |
| 1400 | GEPDTFFQ | -0.98 | Non-Toxin | -0.11 | 0.32 | 940.08 |
| 1401 | GEPDTFWS | -1.12 | Non-Toxin | -0.09 | 0.16 | 938.06 |
| 1402 | GEPDTFWT | -1.05 | Non-Toxin | -0.08 | 0.16 | 952.09 |
| 1403 | GEPDTFWC | -0.52 | Non-Toxin | -0.05 | 0.16 | 954.12 |
| 1404 | GEPDTFWN | -1.18 | Non-Toxin | -0.14 | 0.16 | 965.09 |
| 1405 | GEPDTFWQ | -1.06 | Non-Toxin | -0.14 | 0.32 | 979.12 |
| 1406 | GEPDTFYS | -1.21 | Non-Toxin | -0.13 | 0.16 | 915.02 |
| 1407 | GEPDTFYT | -1.25 | Non-Toxin | -0.12 | 0.16 | 929.05 |
| 1408 | GEPDTFYC | -1.08 | Non-Toxin | -0.09 | 0.16 | 931.08 |
| 1409 | GEPDTFYN | -1.22 | Non-Toxin | -0.18 | 0.16 | 942.05 |
| 1410 | GEPDTFYQ | -0.99 | Non-Toxin | -0.19 | 0.32 | 956.08 |
| 1411 | GEPDTWFS | -1.01 | Non-Toxin | -0.09 | 0.16 | 938.06 |
| 1412 | GEPDTWFT | -0.95 | Non-Toxin | -0.08 | 0.16 | 952.09 |
| 1413 | GEPDTWFC | -0.62 | Non-Toxin | -0.05 | 0.16 | 954.12 |
| 1414 | GEPDTWFN | -0.95 | Non-Toxin | -0.14 | 0.16 | 965.09 |
| 1415 | GEPDTWFQ | -1.01 | Non-Toxin | -0.14 | 0.32 | 979.12 |
| 1416 | GEPDTWWS | -0.98 | Non-Toxin | -0.12 | 0.16 | 977.1 |
| 1417 | GEPDTWWT | -0.94 | Non-Toxin | -0.11 | 0.16 | 991.13 |
| 1418 | GEPDTWWC | -0.37 | Non-Toxin | -0.08 | 0.16 | 993.16 |
| 1419 | GEPDTWWN | -1.04 | Non-Toxin | -0.17 | 0.16 | 1004.13 |
| 1420 | GEPDTWWQ | -0.93 | Non-Toxin | -0.17 | 0.32 | 1018.16 |
| 1421 | GEPDTWYS | -0.83 | Non-Toxin | -0.16 | 0.16 | 954.06 |
| 1422 | GEPDTWYT | -0.87 | Non-Toxin | -0.15 | 0.16 | 968.09 |
| 1423 | GEPDTWYC | -0.64 | Non-Toxin | -0.12 | 0.16 | 970.12 |
| 1424 | GEPDTWYN | -0.83 | Non-Toxin | -0.21 | 0.16 | 981.09 |
| 1425 | GEPDTWYQ | -0.58 | Non-Toxin | -0.22 | 0.32 | 995.12 |
| 1426 | GEPDTYFS | -0.91 | Non-Toxin | -0.13 | 0.16 | 915.02 |
| 1427 | GEPDTYFT | -0.83 | Non-Toxin | -0.12 | 0.16 | 929.05 |
| 1428 | GEPDTYFC | -0.54 | Non-Toxin | -0.09 | 0.16 | 931.08 |
| 1429 | GEPDTYFN | -0.9 | Non-Toxin | -0.18 | 0.16 | 942.05 |
| 1430 | GEPDTYFQ | -0.93 | Non-Toxin | -0.19 | 0.32 | 956.08 |
| 1431 | GEPDTYWS | -0.96 | Non-Toxin | -0.16 | 0.16 | 954.06 |
| 1432 | GEPDTYWT | -0.93 | Non-Toxin | -0.15 | 0.16 | 968.09 |
| 1433 | GEPDTYWC | -0.37 | Non-Toxin | -0.12 | 0.16 | 970.12 |
| 1434 | GEPDTYWN | -1.03 | Non-Toxin | -0.21 | 0.16 | 981.09 |
| 1435 | GEPDTYWQ | -0.91 | Non-Toxin | -0.22 | 0.32 | 995.12 |
| 1436 | GEPDTYYS | -0.86 | Non-Toxin | -0.21 | 0.16 | 931.02 |
| 1437 | GEPDTYYT | -0.86 | Non-Toxin | -0.2 | 0.16 | 945.05 |
| 1438 | GEPDTYYC | -0.61 | Non-Toxin | -0.17 | 0.16 | 947.08 |
| 1439 | GEPDTYYN | -0.85 | Non-Toxin | -0.25 | 0.16 | 958.05 |
| 1440 | GEPDTYYQ | -0.6 | Non-Toxin | -0.26 | 0.32 | 972.08 |
| 1441 | GEPDCFFS | -0.65 | Non-Toxin | -0.03 | 0.16 | 901.05 |
| 1442 | GEPDCFFT | -0.6 | Non-Toxin | -0.02 | 0.16 | 915.08 |
| 1443 | GEPDCFFC | -0.21 | Non-Toxin | 0.01 | 0.16 | 917.11 |
| 1444 | GEPDCFFN | -0.6 | Non-Toxin | -0.08 | 0.16 | 928.08 |
| 1445 | GEPDCFFQ | -0.67 | Non-Toxin | -0.08 | 0.32 | 942.11 |
| 1446 | GEPDCFWS | -0.93 | Non-Toxin | -0.06 | 0.16 | 940.09 |
| 1447 | GEPDCFWT | -0.89 | Non-Toxin | -0.05 | 0.16 | 954.12 |
| 1448 | GEPDCFWC | -0.34 | Non-Toxin | -0.02 | 0.16 | 956.15 |
| 1449 | GEPDCFWN | -0.99 | Non-Toxin | -0.11 | 0.16 | 967.12 |
| 1450 | GEPDCFWQ | -0.88 | Non-Toxin | -0.11 | 0.32 | 981.15 |
| 1451 | GEPDCFYS | -0.97 | Non-Toxin | -0.1 | 0.16 | 917.05 |
| 1452 | GEPDCFYT | -1.02 | Non-Toxin | -0.1 | 0.16 | 931.08 |
| 1453 | GEPDCFYC | -0.93 | Non-Toxin | -0.07 | 0.16 | 933.11 |
| 1454 | GEPDCFYN | -0.98 | Non-Toxin | -0.15 | 0.16 | 944.08 |
| 1455 | GEPDCFYQ | -0.75 | Non-Toxin | -0.16 | 0.32 | 958.11 |
| 1456 | GEPDCWFS | -0.61 | Non-Toxin | -0.06 | 0.16 | 940.09 |
| 1457 | GEPDCWFT | -0.55 | Non-Toxin | -0.05 | 0.16 | 954.12 |
| 1458 | GEPDCWFC | -0.19 | Non-Toxin | -0.02 | 0.16 | 956.15 |
| 1459 | GEPDCWFN | -0.53 | Non-Toxin | -0.11 | 0.16 | 967.12 |
| 1460 | GEPDCWFQ | -0.6 | Non-Toxin | -0.11 | 0.32 | 981.15 |
| 1461 | GEPDCWWS | -0.57 | Non-Toxin | -0.09 | 0.16 | 979.13 |
| 1462 | GEPDCWWT | -0.55 | Non-Toxin | -0.08 | 0.16 | 993.16 |
| 1463 | GEPDCWWC | 0.02 | Toxin | -0.05 | 0.16 | 995.19 |
| 1464 | GEPDCWWN | -0.64 | Non-Toxin | -0.14 | 0.16 | 1006.16 |
| 1465 | GEPDCWWQ | -0.53 | Non-Toxin | -0.14 | 0.32 | 1020.19 |
| 1466 | GEPDCWYS | -0.44 | Non-Toxin | -0.14 | 0.16 | 956.09 |
| 1467 | GEPDCWYT | -0.47 | Non-Toxin | -0.12 | 0.16 | 970.12 |
| 1468 | GEPDCWYC | -0.26 | Non-Toxin | -0.1 | 0.16 | 972.15 |
| 1469 | GEPDCWYN | -0.44 | Non-Toxin | -0.18 | 0.16 | 983.12 |
| 1470 | GEPDCWYQ | -0.18 | Non-Toxin | -0.19 | 0.32 | 997.15 |
| 1471 | GEPDCYFS | -0.55 | Non-Toxin | -0.1 | 0.16 | 917.05 |
| 1472 | GEPDCYFT | -0.48 | Non-Toxin | -0.1 | 0.16 | 931.08 |
| 1473 | GEPDCYFC | -0.17 | Non-Toxin | -0.07 | 0.16 | 933.11 |
| 1474 | GEPDCYFN | -0.49 | Non-Toxin | -0.15 | 0.16 | 944.08 |
| 1475 | GEPDCYFQ | -0.57 | Non-Toxin | -0.16 | 0.32 | 958.11 |
| 1476 | GEPDCYWS | -0.48 | Non-Toxin | -0.14 | 0.16 | 956.09 |
| 1477 | GEPDCYWT | -0.46 | Non-Toxin | -0.12 | 0.16 | 970.12 |
| 1478 | GEPDCYWC | 0.06 | Toxin | -0.1 | 0.16 | 972.15 |
| 1479 | GEPDCYWN | -0.58 | Non-Toxin | -0.18 | 0.16 | 983.12 |
| 1480 | GEPDCYWQ | -0.45 | Non-Toxin | -0.19 | 0.32 | 997.15 |
| 1481 | GEPDCYYS | -0.41 | Non-Toxin | -0.18 | 0.16 | 933.05 |
| 1482 | GEPDCYYT | -0.5 | Non-Toxin | -0.17 | 0.16 | 947.08 |
| 1483 | GEPDCYYC | -0.23 | Non-Toxin | -0.14 | 0.16 | 949.11 |
| 1484 | GEPDCYYN | -0.4 | Non-Toxin | -0.23 | 0.16 | 960.08 |
| 1485 | GEPDCYYQ | -0.18 | Non-Toxin | -0.23 | 0.32 | 974.11 |
| 1486 | GEPDNFFS | -1.15 | Non-Toxin | -0.12 | 0.16 | 912.02 |
| 1487 | GEPDNFFT | -1.09 | Non-Toxin | -0.11 | 0.16 | 926.05 |
| 1488 | GEPDNFFC | -0.75 | Non-Toxin | -0.08 | 0.16 | 928.08 |
| 1489 | GEPDNFFN | -1.08 | Non-Toxin | -0.16 | 0.16 | 939.05 |
| 1490 | GEPDNFFQ | -1.15 | Non-Toxin | -0.17 | 0.32 | 953.08 |
| 1491 | GEPDNFWS | -1.3 | Non-Toxin | -0.15 | 0.16 | 951.06 |
| 1492 | GEPDNFWT | -1.25 | Non-Toxin | -0.14 | 0.16 | 965.09 |
| 1493 | GEPDNFWC | -0.72 | Non-Toxin | -0.11 | 0.16 | 967.12 |
| 1494 | GEPDNFWN | -1.36 | Non-Toxin | -0.19 | 0.16 | 978.09 |
| 1495 | GEPDNFWQ | -1.25 | Non-Toxin | -0.2 | 0.32 | 992.12 |
| 1496 | GEPDNFYS | -1.42 | Non-Toxin | -0.19 | 0.16 | 928.02 |
| 1497 | GEPDNFYT | -1.42 | Non-Toxin | -0.18 | 0.16 | 942.05 |
| 1498 | GEPDNFYC | -1.27 | Non-Toxin | -0.15 | 0.16 | 944.08 |
| 1499 | GEPDNFYN | -1.39 | Non-Toxin | -0.24 | 0.16 | 955.05 |
| 1500 | GEPDNFYQ | -1.18 | Non-Toxin | -0.24 | 0.32 | 969.08 |
| 1501 | GEPDNWFS | -1.06 | Non-Toxin | -0.15 | 0.16 | 951.06 |
| 1502 | GEPDNWFT | -1.02 | Non-Toxin | -0.14 | 0.16 | 965.09 |
| 1503 | GEPDNWFC | -0.69 | Non-Toxin | -0.11 | 0.16 | 967.12 |
| 1504 | GEPDNWFN | -1.02 | Non-Toxin | -0.19 | 0.16 | 978.09 |
| 1505 | GEPDNWFQ | -1.07 | Non-Toxin | -0.2 | 0.32 | 992.12 |
| 1506 | GEPDNWWS | -1.03 | Non-Toxin | -0.18 | 0.16 | 990.1 |
| 1507 | GEPDNWWT | -1 | Non-Toxin | -0.17 | 0.16 | 1004.13 |
| 1508 | GEPDNWWC | -0.45 | Non-Toxin | -0.14 | 0.16 | 1006.16 |
| 1509 | GEPDNWWN | -1.1 | Non-Toxin | -0.22 | 0.16 | 1017.13 |
| 1510 | GEPDNWWQ | -0.99 | Non-Toxin | -0.23 | 0.32 | 1031.16 |
| 1511 | GEPDNWYS | -0.91 | Non-Toxin | -0.22 | 0.16 | 967.06 |
| 1512 | GEPDNWYT | -0.94 | Non-Toxin | -0.21 | 0.16 | 981.09 |
| 1513 | GEPDNWYC | -0.71 | Non-Toxin | -0.18 | 0.16 | 983.12 |
| 1514 | GEPDNWYN | -0.88 | Non-Toxin | -0.27 | 0.16 | 994.09 |
| 1515 | GEPDNWYQ | -0.64 | Non-Toxin | -0.27 | 0.32 | 1008.12 |
| 1516 | GEPDNYFS | -0.96 | Non-Toxin | -0.19 | 0.16 | 928.02 |
| 1517 | GEPDNYFT | -0.93 | Non-Toxin | -0.18 | 0.16 | 942.05 |
| 1518 | GEPDNYFC | -0.61 | Non-Toxin | -0.15 | 0.16 | 944.08 |
| 1519 | GEPDNYFN | -0.96 | Non-Toxin | -0.24 | 0.16 | 955.05 |
| 1520 | GEPDNYFQ | -0.99 | Non-Toxin | -0.24 | 0.32 | 969.08 |
| 1521 | GEPDNYWS | -1.03 | Non-Toxin | -0.22 | 0.16 | 967.06 |
| 1522 | GEPDNYWT | -0.99 | Non-Toxin | -0.21 | 0.16 | 981.09 |
| 1523 | GEPDNYWC | -0.44 | Non-Toxin | -0.18 | 0.16 | 983.12 |
| 1524 | GEPDNYWN | -1.11 | Non-Toxin | -0.27 | 0.16 | 994.09 |
| 1525 | GEPDNYWQ | -0.98 | Non-Toxin | -0.27 | 0.32 | 1008.12 |
| 1526 | GEPDNYYS | -0.93 | Non-Toxin | -0.26 | 0.16 | 944.02 |
| 1527 | GEPDNYYT | -0.92 | Non-Toxin | -0.25 | 0.16 | 958.05 |
| 1528 | GEPDNYYC | -0.61 | Non-Toxin | -0.23 | 0.16 | 960.08 |
| 1529 | GEPDNYYN | -0.91 | Non-Toxin | -0.31 | 0.16 | 971.05 |
| 1530 | GEPDNYYQ | -0.66 | Non-Toxin | -0.32 | 0.32 | 985.08 |
| 1531 | GEPDQFFS | -0.84 | Non-Toxin | -0.12 | 0.32 | 926.05 |
| 1532 | GEPDQFFT | -0.78 | Non-Toxin | -0.11 | 0.32 | 940.08 |
| 1533 | GEPDQFFC | -0.44 | Non-Toxin | -0.09 | 0.32 | 942.11 |
| 1534 | GEPDQFFN | -0.75 | Non-Toxin | -0.17 | 0.32 | 953.08 |
| 1535 | GEPDQFFQ | -0.83 | Non-Toxin | -0.18 | 0.47 | 967.11 |
| 1536 | GEPDQFWS | -0.96 | Non-Toxin | -0.15 | 0.32 | 965.09 |
| 1537 | GEPDQFWT | -0.91 | Non-Toxin | -0.14 | 0.32 | 979.12 |
| 1538 | GEPDQFWC | -0.37 | Non-Toxin | -0.12 | 0.32 | 981.15 |
| 1539 | GEPDQFWN | -1.02 | Non-Toxin | -0.2 | 0.32 | 992.12 |
| 1540 | GEPDQFWQ | -0.92 | Non-Toxin | -0.21 | 0.47 | 1006.15 |
| 1541 | GEPDQFYS | -1.04 | Non-Toxin | -0.2 | 0.32 | 942.05 |
| 1542 | GEPDQFYT | -1.06 | Non-Toxin | -0.19 | 0.32 | 956.08 |
| 1543 | GEPDQFYC | -0.88 | Non-Toxin | -0.16 | 0.32 | 958.11 |
| 1544 | GEPDQFYN | -1.06 | Non-Toxin | -0.24 | 0.32 | 969.08 |
| 1545 | GEPDQFYQ | -0.79 | Non-Toxin | -0.25 | 0.47 | 983.11 |
| 1546 | GEPDQWFS | -0.91 | Non-Toxin | -0.15 | 0.32 | 965.09 |
| 1547 | GEPDQWFT | -0.87 | Non-Toxin | -0.14 | 0.32 | 979.12 |
| 1548 | GEPDQWFC | -0.54 | Non-Toxin | -0.11 | 0.32 | 981.15 |
| 1549 | GEPDQWFN | -0.86 | Non-Toxin | -0.2 | 0.32 | 992.12 |
| 1550 | GEPDQWFQ | -0.9 | Non-Toxin | -0.21 | 0.47 | 1006.15 |
| 1551 | GEPDQWWS | -0.88 | Non-Toxin | -0.18 | 0.32 | 1004.13 |
| 1552 | GEPDQWWT | -0.84 | Non-Toxin | -0.17 | 0.32 | 1018.16 |
| 1553 | GEPDQWWC | -0.29 | Non-Toxin | -0.14 | 0.32 | 1020.19 |
| 1554 | GEPDQWWN | -0.94 | Non-Toxin | -0.23 | 0.32 | 1031.16 |
| 1555 | GEPDQWWQ | -0.83 | Non-Toxin | -0.24 | 0.47 | 1045.19 |
| 1556 | GEPDQWYS | -0.71 | Non-Toxin | -0.23 | 0.32 | 981.09 |
| 1557 | GEPDQWYT | -0.75 | Non-Toxin | -0.22 | 0.32 | 995.12 |
| 1558 | GEPDQWYC | -0.51 | Non-Toxin | -0.19 | 0.32 | 997.15 |
| 1559 | GEPDQWYN | -0.72 | Non-Toxin | -0.27 | 0.32 | 1008.12 |
| 1560 | GEPDQWYQ | -0.48 | Non-Toxin | -0.28 | 0.47 | 1022.15 |
| 1561 | GEPDQYFS | -1.03 | Non-Toxin | -0.2 | 0.32 | 942.05 |
| 1562 | GEPDQYFT | -0.97 | Non-Toxin | -0.19 | 0.32 | 956.08 |
| 1563 | GEPDQYFC | -0.67 | Non-Toxin | -0.16 | 0.32 | 958.11 |
| 1564 | GEPDQYFN | -1 | Non-Toxin | -0.24 | 0.32 | 969.08 |
| 1565 | GEPDQYFQ | -1.04 | Non-Toxin | -0.25 | 0.47 | 983.11 |
| 1566 | GEPDQYWS | -1.07 | Non-Toxin | -0.23 | 0.32 | 981.09 |
| 1567 | GEPDQYWT | -1.02 | Non-Toxin | -0.22 | 0.32 | 995.12 |
| 1568 | GEPDQYWC | -0.47 | Non-Toxin | -0.19 | 0.32 | 997.15 |
| 1569 | GEPDQYWN | -1.13 | Non-Toxin | -0.27 | 0.32 | 1008.12 |
| 1570 | GEPDQYWQ | -1.01 | Non-Toxin | -0.28 | 0.47 | 1022.15 |
| 1571 | GEPDQYYS | -0.94 | Non-Toxin | -0.27 | 0.32 | 958.05 |
| 1572 | GEPDQYYT | -0.96 | Non-Toxin | -0.26 | 0.32 | 972.08 |
| 1573 | GEPDQYYC | -0.69 | Non-Toxin | -0.23 | 0.32 | 974.11 |
| 1574 | GEPDQYYN | -0.94 | Non-Toxin | -0.32 | 0.32 | 985.08 |
| 1575 | GEPDQYYQ | -0.71 | Non-Toxin | -0.32 | 0.47 | 999.11 |
| 1576 | GEPESFFS | -0.9 | Non-Toxin | -0.06 | 0.32 | 899.02 |
| 1577 | GEPESFFT | -0.87 | Non-Toxin | -0.05 | 0.32 | 913.05 |
| 1578 | GEPESFFC | -0.52 | Non-Toxin | -0.02 | 0.32 | 915.08 |
| 1579 | GEPESFFN | -0.89 | Non-Toxin | -0.1 | 0.32 | 926.05 |
| 1580 | GEPESFFQ | -0.82 | Non-Toxin | -0.11 | 0.47 | 940.08 |
| 1581 | GEPESFWS | -1.09 | Non-Toxin | -0.09 | 0.32 | 938.06 |
| 1582 | GEPESFWT | -0.97 | Non-Toxin | -0.08 | 0.32 | 952.09 |
| 1583 | GEPESFWC | -0.44 | Non-Toxin | -0.05 | 0.32 | 954.12 |
| 1584 | GEPESFWN | -1.1 | Non-Toxin | -0.13 | 0.32 | 965.09 |
| 1585 | GEPESFWQ | -0.99 | Non-Toxin | -0.14 | 0.47 | 979.12 |
| 1586 | GEPESFYS | -1.22 | Non-Toxin | -0.13 | 0.32 | 915.02 |
| 1587 | GEPESFYT | -1.21 | Non-Toxin | -0.12 | 0.32 | 929.05 |
| 1588 | GEPESFYC | -1.02 | Non-Toxin | -0.09 | 0.32 | 931.08 |
| 1589 | GEPESFYN | -1.17 | Non-Toxin | -0.18 | 0.32 | 942.05 |
| 1590 | GEPESFYQ | -0.92 | Non-Toxin | -0.18 | 0.47 | 956.08 |
| 1591 | GEPESWFS | -1.23 | Non-Toxin | -0.09 | 0.32 | 938.06 |
| 1592 | GEPESWFT | -1.2 | Non-Toxin | -0.08 | 0.32 | 952.09 |
| 1593 | GEPESWFC | -0.84 | Non-Toxin | -0.05 | 0.32 | 954.12 |
| 1594 | GEPESWFN | -1.18 | Non-Toxin | -0.13 | 0.32 | 965.09 |
| 1595 | GEPESWFQ | -1.17 | Non-Toxin | -0.14 | 0.47 | 979.12 |
| 1596 | GEPESWWS | -1.21 | Non-Toxin | -0.12 | 0.32 | 977.1 |
| 1597 | GEPESWWT | -1.14 | Non-Toxin | -0.11 | 0.32 | 991.13 |
| 1598 | GEPESWWC | -0.61 | Non-Toxin | -0.08 | 0.32 | 993.16 |
| 1599 | GEPESWWN | -1.25 | Non-Toxin | -0.16 | 0.32 | 1004.13 |
| 1600 | GEPESWWQ | -1.13 | Non-Toxin | -0.17 | 0.47 | 1018.16 |
| 1601 | GEPESWYS | -1.05 | Non-Toxin | -0.16 | 0.32 | 954.06 |
| 1602 | GEPESWYT | -1.07 | Non-Toxin | -0.15 | 0.32 | 968.09 |
| 1603 | GEPESWYC | -0.8 | Non-Toxin | -0.12 | 0.32 | 970.12 |
| 1604 | GEPESWYN | -1.01 | Non-Toxin | -0.21 | 0.32 | 981.09 |
| 1605 | GEPESWYQ | -0.74 | Non-Toxin | -0.21 | 0.47 | 995.12 |
| 1606 | GEPESYFS | -1.11 | Non-Toxin | -0.13 | 0.32 | 915.02 |
| 1607 | GEPESYFT | -1.07 | Non-Toxin | -0.12 | 0.32 | 929.05 |
| 1608 | GEPESYFC | -0.73 | Non-Toxin | -0.09 | 0.32 | 931.08 |
| 1609 | GEPESYFN | -1.11 | Non-Toxin | -0.18 | 0.32 | 942.05 |
| 1610 | GEPESYFQ | -1.08 | Non-Toxin | -0.18 | 0.47 | 956.08 |
| 1611 | GEPESYWS | -1.18 | Non-Toxin | -0.16 | 0.32 | 954.06 |
| 1612 | GEPESYWT | -1.13 | Non-Toxin | -0.15 | 0.32 | 968.09 |
| 1613 | GEPESYWC | -0.59 | Non-Toxin | -0.12 | 0.32 | 970.12 |
| 1614 | GEPESYWN | -1.23 | Non-Toxin | -0.21 | 0.32 | 981.09 |
| 1615 | GEPESYWQ | -1.11 | Non-Toxin | -0.21 | 0.47 | 995.12 |
| 1616 | GEPESYYS | -1.03 | Non-Toxin | -0.2 | 0.32 | 931.02 |
| 1617 | GEPESYYT | -1.06 | Non-Toxin | -0.19 | 0.32 | 945.05 |
| 1618 | GEPESYYC | -0.72 | Non-Toxin | -0.17 | 0.32 | 947.08 |
| 1619 | GEPESYYN | -1 | Non-Toxin | -0.25 | 0.32 | 958.05 |
| 1620 | GEPESYYQ | -0.72 | Non-Toxin | -0.26 | 0.47 | 972.08 |
| 1621 | GEPETFFS | -1.27 | Non-Toxin | -0.05 | 0.32 | 913.05 |
| 1622 | GEPETFFT | -1.27 | Non-Toxin | -0.04 | 0.32 | 927.08 |
| 1623 | GEPETFFC | -0.89 | Non-Toxin | -0.01 | 0.32 | 929.11 |
| 1624 | GEPETFFN | -1.19 | Non-Toxin | -0.09 | 0.32 | 940.08 |
| 1625 | GEPETFFQ | -1.24 | Non-Toxin | -0.1 | 0.47 | 954.11 |
| 1626 | GEPETFWS | -1.39 | Non-Toxin | -0.08 | 0.32 | 952.09 |
| 1627 | GEPETFWT | -1.31 | Non-Toxin | -0.07 | 0.32 | 966.12 |
| 1628 | GEPETFWC | -0.79 | Non-Toxin | -0.04 | 0.32 | 968.15 |
| 1629 | GEPETFWN | -1.44 | Non-Toxin | -0.12 | 0.32 | 979.12 |
| 1630 | GEPETFWQ | -1.33 | Non-Toxin | -0.13 | 0.47 | 993.15 |
| 1631 | GEPETFYS | -1.53 | Non-Toxin | -0.12 | 0.32 | 929.05 |
| 1632 | GEPETFYT | -1.52 | Non-Toxin | -0.11 | 0.32 | 943.08 |
| 1633 | GEPETFYC | -1.33 | Non-Toxin | -0.08 | 0.32 | 945.11 |
| 1634 | GEPETFYN | -1.49 | Non-Toxin | -0.17 | 0.32 | 956.08 |
| 1635 | GEPETFYQ | -1.25 | Non-Toxin | -0.17 | 0.47 | 970.11 |
| 1636 | GEPETWFS | -1.25 | Non-Toxin | -0.08 | 0.32 | 952.09 |
| 1637 | GEPETWFT | -1.25 | Non-Toxin | -0.07 | 0.32 | 966.12 |
| 1638 | GEPETWFC | -0.89 | Non-Toxin | -0.04 | 0.32 | 968.15 |
| 1639 | GEPETWFN | -1.2 | Non-Toxin | -0.12 | 0.32 | 979.12 |
| 1640 | GEPETWFQ | -1.22 | Non-Toxin | -0.13 | 0.47 | 993.15 |
| 1641 | GEPETWWS | -1.24 | Non-Toxin | -0.11 | 0.32 | 991.13 |
| 1642 | GEPETWWT | -1.19 | Non-Toxin | -0.1 | 0.32 | 1005.16 |
| 1643 | GEPETWWC | -0.62 | Non-Toxin | -0.07 | 0.32 | 1007.19 |
| 1644 | GEPETWWN | -1.29 | Non-Toxin | -0.15 | 0.32 | 1018.16 |
| 1645 | GEPETWWQ | -1.17 | Non-Toxin | -0.16 | 0.47 | 1032.19 |
| 1646 | GEPETWYS | -1.12 | Non-Toxin | -0.15 | 0.32 | 968.09 |
| 1647 | GEPETWYT | -1.11 | Non-Toxin | -0.14 | 0.32 | 982.12 |
| 1648 | GEPETWYC | -0.86 | Non-Toxin | -0.11 | 0.32 | 984.15 |
| 1649 | GEPETWYN | -1.07 | Non-Toxin | -0.2 | 0.32 | 995.12 |
| 1650 | GEPETWYQ | -0.81 | Non-Toxin | -0.2 | 0.47 | 1009.15 |
| 1651 | GEPETYFS | -1.13 | Non-Toxin | -0.12 | 0.32 | 929.05 |
| 1652 | GEPETYFT | -1.11 | Non-Toxin | -0.11 | 0.32 | 943.08 |
| 1653 | GEPETYFC | -0.78 | Non-Toxin | -0.08 | 0.32 | 945.11 |
| 1654 | GEPETYFN | -1.13 | Non-Toxin | -0.17 | 0.32 | 956.08 |
| 1655 | GEPETYFQ | -1.12 | Non-Toxin | -0.17 | 0.47 | 970.11 |
| 1656 | GEPETYWS | -1.21 | Non-Toxin | -0.15 | 0.32 | 968.09 |
| 1657 | GEPETYWT | -1.16 | Non-Toxin | -0.14 | 0.32 | 982.12 |
| 1658 | GEPETYWC | -0.61 | Non-Toxin | -0.11 | 0.32 | 984.15 |
| 1659 | GEPETYWN | -1.27 | Non-Toxin | -0.2 | 0.32 | 995.12 |
| 1660 | GEPETYWQ | -1.15 | Non-Toxin | -0.2 | 0.47 | 1009.15 |
| 1661 | GEPETYYS | -1.12 | Non-Toxin | -0.19 | 0.32 | 945.05 |
| 1662 | GEPETYYT | -1.08 | Non-Toxin | -0.18 | 0.32 | 959.08 |
| 1663 | GEPETYYC | -0.81 | Non-Toxin | -0.16 | 0.32 | 961.11 |
| 1664 | GEPETYYN | -1.07 | Non-Toxin | -0.24 | 0.32 | 972.08 |
| 1665 | GEPETYYQ | -0.8 | Non-Toxin | -0.25 | 0.47 | 986.11 |
| 1666 | GEPECFFS | -0.64 | Non-Toxin | -0.02 | 0.32 | 915.08 |
| 1667 | GEPECFFT | -0.57 | Non-Toxin | -0.01 | 0.32 | 929.11 |
| 1668 | GEPECFFC | -0.17 | Non-Toxin | 0.02 | 0.32 | 931.14 |
| 1669 | GEPECFFN | -0.57 | Non-Toxin | -0.07 | 0.32 | 942.11 |
| 1670 | GEPECFFQ | -0.6 | Non-Toxin | -0.07 | 0.47 | 956.14 |
| 1671 | GEPECFWS | -0.9 | Non-Toxin | -0.05 | 0.32 | 954.12 |
| 1672 | GEPECFWT | -0.85 | Non-Toxin | -0.04 | 0.32 | 968.15 |
| 1673 | GEPECFWC | -0.3 | Non-Toxin | -0.01 | 0.32 | 970.18 |
| 1674 | GEPECFWN | -0.95 | Non-Toxin | -0.1 | 0.32 | 981.15 |
| 1675 | GEPECFWQ | -0.83 | Non-Toxin | -0.1 | 0.47 | 995.18 |
| 1676 | GEPECFYS | -0.93 | Non-Toxin | -0.09 | 0.32 | 931.08 |
| 1677 | GEPECFYT | -0.92 | Non-Toxin | -0.08 | 0.32 | 945.11 |
| 1678 | GEPECFYC | -0.82 | Non-Toxin | -0.05 | 0.32 | 947.14 |
| 1679 | GEPECFYN | -0.89 | Non-Toxin | -0.14 | 0.32 | 958.11 |
| 1680 | GEPECFYQ | -0.65 | Non-Toxin | -0.15 | 0.47 | 972.14 |
| 1681 | GEPECWFS | -0.6 | Non-Toxin | -0.05 | 0.32 | 954.12 |
| 1682 | GEPECWFT | -0.53 | Non-Toxin | -0.04 | 0.32 | 968.15 |
| 1683 | GEPECWFC | -0.18 | Non-Toxin | -0.01 | 0.32 | 970.18 |
| 1684 | GEPECWFN | -0.51 | Non-Toxin | -0.1 | 0.32 | 981.15 |
| 1685 | GEPECWFQ | -0.55 | Non-Toxin | -0.1 | 0.47 | 995.18 |
| 1686 | GEPECWWS | -0.54 | Non-Toxin | -0.08 | 0.32 | 993.16 |
| 1687 | GEPECWWT | -0.51 | Non-Toxin | -0.07 | 0.32 | 1007.19 |
| 1688 | GEPECWWC | 0.06 | Toxin | -0.04 | 0.32 | 1009.22 |
| 1689 | GEPECWWN | -0.6 | Non-Toxin | -0.13 | 0.32 | 1020.19 |
| 1690 | GEPECWWQ | -0.49 | Non-Toxin | -0.13 | 0.47 | 1034.22 |
| 1691 | GEPECWYS | -0.43 | Non-Toxin | -0.12 | 0.32 | 970.12 |
| 1692 | GEPECWYT | -0.38 | Non-Toxin | -0.11 | 0.32 | 984.15 |
| 1693 | GEPECWYC | -0.18 | Non-Toxin | -0.08 | 0.32 | 986.18 |
| 1694 | GEPECWYN | -0.37 | Non-Toxin | -0.17 | 0.32 | 997.15 |
| 1695 | GEPECWYQ | -0.11 | Non-Toxin | -0.18 | 0.47 | 1011.18 |
| 1696 | GEPECYFS | -0.57 | Non-Toxin | -0.09 | 0.32 | 931.08 |
| 1697 | GEPECYFT | -0.49 | Non-Toxin | -0.08 | 0.32 | 945.11 |
| 1698 | GEPECYFC | -0.18 | Non-Toxin | -0.05 | 0.32 | 947.14 |
| 1699 | GEPECYFN | -0.51 | Non-Toxin | -0.14 | 0.32 | 958.11 |
| 1700 | GEPECYFQ | -0.54 | Non-Toxin | -0.15 | 0.47 | 972.14 |
| 1701 | GEPECYWS | -0.53 | Non-Toxin | -0.12 | 0.32 | 970.12 |
| 1702 | GEPECYWT | -0.5 | Non-Toxin | -0.11 | 0.32 | 984.15 |
| 1703 | GEPECYWC | 0.02 | Toxin | -0.08 | 0.32 | 986.18 |
| 1704 | GEPECYWN | -0.62 | Non-Toxin | -0.17 | 0.32 | 997.15 |
| 1705 | GEPECYWQ | -0.49 | Non-Toxin | -0.18 | 0.47 | 1011.18 |
| 1706 | GEPECYYS | -0.43 | Non-Toxin | -0.17 | 0.32 | 947.08 |
| 1707 | GEPECYYT | -0.44 | Non-Toxin | -0.16 | 0.32 | 961.11 |
| 1708 | GEPECYYC | -0.18 | Non-Toxin | -0.13 | 0.32 | 963.14 |
| 1709 | GEPECYYN | -0.37 | Non-Toxin | -0.21 | 0.32 | 974.11 |
| 1710 | GEPECYYQ | -0.14 | Non-Toxin | -0.22 | 0.47 | 988.14 |
| 1711 | GEPENFFS | -1.22 | Non-Toxin | -0.1 | 0.32 | 926.05 |
| 1712 | GEPENFFT | -1.14 | Non-Toxin | -0.09 | 0.32 | 940.08 |
| 1713 | GEPENFFC | -0.78 | Non-Toxin | -0.07 | 0.32 | 942.11 |
| 1714 | GEPENFFN | -1.12 | Non-Toxin | -0.15 | 0.32 | 953.08 |
| 1715 | GEPENFFQ | -1.14 | Non-Toxin | -0.16 | 0.47 | 967.11 |
| 1716 | GEPENFWS | -1.33 | Non-Toxin | -0.13 | 0.32 | 965.09 |
| 1717 | GEPENFWT | -1.27 | Non-Toxin | -0.12 | 0.32 | 979.12 |
| 1718 | GEPENFWC | -0.76 | Non-Toxin | -0.1 | 0.32 | 981.15 |
| 1719 | GEPENFWN | -1.38 | Non-Toxin | -0.18 | 0.32 | 992.12 |
| 1720 | GEPENFWQ | -1.27 | Non-Toxin | -0.19 | 0.47 | 1006.15 |
| 1721 | GEPENFYS | -1.42 | Non-Toxin | -0.18 | 0.32 | 942.05 |
| 1722 | GEPENFYT | -1.4 | Non-Toxin | -0.17 | 0.32 | 956.08 |
| 1723 | GEPENFYC | -1.22 | Non-Toxin | -0.14 | 0.32 | 958.11 |
| 1724 | GEPENFYN | -1.4 | Non-Toxin | -0.23 | 0.32 | 969.08 |
| 1725 | GEPENFYQ | -1.16 | Non-Toxin | -0.23 | 0.47 | 983.11 |
| 1726 | GEPENWFS | -1.11 | Non-Toxin | -0.13 | 0.32 | 965.09 |
| 1727 | GEPENWFT | -1.06 | Non-Toxin | -0.12 | 0.32 | 979.12 |
| 1728 | GEPENWFC | -0.71 | Non-Toxin | -0.1 | 0.32 | 981.15 |
| 1729 | GEPENWFN | -1.04 | Non-Toxin | -0.18 | 0.32 | 992.12 |
| 1730 | GEPENWFQ | -1.04 | Non-Toxin | -0.19 | 0.47 | 1006.15 |
| 1731 | GEPENWWS | -1.06 | Non-Toxin | -0.16 | 0.32 | 1004.13 |
| 1732 | GEPENWWT | -1.02 | Non-Toxin | -0.15 | 0.32 | 1018.16 |
| 1733 | GEPENWWC | -0.49 | Non-Toxin | -0.13 | 0.32 | 1020.19 |
| 1734 | GEPENWWN | -1.12 | Non-Toxin | -0.21 | 0.32 | 1031.16 |
| 1735 | GEPENWWQ | -1.01 | Non-Toxin | -0.22 | 0.47 | 1045.19 |
| 1736 | GEPENWYS | -0.93 | Non-Toxin | -0.21 | 0.32 | 981.09 |
| 1737 | GEPENWYT | -0.93 | Non-Toxin | -0.2 | 0.32 | 995.12 |
| 1738 | GEPENWYC | -0.68 | Non-Toxin | -0.17 | 0.32 | 997.15 |
| 1739 | GEPENWYN | -0.9 | Non-Toxin | -0.26 | 0.32 | 1008.12 |
| 1740 | GEPENWYQ | -0.64 | Non-Toxin | -0.26 | 0.47 | 1022.15 |
| 1741 | GEPENYFS | -0.99 | Non-Toxin | -0.18 | 0.32 | 942.05 |
| 1742 | GEPENYFT | -0.94 | Non-Toxin | -0.17 | 0.32 | 956.08 |
| 1743 | GEPENYFC | -0.6 | Non-Toxin | -0.14 | 0.32 | 958.11 |
| 1744 | GEPENYFN | -0.96 | Non-Toxin | -0.23 | 0.32 | 969.08 |
| 1745 | GEPENYFQ | -0.94 | Non-Toxin | -0.23 | 0.47 | 983.11 |
| 1746 | GEPENYWS | -1.05 | Non-Toxin | -0.21 | 0.32 | 981.09 |
| 1747 | GEPENYWT | -1.01 | Non-Toxin | -0.2 | 0.32 | 995.12 |
| 1748 | GEPENYWC | -0.47 | Non-Toxin | -0.17 | 0.32 | 997.15 |
| 1749 | GEPENYWN | -1.13 | Non-Toxin | -0.26 | 0.32 | 1008.12 |
| 1750 | GEPENYWQ | -1 | Non-Toxin | -0.26 | 0.47 | 1022.15 |
| 1751 | GEPENYYS | -0.92 | Non-Toxin | -0.25 | 0.32 | 958.05 |
| 1752 | GEPENYYT | -0.88 | Non-Toxin | -0.24 | 0.32 | 972.08 |
| 1753 | GEPENYYC | -0.55 | Non-Toxin | -0.21 | 0.32 | 974.11 |
| 1754 | GEPENYYN | -0.91 | Non-Toxin | -0.3 | 0.32 | 985.08 |
| 1755 | GEPENYYQ | -0.63 | Non-Toxin | -0.3 | 0.47 | 999.11 |
| 1756 | GEPEQFFS | -1.02 | Non-Toxin | -0.11 | 0.47 | 940.08 |
| 1757 | GEPEQFFT | -0.93 | Non-Toxin | -0.1 | 0.47 | 954.11 |
| 1758 | GEPEQFFC | -0.58 | Non-Toxin | -0.07 | 0.47 | 956.14 |
| 1759 | GEPEQFFN | -0.92 | Non-Toxin | -0.16 | 0.47 | 967.11 |
| 1760 | GEPEQFFQ | -0.96 | Non-Toxin | -0.16 | 0.63 | 981.14 |
| 1761 | GEPEQFWS | -1.15 | Non-Toxin | -0.14 | 0.47 | 979.12 |
| 1762 | GEPEQFWT | -1.07 | Non-Toxin | -0.13 | 0.47 | 993.15 |
| 1763 | GEPEQFWC | -0.55 | Non-Toxin | -0.1 | 0.47 | 995.18 |
| 1764 | GEPEQFWN | -1.18 | Non-Toxin | -0.19 | 0.47 | 1006.15 |
| 1765 | GEPEQFWQ | -1.08 | Non-Toxin | -0.19 | 0.63 | 1020.18 |
| 1766 | GEPEQFYS | -1.26 | Non-Toxin | -0.18 | 0.47 | 956.08 |
| 1767 | GEPEQFYT | -1.22 | Non-Toxin | -0.17 | 0.47 | 970.11 |
| 1768 | GEPEQFYC | -1.04 | Non-Toxin | -0.15 | 0.47 | 972.14 |
| 1769 | GEPEQFYN | -1.22 | Non-Toxin | -0.23 | 0.47 | 983.11 |
| 1770 | GEPEQFYQ | -0.96 | Non-Toxin | -0.24 | 0.63 | 997.14 |
| 1771 | GEPEQWFS | -1.12 | Non-Toxin | -0.14 | 0.47 | 979.12 |
| 1772 | GEPEQWFT | -1.06 | Non-Toxin | -0.13 | 0.47 | 993.15 |
| 1773 | GEPEQWFC | -0.71 | Non-Toxin | -0.1 | 0.47 | 995.18 |
| 1774 | GEPEQWFN | -1.05 | Non-Toxin | -0.19 | 0.47 | 1006.15 |
| 1775 | GEPEQWFQ | -1.06 | Non-Toxin | -0.19 | 0.63 | 1020.18 |
| 1776 | GEPEQWWS | -1.1 | Non-Toxin | -0.17 | 0.47 | 1018.16 |
| 1777 | GEPEQWWT | -1.03 | Non-Toxin | -0.16 | 0.47 | 1032.19 |
| 1778 | GEPEQWWC | -0.51 | Non-Toxin | -0.13 | 0.47 | 1034.22 |
| 1779 | GEPEQWWN | -1.14 | Non-Toxin | -0.22 | 0.47 | 1045.19 |
| 1780 | GEPEQWWQ | -1.03 | Non-Toxin | -0.22 | 0.63 | 1059.22 |
| 1781 | GEPEQWYS | -0.95 | Non-Toxin | -0.21 | 0.47 | 995.12 |
| 1782 | GEPEQWYT | -0.94 | Non-Toxin | -0.2 | 0.47 | 1009.15 |
| 1783 | GEPEQWYC | -0.69 | Non-Toxin | -0.18 | 0.47 | 1011.18 |
| 1784 | GEPEQWYN | -0.9 | Non-Toxin | -0.26 | 0.47 | 1022.15 |
| 1785 | GEPEQWYQ | -0.67 | Non-Toxin | -0.27 | 0.63 | 1036.18 |
| 1786 | GEPEQYFS | -1.24 | Non-Toxin | -0.18 | 0.47 | 956.08 |
| 1787 | GEPEQYFT | -1.15 | Non-Toxin | -0.17 | 0.47 | 970.11 |
| 1788 | GEPEQYFC | -0.84 | Non-Toxin | -0.15 | 0.47 | 972.14 |
| 1789 | GEPEQYFN | -1.18 | Non-Toxin | -0.23 | 0.47 | 983.11 |
| 1790 | GEPEQYFQ | -1.19 | Non-Toxin | -0.24 | 0.63 | 997.14 |
| 1791 | GEPEQYWS | -1.28 | Non-Toxin | -0.21 | 0.47 | 995.12 |
| 1792 | GEPEQYWT | -1.21 | Non-Toxin | -0.2 | 0.47 | 1009.15 |
| 1793 | GEPEQYWC | -0.68 | Non-Toxin | -0.18 | 0.47 | 1011.18 |
| 1794 | GEPEQYWN | -1.32 | Non-Toxin | -0.26 | 0.47 | 1022.15 |
| 1795 | GEPEQYWQ | -1.2 | Non-Toxin | -0.27 | 0.63 | 1036.18 |
| 1796 | GEPEQYYS | -1.17 | Non-Toxin | -0.26 | 0.47 | 972.08 |
| 1797 | GEPEQYYT | -1.14 | Non-Toxin | -0.25 | 0.47 | 986.11 |
| 1798 | GEPEQYYC | -0.86 | Non-Toxin | -0.22 | 0.47 | 988.14 |
| 1799 | GEPEQYYN | -1.12 | Non-Toxin | -0.3 | 0.47 | 999.11 |
| 1800 | GEPEQYYQ | -0.89 | Non-Toxin | -0.31 | 0.63 | 1013.14 |
| 1801 | PDGDSFFS | -0.89 | Non-Toxin | -0.08 | 0 | 870.96 |
| 1802 | PDGDSFFT | -0.82 | Non-Toxin | -0.07 | 0 | 884.99 |
| 1803 | PDGDSFFC | -0.54 | Non-Toxin | -0.04 | 0 | 887.02 |
| 1804 | PDGDSFFN | -0.86 | Non-Toxin | -0.13 | 0 | 897.99 |
| 1805 | PDGDSFFQ | -0.89 | Non-Toxin | -0.14 | 0.16 | 912.02 |
| 1806 | PDGDSFWS | -1.06 | Non-Toxin | -0.11 | 0 | 910 |
| 1807 | PDGDSFWT | -0.95 | Non-Toxin | -0.1 | 0 | 924.03 |
| 1808 | PDGDSFWC | -0.41 | Non-Toxin | -0.07 | 0 | 926.06 |
| 1809 | PDGDSFWN | -1.06 | Non-Toxin | -0.16 | 0 | 937.03 |
| 1810 | PDGDSFWQ | -0.95 | Non-Toxin | -0.16 | 0.16 | 951.06 |
| 1811 | PDGDSFYS | -1.15 | Non-Toxin | -0.15 | 0 | 886.96 |
| 1812 | PDGDSFYT | -1.11 | Non-Toxin | -0.14 | 0 | 900.99 |
| 1813 | PDGDSFYC | -0.98 | Non-Toxin | -0.12 | 0 | 903.02 |
| 1814 | PDGDSFYN | -1.12 | Non-Toxin | -0.2 | 0 | 913.99 |
| 1815 | PDGDSFYQ | -0.94 | Non-Toxin | -0.21 | 0.16 | 928.02 |
| 1816 | PDGDSWFS | -1.17 | Non-Toxin | -0.11 | 0 | 910 |
| 1817 | PDGDSWFT | -1.1 | Non-Toxin | -0.1 | 0 | 924.03 |
| 1818 | PDGDSWFC | -0.81 | Non-Toxin | -0.07 | 0 | 926.06 |
| 1819 | PDGDSWFN | -1.1 | Non-Toxin | -0.16 | 0 | 937.03 |
| 1820 | PDGDSWFQ | -1.17 | Non-Toxin | -0.17 | 0.16 | 951.06 |
| 1821 | PDGDSWWS | -1.11 | Non-Toxin | -0.14 | 0 | 949.04 |
| 1822 | PDGDSWWT | -1.05 | Non-Toxin | -0.13 | 0 | 963.07 |
| 1823 | PDGDSWWC | -0.51 | Non-Toxin | -0.1 | 0 | 965.1 |
| 1824 | PDGDSWWN | -1.14 | Non-Toxin | -0.19 | 0 | 976.07 |
| 1825 | PDGDSWWQ | -1.03 | Non-Toxin | -0.2 | 0.16 | 990.1 |
| 1826 | PDGDSWYS | -0.92 | Non-Toxin | -0.19 | 0 | 926 |
| 1827 | PDGDSWYT | -0.91 | Non-Toxin | -0.18 | 0 | 940.03 |
| 1828 | PDGDSWYC | -0.71 | Non-Toxin | -0.15 | 0 | 942.06 |
| 1829 | PDGDSWYN | -0.9 | Non-Toxin | -0.23 | 0 | 953.03 |
| 1830 | PDGDSWYQ | -0.71 | Non-Toxin | -0.24 | 0.16 | 967.06 |
| 1831 | PDGDSYFS | -1 | Non-Toxin | -0.16 | 0 | 886.96 |
| 1832 | PDGDSYFT | -0.91 | Non-Toxin | -0.15 | 0 | 900.99 |
| 1833 | PDGDSYFC | -0.64 | Non-Toxin | -0.12 | 0 | 903.02 |
| 1834 | PDGDSYFN | -0.96 | Non-Toxin | -0.2 | 0 | 913.99 |
| 1835 | PDGDSYFQ | -1.02 | Non-Toxin | -0.21 | 0.16 | 928.02 |
| 1836 | PDGDSYWS | -1.06 | Non-Toxin | -0.19 | 0 | 926 |
| 1837 | PDGDSYWT | -1.02 | Non-Toxin | -0.18 | 0 | 940.03 |
| 1838 | PDGDSYWC | -0.46 | Non-Toxin | -0.15 | 0 | 942.06 |
| 1839 | PDGDSYWN | -1.1 | Non-Toxin | -0.23 | 0 | 953.03 |
| 1840 | PDGDSYWQ | -0.98 | Non-Toxin | -0.24 | 0.16 | 967.06 |
| 1841 | PDGDSYYS | -0.9 | Non-Toxin | -0.23 | 0 | 902.96 |
| 1842 | PDGDSYYT | -0.89 | Non-Toxin | -0.22 | 0 | 916.99 |
| 1843 | PDGDSYYC | -0.62 | Non-Toxin | -0.19 | 0 | 919.02 |
| 1844 | PDGDSYYN | -0.88 | Non-Toxin | -0.28 | 0 | 929.99 |
| 1845 | PDGDSYYQ | -0.67 | Non-Toxin | -0.28 | 0.16 | 944.02 |
| 1846 | PDGDTFFS | -0.81 | Non-Toxin | -0.07 | 0 | 884.99 |
| 1847 | PDGDTFFT | -0.72 | Non-Toxin | -0.06 | 0 | 899.02 |
| 1848 | PDGDTFFC | -0.37 | Non-Toxin | -0.03 | 0 | 901.05 |
| 1849 | PDGDTFFN | -0.71 | Non-Toxin | -0.12 | 0 | 912.02 |
| 1850 | PDGDTFFQ | -0.82 | Non-Toxin | -0.12 | 0.16 | 926.05 |
| 1851 | PDGDTFWS | -0.92 | Non-Toxin | -0.1 | 0 | 924.03 |
| 1852 | PDGDTFWT | -0.85 | Non-Toxin | -0.09 | 0 | 938.06 |
| 1853 | PDGDTFWC | -0.34 | Non-Toxin | -0.06 | 0 | 940.09 |
| 1854 | PDGDTFWN | -0.96 | Non-Toxin | -0.15 | 0 | 951.06 |
| 1855 | PDGDTFWQ | -0.85 | Non-Toxin | -0.15 | 0.16 | 965.09 |
| 1856 | PDGDTFYS | -0.98 | Non-Toxin | -0.15 | 0 | 900.99 |
| 1857 | PDGDTFYT | -0.98 | Non-Toxin | -0.14 | 0 | 915.02 |
| 1858 | PDGDTFYC | -0.77 | Non-Toxin | -0.11 | 0 | 917.05 |
| 1859 | PDGDTFYN | -0.99 | Non-Toxin | -0.19 | 0 | 928.02 |
| 1860 | PDGDTFYQ | -0.8 | Non-Toxin | -0.2 | 0.16 | 942.05 |
| 1861 | PDGDTWFS | -0.88 | Non-Toxin | -0.1 | 0 | 924.03 |
| 1862 | PDGDTWFT | -0.79 | Non-Toxin | -0.09 | 0 | 938.06 |
| 1863 | PDGDTWFC | -0.46 | Non-Toxin | -0.06 | 0 | 940.09 |
| 1864 | PDGDTWFN | -0.8 | Non-Toxin | -0.15 | 0 | 951.06 |
| 1865 | PDGDTWFQ | -0.88 | Non-Toxin | -0.16 | 0.16 | 965.09 |
| 1866 | PDGDTWWS | -0.82 | Non-Toxin | -0.13 | 0 | 963.07 |
| 1867 | PDGDTWWT | -0.79 | Non-Toxin | -0.12 | 0 | 977.1 |
| 1868 | PDGDTWWC | -0.23 | Non-Toxin | -0.09 | 0 | 979.13 |
| 1869 | PDGDTWWN | -0.87 | Non-Toxin | -0.18 | 0 | 990.1 |
| 1870 | PDGDTWWQ | -0.75 | Non-Toxin | -0.18 | 0.16 | 1004.13 |
| 1871 | PDGDTWYS | -0.68 | Non-Toxin | -0.18 | 0 | 940.03 |
| 1872 | PDGDTWYT | -0.67 | Non-Toxin | -0.17 | 0 | 954.06 |
| 1873 | PDGDTWYC | -0.41 | Non-Toxin | -0.14 | 0 | 956.09 |
| 1874 | PDGDTWYN | -0.68 | Non-Toxin | -0.22 | 0 | 967.06 |
| 1875 | PDGDTWYQ | -0.47 | Non-Toxin | -0.23 | 0.16 | 981.09 |
| 1876 | PDGDTYFS | -0.74 | Non-Toxin | -0.15 | 0 | 900.99 |
| 1877 | PDGDTYFT | -0.63 | Non-Toxin | -0.14 | 0 | 915.02 |
| 1878 | PDGDTYFC | -0.34 | Non-Toxin | -0.11 | 0 | 917.05 |
| 1879 | PDGDTYFN | -0.72 | Non-Toxin | -0.19 | 0 | 928.02 |
| 1880 | PDGDTYFQ | -0.77 | Non-Toxin | -0.2 | 0.16 | 942.05 |
| 1881 | PDGDTYWS | -0.81 | Non-Toxin | -0.18 | 0 | 940.03 |
| 1882 | PDGDTYWT | -0.78 | Non-Toxin | -0.17 | 0 | 954.06 |
| 1883 | PDGDTYWC | -0.23 | Non-Toxin | -0.14 | 0 | 956.09 |
| 1884 | PDGDTYWN | -0.86 | Non-Toxin | -0.22 | 0 | 967.06 |
| 1885 | PDGDTYWQ | -0.74 | Non-Toxin | -0.23 | 0.16 | 981.09 |
| 1886 | PDGDTYYS | -0.72 | Non-Toxin | -0.22 | 0 | 916.99 |
| 1887 | PDGDTYYT | -0.67 | Non-Toxin | -0.21 | 0 | 931.02 |
| 1888 | PDGDTYYC | -0.41 | Non-Toxin | -0.18 | 0 | 933.05 |
| 1889 | PDGDTYYN | -0.71 | Non-Toxin | -0.27 | 0 | 944.02 |
| 1890 | PDGDTYYQ | -0.5 | Non-Toxin | -0.27 | 0.16 | 958.05 |
| 1891 | PDGDCFFS | -0.49 | Non-Toxin | -0.04 | 0 | 887.02 |
| 1892 | PDGDCFFT | -0.4 | Non-Toxin | -0.03 | 0 | 901.05 |
| 1893 | PDGDCFFC | -0.01 | Non-Toxin | -0.01 | 0 | 903.08 |
| 1894 | PDGDCFFN | -0.41 | Non-Toxin | -0.09 | 0 | 914.05 |
| 1895 | PDGDCFFQ | -0.51 | Non-Toxin | -0.1 | 0.16 | 928.08 |
| 1896 | PDGDCFWS | -0.73 | Non-Toxin | -0.07 | 0 | 926.06 |
| 1897 | PDGDCFWT | -0.7 | Non-Toxin | -0.06 | 0 | 940.09 |
| 1898 | PDGDCFWC | -0.16 | Non-Toxin | -0.04 | 0 | 942.12 |
| 1899 | PDGDCFWN | -0.78 | Non-Toxin | -0.12 | 0 | 953.09 |
| 1900 | PDGDCFWQ | -0.67 | Non-Toxin | -0.13 | 0.16 | 967.12 |
| 1901 | PDGDCFYS | -0.73 | Non-Toxin | -0.12 | 0 | 903.02 |
| 1902 | PDGDCFYT | -0.74 | Non-Toxin | -0.11 | 0 | 917.05 |
| 1903 | PDGDCFYC | -0.62 | Non-Toxin | -0.08 | 0 | 919.08 |
| 1904 | PDGDCFYN | -0.75 | Non-Toxin | -0.17 | 0 | 930.05 |
| 1905 | PDGDCFYQ | -0.56 | Non-Toxin | -0.17 | 0.16 | 944.08 |
| 1906 | PDGDCWFS | -0.46 | Non-Toxin | -0.07 | 0 | 926.06 |
| 1907 | PDGDCWFT | -0.37 | Non-Toxin | -0.06 | 0 | 940.09 |
| 1908 | PDGDCWFC | -0.02 | Non-Toxin | -0.04 | 0 | 942.12 |
| 1909 | PDGDCWFN | -0.37 | Non-Toxin | -0.12 | 0 | 953.09 |
| 1910 | PDGDCWFQ | -0.47 | Non-Toxin | -0.13 | 0.16 | 967.12 |
| 1911 | PDGDCWWS | -0.39 | Non-Toxin | -0.1 | 0 | 965.1 |
| 1912 | PDGDCWWT | -0.38 | Non-Toxin | -0.09 | 0 | 979.13 |
| 1913 | PDGDCWWC | 0.18 | Toxin | -0.07 | 0 | 981.16 |
| 1914 | PDGDCWWN | -0.45 | Non-Toxin | -0.15 | 0 | 992.13 |
| 1915 | PDGDCWWQ | -0.35 | Non-Toxin | -0.16 | 0.16 | 1006.16 |
| 1916 | PDGDCWYS | -0.27 | Non-Toxin | -0.15 | 0 | 942.06 |
| 1917 | PDGDCWYT | -0.25 | Non-Toxin | -0.14 | 0 | 956.09 |
| 1918 | PDGDCWYC | -0.03 | Non-Toxin | -0.11 | 0 | 958.12 |
| 1919 | PDGDCWYN | -0.27 | Non-Toxin | -0.2 | 0 | 969.09 |
| 1920 | PDGDCWYQ | -0.05 | Non-Toxin | -0.2 | 0.16 | 983.12 |
| 1921 | PDGDCYFS | -0.33 | Non-Toxin | -0.12 | 0 | 903.02 |
| 1922 | PDGDCYFT | -0.21 | Non-Toxin | -0.11 | 0 | 917.05 |
| 1923 | PDGDCYFC | 0.1 | Toxin | -0.08 | 0 | 919.08 |
| 1924 | PDGDCYFN | -0.25 | Non-Toxin | -0.17 | 0 | 930.05 |
| 1925 | PDGDCYFQ | -0.35 | Non-Toxin | -0.17 | 0.16 | 944.08 |
| 1926 | PDGDCYWS | -0.26 | Non-Toxin | -0.15 | 0 | 942.06 |
| 1927 | PDGDCYWT | -0.25 | Non-Toxin | -0.14 | 0 | 956.09 |
| 1928 | PDGDCYWC | 0.26 | Toxin | -0.11 | 0 | 958.12 |
| 1929 | PDGDCYWN | -0.35 | Non-Toxin | -0.2 | 0 | 969.09 |
| 1930 | PDGDCYWQ | -0.22 | Non-Toxin | -0.2 | 0.16 | 983.12 |
| 1931 | PDGDCYYS | -0.2 | Non-Toxin | -0.19 | 0 | 919.02 |
| 1932 | PDGDCYYT | -0.25 | Non-Toxin | -0.18 | 0 | 933.05 |
| 1933 | PDGDCYYC | 0.04 | Toxin | -0.15 | 0 | 935.08 |
| 1934 | PDGDCYYN | -0.2 | Non-Toxin | -0.24 | 0 | 946.05 |
| 1935 | PDGDCYYQ | -0.02 | Non-Toxin | -0.24 | 0.16 | 960.08 |
| 1936 | PDGDNFFS | -1.11 | Non-Toxin | -0.13 | 0 | 897.99 |
| 1937 | PDGDNFFT | -1.02 | Non-Toxin | -0.12 | 0 | 912.02 |
| 1938 | PDGDNFFC | -0.68 | Non-Toxin | -0.09 | 0 | 914.05 |
| 1939 | PDGDNFFN | -1.03 | Non-Toxin | -0.18 | 0 | 925.02 |
| 1940 | PDGDNFFQ | -1.12 | Non-Toxin | -0.18 | 0.16 | 939.05 |
| 1941 | PDGDNFWS | -1.23 | Non-Toxin | -0.16 | 0 | 937.03 |
| 1942 | PDGDNFWT | -1.18 | Non-Toxin | -0.15 | 0 | 951.06 |
| 1943 | PDGDNFWC | -0.67 | Non-Toxin | -0.12 | 0 | 953.09 |
| 1944 | PDGDNFWN | -1.28 | Non-Toxin | -0.21 | 0 | 964.06 |
| 1945 | PDGDNFWQ | -1.17 | Non-Toxin | -0.21 | 0.16 | 978.09 |
| 1946 | PDGDNFYS | -1.32 | Non-Toxin | -0.2 | 0 | 913.99 |
| 1947 | PDGDNFYT | -1.28 | Non-Toxin | -0.19 | 0 | 928.02 |
| 1948 | PDGDNFYC | -1.1 | Non-Toxin | -0.17 | 0 | 930.05 |
| 1949 | PDGDNFYN | -1.29 | Non-Toxin | -0.25 | 0 | 941.02 |
| 1950 | PDGDNFYQ | -1.12 | Non-Toxin | -0.26 | 0.16 | 955.05 |
| 1951 | PDGDNWFS | -0.99 | Non-Toxin | -0.16 | 0 | 937.03 |
| 1952 | PDGDNWFT | -0.92 | Non-Toxin | -0.15 | 0 | 951.06 |
| 1953 | PDGDNWFC | -0.59 | Non-Toxin | -0.12 | 0 | 953.09 |
| 1954 | PDGDNWFN | -0.93 | Non-Toxin | -0.21 | 0 | 964.06 |
| 1955 | PDGDNWFQ | -1 | Non-Toxin | -0.21 | 0.16 | 978.09 |
| 1956 | PDGDNWWS | -0.93 | Non-Toxin | -0.19 | 0 | 976.07 |
| 1957 | PDGDNWWT | -0.9 | Non-Toxin | -0.18 | 0 | 990.1 |
| 1958 | PDGDNWWC | -0.37 | Non-Toxin | -0.15 | 0 | 992.13 |
| 1959 | PDGDNWWN | -0.98 | Non-Toxin | -0.24 | 0 | 1003.1 |
| 1960 | PDGDNWWQ | -0.87 | Non-Toxin | -0.24 | 0.16 | 1017.13 |
| 1961 | PDGDNWYS | -0.81 | Non-Toxin | -0.23 | 0 | 953.03 |
| 1962 | PDGDNWYT | -0.8 | Non-Toxin | -0.22 | 0 | 967.06 |
| 1963 | PDGDNWYC | -0.55 | Non-Toxin | -0.2 | 0 | 969.09 |
| 1964 | PDGDNWYN | -0.78 | Non-Toxin | -0.28 | 0 | 980.06 |
| 1965 | PDGDNWYQ | -0.59 | Non-Toxin | -0.29 | 0.16 | 994.09 |
| 1966 | PDGDNYFS | -0.89 | Non-Toxin | -0.2 | 0 | 913.99 |
| 1967 | PDGDNYFT | -0.82 | Non-Toxin | -0.19 | 0 | 928.02 |
| 1968 | PDGDNYFC | -0.51 | Non-Toxin | -0.17 | 0 | 930.05 |
| 1969 | PDGDNYFN | -0.88 | Non-Toxin | -0.25 | 0 | 941.02 |
| 1970 | PDGDNYFQ | -0.93 | Non-Toxin | -0.26 | 0.16 | 955.05 |
| 1971 | PDGDNYWS | -0.97 | Non-Toxin | -0.23 | 0 | 953.03 |
| 1972 | PDGDNYWT | -0.94 | Non-Toxin | -0.22 | 0 | 967.06 |
| 1973 | PDGDNYWC | -0.4 | Non-Toxin | -0.2 | 0 | 969.09 |
| 1974 | PDGDNYWN | -1.04 | Non-Toxin | -0.28 | 0 | 980.06 |
| 1975 | PDGDNYWQ | -0.91 | Non-Toxin | -0.29 | 0.16 | 994.09 |
| 1976 | PDGDNYYS | -0.89 | Non-Toxin | -0.28 | 0 | 929.99 |
| 1977 | PDGDNYYT | -0.84 | Non-Toxin | -0.27 | 0 | 944.02 |
| 1978 | PDGDNYYC | -0.5 | Non-Toxin | -0.24 | 0 | 946.05 |
| 1979 | PDGDNYYN | -0.87 | Non-Toxin | -0.32 | 0 | 957.02 |
| 1980 | PDGDNYYQ | -0.66 | Non-Toxin | -0.33 | 0.16 | 971.05 |
| 1981 | PDGDQFFS | -0.71 | Non-Toxin | -0.14 | 0.16 | 912.02 |
| 1982 | PDGDQFFT | -0.62 | Non-Toxin | -0.13 | 0.16 | 926.05 |
| 1983 | PDGDQFFC | -0.28 | Non-Toxin | -0.1 | 0.16 | 928.08 |
| 1984 | PDGDQFFN | -0.61 | Non-Toxin | -0.18 | 0.16 | 939.05 |
| 1985 | PDGDQFFQ | -0.72 | Non-Toxin | -0.19 | 0.31 | 953.08 |
| 1986 | PDGDQFWS | -0.81 | Non-Toxin | -0.17 | 0.16 | 951.06 |
| 1987 | PDGDQFWT | -0.75 | Non-Toxin | -0.15 | 0.16 | 965.09 |
| 1988 | PDGDQFWC | -0.23 | Non-Toxin | -0.13 | 0.16 | 967.12 |
| 1989 | PDGDQFWN | -0.85 | Non-Toxin | -0.21 | 0.16 | 978.09 |
| 1990 | PDGDQFWQ | -0.75 | Non-Toxin | -0.22 | 0.31 | 992.12 |
| 1991 | PDGDQFYS | -0.85 | Non-Toxin | -0.21 | 0.16 | 928.02 |
| 1992 | PDGDQFYT | -0.83 | Non-Toxin | -0.2 | 0.16 | 942.05 |
| 1993 | PDGDQFYC | -0.63 | Non-Toxin | -0.17 | 0.16 | 944.08 |
| 1994 | PDGDQFYN | -0.87 | Non-Toxin | -0.26 | 0.16 | 955.05 |
| 1995 | PDGDQFYQ | -0.65 | Non-Toxin | -0.26 | 0.31 | 969.08 |
| 1996 | PDGDQWFS | -0.79 | Non-Toxin | -0.17 | 0.16 | 951.06 |
| 1997 | PDGDQWFT | -0.71 | Non-Toxin | -0.15 | 0.16 | 965.09 |
| 1998 | PDGDQWFC | -0.39 | Non-Toxin | -0.13 | 0.16 | 967.12 |
| 1999 | PDGDQWFN | -0.72 | Non-Toxin | -0.21 | 0.16 | 978.09 |
| 2000 | PDGDQWFQ | -0.78 | Non-Toxin | -0.22 | 0.31 | 992.12 |
| 2001 | PDGDQWWS | -0.73 | Non-Toxin | -0.19 | 0.16 | 990.1 |
| 2002 | PDGDQWWT | -0.69 | Non-Toxin | -0.18 | 0.16 | 1004.13 |
| 2003 | PDGDQWWC | -0.15 | Non-Toxin | -0.16 | 0.16 | 1006.16 |
| 2004 | PDGDQWWN | -0.77 | Non-Toxin | -0.24 | 0.16 | 1017.13 |
| 2005 | PDGDQWWQ | -0.66 | Non-Toxin | -0.25 | 0.31 | 1031.16 |
| 2006 | PDGDQWYS | -0.56 | Non-Toxin | -0.24 | 0.16 | 967.06 |
| 2007 | PDGDQWYT | -0.56 | Non-Toxin | -0.23 | 0.16 | 981.09 |
| 2008 | PDGDQWYC | -0.3 | Non-Toxin | -0.2 | 0.16 | 983.12 |
| 2009 | PDGDQWYN | -0.57 | Non-Toxin | -0.29 | 0.16 | 994.09 |
| 2010 | PDGDQWYQ | -0.37 | Non-Toxin | -0.29 | 0.31 | 1008.12 |
| 2011 | PDGDQYFS | -0.88 | Non-Toxin | -0.21 | 0.16 | 928.02 |
| 2012 | PDGDQYFT | -0.78 | Non-Toxin | -0.2 | 0.16 | 942.05 |
| 2013 | PDGDQYFC | -0.49 | Non-Toxin | -0.17 | 0.16 | 944.08 |
| 2014 | PDGDQYFN | -0.83 | Non-Toxin | -0.26 | 0.16 | 955.05 |
| 2015 | PDGDQYFQ | -0.9 | Non-Toxin | -0.26 | 0.31 | 969.08 |
| 2016 | PDGDQYWS | -0.93 | Non-Toxin | -0.24 | 0.16 | 967.06 |
| 2017 | PDGDQYWT | -0.88 | Non-Toxin | -0.23 | 0.16 | 981.09 |
| 2018 | PDGDQYWC | -0.34 | Non-Toxin | -0.2 | 0.16 | 983.12 |
| 2019 | PDGDQYWN | -0.97 | Non-Toxin | -0.29 | 0.16 | 994.09 |
| 2020 | PDGDQYWQ | -0.85 | Non-Toxin | -0.29 | 0.31 | 1008.12 |
| 2021 | PDGDQYYS | -0.81 | Non-Toxin | -0.28 | 0.16 | 944.02 |
| 2022 | PDGDQYYT | -0.79 | Non-Toxin | -0.27 | 0.16 | 958.05 |
| 2023 | PDGDQYYC | -0.5 | Non-Toxin | -0.24 | 0.16 | 960.08 |
| 2024 | PDGDQYYN | -0.82 | Non-Toxin | -0.33 | 0.16 | 971.05 |
| 2025 | PDGDQYYQ | -0.62 | Non-Toxin | -0.34 | 0.31 | 985.08 |
| 2026 | PDGESFFS | -0.93 | Non-Toxin | -0.07 | 0.16 | 884.99 |
| 2027 | PDGESFFT | -0.91 | Non-Toxin | -0.06 | 0.16 | 899.02 |
| 2028 | PDGESFFC | -0.6 | Non-Toxin | -0.03 | 0.16 | 901.05 |
| 2029 | PDGESFFN | -0.93 | Non-Toxin | -0.12 | 0.16 | 912.02 |
| 2030 | PDGESFFQ | -0.94 | Non-Toxin | -0.12 | 0.32 | 926.05 |
| 2031 | PDGESFWS | -1.14 | Non-Toxin | -0.1 | 0.16 | 924.03 |
| 2032 | PDGESFWT | -1.03 | Non-Toxin | -0.09 | 0.16 | 938.06 |
| 2033 | PDGESFWC | -0.49 | Non-Toxin | -0.06 | 0.16 | 940.09 |
| 2034 | PDGESFWN | -1.15 | Non-Toxin | -0.15 | 0.16 | 951.06 |
| 2035 | PDGESFWQ | -1.04 | Non-Toxin | -0.15 | 0.32 | 965.09 |
| 2036 | PDGESFYS | -1.23 | Non-Toxin | -0.14 | 0.16 | 900.99 |
| 2037 | PDGESFYT | -1.25 | Non-Toxin | -0.13 | 0.16 | 915.02 |
| 2038 | PDGESFYC | -1.07 | Non-Toxin | -0.1 | 0.16 | 917.05 |
| 2039 | PDGESFYN | -1.23 | Non-Toxin | -0.19 | 0.16 | 928.02 |
| 2040 | PDGESFYQ | -0.98 | Non-Toxin | -0.2 | 0.32 | 942.05 |
| 2041 | PDGESWFS | -1.23 | Non-Toxin | -0.1 | 0.16 | 924.03 |
| 2042 | PDGESWFT | -1.2 | Non-Toxin | -0.09 | 0.16 | 938.06 |
| 2043 | PDGESWFC | -0.88 | Non-Toxin | -0.06 | 0.16 | 940.09 |
| 2044 | PDGESWFN | -1.19 | Non-Toxin | -0.15 | 0.16 | 951.06 |
| 2045 | PDGESWFQ | -1.24 | Non-Toxin | -0.15 | 0.32 | 965.09 |
| 2046 | PDGESWWS | -1.2 | Non-Toxin | -0.13 | 0.16 | 963.07 |
| 2047 | PDGESWWT | -1.14 | Non-Toxin | -0.12 | 0.16 | 977.1 |
| 2048 | PDGESWWC | -0.62 | Non-Toxin | -0.09 | 0.16 | 979.13 |
| 2049 | PDGESWWN | -1.24 | Non-Toxin | -0.18 | 0.16 | 990.1 |
| 2050 | PDGESWWQ | -1.13 | Non-Toxin | -0.18 | 0.32 | 1004.13 |
| 2051 | PDGESWYS | -1.03 | Non-Toxin | -0.17 | 0.16 | 940.03 |
| 2052 | PDGESWYT | -1.08 | Non-Toxin | -0.16 | 0.16 | 954.06 |
| 2053 | PDGESWYC | -0.82 | Non-Toxin | -0.14 | 0.16 | 956.09 |
| 2054 | PDGESWYN | -1.04 | Non-Toxin | -0.22 | 0.16 | 967.06 |
| 2055 | PDGESWYQ | -0.77 | Non-Toxin | -0.23 | 0.32 | 981.09 |
| 2056 | PDGESYFS | -1.12 | Non-Toxin | -0.14 | 0.16 | 900.99 |
| 2057 | PDGESYFT | -1.08 | Non-Toxin | -0.13 | 0.16 | 915.02 |
| 2058 | PDGESYFC | -0.78 | Non-Toxin | -0.1 | 0.16 | 917.05 |
| 2059 | PDGESYFN | -1.12 | Non-Toxin | -0.19 | 0.16 | 928.02 |
| 2060 | PDGESYFQ | -1.16 | Non-Toxin | -0.2 | 0.32 | 942.05 |
| 2061 | PDGESYWS | -1.19 | Non-Toxin | -0.17 | 0.16 | 940.03 |
| 2062 | PDGESYWT | -1.14 | Non-Toxin | -0.16 | 0.16 | 954.06 |
| 2063 | PDGESYWC | -0.6 | Non-Toxin | -0.14 | 0.16 | 956.09 |
| 2064 | PDGESYWN | -1.24 | Non-Toxin | -0.22 | 0.16 | 967.06 |
| 2065 | PDGESYWQ | -1.12 | Non-Toxin | -0.23 | 0.32 | 981.09 |
| 2066 | PDGESYYS | -1.04 | Non-Toxin | -0.22 | 0.16 | 916.99 |
| 2067 | PDGESYYT | -1.09 | Non-Toxin | -0.21 | 0.16 | 931.02 |
| 2068 | PDGESYYC | -0.76 | Non-Toxin | -0.18 | 0.16 | 933.05 |
| 2069 | PDGESYYN | -1.05 | Non-Toxin | -0.26 | 0.16 | 944.02 |
| 2070 | PDGESYYQ | -0.77 | Non-Toxin | -0.27 | 0.32 | 958.05 |
| 2071 | PDGETFFS | -1.21 | Non-Toxin | -0.06 | 0.16 | 899.02 |
| 2072 | PDGETFFT | -1.21 | Non-Toxin | -0.05 | 0.16 | 913.05 |
| 2073 | PDGETFFC | -0.87 | Non-Toxin | -0.02 | 0.16 | 915.08 |
| 2074 | PDGETFFN | -1.14 | Non-Toxin | -0.11 | 0.16 | 926.05 |
| 2075 | PDGETFFQ | -1.25 | Non-Toxin | -0.11 | 0.32 | 940.08 |
| 2076 | PDGETFWS | -1.34 | Non-Toxin | -0.09 | 0.16 | 938.06 |
| 2077 | PDGETFWT | -1.27 | Non-Toxin | -0.08 | 0.16 | 952.09 |
| 2078 | PDGETFWC | -0.75 | Non-Toxin | -0.05 | 0.16 | 954.12 |
| 2079 | PDGETFWN | -1.4 | Non-Toxin | -0.14 | 0.16 | 965.09 |
| 2080 | PDGETFWQ | -1.28 | Non-Toxin | -0.14 | 0.32 | 979.12 |
| 2081 | PDGETFYS | -1.45 | Non-Toxin | -0.13 | 0.16 | 915.02 |
| 2082 | PDGETFYT | -1.48 | Non-Toxin | -0.12 | 0.16 | 929.05 |
| 2083 | PDGETFYC | -1.3 | Non-Toxin | -0.09 | 0.16 | 931.08 |
| 2084 | PDGETFYN | -1.46 | Non-Toxin | -0.18 | 0.16 | 942.05 |
| 2085 | PDGETFYQ | -1.22 | Non-Toxin | -0.19 | 0.32 | 956.08 |
| 2086 | PDGETWFS | -1.2 | Non-Toxin | -0.09 | 0.16 | 938.06 |
| 2087 | PDGETWFT | -1.2 | Non-Toxin | -0.08 | 0.16 | 952.09 |
| 2088 | PDGETWFC | -0.87 | Non-Toxin | -0.05 | 0.16 | 954.12 |
| 2089 | PDGETWFN | -1.16 | Non-Toxin | -0.14 | 0.16 | 965.09 |
| 2090 | PDGETWFQ | -1.24 | Non-Toxin | -0.14 | 0.32 | 979.12 |
| 2091 | PDGETWWS | -1.18 | Non-Toxin | -0.12 | 0.16 | 977.1 |
| 2092 | PDGETWWT | -1.14 | Non-Toxin | -0.11 | 0.16 | 991.13 |
| 2093 | PDGETWWC | -0.57 | Non-Toxin | -0.08 | 0.16 | 993.16 |
| 2094 | PDGETWWN | -1.23 | Non-Toxin | -0.17 | 0.16 | 1004.13 |
| 2095 | PDGETWWQ | -1.12 | Non-Toxin | -0.17 | 0.32 | 1018.16 |
| 2096 | PDGETWYS | -1.05 | Non-Toxin | -0.16 | 0.16 | 954.06 |
| 2097 | PDGETWYT | -1.07 | Non-Toxin | -0.15 | 0.16 | 968.09 |
| 2098 | PDGETWYC | -0.83 | Non-Toxin | -0.12 | 0.16 | 970.12 |
| 2099 | PDGETWYN | -1.05 | Non-Toxin | -0.21 | 0.16 | 981.09 |
| 2100 | PDGETWYQ | -0.79 | Non-Toxin | -0.22 | 0.32 | 995.12 |
| 2101 | PDGETYFS | -1.06 | Non-Toxin | -0.13 | 0.16 | 915.02 |
| 2102 | PDGETYFT | -1.05 | Non-Toxin | -0.12 | 0.16 | 929.05 |
| 2103 | PDGETYFC | -0.76 | Non-Toxin | -0.09 | 0.16 | 931.08 |
| 2104 | PDGETYFN | -1.08 | Non-Toxin | -0.18 | 0.16 | 942.05 |
| 2105 | PDGETYFQ | -1.13 | Non-Toxin | -0.19 | 0.32 | 956.08 |
| 2106 | PDGETYWS | -1.14 | Non-Toxin | -0.16 | 0.16 | 954.06 |
| 2107 | PDGETYWT | -1.11 | Non-Toxin | -0.15 | 0.16 | 968.09 |
| 2108 | PDGETYWC | -0.56 | Non-Toxin | -0.12 | 0.16 | 970.12 |
| 2109 | PDGETYWN | -1.21 | Non-Toxin | -0.21 | 0.16 | 981.09 |
| 2110 | PDGETYWQ | -1.09 | Non-Toxin | -0.22 | 0.32 | 995.12 |
| 2111 | PDGETYYS | -1.06 | Non-Toxin | -0.21 | 0.16 | 931.02 |
| 2112 | PDGETYYT | -1.05 | Non-Toxin | -0.2 | 0.16 | 945.05 |
| 2113 | PDGETYYC | -0.79 | Non-Toxin | -0.17 | 0.16 | 947.08 |
| 2114 | PDGETYYN | -1.05 | Non-Toxin | -0.25 | 0.16 | 958.05 |
| 2115 | PDGETYYQ | -0.79 | Non-Toxin | -0.26 | 0.32 | 972.08 |
| 2116 | PDGECFFS | -0.5 | Non-Toxin | -0.03 | 0.16 | 901.05 |
| 2117 | PDGECFFT | -0.44 | Non-Toxin | -0.02 | 0.16 | 915.08 |
| 2118 | PDGECFFC | -0.08 | Non-Toxin | 0.01 | 0.16 | 917.11 |
| 2119 | PDGECFFN | -0.44 | Non-Toxin | -0.08 | 0.16 | 928.08 |
| 2120 | PDGECFFQ | -0.54 | Non-Toxin | -0.08 | 0.32 | 942.11 |
| 2121 | PDGECFWS | -0.77 | Non-Toxin | -0.06 | 0.16 | 940.09 |
| 2122 | PDGECFWT | -0.74 | Non-Toxin | -0.05 | 0.16 | 954.12 |
| 2123 | PDGECFWC | -0.19 | Non-Toxin | -0.02 | 0.16 | 956.15 |
| 2124 | PDGECFWN | -0.83 | Non-Toxin | -0.11 | 0.16 | 967.12 |
| 2125 | PDGECFWQ | -0.71 | Non-Toxin | -0.11 | 0.32 | 981.15 |
| 2126 | PDGECFYS | -0.78 | Non-Toxin | -0.1 | 0.16 | 917.05 |
| 2127 | PDGECFYT | -0.8 | Non-Toxin | -0.1 | 0.16 | 931.08 |
| 2128 | PDGECFYC | -0.71 | Non-Toxin | -0.07 | 0.16 | 933.11 |
| 2129 | PDGECFYN | -0.79 | Non-Toxin | -0.15 | 0.16 | 944.08 |
| 2130 | PDGECFYQ | -0.55 | Non-Toxin | -0.16 | 0.32 | 958.11 |
| 2131 | PDGECWFS | -0.46 | Non-Toxin | -0.06 | 0.16 | 940.09 |
| 2132 | PDGECWFT | -0.39 | Non-Toxin | -0.05 | 0.16 | 954.12 |
| 2133 | PDGECWFC | -0.07 | Non-Toxin | -0.02 | 0.16 | 956.15 |
| 2134 | PDGECWFN | -0.37 | Non-Toxin | -0.11 | 0.16 | 967.12 |
| 2135 | PDGECWFQ | -0.47 | Non-Toxin | -0.11 | 0.32 | 981.15 |
| 2136 | PDGECWWS | -0.38 | Non-Toxin | -0.09 | 0.16 | 979.13 |
| 2137 | PDGECWWT | -0.37 | Non-Toxin | -0.08 | 0.16 | 993.16 |
| 2138 | PDGECWWC | 0.2 | Toxin | -0.05 | 0.16 | 995.19 |
| 2139 | PDGECWWN | -0.45 | Non-Toxin | -0.14 | 0.16 | 1006.16 |
| 2140 | PDGECWWQ | -0.33 | Non-Toxin | -0.14 | 0.32 | 1020.19 |
| 2141 | PDGECWYS | -0.25 | Non-Toxin | -0.14 | 0.16 | 956.09 |
| 2142 | PDGECWYT | -0.25 | Non-Toxin | -0.12 | 0.16 | 970.12 |
| 2143 | PDGECWYC | -0.05 | Non-Toxin | -0.1 | 0.16 | 972.15 |
| 2144 | PDGECWYN | -0.25 | Non-Toxin | -0.18 | 0.16 | 983.12 |
| 2145 | PDGECWYQ | 0.01 | Toxin | -0.19 | 0.32 | 997.15 |
| 2146 | PDGECYFS | -0.42 | Non-Toxin | -0.1 | 0.16 | 917.05 |
| 2147 | PDGECYFT | -0.33 | Non-Toxin | -0.1 | 0.16 | 931.08 |
| 2148 | PDGECYFC | -0.06 | Non-Toxin | -0.07 | 0.16 | 933.11 |
| 2149 | PDGECYFN | -0.36 | Non-Toxin | -0.15 | 0.16 | 944.08 |
| 2150 | PDGECYFQ | -0.45 | Non-Toxin | -0.16 | 0.32 | 958.11 |
| 2151 | PDGECYWS | -0.37 | Non-Toxin | -0.14 | 0.16 | 956.09 |
| 2152 | PDGECYWT | -0.35 | Non-Toxin | -0.12 | 0.16 | 970.12 |
| 2153 | PDGECYWC | 0.17 | Toxin | -0.1 | 0.16 | 972.15 |
| 2154 | PDGECYWN | -0.46 | Non-Toxin | -0.18 | 0.16 | 983.12 |
| 2155 | PDGECYWQ | -0.33 | Non-Toxin | -0.19 | 0.32 | 997.15 |
| 2156 | PDGECYYS | -0.26 | Non-Toxin | -0.18 | 0.16 | 933.05 |
| 2157 | PDGECYYT | -0.32 | Non-Toxin | -0.17 | 0.16 | 947.08 |
| 2158 | PDGECYYC | -0.06 | Non-Toxin | -0.14 | 0.16 | 949.11 |
| 2159 | PDGECYYN | -0.25 | Non-Toxin | -0.23 | 0.16 | 960.08 |
| 2160 | PDGECYYQ | -0.03 | Non-Toxin | -0.23 | 0.32 | 974.11 |
| 2161 | PDGENFFS | -1.2 | Non-Toxin | -0.12 | 0.16 | 912.02 |
| 2162 | PDGENFFT | -1.13 | Non-Toxin | -0.11 | 0.16 | 926.05 |
| 2163 | PDGENFFC | -0.8 | Non-Toxin | -0.08 | 0.16 | 928.08 |
| 2164 | PDGENFFN | -1.11 | Non-Toxin | -0.16 | 0.16 | 939.05 |
| 2165 | PDGENFFQ | -1.2 | Non-Toxin | -0.17 | 0.32 | 953.08 |
| 2166 | PDGENFWS | -1.32 | Non-Toxin | -0.15 | 0.16 | 951.06 |
| 2167 | PDGENFWT | -1.27 | Non-Toxin | -0.14 | 0.16 | 965.09 |
| 2168 | PDGENFWC | -0.76 | Non-Toxin | -0.11 | 0.16 | 967.12 |
| 2169 | PDGENFWN | -1.38 | Non-Toxin | -0.19 | 0.16 | 978.09 |
| 2170 | PDGENFWQ | -1.27 | Non-Toxin | -0.2 | 0.32 | 992.12 |
| 2171 | PDGENFYS | -1.39 | Non-Toxin | -0.19 | 0.16 | 928.02 |
| 2172 | PDGENFYT | -1.4 | Non-Toxin | -0.18 | 0.16 | 942.05 |
| 2173 | PDGENFYC | -1.23 | Non-Toxin | -0.15 | 0.16 | 944.08 |
| 2174 | PDGENFYN | -1.41 | Non-Toxin | -0.24 | 0.16 | 955.05 |
| 2175 | PDGENFYQ | -1.18 | Non-Toxin | -0.24 | 0.32 | 969.08 |
| 2176 | PDGENWFS | -1.06 | Non-Toxin | -0.15 | 0.16 | 951.06 |
| 2177 | PDGENWFT | -1.02 | Non-Toxin | -0.14 | 0.16 | 965.09 |
| 2178 | PDGENWFC | -0.7 | Non-Toxin | -0.11 | 0.16 | 967.12 |
| 2179 | PDGENWFN | -1 | Non-Toxin | -0.19 | 0.16 | 978.09 |
| 2180 | PDGENWFQ | -1.07 | Non-Toxin | -0.2 | 0.32 | 992.12 |
| 2181 | PDGENWWS | -1.01 | Non-Toxin | -0.18 | 0.16 | 990.1 |
| 2182 | PDGENWWT | -0.98 | Non-Toxin | -0.17 | 0.16 | 1004.13 |
| 2183 | PDGENWWC | -0.45 | Non-Toxin | -0.14 | 0.16 | 1006.16 |
| 2184 | PDGENWWN | -1.07 | Non-Toxin | -0.22 | 0.16 | 1017.13 |
| 2185 | PDGENWWQ | -0.96 | Non-Toxin | -0.23 | 0.32 | 1031.16 |
| 2186 | PDGENWYS | -0.86 | Non-Toxin | -0.22 | 0.16 | 967.06 |
| 2187 | PDGENWYT | -0.9 | Non-Toxin | -0.21 | 0.16 | 981.09 |
| 2188 | PDGENWYC | -0.66 | Non-Toxin | -0.18 | 0.16 | 983.12 |
| 2189 | PDGENWYN | -0.88 | Non-Toxin | -0.27 | 0.16 | 994.09 |
| 2190 | PDGENWYQ | -0.63 | Non-Toxin | -0.27 | 0.32 | 1008.12 |
| 2191 | PDGENYFS | -0.99 | Non-Toxin | -0.19 | 0.16 | 928.02 |
| 2192 | PDGENYFT | -0.94 | Non-Toxin | -0.18 | 0.16 | 942.05 |
| 2193 | PDGENYFC | -0.65 | Non-Toxin | -0.15 | 0.16 | 944.08 |
| 2194 | PDGENYFN | -0.97 | Non-Toxin | -0.24 | 0.16 | 955.05 |
| 2195 | PDGENYFQ | -1.02 | Non-Toxin | -0.24 | 0.32 | 969.08 |
| 2196 | PDGENYWS | -1.05 | Non-Toxin | -0.22 | 0.16 | 967.06 |
| 2197 | PDGENYWT | -1.02 | Non-Toxin | -0.21 | 0.16 | 981.09 |
| 2198 | PDGENYWC | -0.49 | Non-Toxin | -0.18 | 0.16 | 983.12 |
| 2199 | PDGENYWN | -1.13 | Non-Toxin | -0.27 | 0.16 | 994.09 |
| 2200 | PDGENYWQ | -1 | Non-Toxin | -0.27 | 0.32 | 1008.12 |
| 2201 | PDGENYYS | -0.92 | Non-Toxin | -0.26 | 0.16 | 944.02 |
| 2202 | PDGENYYT | -0.92 | Non-Toxin | -0.25 | 0.16 | 958.05 |
| 2203 | PDGENYYC | -0.59 | Non-Toxin | -0.23 | 0.16 | 960.08 |
| 2204 | PDGENYYN | -0.95 | Non-Toxin | -0.31 | 0.16 | 971.05 |
| 2205 | PDGENYYQ | -0.69 | Non-Toxin | -0.32 | 0.32 | 985.08 |
| 2206 | PDGEQFFS | -0.94 | Non-Toxin | -0.12 | 0.32 | 926.05 |
| 2207 | PDGEQFFT | -0.86 | Non-Toxin | -0.11 | 0.32 | 940.08 |
| 2208 | PDGEQFFC | -0.55 | Non-Toxin | -0.09 | 0.32 | 942.11 |
| 2209 | PDGEQFFN | -0.85 | Non-Toxin | -0.17 | 0.32 | 953.08 |
| 2210 | PDGEQFFQ | -0.96 | Non-Toxin | -0.18 | 0.47 | 967.11 |
| 2211 | PDGEQFWS | -1.09 | Non-Toxin | -0.15 | 0.32 | 965.09 |
| 2212 | PDGEQFWT | -1.02 | Non-Toxin | -0.14 | 0.32 | 979.12 |
| 2213 | PDGEQFWC | -0.5 | Non-Toxin | -0.12 | 0.32 | 981.15 |
| 2214 | PDGEQFWN | -1.12 | Non-Toxin | -0.2 | 0.32 | 992.12 |
| 2215 | PDGEQFWQ | -1.02 | Non-Toxin | -0.21 | 0.47 | 1006.15 |
| 2216 | PDGEQFYS | -1.17 | Non-Toxin | -0.2 | 0.32 | 942.05 |
| 2217 | PDGEQFYT | -1.16 | Non-Toxin | -0.19 | 0.32 | 956.08 |
| 2218 | PDGEQFYC | -0.99 | Non-Toxin | -0.16 | 0.32 | 958.11 |
| 2219 | PDGEQFYN | -1.17 | Non-Toxin | -0.24 | 0.32 | 969.08 |
| 2220 | PDGEQFYQ | -0.92 | Non-Toxin | -0.25 | 0.47 | 983.11 |
| 2221 | PDGEQWFS | -1.03 | Non-Toxin | -0.15 | 0.32 | 965.09 |
| 2222 | PDGEQWFT | -0.97 | Non-Toxin | -0.14 | 0.32 | 979.12 |
| 2223 | PDGEQWFC | -0.66 | Non-Toxin | -0.11 | 0.32 | 981.15 |
| 2224 | PDGEQWFN | -0.97 | Non-Toxin | -0.2 | 0.32 | 992.12 |
| 2225 | PDGEQWFQ | -1.03 | Non-Toxin | -0.21 | 0.47 | 1006.15 |
| 2226 | PDGEQWWS | -1 | Non-Toxin | -0.18 | 0.32 | 1004.13 |
| 2227 | PDGEQWWT | -0.95 | Non-Toxin | -0.17 | 0.32 | 1018.16 |
| 2228 | PDGEQWWC | -0.42 | Non-Toxin | -0.14 | 0.32 | 1020.19 |
| 2229 | PDGEQWWN | -1.05 | Non-Toxin | -0.23 | 0.32 | 1031.16 |
| 2230 | PDGEQWWQ | -0.93 | Non-Toxin | -0.24 | 0.47 | 1045.19 |
| 2231 | PDGEQWYS | -0.84 | Non-Toxin | -0.23 | 0.32 | 981.09 |
| 2232 | PDGEQWYT | -0.86 | Non-Toxin | -0.22 | 0.32 | 995.12 |
| 2233 | PDGEQWYC | -0.62 | Non-Toxin | -0.19 | 0.32 | 997.15 |
| 2234 | PDGEQWYN | -0.84 | Non-Toxin | -0.27 | 0.32 | 1008.12 |
| 2235 | PDGEQWYQ | -0.61 | Non-Toxin | -0.28 | 0.47 | 1022.15 |
| 2236 | PDGEQYFS | -1.17 | Non-Toxin | -0.2 | 0.32 | 942.05 |
| 2237 | PDGEQYFT | -1.08 | Non-Toxin | -0.19 | 0.32 | 956.08 |
| 2238 | PDGEQYFC | -0.81 | Non-Toxin | -0.16 | 0.32 | 958.11 |
| 2239 | PDGEQYFN | -1.12 | Non-Toxin | -0.24 | 0.32 | 969.08 |
| 2240 | PDGEQYFQ | -1.19 | Non-Toxin | -0.25 | 0.47 | 983.11 |
| 2241 | PDGEQYWS | -1.21 | Non-Toxin | -0.23 | 0.32 | 981.09 |
| 2242 | PDGEQYWT | -1.15 | Non-Toxin | -0.22 | 0.32 | 995.12 |
| 2243 | PDGEQYWC | -0.62 | Non-Toxin | -0.19 | 0.32 | 997.15 |
| 2244 | PDGEQYWN | -1.25 | Non-Toxin | -0.27 | 0.32 | 1008.12 |
| 2245 | PDGEQYWQ | -1.13 | Non-Toxin | -0.28 | 0.47 | 1022.15 |
| 2246 | PDGEQYYS | -1.1 | Non-Toxin | -0.27 | 0.32 | 958.05 |
| 2247 | PDGEQYYT | -1.1 | Non-Toxin | -0.26 | 0.32 | 972.08 |
| 2248 | PDGEQYYC | -0.83 | Non-Toxin | -0.23 | 0.32 | 974.11 |
| 2249 | PDGEQYYN | -1.09 | Non-Toxin | -0.32 | 0.32 | 985.08 |
| 2250 | PDGEQYYQ | -0.87 | Non-Toxin | -0.32 | 0.47 | 999.11 |
| 2251 | PDPDSFFS | -0.81 | Non-Toxin | -0.11 | 0 | 911.02 |
| 2252 | PDPDSFFT | -0.77 | Non-Toxin | -0.1 | 0 | 925.05 |
| 2253 | PDPDSFFC | -0.57 | Non-Toxin | -0.07 | 0 | 927.08 |
| 2254 | PDPDSFFN | -0.79 | Non-Toxin | -0.16 | 0 | 938.05 |
| 2255 | PDPDSFFQ | -0.81 | Non-Toxin | -0.16 | 0.16 | 952.08 |
| 2256 | PDPDSFWS | -0.94 | Non-Toxin | -0.14 | 0 | 950.06 |
| 2257 | PDPDSFWT | -0.89 | Non-Toxin | -0.13 | 0 | 964.09 |
| 2258 | PDPDSFWC | -0.51 | Non-Toxin | -0.1 | 0 | 966.12 |
| 2259 | PDPDSFWN | -0.96 | Non-Toxin | -0.19 | 0 | 977.09 |
| 2260 | PDPDSFWQ | -0.89 | Non-Toxin | -0.19 | 0.16 | 991.12 |
| 2261 | PDPDSFYS | -1.01 | Non-Toxin | -0.18 | 0 | 927.02 |
| 2262 | PDPDSFYT | -1.04 | Non-Toxin | -0.17 | 0 | 941.05 |
| 2263 | PDPDSFYC | -0.98 | Non-Toxin | -0.15 | 0 | 943.08 |
| 2264 | PDPDSFYN | -1.02 | Non-Toxin | -0.23 | 0 | 954.05 |
| 2265 | PDPDSFYQ | -0.92 | Non-Toxin | -0.24 | 0.16 | 968.08 |
| 2266 | PDPDSWFS | -1.01 | Non-Toxin | -0.14 | 0 | 950.06 |
| 2267 | PDPDSWFT | -0.97 | Non-Toxin | -0.13 | 0 | 964.09 |
| 2268 | PDPDSWFC | -0.76 | Non-Toxin | -0.1 | 0 | 966.12 |
| 2269 | PDPDSWFN | -0.97 | Non-Toxin | -0.19 | 0 | 977.09 |
| 2270 | PDPDSWFQ | -1.02 | Non-Toxin | -0.19 | 0.16 | 991.12 |
| 2271 | PDPDSWWS | -0.96 | Non-Toxin | -0.17 | 0 | 989.1 |
| 2272 | PDPDSWWT | -0.95 | Non-Toxin | -0.16 | 0 | 1003.13 |
| 2273 | PDPDSWWC | -0.57 | Non-Toxin | -0.13 | 0 | 1005.16 |
| 2274 | PDPDSWWN | -1 | Non-Toxin | -0.22 | 0 | 1016.13 |
| 2275 | PDPDSWWQ | -0.93 | Non-Toxin | -0.22 | 0.16 | 1030.16 |
| 2276 | PDPDSWYS | -0.81 | Non-Toxin | -0.21 | 0 | 966.06 |
| 2277 | PDPDSWYT | -0.86 | Non-Toxin | -0.2 | 0 | 980.09 |
| 2278 | PDPDSWYC | -0.75 | Non-Toxin | -0.18 | 0 | 982.12 |
| 2279 | PDPDSWYN | -0.83 | Non-Toxin | -0.26 | 0 | 993.09 |
| 2280 | PDPDSWYQ | -0.72 | Non-Toxin | -0.27 | 0.16 | 1007.12 |
| 2281 | PDPDSYFS | -0.92 | Non-Toxin | -0.18 | 0 | 927.02 |
| 2282 | PDPDSYFT | -0.86 | Non-Toxin | -0.17 | 0 | 941.05 |
| 2283 | PDPDSYFC | -0.67 | Non-Toxin | -0.15 | 0 | 943.08 |
| 2284 | PDPDSYFN | -0.9 | Non-Toxin | -0.23 | 0 | 954.05 |
| 2285 | PDPDSYFQ | -0.94 | Non-Toxin | -0.24 | 0.16 | 968.08 |
| 2286 | PDPDSYWS | -0.93 | Non-Toxin | -0.21 | 0 | 966.06 |
| 2287 | PDPDSYWT | -0.93 | Non-Toxin | -0.2 | 0 | 980.09 |
| 2288 | PDPDSYWC | -0.55 | Non-Toxin | -0.18 | 0 | 982.12 |
| 2289 | PDPDSYWN | -0.98 | Non-Toxin | -0.26 | 0 | 993.09 |
| 2290 | PDPDSYWQ | -0.91 | Non-Toxin | -0.27 | 0.16 | 1007.12 |
| 2291 | PDPDSYYS | -0.79 | Non-Toxin | -0.26 | 0 | 943.02 |
| 2292 | PDPDSYYT | -0.84 | Non-Toxin | -0.25 | 0 | 957.05 |
| 2293 | PDPDSYYC | -0.68 | Non-Toxin | -0.22 | 0 | 959.08 |
| 2294 | PDPDSYYN | -0.81 | Non-Toxin | -0.3 | 0 | 970.05 |
| 2295 | PDPDSYYQ | -0.69 | Non-Toxin | -0.31 | 0.16 | 984.08 |
| 2296 | PDPDTFFS | -0.69 | Non-Toxin | -0.1 | 0 | 925.05 |
| 2297 | PDPDTFFT | -0.64 | Non-Toxin | -0.09 | 0 | 939.08 |
| 2298 | PDPDTFFC | -0.39 | Non-Toxin | -0.06 | 0 | 941.11 |
| 2299 | PDPDTFFN | -0.63 | Non-Toxin | -0.15 | 0 | 952.08 |
| 2300 | PDPDTFFQ | -0.7 | Non-Toxin | -0.15 | 0.16 | 966.11 |
| 2301 | PDPDTFWS | -0.79 | Non-Toxin | -0.13 | 0 | 964.09 |
| 2302 | PDPDTFWT | -0.77 | Non-Toxin | -0.12 | 0 | 978.12 |
| 2303 | PDPDTFWC | -0.41 | Non-Toxin | -0.09 | 0 | 980.15 |
| 2304 | PDPDTFWN | -0.84 | Non-Toxin | -0.18 | 0 | 991.12 |
| 2305 | PDPDTFWQ | -0.77 | Non-Toxin | -0.18 | 0.16 | 1005.15 |
| 2306 | PDPDTFYS | -0.83 | Non-Toxin | -0.17 | 0 | 941.05 |
| 2307 | PDPDTFYT | -0.9 | Non-Toxin | -0.16 | 0 | 955.08 |
| 2308 | PDPDTFYC | -0.78 | Non-Toxin | -0.14 | 0 | 957.11 |
| 2309 | PDPDTFYN | -0.88 | Non-Toxin | -0.22 | 0 | 968.08 |
| 2310 | PDPDTFYQ | -0.78 | Non-Toxin | -0.23 | 0.16 | 982.11 |
| 2311 | PDPDTWFS | -0.8 | Non-Toxin | -0.13 | 0 | 964.09 |
| 2312 | PDPDTWFT | -0.74 | Non-Toxin | -0.12 | 0 | 978.12 |
| 2313 | PDPDTWFC | -0.51 | Non-Toxin | -0.09 | 0 | 980.15 |
| 2314 | PDPDTWFN | -0.75 | Non-Toxin | -0.18 | 0 | 991.12 |
| 2315 | PDPDTWFQ | -0.81 | Non-Toxin | -0.18 | 0.16 | 1005.15 |
| 2316 | PDPDTWWS | -0.75 | Non-Toxin | -0.16 | 0 | 1003.13 |
| 2317 | PDPDTWWT | -0.75 | Non-Toxin | -0.15 | 0 | 1017.16 |
| 2318 | PDPDTWWC | -0.37 | Non-Toxin | -0.12 | 0 | 1019.19 |
| 2319 | PDPDTWWN | -0.8 | Non-Toxin | -0.21 | 0 | 1030.16 |
| 2320 | PDPDTWWQ | -0.73 | Non-Toxin | -0.21 | 0.16 | 1044.19 |
| 2321 | PDPDTWYS | -0.62 | Non-Toxin | -0.2 | 0 | 980.09 |
| 2322 | PDPDTWYT | -0.68 | Non-Toxin | -0.19 | 0 | 994.12 |
| 2323 | PDPDTWYC | -0.53 | Non-Toxin | -0.17 | 0 | 996.15 |
| 2324 | PDPDTWYN | -0.66 | Non-Toxin | -0.25 | 0 | 1007.12 |
| 2325 | PDPDTWYQ | -0.54 | Non-Toxin | -0.26 | 0.16 | 1021.15 |
| 2326 | PDPDTYFS | -0.72 | Non-Toxin | -0.17 | 0 | 941.05 |
| 2327 | PDPDTYFT | -0.65 | Non-Toxin | -0.16 | 0 | 955.08 |
| 2328 | PDPDTYFC | -0.44 | Non-Toxin | -0.14 | 0 | 957.11 |
| 2329 | PDPDTYFN | -0.71 | Non-Toxin | -0.22 | 0 | 968.08 |
| 2330 | PDPDTYFQ | -0.75 | Non-Toxin | -0.23 | 0.16 | 982.11 |
| 2331 | PDPDTYWS | -0.74 | Non-Toxin | -0.2 | 0 | 980.09 |
| 2332 | PDPDTYWT | -0.74 | Non-Toxin | -0.19 | 0 | 994.12 |
| 2333 | PDPDTYWC | -0.37 | Non-Toxin | -0.17 | 0 | 996.15 |
| 2334 | PDPDTYWN | -0.8 | Non-Toxin | -0.25 | 0 | 1007.12 |
| 2335 | PDPDTYWQ | -0.72 | Non-Toxin | -0.26 | 0.16 | 1021.15 |
| 2336 | PDPDTYYS | -0.64 | Non-Toxin | -0.25 | 0 | 957.05 |
| 2337 | PDPDTYYT | -0.68 | Non-Toxin | -0.24 | 0 | 971.08 |
| 2338 | PDPDTYYC | -0.51 | Non-Toxin | -0.21 | 0 | 973.11 |
| 2339 | PDPDTYYN | -0.67 | Non-Toxin | -0.29 | 0 | 984.08 |
| 2340 | PDPDTYYQ | -0.55 | Non-Toxin | -0.3 | 0.16 | 998.11 |
| 2341 | PDPDCFFS | -0.53 | Non-Toxin | -0.07 | 0 | 927.08 |
| 2342 | PDPDCFFT | -0.47 | Non-Toxin | -0.06 | 0 | 941.11 |
| 2343 | PDPDCFFC | -0.2 | Non-Toxin | -0.04 | 0 | 943.14 |
| 2344 | PDPDCFFN | -0.48 | Non-Toxin | -0.12 | 0 | 954.11 |
| 2345 | PDPDCFFQ | -0.55 | Non-Toxin | -0.13 | 0.16 | 968.14 |
| 2346 | PDPDCFWS | -0.71 | Non-Toxin | -0.1 | 0 | 966.12 |
| 2347 | PDPDCFWT | -0.71 | Non-Toxin | -0.09 | 0 | 980.15 |
| 2348 | PDPDCFWC | -0.34 | Non-Toxin | -0.07 | 0 | 982.18 |
| 2349 | PDPDCFWN | -0.76 | Non-Toxin | -0.15 | 0 | 993.15 |
| 2350 | PDPDCFWQ | -0.69 | Non-Toxin | -0.16 | 0.16 | 1007.18 |
| 2351 | PDPDCFYS | -0.73 | Non-Toxin | -0.15 | 0 | 943.08 |
| 2352 | PDPDCFYT | -0.79 | Non-Toxin | -0.14 | 0 | 957.11 |
| 2353 | PDPDCFYC | -0.73 | Non-Toxin | -0.11 | 0 | 959.14 |
| 2354 | PDPDCFYN | -0.77 | Non-Toxin | -0.19 | 0 | 970.11 |
| 2355 | PDPDCFYQ | -0.66 | Non-Toxin | -0.2 | 0.16 | 984.14 |
| 2356 | PDPDCWFS | -0.53 | Non-Toxin | -0.1 | 0 | 966.12 |
| 2357 | PDPDCWFT | -0.47 | Non-Toxin | -0.09 | 0 | 980.15 |
| 2358 | PDPDCWFC | -0.22 | Non-Toxin | -0.06 | 0 | 982.18 |
| 2359 | PDPDCWFN | -0.47 | Non-Toxin | -0.15 | 0 | 993.15 |
| 2360 | PDPDCWFQ | -0.54 | Non-Toxin | -0.16 | 0.16 | 1007.18 |
| 2361 | PDPDCWWS | -0.47 | Non-Toxin | -0.13 | 0 | 1005.16 |
| 2362 | PDPDCWWT | -0.49 | Non-Toxin | -0.12 | 0 | 1019.19 |
| 2363 | PDPDCWWC | -0.1 | Non-Toxin | -0.09 | 0 | 1021.22 |
| 2364 | PDPDCWWN | -0.53 | Non-Toxin | -0.18 | 0 | 1032.19 |
| 2365 | PDPDCWWQ | -0.46 | Non-Toxin | -0.19 | 0.16 | 1046.22 |
| 2366 | PDPDCWYS | -0.36 | Non-Toxin | -0.18 | 0 | 982.12 |
| 2367 | PDPDCWYT | -0.41 | Non-Toxin | -0.17 | 0 | 996.15 |
| 2368 | PDPDCWYC | -0.28 | Non-Toxin | -0.14 | 0 | 998.18 |
| 2369 | PDPDCWYN | -0.4 | Non-Toxin | -0.22 | 0 | 1009.15 |
| 2370 | PDPDCWYQ | -0.27 | Non-Toxin | -0.23 | 0.16 | 1023.18 |
| 2371 | PDPDCYFS | -0.46 | Non-Toxin | -0.15 | 0 | 943.08 |
| 2372 | PDPDCYFT | -0.39 | Non-Toxin | -0.14 | 0 | 957.11 |
| 2373 | PDPDCYFC | -0.17 | Non-Toxin | -0.11 | 0 | 959.14 |
| 2374 | PDPDCYFN | -0.41 | Non-Toxin | -0.19 | 0 | 970.11 |
| 2375 | PDPDCYFQ | -0.48 | Non-Toxin | -0.2 | 0.16 | 984.14 |
| 2376 | PDPDCYWS | -0.39 | Non-Toxin | -0.18 | 0 | 982.12 |
| 2377 | PDPDCYWT | -0.4 | Non-Toxin | -0.17 | 0 | 996.15 |
| 2378 | PDPDCYWC | -0.05 | Non-Toxin | -0.14 | 0 | 998.18 |
| 2379 | PDPDCYWN | -0.47 | Non-Toxin | -0.22 | 0 | 1009.15 |
| 2380 | PDPDCYWQ | -0.39 | Non-Toxin | -0.23 | 0.16 | 1023.18 |
| 2381 | PDPDCYYS | -0.31 | Non-Toxin | -0.22 | 0 | 959.08 |
| 2382 | PDPDCYYT | -0.41 | Non-Toxin | -0.21 | 0 | 973.11 |
| 2383 | PDPDCYYC | -0.23 | Non-Toxin | -0.18 | 0 | 975.14 |
| 2384 | PDPDCYYN | -0.35 | Non-Toxin | -0.27 | 0 | 986.11 |
| 2385 | PDPDCYYQ | -0.25 | Non-Toxin | -0.27 | 0.16 | 1000.14 |
| 2386 | PDPDNFFS | -0.86 | Non-Toxin | -0.16 | 0 | 938.05 |
| 2387 | PDPDNFFT | -0.8 | Non-Toxin | -0.15 | 0 | 952.08 |
| 2388 | PDPDNFFC | -0.57 | Non-Toxin | -0.12 | 0 | 954.11 |
| 2389 | PDPDNFFN | -0.81 | Non-Toxin | -0.21 | 0 | 965.08 |
| 2390 | PDPDNFFQ | -0.87 | Non-Toxin | -0.21 | 0.16 | 979.11 |
| 2391 | PDPDNFWS | -0.96 | Non-Toxin | -0.19 | 0 | 977.09 |
| 2392 | PDPDNFWT | -0.95 | Non-Toxin | -0.18 | 0 | 991.12 |
| 2393 | PDPDNFWC | -0.6 | Non-Toxin | -0.15 | 0 | 993.15 |
| 2394 | PDPDNFWN | -1.01 | Non-Toxin | -0.24 | 0 | 1004.12 |
| 2395 | PDPDNFWQ | -0.94 | Non-Toxin | -0.24 | 0.16 | 1018.15 |
| 2396 | PDPDNFYS | -1.03 | Non-Toxin | -0.23 | 0 | 954.05 |
| 2397 | PDPDNFYT | -1.07 | Non-Toxin | -0.22 | 0 | 968.08 |
| 2398 | PDPDNFYC | -0.96 | Non-Toxin | -0.19 | 0 | 970.11 |
| 2399 | PDPDNFYN | -1.05 | Non-Toxin | -0.28 | 0 | 981.08 |
| 2400 | PDPDNFYQ | -0.95 | Non-Toxin | -0.29 | 0.16 | 995.11 |
| 2401 | PDPDNWFS | -0.81 | Non-Toxin | -0.19 | 0 | 977.09 |
| 2402 | PDPDNWFT | -0.77 | Non-Toxin | -0.18 | 0 | 991.12 |
| 2403 | PDPDNWFC | -0.54 | Non-Toxin | -0.15 | 0 | 993.15 |
| 2404 | PDPDNWFN | -0.78 | Non-Toxin | -0.24 | 0 | 1004.12 |
| 2405 | PDPDNWFQ | -0.83 | Non-Toxin | -0.24 | 0.16 | 1018.15 |
| 2406 | PDPDNWWS | -0.76 | Non-Toxin | -0.22 | 0 | 1016.13 |
| 2407 | PDPDNWWT | -0.77 | Non-Toxin | -0.21 | 0 | 1030.16 |
| 2408 | PDPDNWWC | -0.4 | Non-Toxin | -0.18 | 0 | 1032.19 |
| 2409 | PDPDNWWN | -0.82 | Non-Toxin | -0.27 | 0 | 1043.16 |
| 2410 | PDPDNWWQ | -0.75 | Non-Toxin | -0.27 | 0.16 | 1057.19 |
| 2411 | PDPDNWYS | -0.65 | Non-Toxin | -0.26 | 0 | 993.09 |
| 2412 | PDPDNWYT | -0.71 | Non-Toxin | -0.25 | 0 | 1007.12 |
| 2413 | PDPDNWYC | -0.56 | Non-Toxin | -0.22 | 0 | 1009.15 |
| 2414 | PDPDNWYN | -0.67 | Non-Toxin | -0.31 | 0 | 1020.12 |
| 2415 | PDPDNWYQ | -0.56 | Non-Toxin | -0.32 | 0.16 | 1034.15 |
| 2416 | PDPDNYFS | -0.75 | Non-Toxin | -0.23 | 0 | 954.05 |
| 2417 | PDPDNYFT | -0.71 | Non-Toxin | -0.22 | 0 | 968.08 |
| 2418 | PDPDNYFC | -0.49 | Non-Toxin | -0.19 | 0 | 970.11 |
| 2419 | PDPDNYFN | -0.74 | Non-Toxin | -0.28 | 0 | 981.08 |
| 2420 | PDPDNYFQ | -0.78 | Non-Toxin | -0.29 | 0.16 | 995.11 |
| 2421 | PDPDNYWS | -0.77 | Non-Toxin | -0.26 | 0 | 993.09 |
| 2422 | PDPDNYWT | -0.78 | Non-Toxin | -0.25 | 0 | 1007.12 |
| 2423 | PDPDNYWC | -0.41 | Non-Toxin | -0.22 | 0 | 1009.15 |
| 2424 | PDPDNYWN | -0.84 | Non-Toxin | -0.31 | 0 | 1020.12 |
| 2425 | PDPDNYWQ | -0.76 | Non-Toxin | -0.32 | 0.16 | 1034.15 |
| 2426 | PDPDNYYS | -0.68 | Non-Toxin | -0.31 | 0 | 970.05 |
| 2427 | PDPDNYYT | -0.71 | Non-Toxin | -0.3 | 0 | 984.08 |
| 2428 | PDPDNYYC | -0.5 | Non-Toxin | -0.27 | 0 | 986.11 |
| 2429 | PDPDNYYN | -0.71 | Non-Toxin | -0.35 | 0 | 997.08 |
| 2430 | PDPDNYYQ | -0.58 | Non-Toxin | -0.36 | 0.16 | 1011.11 |
| 2431 | PDPDQFFS | -0.64 | Non-Toxin | -0.16 | 0.16 | 952.08 |
| 2432 | PDPDQFFT | -0.58 | Non-Toxin | -0.15 | 0.16 | 966.11 |
| 2433 | PDPDQFFC | -0.34 | Non-Toxin | -0.13 | 0.16 | 968.14 |
| 2434 | PDPDQFFN | -0.58 | Non-Toxin | -0.21 | 0.16 | 979.11 |
| 2435 | PDPDQFFQ | -0.65 | Non-Toxin | -0.22 | 0.31 | 993.14 |
| 2436 | PDPDQFWS | -0.72 | Non-Toxin | -0.19 | 0.16 | 991.12 |
| 2437 | PDPDQFWT | -0.71 | Non-Toxin | -0.18 | 0.16 | 1005.15 |
| 2438 | PDPDQFWC | -0.35 | Non-Toxin | -0.16 | 0.16 | 1007.18 |
| 2439 | PDPDQFWN | -0.77 | Non-Toxin | -0.24 | 0.16 | 1018.15 |
| 2440 | PDPDQFWQ | -0.71 | Non-Toxin | -0.25 | 0.31 | 1032.18 |
| 2441 | PDPDQFYS | -0.76 | Non-Toxin | -0.24 | 0.16 | 968.08 |
| 2442 | PDPDQFYT | -0.81 | Non-Toxin | -0.23 | 0.16 | 982.11 |
| 2443 | PDPDQFYC | -0.69 | Non-Toxin | -0.2 | 0.16 | 984.14 |
| 2444 | PDPDQFYN | -0.81 | Non-Toxin | -0.29 | 0.16 | 995.11 |
| 2445 | PDPDQFYQ | -0.68 | Non-Toxin | -0.29 | 0.31 | 1009.14 |
| 2446 | PDPDQWFS | -0.7 | Non-Toxin | -0.19 | 0.16 | 991.12 |
| 2447 | PDPDQWFT | -0.66 | Non-Toxin | -0.18 | 0.16 | 1005.15 |
| 2448 | PDPDQWFC | -0.43 | Non-Toxin | -0.16 | 0.16 | 1007.18 |
| 2449 | PDPDQWFN | -0.66 | Non-Toxin | -0.24 | 0.16 | 1018.15 |
| 2450 | PDPDQWFQ | -0.71 | Non-Toxin | -0.25 | 0.31 | 1032.18 |
| 2451 | PDPDQWWS | -0.65 | Non-Toxin | -0.22 | 0.16 | 1030.16 |
| 2452 | PDPDQWWT | -0.65 | Non-Toxin | -0.21 | 0.16 | 1044.19 |
| 2453 | PDPDQWWC | -0.28 | Non-Toxin | -0.19 | 0.16 | 1046.22 |
| 2454 | PDPDQWWN | -0.7 | Non-Toxin | -0.27 | 0.16 | 1057.19 |
| 2455 | PDPDQWWQ | -0.64 | Non-Toxin | -0.28 | 0.31 | 1071.22 |
| 2456 | PDPDQWYS | -0.51 | Non-Toxin | -0.27 | 0.16 | 1007.12 |
| 2457 | PDPDQWYT | -0.58 | Non-Toxin | -0.26 | 0.16 | 1021.15 |
| 2458 | PDPDQWYC | -0.42 | Non-Toxin | -0.23 | 0.16 | 1023.18 |
| 2459 | PDPDQWYN | -0.55 | Non-Toxin | -0.32 | 0.16 | 1034.15 |
| 2460 | PDPDQWYQ | -0.44 | Non-Toxin | -0.32 | 0.31 | 1048.18 |
| 2461 | PDPDQYFS | -0.8 | Non-Toxin | -0.24 | 0.16 | 968.08 |
| 2462 | PDPDQYFT | -0.73 | Non-Toxin | -0.23 | 0.16 | 982.11 |
| 2463 | PDPDQYFC | -0.53 | Non-Toxin | -0.2 | 0.16 | 984.14 |
| 2464 | PDPDQYFN | -0.77 | Non-Toxin | -0.29 | 0.16 | 995.11 |
| 2465 | PDPDQYFQ | -0.82 | Non-Toxin | -0.29 | 0.31 | 1009.14 |
| 2466 | PDPDQYWS | -0.8 | Non-Toxin | -0.27 | 0.16 | 1007.12 |
| 2467 | PDPDQYWT | -0.8 | Non-Toxin | -0.26 | 0.16 | 1021.15 |
| 2468 | PDPDQYWC | -0.43 | Non-Toxin | -0.23 | 0.16 | 1023.18 |
| 2469 | PDPDQYWN | -0.86 | Non-Toxin | -0.32 | 0.16 | 1034.15 |
| 2470 | PDPDQYWQ | -0.78 | Non-Toxin | -0.32 | 0.31 | 1048.18 |
| 2471 | PDPDQYYS | -0.69 | Non-Toxin | -0.31 | 0.16 | 984.08 |
| 2472 | PDPDQYYT | -0.74 | Non-Toxin | -0.3 | 0.16 | 998.11 |
| 2473 | PDPDQYYC | -0.56 | Non-Toxin | -0.27 | 0.16 | 1000.14 |
| 2474 | PDPDQYYN | -0.73 | Non-Toxin | -0.36 | 0.16 | 1011.11 |
| 2475 | PDPDQYYQ | -0.62 | Non-Toxin | -0.36 | 0.31 | 1025.14 |
| 2476 | PDPESFFS | -0.76 | Non-Toxin | -0.1 | 0.16 | 925.05 |
| 2477 | PDPESFFT | -0.7 | Non-Toxin | -0.09 | 0.16 | 939.08 |
| 2478 | PDPESFFC | -0.33 | Non-Toxin | -0.06 | 0.16 | 941.11 |
| 2479 | PDPESFFN | -0.74 | Non-Toxin | -0.14 | 0.16 | 952.08 |
| 2480 | PDPESFFQ | -0.72 | Non-Toxin | -0.15 | 0.32 | 966.11 |
| 2481 | PDPESFWS | -0.95 | Non-Toxin | -0.13 | 0.16 | 964.09 |
| 2482 | PDPESFWT | -0.86 | Non-Toxin | -0.12 | 0.16 | 978.12 |
| 2483 | PDPESFWC | -0.32 | Non-Toxin | -0.09 | 0.16 | 980.15 |
| 2484 | PDPESFWN | -0.97 | Non-Toxin | -0.17 | 0.16 | 991.12 |
| 2485 | PDPESFWQ | -0.87 | Non-Toxin | -0.18 | 0.32 | 1005.15 |
| 2486 | PDPESFYS | -1.05 | Non-Toxin | -0.17 | 0.16 | 941.05 |
| 2487 | PDPESFYT | -1.08 | Non-Toxin | -0.16 | 0.16 | 955.08 |
| 2488 | PDPESFYC | -0.89 | Non-Toxin | -0.13 | 0.16 | 957.11 |
| 2489 | PDPESFYN | -1.05 | Non-Toxin | -0.22 | 0.16 | 968.08 |
| 2490 | PDPESFYQ | -0.87 | Non-Toxin | -0.22 | 0.32 | 982.11 |
| 2491 | PDPESWFS | -1.07 | Non-Toxin | -0.13 | 0.16 | 964.09 |
| 2492 | PDPESWFT | -1.01 | Non-Toxin | -0.12 | 0.16 | 978.12 |
| 2493 | PDPESWFC | -0.63 | Non-Toxin | -0.09 | 0.16 | 980.15 |
| 2494 | PDPESWFN | -1.01 | Non-Toxin | -0.18 | 0.16 | 991.12 |
| 2495 | PDPESWFQ | -1.03 | Non-Toxin | -0.18 | 0.32 | 1005.15 |
| 2496 | PDPESWWS | -1 | Non-Toxin | -0.16 | 0.16 | 1003.13 |
| 2497 | PDPESWWT | -0.97 | Non-Toxin | -0.15 | 0.16 | 1017.16 |
| 2498 | PDPESWWC | -0.44 | Non-Toxin | -0.12 | 0.16 | 1019.19 |
| 2499 | PDPESWWN | -1.06 | Non-Toxin | -0.21 | 0.16 | 1030.16 |
| 2500 | PDPESWWQ | -0.95 | Non-Toxin | -0.21 | 0.32 | 1044.19 |
| 2501 | PDPESWYS | -0.81 | Non-Toxin | -0.2 | 0.16 | 980.09 |
| 2502 | PDPESWYT | -0.87 | Non-Toxin | -0.19 | 0.16 | 994.12 |
| 2503 | PDPESWYC | -0.61 | Non-Toxin | -0.16 | 0.16 | 996.15 |
| 2504 | PDPESWYN | -0.82 | Non-Toxin | -0.25 | 0.16 | 1007.12 |
| 2505 | PDPESWYQ | -0.62 | Non-Toxin | -0.26 | 0.32 | 1021.15 |
| 2506 | PDPESYFS | -0.93 | Non-Toxin | -0.17 | 0.16 | 941.05 |
| 2507 | PDPESYFT | -0.86 | Non-Toxin | -0.16 | 0.16 | 955.08 |
| 2508 | PDPESYFC | -0.5 | Non-Toxin | -0.13 | 0.16 | 957.11 |
| 2509 | PDPESYFN | -0.91 | Non-Toxin | -0.22 | 0.16 | 968.08 |
| 2510 | PDPESYFQ | -0.92 | Non-Toxin | -0.22 | 0.32 | 982.11 |
| 2511 | PDPESYWS | -0.98 | Non-Toxin | -0.2 | 0.16 | 980.09 |
| 2512 | PDPESYWT | -0.96 | Non-Toxin | -0.19 | 0.16 | 994.12 |
| 2513 | PDPESYWC | -0.42 | Non-Toxin | -0.16 | 0.16 | 996.15 |
| 2514 | PDPESYWN | -1.05 | Non-Toxin | -0.25 | 0.16 | 1007.12 |
| 2515 | PDPESYWQ | -0.93 | Non-Toxin | -0.26 | 0.32 | 1021.15 |
| 2516 | PDPESYYS | -0.79 | Non-Toxin | -0.24 | 0.16 | 957.05 |
| 2517 | PDPESYYT | -0.85 | Non-Toxin | -0.23 | 0.16 | 971.08 |
| 2518 | PDPESYYC | -0.52 | Non-Toxin | -0.21 | 0.16 | 973.11 |
| 2519 | PDPESYYN | -0.8 | Non-Toxin | -0.29 | 0.16 | 984.08 |
| 2520 | PDPESYYQ | -0.59 | Non-Toxin | -0.3 | 0.32 | 998.11 |
| 2521 | PDPETFFS | -0.91 | Non-Toxin | -0.09 | 0.16 | 939.08 |
| 2522 | PDPETFFT | -0.88 | Non-Toxin | -0.08 | 0.16 | 953.11 |
| 2523 | PDPETFFC | -0.48 | Non-Toxin | -0.05 | 0.16 | 955.14 |
| 2524 | PDPETFFN | -0.82 | Non-Toxin | -0.13 | 0.16 | 966.11 |
| 2525 | PDPETFFQ | -0.9 | Non-Toxin | -0.14 | 0.32 | 980.14 |
| 2526 | PDPETFWS | -1.03 | Non-Toxin | -0.12 | 0.16 | 978.12 |
| 2527 | PDPETFWT | -0.99 | Non-Toxin | -0.11 | 0.16 | 992.15 |
| 2528 | PDPETFWC | -0.47 | Non-Toxin | -0.08 | 0.16 | 994.18 |
| 2529 | PDPETFWN | -1.1 | Non-Toxin | -0.16 | 0.16 | 1005.15 |
| 2530 | PDPETFWQ | -0.99 | Non-Toxin | -0.17 | 0.32 | 1019.18 |
| 2531 | PDPETFYS | -1.13 | Non-Toxin | -0.16 | 0.16 | 955.08 |
| 2532 | PDPETFYT | -1.17 | Non-Toxin | -0.15 | 0.16 | 969.11 |
| 2533 | PDPETFYC | -0.99 | Non-Toxin | -0.12 | 0.16 | 971.14 |
| 2534 | PDPETFYN | -1.15 | Non-Toxin | -0.21 | 0.16 | 982.11 |
| 2535 | PDPETFYQ | -0.98 | Non-Toxin | -0.21 | 0.32 | 996.14 |
| 2536 | PDPETWFS | -1.01 | Non-Toxin | -0.12 | 0.16 | 978.12 |
| 2537 | PDPETWFT | -0.98 | Non-Toxin | -0.11 | 0.16 | 992.15 |
| 2538 | PDPETWFC | -0.6 | Non-Toxin | -0.08 | 0.16 | 994.18 |
| 2539 | PDPETWFN | -0.95 | Non-Toxin | -0.17 | 0.16 | 1005.15 |
| 2540 | PDPETWFQ | -1 | Non-Toxin | -0.17 | 0.32 | 1019.18 |
| 2541 | PDPETWWS | -0.95 | Non-Toxin | -0.15 | 0.16 | 1017.16 |
| 2542 | PDPETWWT | -0.94 | Non-Toxin | -0.14 | 0.16 | 1031.19 |
| 2543 | PDPETWWC | -0.37 | Non-Toxin | -0.11 | 0.16 | 1033.22 |
| 2544 | PDPETWWN | -1.02 | Non-Toxin | -0.2 | 0.16 | 1044.19 |
| 2545 | PDPETWWQ | -0.91 | Non-Toxin | -0.2 | 0.32 | 1058.22 |
| 2546 | PDPETWYS | -0.8 | Non-Toxin | -0.19 | 0.16 | 994.12 |
| 2547 | PDPETWYT | -0.83 | Non-Toxin | -0.18 | 0.16 | 1008.15 |
| 2548 | PDPETWYC | -0.59 | Non-Toxin | -0.15 | 0.16 | 1010.18 |
| 2549 | PDPETWYN | -0.8 | Non-Toxin | -0.24 | 0.16 | 1021.15 |
| 2550 | PDPETWYQ | -0.61 | Non-Toxin | -0.24 | 0.32 | 1035.18 |
| 2551 | PDPETYFS | -0.87 | Non-Toxin | -0.16 | 0.16 | 955.08 |
| 2552 | PDPETYFT | -0.82 | Non-Toxin | -0.15 | 0.16 | 969.11 |
| 2553 | PDPETYFC | -0.47 | Non-Toxin | -0.12 | 0.16 | 971.14 |
| 2554 | PDPETYFN | -0.86 | Non-Toxin | -0.21 | 0.16 | 982.11 |
| 2555 | PDPETYFQ | -0.88 | Non-Toxin | -0.21 | 0.32 | 996.14 |
| 2556 | PDPETYWS | -0.92 | Non-Toxin | -0.19 | 0.16 | 994.12 |
| 2557 | PDPETYWT | -0.92 | Non-Toxin | -0.18 | 0.16 | 1008.15 |
| 2558 | PDPETYWC | -0.36 | Non-Toxin | -0.15 | 0.16 | 1010.18 |
| 2559 | PDPETYWN | -1 | Non-Toxin | -0.24 | 0.16 | 1021.15 |
| 2560 | PDPETYWQ | -0.89 | Non-Toxin | -0.24 | 0.32 | 1035.18 |
| 2561 | PDPETYYS | -0.8 | Non-Toxin | -0.23 | 0.16 | 971.08 |
| 2562 | PDPETYYT | -0.8 | Non-Toxin | -0.22 | 0.16 | 985.11 |
| 2563 | PDPETYYC | -0.54 | Non-Toxin | -0.2 | 0.16 | 987.14 |
| 2564 | PDPETYYN | -0.8 | Non-Toxin | -0.28 | 0.16 | 998.11 |
| 2565 | PDPETYYQ | -0.6 | Non-Toxin | -0.29 | 0.32 | 1012.14 |
| 2566 | PDPECFFS | -0.4 | Non-Toxin | -0.06 | 0.16 | 941.11 |
| 2567 | PDPECFFT | -0.3 | Non-Toxin | -0.05 | 0.16 | 955.14 |
| 2568 | PDPECFFC | 0.12 | Toxin | -0.02 | 0.16 | 957.17 |
| 2569 | PDPECFFN | -0.32 | Non-Toxin | -0.11 | 0.16 | 968.14 |
| 2570 | PDPECFFQ | -0.39 | Non-Toxin | -0.11 | 0.32 | 982.17 |
| 2571 | PDPECFWS | -0.65 | Non-Toxin | -0.09 | 0.16 | 980.15 |
| 2572 | PDPECFWT | -0.64 | Non-Toxin | -0.08 | 0.16 | 994.18 |
| 2573 | PDPECFWC | -0.09 | Non-Toxin | -0.05 | 0.16 | 996.21 |
| 2574 | PDPECFWN | -0.72 | Non-Toxin | -0.14 | 0.16 | 1007.18 |
| 2575 | PDPECFWQ | -0.61 | Non-Toxin | -0.14 | 0.32 | 1021.21 |
| 2576 | PDPECFYS | -0.65 | Non-Toxin | -0.13 | 0.16 | 957.11 |
| 2577 | PDPECFYT | -0.68 | Non-Toxin | -0.12 | 0.16 | 971.14 |
| 2578 | PDPECFYC | -0.59 | Non-Toxin | -0.1 | 0.16 | 973.17 |
| 2579 | PDPECFYN | -0.66 | Non-Toxin | -0.18 | 0.16 | 984.14 |
| 2580 | PDPECFYQ | -0.5 | Non-Toxin | -0.19 | 0.32 | 998.17 |
| 2581 | PDPECWFS | -0.42 | Non-Toxin | -0.09 | 0.16 | 980.15 |
| 2582 | PDPECWFT | -0.32 | Non-Toxin | -0.08 | 0.16 | 994.18 |
| 2583 | PDPECWFC | 0.06 | Toxin | -0.05 | 0.16 | 996.21 |
| 2584 | PDPECWFN | -0.32 | Non-Toxin | -0.14 | 0.16 | 1007.18 |
| 2585 | PDPECWFQ | -0.39 | Non-Toxin | -0.14 | 0.32 | 1021.21 |
| 2586 | PDPECWWS | -0.31 | Non-Toxin | -0.12 | 0.16 | 1019.19 |
| 2587 | PDPECWWT | -0.31 | Non-Toxin | -0.11 | 0.16 | 1033.22 |
| 2588 | PDPECWWC | 0.26 | Toxin | -0.08 | 0.16 | 1035.25 |
| 2589 | PDPECWWN | -0.38 | Non-Toxin | -0.17 | 0.16 | 1046.22 |
| 2590 | PDPECWWQ | -0.28 | Non-Toxin | -0.17 | 0.32 | 1060.25 |
| 2591 | PDPECWYS | -0.16 | Non-Toxin | -0.16 | 0.16 | 996.15 |
| 2592 | PDPECWYT | -0.16 | Non-Toxin | -0.15 | 0.16 | 1010.18 |
| 2593 | PDPECWYC | 0.04 | Toxin | -0.13 | 0.16 | 1012.21 |
| 2594 | PDPECWYN | -0.15 | Non-Toxin | -0.21 | 0.16 | 1023.18 |
| 2595 | PDPECWYQ | 0.04 | Toxin | -0.22 | 0.32 | 1037.21 |
| 2596 | PDPECYFS | -0.33 | Non-Toxin | -0.13 | 0.16 | 957.11 |
| 2597 | PDPECYFT | -0.21 | Non-Toxin | -0.12 | 0.16 | 971.14 |
| 2598 | PDPECYFC | 0.12 | Toxin | -0.1 | 0.16 | 973.17 |
| 2599 | PDPECYFN | -0.25 | Non-Toxin | -0.18 | 0.16 | 984.14 |
| 2600 | PDPECYFQ | -0.32 | Non-Toxin | -0.19 | 0.32 | 998.17 |
| 2601 | PDPECYWS | -0.26 | Non-Toxin | -0.16 | 0.16 | 996.15 |
| 2602 | PDPECYWT | -0.27 | Non-Toxin | -0.15 | 0.16 | 1010.18 |
| 2603 | PDPECYWC | 0.26 | Toxin | -0.13 | 0.16 | 1012.21 |
| 2604 | PDPECYWN | -0.37 | Non-Toxin | -0.21 | 0.16 | 1023.18 |
| 2605 | PDPECYWQ | -0.24 | Non-Toxin | -0.22 | 0.32 | 1037.21 |
| 2606 | PDPECYYS | -0.12 | Non-Toxin | -0.21 | 0.16 | 973.11 |
| 2607 | PDPECYYT | -0.18 | Non-Toxin | -0.2 | 0.16 | 987.14 |
| 2608 | PDPECYYC | 0.08 | Toxin | -0.17 | 0.16 | 989.17 |
| 2609 | PDPECYYN | -0.11 | Non-Toxin | -0.26 | 0.16 | 1000.14 |
| 2610 | PDPECYYQ | 0.04 | Toxin | -0.26 | 0.32 | 1014.17 |
| 2611 | PDPENFFS | -1.05 | Non-Toxin | -0.15 | 0.16 | 952.08 |
| 2612 | PDPENFFT | -0.95 | Non-Toxin | -0.14 | 0.16 | 966.11 |
| 2613 | PDPENFFC | -0.58 | Non-Toxin | -0.11 | 0.16 | 968.14 |
| 2614 | PDPENFFN | -0.95 | Non-Toxin | -0.19 | 0.16 | 979.11 |
| 2615 | PDPENFFQ | -1 | Non-Toxin | -0.2 | 0.32 | 993.14 |
| 2616 | PDPENFWS | -1.16 | Non-Toxin | -0.18 | 0.16 | 991.12 |
| 2617 | PDPENFWT | -1.13 | Non-Toxin | -0.17 | 0.16 | 1005.15 |
| 2618 | PDPENFWC | -0.62 | Non-Toxin | -0.14 | 0.16 | 1007.18 |
| 2619 | PDPENFWN | -1.23 | Non-Toxin | -0.22 | 0.16 | 1018.15 |
| 2620 | PDPENFWQ | -1.13 | Non-Toxin | -0.23 | 0.32 | 1032.18 |
| 2621 | PDPENFYS | -1.22 | Non-Toxin | -0.22 | 0.16 | 968.08 |
| 2622 | PDPENFYT | -1.25 | Non-Toxin | -0.21 | 0.16 | 982.11 |
| 2623 | PDPENFYC | -1.07 | Non-Toxin | -0.18 | 0.16 | 984.14 |
| 2624 | PDPENFYN | -1.25 | Non-Toxin | -0.27 | 0.16 | 995.11 |
| 2625 | PDPENFYQ | -1.09 | Non-Toxin | -0.27 | 0.32 | 1009.14 |
| 2626 | PDPENWFS | -0.95 | Non-Toxin | -0.18 | 0.16 | 991.12 |
| 2627 | PDPENWFT | -0.87 | Non-Toxin | -0.17 | 0.16 | 1005.15 |
| 2628 | PDPENWFC | -0.51 | Non-Toxin | -0.14 | 0.16 | 1007.18 |
| 2629 | PDPENWFN | -0.87 | Non-Toxin | -0.22 | 0.16 | 1018.15 |
| 2630 | PDPENWFQ | -0.91 | Non-Toxin | -0.23 | 0.32 | 1032.18 |
| 2631 | PDPENWWS | -0.87 | Non-Toxin | -0.2 | 0.16 | 1030.16 |
| 2632 | PDPENWWT | -0.85 | Non-Toxin | -0.19 | 0.16 | 1044.19 |
| 2633 | PDPENWWC | -0.32 | Non-Toxin | -0.17 | 0.16 | 1046.22 |
| 2634 | PDPENWWN | -0.94 | Non-Toxin | -0.25 | 0.16 | 1057.19 |
| 2635 | PDPENWWQ | -0.83 | Non-Toxin | -0.26 | 0.32 | 1071.22 |
| 2636 | PDPENWYS | -0.69 | Non-Toxin | -0.25 | 0.16 | 1007.12 |
| 2637 | PDPENWYT | -0.74 | Non-Toxin | -0.24 | 0.16 | 1021.15 |
| 2638 | PDPENWYC | -0.5 | Non-Toxin | -0.21 | 0.16 | 1023.18 |
| 2639 | PDPENWYN | -0.72 | Non-Toxin | -0.3 | 0.16 | 1034.15 |
| 2640 | PDPENWYQ | -0.53 | Non-Toxin | -0.3 | 0.32 | 1048.18 |
| 2641 | PDPENYFS | -0.84 | Non-Toxin | -0.22 | 0.16 | 968.08 |
| 2642 | PDPENYFT | -0.76 | Non-Toxin | -0.21 | 0.16 | 982.11 |
| 2643 | PDPENYFC | -0.4 | Non-Toxin | -0.18 | 0.16 | 984.14 |
| 2644 | PDPENYFN | -0.79 | Non-Toxin | -0.27 | 0.16 | 995.11 |
| 2645 | PDPENYFQ | -0.81 | Non-Toxin | -0.27 | 0.32 | 1009.14 |
| 2646 | PDPENYWS | -0.88 | Non-Toxin | -0.25 | 0.16 | 1007.12 |
| 2647 | PDPENYWT | -0.87 | Non-Toxin | -0.24 | 0.16 | 1021.15 |
| 2648 | PDPENYWC | -0.33 | Non-Toxin | -0.21 | 0.16 | 1023.18 |
| 2649 | PDPENYWN | -0.97 | Non-Toxin | -0.3 | 0.16 | 1034.15 |
| 2650 | PDPENYWQ | -0.85 | Non-Toxin | -0.3 | 0.32 | 1048.18 |
| 2651 | PDPENYYS | -0.71 | Non-Toxin | -0.29 | 0.16 | 984.08 |
| 2652 | PDPENYYT | -0.71 | Non-Toxin | -0.28 | 0.16 | 998.11 |
| 2653 | PDPENYYC | -0.38 | Non-Toxin | -0.26 | 0.16 | 1000.14 |
| 2654 | PDPENYYN | -0.74 | Non-Toxin | -0.34 | 0.16 | 1011.11 |
| 2655 | PDPENYYQ | -0.54 | Non-Toxin | -0.35 | 0.32 | 1025.14 |
| 2656 | PDPEQFFS | -0.81 | Non-Toxin | -0.15 | 0.32 | 966.11 |
| 2657 | PDPEQFFT | -0.7 | Non-Toxin | -0.14 | 0.32 | 980.14 |
| 2658 | PDPEQFFC | -0.33 | Non-Toxin | -0.11 | 0.32 | 982.17 |
| 2659 | PDPEQFFN | -0.7 | Non-Toxin | -0.2 | 0.32 | 993.14 |
| 2660 | PDPEQFFQ | -0.78 | Non-Toxin | -0.21 | 0.47 | 1007.17 |
| 2661 | PDPEQFWS | -0.94 | Non-Toxin | -0.18 | 0.32 | 1005.15 |
| 2662 | PDPEQFWT | -0.9 | Non-Toxin | -0.17 | 0.32 | 1019.18 |
| 2663 | PDPEQFWC | -0.38 | Non-Toxin | -0.14 | 0.32 | 1021.21 |
| 2664 | PDPEQFWN | -0.99 | Non-Toxin | -0.23 | 0.32 | 1032.18 |
| 2665 | PDPEQFWQ | -0.89 | Non-Toxin | -0.23 | 0.47 | 1046.21 |
| 2666 | PDPEQFYS | -1.02 | Non-Toxin | -0.23 | 0.32 | 982.11 |
| 2667 | PDPEQFYT | -1.03 | Non-Toxin | -0.21 | 0.32 | 996.14 |
| 2668 | PDPEQFYC | -0.85 | Non-Toxin | -0.19 | 0.32 | 998.17 |
| 2669 | PDPEQFYN | -1.03 | Non-Toxin | -0.27 | 0.32 | 1009.14 |
| 2670 | PDPEQFYQ | -0.85 | Non-Toxin | -0.28 | 0.47 | 1023.17 |
| 2671 | PDPEQWFS | -0.94 | Non-Toxin | -0.18 | 0.32 | 1005.15 |
| 2672 | PDPEQWFT | -0.85 | Non-Toxin | -0.17 | 0.32 | 1019.18 |
| 2673 | PDPEQWFC | -0.49 | Non-Toxin | -0.14 | 0.32 | 1021.21 |
| 2674 | PDPEQWFN | -0.86 | Non-Toxin | -0.23 | 0.32 | 1032.18 |
| 2675 | PDPEQWFQ | -0.9 | Non-Toxin | -0.23 | 0.47 | 1046.21 |
| 2676 | PDPEQWWS | -0.88 | Non-Toxin | -0.21 | 0.32 | 1044.19 |
| 2677 | PDPEQWWT | -0.85 | Non-Toxin | -0.2 | 0.32 | 1058.22 |
| 2678 | PDPEQWWC | -0.32 | Non-Toxin | -0.17 | 0.32 | 1060.25 |
| 2679 | PDPEQWWN | -0.93 | Non-Toxin | -0.26 | 0.32 | 1071.22 |
| 2680 | PDPEQWWQ | -0.83 | Non-Toxin | -0.26 | 0.47 | 1085.25 |
| 2681 | PDPEQWYS | -0.69 | Non-Toxin | -0.26 | 0.32 | 1021.15 |
| 2682 | PDPEQWYT | -0.72 | Non-Toxin | -0.24 | 0.32 | 1035.18 |
| 2683 | PDPEQWYC | -0.48 | Non-Toxin | -0.22 | 0.32 | 1037.21 |
| 2684 | PDPEQWYN | -0.69 | Non-Toxin | -0.3 | 0.32 | 1048.18 |
| 2685 | PDPEQWYQ | -0.53 | Non-Toxin | -0.31 | 0.47 | 1062.21 |
| 2686 | PDPEQYFS | -1.07 | Non-Toxin | -0.23 | 0.32 | 982.11 |
| 2687 | PDPEQYFT | -0.95 | Non-Toxin | -0.21 | 0.32 | 996.14 |
| 2688 | PDPEQYFC | -0.62 | Non-Toxin | -0.19 | 0.32 | 998.17 |
| 2689 | PDPEQYFN | -1 | Non-Toxin | -0.27 | 0.32 | 1009.14 |
| 2690 | PDPEQYFQ | -1.05 | Non-Toxin | -0.28 | 0.47 | 1023.17 |
| 2691 | PDPEQYWS | -1.09 | Non-Toxin | -0.26 | 0.32 | 1021.15 |
| 2692 | PDPEQYWT | -1.06 | Non-Toxin | -0.24 | 0.32 | 1035.18 |
| 2693 | PDPEQYWC | -0.52 | Non-Toxin | -0.22 | 0.32 | 1037.21 |
| 2694 | PDPEQYWN | -1.15 | Non-Toxin | -0.3 | 0.32 | 1048.18 |
| 2695 | PDPEQYWQ | -1.04 | Non-Toxin | -0.31 | 0.47 | 1062.21 |
| 2696 | PDPEQYYS | -0.95 | Non-Toxin | -0.3 | 0.32 | 998.11 |
| 2697 | PDPEQYYT | -0.95 | Non-Toxin | -0.29 | 0.32 | 1012.14 |
| 2698 | PDPEQYYC | -0.68 | Non-Toxin | -0.26 | 0.32 | 1014.17 |
| 2699 | PDPEQYYN | -0.94 | Non-Toxin | -0.35 | 0.32 | 1025.14 |
| 2700 | PDPEQYYQ | -0.78 | Non-Toxin | -0.35 | 0.47 | 1039.17 |
| 2701 | PEGDSFFS | -0.99 | Non-Toxin | -0.07 | 0.16 | 884.99 |
| 2702 | PEGDSFFT | -0.92 | Non-Toxin | -0.06 | 0.16 | 899.02 |
| 2703 | PEGDSFFC | -0.65 | Non-Toxin | -0.03 | 0.16 | 901.05 |
| 2704 | PEGDSFFN | -0.92 | Non-Toxin | -0.12 | 0.16 | 912.02 |
| 2705 | PEGDSFFQ | -0.95 | Non-Toxin | -0.12 | 0.32 | 926.05 |
| 2706 | PEGDSFWS | -1.13 | Non-Toxin | -0.1 | 0.16 | 924.03 |
| 2707 | PEGDSFWT | -1.03 | Non-Toxin | -0.09 | 0.16 | 938.06 |
| 2708 | PEGDSFWC | -0.51 | Non-Toxin | -0.06 | 0.16 | 940.09 |
| 2709 | PEGDSFWN | -1.14 | Non-Toxin | -0.15 | 0.16 | 951.06 |
| 2710 | PEGDSFWQ | -1.04 | Non-Toxin | -0.15 | 0.32 | 965.09 |
| 2711 | PEGDSFYS | -1.27 | Non-Toxin | -0.14 | 0.16 | 900.99 |
| 2712 | PEGDSFYT | -1.19 | Non-Toxin | -0.13 | 0.16 | 915.02 |
| 2713 | PEGDSFYC | -1.03 | Non-Toxin | -0.1 | 0.16 | 917.05 |
| 2714 | PEGDSFYN | -1.19 | Non-Toxin | -0.19 | 0.16 | 928.02 |
| 2715 | PEGDSFYQ | -1.01 | Non-Toxin | -0.2 | 0.32 | 942.05 |
| 2716 | PEGDSWFS | -1.26 | Non-Toxin | -0.1 | 0.16 | 924.03 |
| 2717 | PEGDSWFT | -1.18 | Non-Toxin | -0.09 | 0.16 | 938.06 |
| 2718 | PEGDSWFC | -0.91 | Non-Toxin | -0.06 | 0.16 | 940.09 |
| 2719 | PEGDSWFN | -1.16 | Non-Toxin | -0.15 | 0.16 | 951.06 |
| 2720 | PEGDSWFQ | -1.22 | Non-Toxin | -0.15 | 0.32 | 965.09 |
| 2721 | PEGDSWWS | -1.18 | Non-Toxin | -0.13 | 0.16 | 963.07 |
| 2722 | PEGDSWWT | -1.13 | Non-Toxin | -0.12 | 0.16 | 977.1 |
| 2723 | PEGDSWWC | -0.62 | Non-Toxin | -0.09 | 0.16 | 979.13 |
| 2724 | PEGDSWWN | -1.22 | Non-Toxin | -0.18 | 0.16 | 990.1 |
| 2725 | PEGDSWWQ | -1.12 | Non-Toxin | -0.18 | 0.32 | 1004.13 |
| 2726 | PEGDSWYS | -1.04 | Non-Toxin | -0.17 | 0.16 | 940.03 |
| 2727 | PEGDSWYT | -0.99 | Non-Toxin | -0.16 | 0.16 | 954.06 |
| 2728 | PEGDSWYC | -0.76 | Non-Toxin | -0.14 | 0.16 | 956.09 |
| 2729 | PEGDSWYN | -0.97 | Non-Toxin | -0.22 | 0.16 | 967.06 |
| 2730 | PEGDSWYQ | -0.77 | Non-Toxin | -0.23 | 0.32 | 981.09 |
| 2731 | PEGDSYFS | -1.08 | Non-Toxin | -0.14 | 0.16 | 900.99 |
| 2732 | PEGDSYFT | -0.98 | Non-Toxin | -0.13 | 0.16 | 915.02 |
| 2733 | PEGDSYFC | -0.73 | Non-Toxin | -0.1 | 0.16 | 917.05 |
| 2734 | PEGDSYFN | -1.01 | Non-Toxin | -0.19 | 0.16 | 928.02 |
| 2735 | PEGDSYFQ | -1.06 | Non-Toxin | -0.2 | 0.32 | 942.05 |
| 2736 | PEGDSYWS | -1.12 | Non-Toxin | -0.17 | 0.16 | 940.03 |
| 2737 | PEGDSYWT | -1.09 | Non-Toxin | -0.16 | 0.16 | 954.06 |
| 2738 | PEGDSYWC | -0.56 | Non-Toxin | -0.14 | 0.16 | 956.09 |
| 2739 | PEGDSYWN | -1.18 | Non-Toxin | -0.22 | 0.16 | 967.06 |
| 2740 | PEGDSYWQ | -1.07 | Non-Toxin | -0.23 | 0.32 | 981.09 |
| 2741 | PEGDSYYS | -0.98 | Non-Toxin | -0.22 | 0.16 | 916.99 |
| 2742 | PEGDSYYT | -0.93 | Non-Toxin | -0.21 | 0.16 | 931.02 |
| 2743 | PEGDSYYC | -0.63 | Non-Toxin | -0.18 | 0.16 | 933.05 |
| 2744 | PEGDSYYN | -0.92 | Non-Toxin | -0.26 | 0.16 | 944.02 |
| 2745 | PEGDSYYQ | -0.7 | Non-Toxin | -0.27 | 0.32 | 958.05 |
| 2746 | PEGDTFFS | -0.9 | Non-Toxin | -0.06 | 0.16 | 899.02 |
| 2747 | PEGDTFFT | -0.8 | Non-Toxin | -0.05 | 0.16 | 913.05 |
| 2748 | PEGDTFFC | -0.47 | Non-Toxin | -0.02 | 0.16 | 915.08 |
| 2749 | PEGDTFFN | -0.76 | Non-Toxin | -0.11 | 0.16 | 926.05 |
| 2750 | PEGDTFFQ | -0.87 | Non-Toxin | -0.11 | 0.32 | 940.08 |
| 2751 | PEGDTFWS | -0.97 | Non-Toxin | -0.09 | 0.16 | 938.06 |
| 2752 | PEGDTFWT | -0.92 | Non-Toxin | -0.08 | 0.16 | 952.09 |
| 2753 | PEGDTFWC | -0.43 | Non-Toxin | -0.05 | 0.16 | 954.12 |
| 2754 | PEGDTFWN | -1.03 | Non-Toxin | -0.14 | 0.16 | 965.09 |
| 2755 | PEGDTFWQ | -0.93 | Non-Toxin | -0.14 | 0.32 | 979.12 |
| 2756 | PEGDTFYS | -1.09 | Non-Toxin | -0.13 | 0.16 | 915.02 |
| 2757 | PEGDTFYT | -1.04 | Non-Toxin | -0.12 | 0.16 | 929.05 |
| 2758 | PEGDTFYC | -0.81 | Non-Toxin | -0.09 | 0.16 | 931.08 |
| 2759 | PEGDTFYN | -1.05 | Non-Toxin | -0.18 | 0.16 | 942.05 |
| 2760 | PEGDTFYQ | -0.86 | Non-Toxin | -0.19 | 0.32 | 956.08 |
| 2761 | PEGDTWFS | -0.96 | Non-Toxin | -0.09 | 0.16 | 938.06 |
| 2762 | PEGDTWFT | -0.86 | Non-Toxin | -0.08 | 0.16 | 952.09 |
| 2763 | PEGDTWFC | -0.54 | Non-Toxin | -0.05 | 0.16 | 954.12 |
| 2764 | PEGDTWFN | -0.85 | Non-Toxin | -0.14 | 0.16 | 965.09 |
| 2765 | PEGDTWFQ | -0.92 | Non-Toxin | -0.14 | 0.32 | 979.12 |
| 2766 | PEGDTWWS | -0.88 | Non-Toxin | -0.12 | 0.16 | 977.1 |
| 2767 | PEGDTWWT | -0.86 | Non-Toxin | -0.11 | 0.16 | 991.13 |
| 2768 | PEGDTWWC | -0.32 | Non-Toxin | -0.08 | 0.16 | 993.16 |
| 2769 | PEGDTWWN | -0.94 | Non-Toxin | -0.17 | 0.16 | 1004.13 |
| 2770 | PEGDTWWQ | -0.84 | Non-Toxin | -0.17 | 0.32 | 1018.16 |
| 2771 | PEGDTWYS | -0.78 | Non-Toxin | -0.16 | 0.16 | 954.06 |
| 2772 | PEGDTWYT | -0.73 | Non-Toxin | -0.15 | 0.16 | 968.09 |
| 2773 | PEGDTWYC | -0.45 | Non-Toxin | -0.12 | 0.16 | 970.12 |
| 2774 | PEGDTWYN | -0.73 | Non-Toxin | -0.21 | 0.16 | 981.09 |
| 2775 | PEGDTWYQ | -0.52 | Non-Toxin | -0.22 | 0.32 | 995.12 |
| 2776 | PEGDTYFS | -0.84 | Non-Toxin | -0.13 | 0.16 | 915.02 |
| 2777 | PEGDTYFT | -0.71 | Non-Toxin | -0.12 | 0.16 | 929.05 |
| 2778 | PEGDTYFC | -0.44 | Non-Toxin | -0.09 | 0.16 | 931.08 |
| 2779 | PEGDTYFN | -0.78 | Non-Toxin | -0.18 | 0.16 | 942.05 |
| 2780 | PEGDTYFQ | -0.83 | Non-Toxin | -0.19 | 0.32 | 956.08 |
| 2781 | PEGDTYWS | -0.88 | Non-Toxin | -0.16 | 0.16 | 954.06 |
| 2782 | PEGDTYWT | -0.87 | Non-Toxin | -0.15 | 0.16 | 968.09 |
| 2783 | PEGDTYWC | -0.35 | Non-Toxin | -0.12 | 0.16 | 970.12 |
| 2784 | PEGDTYWN | -0.96 | Non-Toxin | -0.21 | 0.16 | 981.09 |
| 2785 | PEGDTYWQ | -0.85 | Non-Toxin | -0.22 | 0.32 | 995.12 |
| 2786 | PEGDTYYS | -0.82 | Non-Toxin | -0.21 | 0.16 | 931.02 |
| 2787 | PEGDTYYT | -0.73 | Non-Toxin | -0.2 | 0.16 | 945.05 |
| 2788 | PEGDTYYC | -0.44 | Non-Toxin | -0.17 | 0.16 | 947.08 |
| 2789 | PEGDTYYN | -0.77 | Non-Toxin | -0.25 | 0.16 | 958.05 |
| 2790 | PEGDTYYQ | -0.55 | Non-Toxin | -0.26 | 0.32 | 972.08 |
| 2791 | PEGDCFFS | -0.52 | Non-Toxin | -0.03 | 0.16 | 901.05 |
| 2792 | PEGDCFFT | -0.42 | Non-Toxin | -0.02 | 0.16 | 915.08 |
| 2793 | PEGDCFFC | -0.05 | Non-Toxin | 0.01 | 0.16 | 917.11 |
| 2794 | PEGDCFFN | -0.41 | Non-Toxin | -0.08 | 0.16 | 928.08 |
| 2795 | PEGDCFFQ | -0.5 | Non-Toxin | -0.08 | 0.32 | 942.11 |
| 2796 | PEGDCFWS | -0.73 | Non-Toxin | -0.06 | 0.16 | 940.09 |
| 2797 | PEGDCFWT | -0.71 | Non-Toxin | -0.05 | 0.16 | 954.12 |
| 2798 | PEGDCFWC | -0.19 | Non-Toxin | -0.02 | 0.16 | 956.15 |
| 2799 | PEGDCFWN | -0.79 | Non-Toxin | -0.11 | 0.16 | 967.12 |
| 2800 | PEGDCFWQ | -0.69 | Non-Toxin | -0.11 | 0.32 | 981.15 |
| 2801 | PEGDCFYS | -0.79 | Non-Toxin | -0.1 | 0.16 | 917.05 |
| 2802 | PEGDCFYT | -0.76 | Non-Toxin | -0.1 | 0.16 | 931.08 |
| 2803 | PEGDCFYC | -0.61 | Non-Toxin | -0.07 | 0.16 | 933.11 |
| 2804 | PEGDCFYN | -0.76 | Non-Toxin | -0.15 | 0.16 | 944.08 |
| 2805 | PEGDCFYQ | -0.56 | Non-Toxin | -0.16 | 0.32 | 958.11 |
| 2806 | PEGDCWFS | -0.5 | Non-Toxin | -0.06 | 0.16 | 940.09 |
| 2807 | PEGDCWFT | -0.4 | Non-Toxin | -0.05 | 0.16 | 954.12 |
| 2808 | PEGDCWFC | -0.06 | Non-Toxin | -0.02 | 0.16 | 956.15 |
| 2809 | PEGDCWFN | -0.36 | Non-Toxin | -0.11 | 0.16 | 967.12 |
| 2810 | PEGDCWFQ | -0.46 | Non-Toxin | -0.11 | 0.32 | 981.15 |
| 2811 | PEGDCWWS | -0.41 | Non-Toxin | -0.09 | 0.16 | 979.13 |
| 2812 | PEGDCWWT | -0.4 | Non-Toxin | -0.08 | 0.16 | 993.16 |
| 2813 | PEGDCWWC | 0.13 | Toxin | -0.05 | 0.16 | 995.19 |
| 2814 | PEGDCWWN | -0.48 | Non-Toxin | -0.14 | 0.16 | 1006.16 |
| 2815 | PEGDCWWQ | -0.38 | Non-Toxin | -0.14 | 0.32 | 1020.19 |
| 2816 | PEGDCWYS | -0.33 | Non-Toxin | -0.14 | 0.16 | 956.09 |
| 2817 | PEGDCWYT | -0.27 | Non-Toxin | -0.12 | 0.16 | 970.12 |
| 2818 | PEGDCWYC | -0.02 | Non-Toxin | -0.1 | 0.16 | 972.15 |
| 2819 | PEGDCWYN | -0.28 | Non-Toxin | -0.18 | 0.16 | 983.12 |
| 2820 | PEGDCWYQ | -0.06 | Non-Toxin | -0.19 | 0.32 | 997.15 |
| 2821 | PEGDCYFS | -0.34 | Non-Toxin | -0.1 | 0.16 | 917.05 |
| 2822 | PEGDCYFT | -0.22 | Non-Toxin | -0.1 | 0.16 | 931.08 |
| 2823 | PEGDCYFC | 0.08 | Toxin | -0.07 | 0.16 | 933.11 |
| 2824 | PEGDCYFN | -0.23 | Non-Toxin | -0.15 | 0.16 | 944.08 |
| 2825 | PEGDCYFQ | -0.32 | Non-Toxin | -0.16 | 0.32 | 958.11 |
| 2826 | PEGDCYWS | -0.26 | Non-Toxin | -0.14 | 0.16 | 956.09 |
| 2827 | PEGDCYWT | -0.26 | Non-Toxin | -0.12 | 0.16 | 970.12 |
| 2828 | PEGDCYWC | 0.23 | Toxin | -0.1 | 0.16 | 972.15 |
| 2829 | PEGDCYWN | -0.37 | Non-Toxin | -0.18 | 0.16 | 983.12 |
| 2830 | PEGDCYWQ | -0.25 | Non-Toxin | -0.19 | 0.32 | 997.15 |
| 2831 | PEGDCYYS | -0.23 | Non-Toxin | -0.18 | 0.16 | 933.05 |
| 2832 | PEGDCYYT | -0.23 | Non-Toxin | -0.17 | 0.16 | 947.08 |
| 2833 | PEGDCYYC | 0.08 | Toxin | -0.14 | 0.16 | 949.11 |
| 2834 | PEGDCYYN | -0.18 | Non-Toxin | -0.23 | 0.16 | 960.08 |
| 2835 | PEGDCYYQ | 0 | Toxin | -0.23 | 0.32 | 974.11 |
| 2836 | PEGDNFFS | -1.14 | Non-Toxin | -0.12 | 0.16 | 912.02 |
| 2837 | PEGDNFFT | -1.04 | Non-Toxin | -0.11 | 0.16 | 926.05 |
| 2838 | PEGDNFFC | -0.72 | Non-Toxin | -0.08 | 0.16 | 928.08 |
| 2839 | PEGDNFFN | -1.03 | Non-Toxin | -0.16 | 0.16 | 939.05 |
| 2840 | PEGDNFFQ | -1.11 | Non-Toxin | -0.17 | 0.32 | 953.08 |
| 2841 | PEGDNFWS | -1.22 | Non-Toxin | -0.15 | 0.16 | 951.06 |
| 2842 | PEGDNFWT | -1.19 | Non-Toxin | -0.14 | 0.16 | 965.09 |
| 2843 | PEGDNFWC | -0.7 | Non-Toxin | -0.11 | 0.16 | 967.12 |
| 2844 | PEGDNFWN | -1.28 | Non-Toxin | -0.19 | 0.16 | 978.09 |
| 2845 | PEGDNFWQ | -1.19 | Non-Toxin | -0.2 | 0.32 | 992.12 |
| 2846 | PEGDNFYS | -1.37 | Non-Toxin | -0.19 | 0.16 | 928.02 |
| 2847 | PEGDNFYT | -1.28 | Non-Toxin | -0.18 | 0.16 | 942.05 |
| 2848 | PEGDNFYC | -1.07 | Non-Toxin | -0.15 | 0.16 | 944.08 |
| 2849 | PEGDNFYN | -1.29 | Non-Toxin | -0.24 | 0.16 | 955.05 |
| 2850 | PEGDNFYQ | -1.12 | Non-Toxin | -0.24 | 0.32 | 969.08 |
| 2851 | PEGDNWFS | -1.03 | Non-Toxin | -0.15 | 0.16 | 951.06 |
| 2852 | PEGDNWFT | -0.96 | Non-Toxin | -0.14 | 0.16 | 965.09 |
| 2853 | PEGDNWFC | -0.65 | Non-Toxin | -0.11 | 0.16 | 967.12 |
| 2854 | PEGDNWFN | -0.95 | Non-Toxin | -0.19 | 0.16 | 978.09 |
| 2855 | PEGDNWFQ | -1.01 | Non-Toxin | -0.2 | 0.32 | 992.12 |
| 2856 | PEGDNWWS | -0.96 | Non-Toxin | -0.18 | 0.16 | 990.1 |
| 2857 | PEGDNWWT | -0.94 | Non-Toxin | -0.17 | 0.16 | 1004.13 |
| 2858 | PEGDNWWC | -0.43 | Non-Toxin | -0.14 | 0.16 | 1006.16 |
| 2859 | PEGDNWWN | -1.02 | Non-Toxin | -0.22 | 0.16 | 1017.13 |
| 2860 | PEGDNWWQ | -0.93 | Non-Toxin | -0.23 | 0.32 | 1031.16 |
| 2861 | PEGDNWYS | -0.89 | Non-Toxin | -0.22 | 0.16 | 967.06 |
| 2862 | PEGDNWYT | -0.83 | Non-Toxin | -0.21 | 0.16 | 981.09 |
| 2863 | PEGDNWYC | -0.55 | Non-Toxin | -0.18 | 0.16 | 983.12 |
| 2864 | PEGDNWYN | -0.81 | Non-Toxin | -0.27 | 0.16 | 994.09 |
| 2865 | PEGDNWYQ | -0.61 | Non-Toxin | -0.27 | 0.32 | 1008.12 |
| 2866 | PEGDNYFS | -0.9 | Non-Toxin | -0.19 | 0.16 | 928.02 |
| 2867 | PEGDNYFT | -0.82 | Non-Toxin | -0.18 | 0.16 | 942.05 |
| 2868 | PEGDNYFC | -0.53 | Non-Toxin | -0.15 | 0.16 | 944.08 |
| 2869 | PEGDNYFN | -0.85 | Non-Toxin | -0.24 | 0.16 | 955.05 |
| 2870 | PEGDNYFQ | -0.9 | Non-Toxin | -0.24 | 0.32 | 969.08 |
| 2871 | PEGDNYWS | -0.96 | Non-Toxin | -0.22 | 0.16 | 967.06 |
| 2872 | PEGDNYWT | -0.94 | Non-Toxin | -0.21 | 0.16 | 981.09 |
| 2873 | PEGDNYWC | -0.43 | Non-Toxin | -0.18 | 0.16 | 983.12 |
| 2874 | PEGDNYWN | -1.04 | Non-Toxin | -0.27 | 0.16 | 994.09 |
| 2875 | PEGDNYWQ | -0.93 | Non-Toxin | -0.27 | 0.32 | 1008.12 |
| 2876 | PEGDNYYS | -0.91 | Non-Toxin | -0.26 | 0.16 | 944.02 |
| 2877 | PEGDNYYT | -0.8 | Non-Toxin | -0.25 | 0.16 | 958.05 |
| 2878 | PEGDNYYC | -0.44 | Non-Toxin | -0.23 | 0.16 | 960.08 |
| 2879 | PEGDNYYN | -0.84 | Non-Toxin | -0.31 | 0.16 | 971.05 |
| 2880 | PEGDNYYQ | -0.62 | Non-Toxin | -0.32 | 0.32 | 985.08 |
| 2881 | PEGDQFFS | -0.8 | Non-Toxin | -0.12 | 0.32 | 926.05 |
| 2882 | PEGDQFFT | -0.7 | Non-Toxin | -0.11 | 0.32 | 940.08 |
| 2883 | PEGDQFFC | -0.38 | Non-Toxin | -0.09 | 0.32 | 942.11 |
| 2884 | PEGDQFFN | -0.67 | Non-Toxin | -0.17 | 0.32 | 953.08 |
| 2885 | PEGDQFFQ | -0.77 | Non-Toxin | -0.18 | 0.47 | 967.11 |
| 2886 | PEGDQFWS | -0.86 | Non-Toxin | -0.15 | 0.32 | 965.09 |
| 2887 | PEGDQFWT | -0.82 | Non-Toxin | -0.14 | 0.32 | 979.12 |
| 2888 | PEGDQFWC | -0.32 | Non-Toxin | -0.12 | 0.32 | 981.15 |
| 2889 | PEGDQFWN | -0.92 | Non-Toxin | -0.2 | 0.32 | 992.12 |
| 2890 | PEGDQFWQ | -0.83 | Non-Toxin | -0.21 | 0.47 | 1006.15 |
| 2891 | PEGDQFYS | -0.96 | Non-Toxin | -0.2 | 0.32 | 942.05 |
| 2892 | PEGDQFYT | -0.9 | Non-Toxin | -0.19 | 0.32 | 956.08 |
| 2893 | PEGDQFYC | -0.67 | Non-Toxin | -0.16 | 0.32 | 958.11 |
| 2894 | PEGDQFYN | -0.93 | Non-Toxin | -0.24 | 0.32 | 969.08 |
| 2895 | PEGDQFYQ | -0.7 | Non-Toxin | -0.25 | 0.47 | 983.11 |
| 2896 | PEGDQWFS | -0.89 | Non-Toxin | -0.15 | 0.32 | 965.09 |
| 2897 | PEGDQWFT | -0.81 | Non-Toxin | -0.14 | 0.32 | 979.12 |
| 2898 | PEGDQWFC | -0.5 | Non-Toxin | -0.11 | 0.32 | 981.15 |
| 2899 | PEGDQWFN | -0.78 | Non-Toxin | -0.2 | 0.32 | 992.12 |
| 2900 | PEGDQWFQ | -0.84 | Non-Toxin | -0.21 | 0.47 | 1006.15 |
| 2901 | PEGDQWWS | -0.81 | Non-Toxin | -0.18 | 0.32 | 1004.13 |
| 2902 | PEGDQWWT | -0.78 | Non-Toxin | -0.17 | 0.32 | 1018.16 |
| 2903 | PEGDQWWC | -0.27 | Non-Toxin | -0.14 | 0.32 | 1020.19 |
| 2904 | PEGDQWWN | -0.87 | Non-Toxin | -0.23 | 0.32 | 1031.16 |
| 2905 | PEGDQWWQ | -0.77 | Non-Toxin | -0.24 | 0.47 | 1045.19 |
| 2906 | PEGDQWYS | -0.69 | Non-Toxin | -0.23 | 0.32 | 981.09 |
| 2907 | PEGDQWYT | -0.64 | Non-Toxin | -0.22 | 0.32 | 995.12 |
| 2908 | PEGDQWYC | -0.36 | Non-Toxin | -0.19 | 0.32 | 997.15 |
| 2909 | PEGDQWYN | -0.65 | Non-Toxin | -0.27 | 0.32 | 1008.12 |
| 2910 | PEGDQWYQ | -0.44 | Non-Toxin | -0.28 | 0.47 | 1022.15 |
| 2911 | PEGDQYFS | -0.95 | Non-Toxin | -0.2 | 0.32 | 942.05 |
| 2912 | PEGDQYFT | -0.84 | Non-Toxin | -0.19 | 0.32 | 956.08 |
| 2913 | PEGDQYFC | -0.56 | Non-Toxin | -0.16 | 0.32 | 958.11 |
| 2914 | PEGDQYFN | -0.86 | Non-Toxin | -0.24 | 0.32 | 969.08 |
| 2915 | PEGDQYFQ | -0.93 | Non-Toxin | -0.25 | 0.47 | 983.11 |
| 2916 | PEGDQYWS | -0.98 | Non-Toxin | -0.23 | 0.32 | 981.09 |
| 2917 | PEGDQYWT | -0.94 | Non-Toxin | -0.22 | 0.32 | 995.12 |
| 2918 | PEGDQYWC | -0.43 | Non-Toxin | -0.19 | 0.32 | 997.15 |
| 2919 | PEGDQYWN | -1.04 | Non-Toxin | -0.27 | 0.32 | 1008.12 |
| 2920 | PEGDQYWQ | -0.93 | Non-Toxin | -0.28 | 0.47 | 1022.15 |
| 2921 | PEGDQYYS | -0.89 | Non-Toxin | -0.27 | 0.32 | 958.05 |
| 2922 | PEGDQYYT | -0.82 | Non-Toxin | -0.26 | 0.32 | 972.08 |
| 2923 | PEGDQYYC | -0.5 | Non-Toxin | -0.23 | 0.32 | 974.11 |
| 2924 | PEGDQYYN | -0.84 | Non-Toxin | -0.32 | 0.32 | 985.08 |
| 2925 | PEGDQYYQ | -0.64 | Non-Toxin | -0.32 | 0.47 | 999.11 |
| 2926 | PEGESFFS | -1 | Non-Toxin | -0.06 | 0.32 | 899.02 |
| 2927 | PEGESFFT | -0.97 | Non-Toxin | -0.05 | 0.32 | 913.05 |
| 2928 | PEGESFFC | -0.67 | Non-Toxin | -0.02 | 0.32 | 915.08 |
| 2929 | PEGESFFN | -0.97 | Non-Toxin | -0.1 | 0.32 | 926.05 |
| 2930 | PEGESFFQ | -0.97 | Non-Toxin | -0.11 | 0.47 | 940.08 |
| 2931 | PEGESFWS | -1.17 | Non-Toxin | -0.09 | 0.32 | 938.06 |
| 2932 | PEGESFWT | -1.08 | Non-Toxin | -0.08 | 0.32 | 952.09 |
| 2933 | PEGESFWC | -0.56 | Non-Toxin | -0.05 | 0.32 | 954.12 |
| 2934 | PEGESFWN | -1.2 | Non-Toxin | -0.13 | 0.32 | 965.09 |
| 2935 | PEGESFWQ | -1.1 | Non-Toxin | -0.14 | 0.47 | 979.12 |
| 2936 | PEGESFYS | -1.33 | Non-Toxin | -0.13 | 0.32 | 915.02 |
| 2937 | PEGESFYT | -1.31 | Non-Toxin | -0.12 | 0.32 | 929.05 |
| 2938 | PEGESFYC | -1.1 | Non-Toxin | -0.09 | 0.32 | 931.08 |
| 2939 | PEGESFYN | -1.28 | Non-Toxin | -0.18 | 0.32 | 942.05 |
| 2940 | PEGESFYQ | -1.02 | Non-Toxin | -0.18 | 0.47 | 956.08 |
| 2941 | PEGESWFS | -1.29 | Non-Toxin | -0.09 | 0.32 | 938.06 |
| 2942 | PEGESWFT | -1.25 | Non-Toxin | -0.08 | 0.32 | 952.09 |
| 2943 | PEGESWFC | -0.94 | Non-Toxin | -0.05 | 0.32 | 954.12 |
| 2944 | PEGESWFN | -1.22 | Non-Toxin | -0.13 | 0.32 | 965.09 |
| 2945 | PEGESWFQ | -1.26 | Non-Toxin | -0.14 | 0.47 | 979.12 |
| 2946 | PEGESWWS | -1.24 | Non-Toxin | -0.12 | 0.32 | 977.1 |
| 2947 | PEGESWWT | -1.2 | Non-Toxin | -0.11 | 0.32 | 991.13 |
| 2948 | PEGESWWC | -0.69 | Non-Toxin | -0.08 | 0.32 | 993.16 |
| 2949 | PEGESWWN | -1.3 | Non-Toxin | -0.16 | 0.32 | 1004.13 |
| 2950 | PEGESWWQ | -1.19 | Non-Toxin | -0.17 | 0.47 | 1018.16 |
| 2951 | PEGESWYS | -1.12 | Non-Toxin | -0.16 | 0.32 | 954.06 |
| 2952 | PEGESWYT | -1.12 | Non-Toxin | -0.15 | 0.32 | 968.09 |
| 2953 | PEGESWYC | -0.84 | Non-Toxin | -0.12 | 0.32 | 970.12 |
| 2954 | PEGESWYN | -1.08 | Non-Toxin | -0.21 | 0.32 | 981.09 |
| 2955 | PEGESWYQ | -0.81 | Non-Toxin | -0.21 | 0.47 | 995.12 |
| 2956 | PEGESYFS | -1.17 | Non-Toxin | -0.13 | 0.32 | 915.02 |
| 2957 | PEGESYFT | -1.12 | Non-Toxin | -0.12 | 0.32 | 929.05 |
| 2958 | PEGESYFC | -0.83 | Non-Toxin | -0.09 | 0.32 | 931.08 |
| 2959 | PEGESYFN | -1.14 | Non-Toxin | -0.18 | 0.32 | 942.05 |
| 2960 | PEGESYFQ | -1.17 | Non-Toxin | -0.18 | 0.47 | 956.08 |
| 2961 | PEGESYWS | -1.22 | Non-Toxin | -0.16 | 0.32 | 954.06 |
| 2962 | PEGESYWT | -1.19 | Non-Toxin | -0.15 | 0.32 | 968.09 |
| 2963 | PEGESYWC | -0.67 | Non-Toxin | -0.12 | 0.32 | 970.12 |
| 2964 | PEGESYWN | -1.29 | Non-Toxin | -0.21 | 0.32 | 981.09 |
| 2965 | PEGESYWQ | -1.17 | Non-Toxin | -0.21 | 0.47 | 995.12 |
| 2966 | PEGESYYS | -1.1 | Non-Toxin | -0.2 | 0.32 | 931.02 |
| 2967 | PEGESYYT | -1.1 | Non-Toxin | -0.19 | 0.32 | 945.05 |
| 2968 | PEGESYYC | -0.75 | Non-Toxin | -0.17 | 0.32 | 947.08 |
| 2969 | PEGESYYN | -1.06 | Non-Toxin | -0.25 | 0.32 | 958.05 |
| 2970 | PEGESYYQ | -0.77 | Non-Toxin | -0.26 | 0.47 | 972.08 |
| 2971 | PEGETFFS | -1.28 | Non-Toxin | -0.05 | 0.32 | 913.05 |
| 2972 | PEGETFFT | -1.27 | Non-Toxin | -0.04 | 0.32 | 927.08 |
| 2973 | PEGETFFC | -0.94 | Non-Toxin | -0.01 | 0.32 | 929.11 |
| 2974 | PEGETFFN | -1.17 | Non-Toxin | -0.09 | 0.32 | 940.08 |
| 2975 | PEGETFFQ | -1.28 | Non-Toxin | -0.1 | 0.47 | 954.11 |
| 2976 | PEGETFWS | -1.37 | Non-Toxin | -0.08 | 0.32 | 952.09 |
| 2977 | PEGETFWT | -1.32 | Non-Toxin | -0.07 | 0.32 | 966.12 |
| 2978 | PEGETFWC | -0.82 | Non-Toxin | -0.04 | 0.32 | 968.15 |
| 2979 | PEGETFWN | -1.44 | Non-Toxin | -0.12 | 0.32 | 979.12 |
| 2980 | PEGETFWQ | -1.33 | Non-Toxin | -0.13 | 0.47 | 993.15 |
| 2981 | PEGETFYS | -1.54 | Non-Toxin | -0.12 | 0.32 | 929.05 |
| 2982 | PEGETFYT | -1.51 | Non-Toxin | -0.11 | 0.32 | 943.08 |
| 2983 | PEGETFYC | -1.31 | Non-Toxin | -0.08 | 0.32 | 945.11 |
| 2984 | PEGETFYN | -1.5 | Non-Toxin | -0.17 | 0.32 | 956.08 |
| 2985 | PEGETFYQ | -1.25 | Non-Toxin | -0.17 | 0.47 | 970.11 |
| 2986 | PEGETWFS | -1.26 | Non-Toxin | -0.08 | 0.32 | 952.09 |
| 2987 | PEGETWFT | -1.24 | Non-Toxin | -0.07 | 0.32 | 966.12 |
| 2988 | PEGETWFC | -0.93 | Non-Toxin | -0.04 | 0.32 | 968.15 |
| 2989 | PEGETWFN | -1.18 | Non-Toxin | -0.12 | 0.32 | 979.12 |
| 2990 | PEGETWFQ | -1.25 | Non-Toxin | -0.13 | 0.47 | 993.15 |
| 2991 | PEGETWWS | -1.21 | Non-Toxin | -0.11 | 0.32 | 991.13 |
| 2992 | PEGETWWT | -1.19 | Non-Toxin | -0.1 | 0.32 | 1005.16 |
| 2993 | PEGETWWC | -0.64 | Non-Toxin | -0.07 | 0.32 | 1007.19 |
| 2994 | PEGETWWN | -1.28 | Non-Toxin | -0.15 | 0.32 | 1018.16 |
| 2995 | PEGETWWQ | -1.18 | Non-Toxin | -0.16 | 0.47 | 1032.19 |
| 2996 | PEGETWYS | -1.14 | Non-Toxin | -0.15 | 0.32 | 968.09 |
| 2997 | PEGETWYT | -1.1 | Non-Toxin | -0.14 | 0.32 | 982.12 |
| 2998 | PEGETWYC | -0.84 | Non-Toxin | -0.11 | 0.32 | 984.15 |
| 2999 | PEGETWYN | -1.08 | Non-Toxin | -0.2 | 0.32 | 995.12 |
| 3000 | PEGETWYQ | -0.82 | Non-Toxin | -0.2 | 0.47 | 1009.15 |
| 3001 | PEGETYFS | -1.14 | Non-Toxin | -0.12 | 0.32 | 929.05 |
| 3002 | PEGETYFT | -1.11 | Non-Toxin | -0.11 | 0.32 | 943.08 |
| 3003 | PEGETYFC | -0.84 | Non-Toxin | -0.08 | 0.32 | 945.11 |
| 3004 | PEGETYFN | -1.12 | Non-Toxin | -0.17 | 0.32 | 956.08 |
| 3005 | PEGETYFQ | -1.17 | Non-Toxin | -0.17 | 0.47 | 970.11 |
| 3006 | PEGETYWS | -1.2 | Non-Toxin | -0.15 | 0.32 | 968.09 |
| 3007 | PEGETYWT | -1.18 | Non-Toxin | -0.14 | 0.32 | 982.12 |
| 3008 | PEGETYWC | -0.65 | Non-Toxin | -0.11 | 0.32 | 984.15 |
| 3009 | PEGETYWN | -1.28 | Non-Toxin | -0.2 | 0.32 | 995.12 |
| 3010 | PEGETYWQ | -1.17 | Non-Toxin | -0.2 | 0.47 | 1009.15 |
| 3011 | PEGETYYS | -1.14 | Non-Toxin | -0.19 | 0.32 | 945.05 |
| 3012 | PEGETYYT | -1.08 | Non-Toxin | -0.18 | 0.32 | 959.08 |
| 3013 | PEGETYYC | -0.8 | Non-Toxin | -0.16 | 0.32 | 961.11 |
| 3014 | PEGETYYN | -1.09 | Non-Toxin | -0.24 | 0.32 | 972.08 |
| 3015 | PEGETYYQ | -0.82 | Non-Toxin | -0.25 | 0.47 | 986.11 |
| 3016 | PEGECFFS | -0.66 | Non-Toxin | -0.02 | 0.32 | 915.08 |
| 3017 | PEGECFFT | -0.6 | Non-Toxin | -0.01 | 0.32 | 929.11 |
| 3018 | PEGECFFC | -0.25 | Non-Toxin | 0.02 | 0.32 | 931.14 |
| 3019 | PEGECFFN | -0.57 | Non-Toxin | -0.07 | 0.32 | 942.11 |
| 3020 | PEGECFFQ | -0.66 | Non-Toxin | -0.07 | 0.47 | 956.14 |
| 3021 | PEGECFWS | -0.9 | Non-Toxin | -0.05 | 0.32 | 954.12 |
| 3022 | PEGECFWT | -0.88 | Non-Toxin | -0.04 | 0.32 | 968.15 |
| 3023 | PEGECFWC | -0.35 | Non-Toxin | -0.01 | 0.32 | 970.18 |
| 3024 | PEGECFWN | -0.97 | Non-Toxin | -0.1 | 0.32 | 981.15 |
| 3025 | PEGECFWQ | -0.86 | Non-Toxin | -0.1 | 0.47 | 995.18 |
| 3026 | PEGECFYS | -0.96 | Non-Toxin | -0.09 | 0.32 | 931.08 |
| 3027 | PEGECFYT | -0.93 | Non-Toxin | -0.08 | 0.32 | 945.11 |
| 3028 | PEGECFYC | -0.82 | Non-Toxin | -0.05 | 0.32 | 947.14 |
| 3029 | PEGECFYN | -0.92 | Non-Toxin | -0.14 | 0.32 | 958.11 |
| 3030 | PEGECFYQ | -0.67 | Non-Toxin | -0.15 | 0.47 | 972.14 |
| 3031 | PEGECWFS | -0.61 | Non-Toxin | -0.05 | 0.32 | 954.12 |
| 3032 | PEGECWFT | -0.53 | Non-Toxin | -0.04 | 0.32 | 968.15 |
| 3033 | PEGECWFC | -0.23 | Non-Toxin | -0.01 | 0.32 | 970.18 |
| 3034 | PEGECWFN | -0.49 | Non-Toxin | -0.1 | 0.32 | 981.15 |
| 3035 | PEGECWFQ | -0.58 | Non-Toxin | -0.1 | 0.47 | 995.18 |
| 3036 | PEGECWWS | -0.51 | Non-Toxin | -0.08 | 0.32 | 993.16 |
| 3037 | PEGECWWT | -0.51 | Non-Toxin | -0.07 | 0.32 | 1007.19 |
| 3038 | PEGECWWC | 0.03 | Toxin | -0.04 | 0.32 | 1009.22 |
| 3039 | PEGECWWN | -0.59 | Non-Toxin | -0.13 | 0.32 | 1020.19 |
| 3040 | PEGECWWQ | -0.49 | Non-Toxin | -0.13 | 0.47 | 1034.22 |
| 3041 | PEGECWYS | -0.44 | Non-Toxin | -0.12 | 0.32 | 970.12 |
| 3042 | PEGECWYT | -0.38 | Non-Toxin | -0.11 | 0.32 | 984.15 |
| 3043 | PEGECWYC | -0.16 | Non-Toxin | -0.08 | 0.32 | 986.18 |
| 3044 | PEGECWYN | -0.38 | Non-Toxin | -0.17 | 0.32 | 997.15 |
| 3045 | PEGECWYQ | -0.11 | Non-Toxin | -0.18 | 0.47 | 1011.18 |
| 3046 | PEGECYFS | -0.56 | Non-Toxin | -0.09 | 0.32 | 931.08 |
| 3047 | PEGECYFT | -0.47 | Non-Toxin | -0.08 | 0.32 | 945.11 |
| 3048 | PEGECYFC | -0.2 | Non-Toxin | -0.05 | 0.32 | 947.14 |
| 3049 | PEGECYFN | -0.46 | Non-Toxin | -0.14 | 0.32 | 958.11 |
| 3050 | PEGECYFQ | -0.55 | Non-Toxin | -0.15 | 0.47 | 972.14 |
| 3051 | PEGECYWS | -0.49 | Non-Toxin | -0.12 | 0.32 | 970.12 |
| 3052 | PEGECYWT | -0.49 | Non-Toxin | -0.11 | 0.32 | 984.15 |
| 3053 | PEGECYWC | 0.01 | Toxin | -0.08 | 0.32 | 986.18 |
| 3054 | PEGECYWN | -0.6 | Non-Toxin | -0.17 | 0.32 | 997.15 |
| 3055 | PEGECYWQ | -0.47 | Non-Toxin | -0.18 | 0.47 | 1011.18 |
| 3056 | PEGECYYS | -0.42 | Non-Toxin | -0.17 | 0.32 | 947.08 |
| 3057 | PEGECYYT | -0.42 | Non-Toxin | -0.16 | 0.32 | 961.11 |
| 3058 | PEGECYYC | -0.14 | Non-Toxin | -0.13 | 0.32 | 963.14 |
| 3059 | PEGECYYN | -0.35 | Non-Toxin | -0.21 | 0.32 | 974.11 |
| 3060 | PEGECYYQ | -0.12 | Non-Toxin | -0.22 | 0.47 | 988.14 |
| 3061 | PEGENFFS | -1.26 | Non-Toxin | -0.1 | 0.32 | 926.05 |
| 3062 | PEGENFFT | -1.18 | Non-Toxin | -0.09 | 0.32 | 940.08 |
| 3063 | PEGENFFC | -0.88 | Non-Toxin | -0.07 | 0.32 | 942.11 |
| 3064 | PEGENFFN | -1.14 | Non-Toxin | -0.15 | 0.32 | 953.08 |
| 3065 | PEGENFFQ | -1.22 | Non-Toxin | -0.16 | 0.47 | 967.11 |
| 3066 | PEGENFWS | -1.35 | Non-Toxin | -0.13 | 0.32 | 965.09 |
| 3067 | PEGENFWT | -1.31 | Non-Toxin | -0.12 | 0.32 | 979.12 |
| 3068 | PEGENFWC | -0.82 | Non-Toxin | -0.1 | 0.32 | 981.15 |
| 3069 | PEGENFWN | -1.41 | Non-Toxin | -0.18 | 0.32 | 992.12 |
| 3070 | PEGENFWQ | -1.32 | Non-Toxin | -0.19 | 0.47 | 1006.15 |
| 3071 | PEGENFYS | -1.47 | Non-Toxin | -0.18 | 0.32 | 942.05 |
| 3072 | PEGENFYT | -1.43 | Non-Toxin | -0.17 | 0.32 | 956.08 |
| 3073 | PEGENFYC | -1.24 | Non-Toxin | -0.14 | 0.32 | 958.11 |
| 3074 | PEGENFYN | -1.44 | Non-Toxin | -0.23 | 0.32 | 969.08 |
| 3075 | PEGENFYQ | -1.2 | Non-Toxin | -0.23 | 0.47 | 983.11 |
| 3076 | PEGENWFS | -1.15 | Non-Toxin | -0.13 | 0.32 | 965.09 |
| 3077 | PEGENWFT | -1.09 | Non-Toxin | -0.12 | 0.32 | 979.12 |
| 3078 | PEGENWFC | -0.79 | Non-Toxin | -0.1 | 0.32 | 981.15 |
| 3079 | PEGENWFN | -1.05 | Non-Toxin | -0.18 | 0.32 | 992.12 |
| 3080 | PEGENWFQ | -1.11 | Non-Toxin | -0.19 | 0.47 | 1006.15 |
| 3081 | PEGENWWS | -1.07 | Non-Toxin | -0.16 | 0.32 | 1004.13 |
| 3082 | PEGENWWT | -1.05 | Non-Toxin | -0.15 | 0.32 | 1018.16 |
| 3083 | PEGENWWC | -0.54 | Non-Toxin | -0.13 | 0.32 | 1020.19 |
| 3084 | PEGENWWN | -1.15 | Non-Toxin | -0.21 | 0.32 | 1031.16 |
| 3085 | PEGENWWQ | -1.05 | Non-Toxin | -0.22 | 0.47 | 1045.19 |
| 3086 | PEGENWYS | -0.97 | Non-Toxin | -0.21 | 0.32 | 981.09 |
| 3087 | PEGENWYT | -0.96 | Non-Toxin | -0.2 | 0.32 | 995.12 |
| 3088 | PEGENWYC | -0.7 | Non-Toxin | -0.17 | 0.32 | 997.15 |
| 3089 | PEGENWYN | -0.94 | Non-Toxin | -0.26 | 0.32 | 1008.12 |
| 3090 | PEGENWYQ | -0.68 | Non-Toxin | -0.26 | 0.47 | 1022.15 |
| 3091 | PEGENYFS | -1.03 | Non-Toxin | -0.18 | 0.32 | 942.05 |
| 3092 | PEGENYFT | -0.98 | Non-Toxin | -0.17 | 0.32 | 956.08 |
| 3093 | PEGENYFC | -0.7 | Non-Toxin | -0.14 | 0.32 | 958.11 |
| 3094 | PEGENYFN | -0.98 | Non-Toxin | -0.23 | 0.32 | 969.08 |
| 3095 | PEGENYFQ | -1.02 | Non-Toxin | -0.23 | 0.47 | 983.11 |
| 3096 | PEGENYWS | -1.08 | Non-Toxin | -0.21 | 0.32 | 981.09 |
| 3097 | PEGENYWT | -1.06 | Non-Toxin | -0.2 | 0.32 | 995.12 |
| 3098 | PEGENYWC | -0.54 | Non-Toxin | -0.17 | 0.32 | 997.15 |
| 3099 | PEGENYWN | -1.17 | Non-Toxin | -0.26 | 0.32 | 1008.12 |
| 3100 | PEGENYWQ | -1.05 | Non-Toxin | -0.26 | 0.47 | 1022.15 |
| 3101 | PEGENYYS | -0.97 | Non-Toxin | -0.25 | 0.32 | 958.05 |
| 3102 | PEGENYYT | -0.92 | Non-Toxin | -0.24 | 0.32 | 972.08 |
| 3103 | PEGENYYC | -0.57 | Non-Toxin | -0.21 | 0.32 | 974.11 |
| 3104 | PEGENYYN | -0.95 | Non-Toxin | -0.3 | 0.32 | 985.08 |
| 3105 | PEGENYYQ | -0.68 | Non-Toxin | -0.3 | 0.47 | 999.11 |
| 3106 | PEGEQFFS | -1.04 | Non-Toxin | -0.11 | 0.47 | 940.08 |
| 3107 | PEGEQFFT | -0.95 | Non-Toxin | -0.1 | 0.47 | 954.11 |
| 3108 | PEGEQFFC | -0.65 | Non-Toxin | -0.07 | 0.47 | 956.14 |
| 3109 | PEGEQFFN | -0.92 | Non-Toxin | -0.16 | 0.47 | 967.11 |
| 3110 | PEGEQFFQ | -1.02 | Non-Toxin | -0.16 | 0.63 | 981.14 |
| 3111 | PEGEQFWS | -1.15 | Non-Toxin | -0.14 | 0.47 | 979.12 |
| 3112 | PEGEQFWT | -1.09 | Non-Toxin | -0.13 | 0.47 | 993.15 |
| 3113 | PEGEQFWC | -0.6 | Non-Toxin | -0.1 | 0.47 | 995.18 |
| 3114 | PEGEQFWN | -1.2 | Non-Toxin | -0.19 | 0.47 | 1006.15 |
| 3115 | PEGEQFWQ | -1.11 | Non-Toxin | -0.19 | 0.63 | 1020.18 |
| 3116 | PEGEQFYS | -1.29 | Non-Toxin | -0.18 | 0.47 | 956.08 |
| 3117 | PEGEQFYT | -1.24 | Non-Toxin | -0.17 | 0.47 | 970.11 |
| 3118 | PEGEQFYC | -1.04 | Non-Toxin | -0.15 | 0.47 | 972.14 |
| 3119 | PEGEQFYN | -1.24 | Non-Toxin | -0.23 | 0.47 | 983.11 |
| 3120 | PEGEQFYQ | -0.98 | Non-Toxin | -0.24 | 0.63 | 997.14 |
| 3121 | PEGEQWFS | -1.14 | Non-Toxin | -0.14 | 0.47 | 979.12 |
| 3122 | PEGEQWFT | -1.07 | Non-Toxin | -0.13 | 0.47 | 993.15 |
| 3123 | PEGEQWFC | -0.78 | Non-Toxin | -0.1 | 0.47 | 995.18 |
| 3124 | PEGEQWFN | -1.04 | Non-Toxin | -0.19 | 0.47 | 1006.15 |
| 3125 | PEGEQWFQ | -1.1 | Non-Toxin | -0.19 | 0.63 | 1020.18 |
| 3126 | PEGEQWWS | -1.09 | Non-Toxin | -0.17 | 0.47 | 1018.16 |
| 3127 | PEGEQWWT | -1.05 | Non-Toxin | -0.16 | 0.47 | 1032.19 |
| 3128 | PEGEQWWC | -0.54 | Non-Toxin | -0.13 | 0.47 | 1034.22 |
| 3129 | PEGEQWWN | -1.15 | Non-Toxin | -0.22 | 0.47 | 1045.19 |
| 3130 | PEGEQWWQ | -1.05 | Non-Toxin | -0.22 | 0.63 | 1059.22 |
| 3131 | PEGEQWYS | -0.98 | Non-Toxin | -0.21 | 0.47 | 995.12 |
| 3132 | PEGEQWYT | -0.95 | Non-Toxin | -0.2 | 0.47 | 1009.15 |
| 3133 | PEGEQWYC | -0.69 | Non-Toxin | -0.18 | 0.47 | 1011.18 |
| 3134 | PEGEQWYN | -0.92 | Non-Toxin | -0.26 | 0.47 | 1022.15 |
| 3135 | PEGEQWYQ | -0.69 | Non-Toxin | -0.27 | 0.63 | 1036.18 |
| 3136 | PEGEQYFS | -1.24 | Non-Toxin | -0.18 | 0.47 | 956.08 |
| 3137 | PEGEQYFT | -1.15 | Non-Toxin | -0.17 | 0.47 | 970.11 |
| 3138 | PEGEQYFC | -0.89 | Non-Toxin | -0.15 | 0.47 | 972.14 |
| 3139 | PEGEQYFN | -1.16 | Non-Toxin | -0.23 | 0.47 | 983.11 |
| 3140 | PEGEQYFQ | -1.23 | Non-Toxin | -0.24 | 0.63 | 997.14 |
| 3141 | PEGEQYWS | -1.26 | Non-Toxin | -0.21 | 0.47 | 995.12 |
| 3142 | PEGEQYWT | -1.22 | Non-Toxin | -0.2 | 0.47 | 1009.15 |
| 3143 | PEGEQYWC | -0.71 | Non-Toxin | -0.18 | 0.47 | 1011.18 |
| 3144 | PEGEQYWN | -1.32 | Non-Toxin | -0.26 | 0.47 | 1022.15 |
| 3145 | PEGEQYWQ | -1.21 | Non-Toxin | -0.27 | 0.63 | 1036.18 |
| 3146 | PEGEQYYS | -1.19 | Non-Toxin | -0.26 | 0.47 | 972.08 |
| 3147 | PEGEQYYT | -1.13 | Non-Toxin | -0.25 | 0.47 | 986.11 |
| 3148 | PEGEQYYC | -0.84 | Non-Toxin | -0.22 | 0.47 | 988.14 |
| 3149 | PEGEQYYN | -1.12 | Non-Toxin | -0.3 | 0.47 | 999.11 |
| 3150 | PEGEQYYQ | -0.89 | Non-Toxin | -0.31 | 0.63 | 1013.14 |
| 3151 | PEPDSFFS | -0.96 | Non-Toxin | -0.1 | 0.16 | 925.05 |
| 3152 | PEPDSFFT | -0.9 | Non-Toxin | -0.09 | 0.16 | 939.08 |
| 3153 | PEPDSFFC | -0.56 | Non-Toxin | -0.06 | 0.16 | 941.11 |
| 3154 | PEPDSFFN | -0.93 | Non-Toxin | -0.14 | 0.16 | 952.08 |
| 3155 | PEPDSFFQ | -0.9 | Non-Toxin | -0.15 | 0.32 | 966.11 |
| 3156 | PEPDSFWS | -1.12 | Non-Toxin | -0.13 | 0.16 | 964.09 |
| 3157 | PEPDSFWT | -1.02 | Non-Toxin | -0.12 | 0.16 | 978.12 |
| 3158 | PEPDSFWC | -0.47 | Non-Toxin | -0.09 | 0.16 | 980.15 |
| 3159 | PEPDSFWN | -1.13 | Non-Toxin | -0.17 | 0.16 | 991.12 |
| 3160 | PEPDSFWQ | -1.03 | Non-Toxin | -0.18 | 0.32 | 1005.15 |
| 3161 | PEPDSFYS | -1.24 | Non-Toxin | -0.17 | 0.16 | 941.05 |
| 3162 | PEPDSFYT | -1.21 | Non-Toxin | -0.16 | 0.16 | 955.08 |
| 3163 | PEPDSFYC | -1.11 | Non-Toxin | -0.13 | 0.16 | 957.11 |
| 3164 | PEPDSFYN | -1.18 | Non-Toxin | -0.22 | 0.16 | 968.08 |
| 3165 | PEPDSFYQ | -1 | Non-Toxin | -0.22 | 0.32 | 982.11 |
| 3166 | PEPDSWFS | -1.28 | Non-Toxin | -0.13 | 0.16 | 964.09 |
| 3167 | PEPDSWFT | -1.21 | Non-Toxin | -0.12 | 0.16 | 978.12 |
| 3168 | PEPDSWFC | -0.86 | Non-Toxin | -0.09 | 0.16 | 980.15 |
| 3169 | PEPDSWFN | -1.21 | Non-Toxin | -0.18 | 0.16 | 991.12 |
| 3170 | PEPDSWFQ | -1.22 | Non-Toxin | -0.18 | 0.32 | 1005.15 |
| 3171 | PEPDSWWS | -1.21 | Non-Toxin | -0.16 | 0.16 | 1003.13 |
| 3172 | PEPDSWWT | -1.16 | Non-Toxin | -0.15 | 0.16 | 1017.16 |
| 3173 | PEPDSWWC | -0.62 | Non-Toxin | -0.12 | 0.16 | 1019.19 |
| 3174 | PEPDSWWN | -1.26 | Non-Toxin | -0.21 | 0.16 | 1030.16 |
| 3175 | PEPDSWWQ | -1.15 | Non-Toxin | -0.21 | 0.32 | 1044.19 |
| 3176 | PEPDSWYS | -1.04 | Non-Toxin | -0.2 | 0.16 | 980.09 |
| 3177 | PEPDSWYT | -1.03 | Non-Toxin | -0.19 | 0.16 | 994.12 |
| 3178 | PEPDSWYC | -0.86 | Non-Toxin | -0.16 | 0.16 | 996.15 |
| 3179 | PEPDSWYN | -0.99 | Non-Toxin | -0.25 | 0.16 | 1007.12 |
| 3180 | PEPDSWYQ | -0.79 | Non-Toxin | -0.26 | 0.32 | 1021.15 |
| 3181 | PEPDSYFS | -1.12 | Non-Toxin | -0.17 | 0.16 | 941.05 |
| 3182 | PEPDSYFT | -1.03 | Non-Toxin | -0.16 | 0.16 | 955.08 |
| 3183 | PEPDSYFC | -0.71 | Non-Toxin | -0.13 | 0.16 | 957.11 |
| 3184 | PEPDSYFN | -1.08 | Non-Toxin | -0.22 | 0.16 | 968.08 |
| 3185 | PEPDSYFQ | -1.08 | Non-Toxin | -0.22 | 0.32 | 982.11 |
| 3186 | PEPDSYWS | -1.15 | Non-Toxin | -0.2 | 0.16 | 980.09 |
| 3187 | PEPDSYWT | -1.11 | Non-Toxin | -0.19 | 0.16 | 994.12 |
| 3188 | PEPDSYWC | -0.56 | Non-Toxin | -0.16 | 0.16 | 996.15 |
| 3189 | PEPDSYWN | -1.2 | Non-Toxin | -0.25 | 0.16 | 1007.12 |
| 3190 | PEPDSYWQ | -1.09 | Non-Toxin | -0.26 | 0.32 | 1021.15 |
| 3191 | PEPDSYYS | -0.98 | Non-Toxin | -0.24 | 0.16 | 957.05 |
| 3192 | PEPDSYYT | -0.98 | Non-Toxin | -0.23 | 0.16 | 971.08 |
| 3193 | PEPDSYYC | -0.74 | Non-Toxin | -0.21 | 0.16 | 973.11 |
| 3194 | PEPDSYYN | -0.94 | Non-Toxin | -0.29 | 0.16 | 984.08 |
| 3195 | PEPDSYYQ | -0.72 | Non-Toxin | -0.3 | 0.32 | 998.11 |
| 3196 | PEPDTFFS | -0.93 | Non-Toxin | -0.09 | 0.16 | 939.08 |
| 3197 | PEPDTFFT | -0.84 | Non-Toxin | -0.08 | 0.16 | 953.11 |
| 3198 | PEPDTFFC | -0.44 | Non-Toxin | -0.05 | 0.16 | 955.14 |
| 3199 | PEPDTFFN | -0.82 | Non-Toxin | -0.13 | 0.16 | 966.11 |
| 3200 | PEPDTFFQ | -0.88 | Non-Toxin | -0.14 | 0.32 | 980.14 |
| 3201 | PEPDTFWS | -1.02 | Non-Toxin | -0.12 | 0.16 | 978.12 |
| 3202 | PEPDTFWT | -0.95 | Non-Toxin | -0.11 | 0.16 | 992.15 |
| 3203 | PEPDTFWC | -0.44 | Non-Toxin | -0.08 | 0.16 | 994.18 |
| 3204 | PEPDTFWN | -1.08 | Non-Toxin | -0.16 | 0.16 | 1005.15 |
| 3205 | PEPDTFWQ | -0.96 | Non-Toxin | -0.17 | 0.32 | 1019.18 |
| 3206 | PEPDTFYS | -1.11 | Non-Toxin | -0.16 | 0.16 | 955.08 |
| 3207 | PEPDTFYT | -1.12 | Non-Toxin | -0.15 | 0.16 | 969.11 |
| 3208 | PEPDTFYC | -0.94 | Non-Toxin | -0.12 | 0.16 | 971.14 |
| 3209 | PEPDTFYN | -1.1 | Non-Toxin | -0.21 | 0.16 | 982.11 |
| 3210 | PEPDTFYQ | -0.9 | Non-Toxin | -0.21 | 0.32 | 996.14 |
| 3211 | PEPDTWFS | -0.98 | Non-Toxin | -0.12 | 0.16 | 978.12 |
| 3212 | PEPDTWFT | -0.88 | Non-Toxin | -0.11 | 0.16 | 992.15 |
| 3213 | PEPDTWFC | -0.5 | Non-Toxin | -0.08 | 0.16 | 994.18 |
| 3214 | PEPDTWFN | -0.89 | Non-Toxin | -0.17 | 0.16 | 1005.15 |
| 3215 | PEPDTWFQ | -0.93 | Non-Toxin | -0.17 | 0.32 | 1019.18 |
| 3216 | PEPDTWWS | -0.92 | Non-Toxin | -0.15 | 0.16 | 1017.16 |
| 3217 | PEPDTWWT | -0.88 | Non-Toxin | -0.14 | 0.16 | 1031.19 |
| 3218 | PEPDTWWC | -0.33 | Non-Toxin | -0.11 | 0.16 | 1033.22 |
| 3219 | PEPDTWWN | -0.98 | Non-Toxin | -0.2 | 0.16 | 1044.19 |
| 3220 | PEPDTWWQ | -0.87 | Non-Toxin | -0.2 | 0.32 | 1058.22 |
| 3221 | PEPDTWYS | -0.78 | Non-Toxin | -0.19 | 0.16 | 994.12 |
| 3222 | PEPDTWYT | -0.78 | Non-Toxin | -0.18 | 0.16 | 1008.15 |
| 3223 | PEPDTWYC | -0.55 | Non-Toxin | -0.15 | 0.16 | 1010.18 |
| 3224 | PEPDTWYN | -0.75 | Non-Toxin | -0.24 | 0.16 | 1021.15 |
| 3225 | PEPDTWYQ | -0.54 | Non-Toxin | -0.24 | 0.32 | 1035.18 |
| 3226 | PEPDTYFS | -0.87 | Non-Toxin | -0.16 | 0.16 | 955.08 |
| 3227 | PEPDTYFT | -0.76 | Non-Toxin | -0.15 | 0.16 | 969.11 |
| 3228 | PEPDTYFC | -0.41 | Non-Toxin | -0.12 | 0.16 | 971.14 |
| 3229 | PEPDTYFN | -0.84 | Non-Toxin | -0.21 | 0.16 | 982.11 |
| 3230 | PEPDTYFQ | -0.84 | Non-Toxin | -0.21 | 0.32 | 996.14 |
| 3231 | PEPDTYWS | -0.9 | Non-Toxin | -0.19 | 0.16 | 994.12 |
| 3232 | PEPDTYWT | -0.87 | Non-Toxin | -0.18 | 0.16 | 1008.15 |
| 3233 | PEPDTYWC | -0.33 | Non-Toxin | -0.15 | 0.16 | 1010.18 |
| 3234 | PEPDTYWN | -0.97 | Non-Toxin | -0.24 | 0.16 | 1021.15 |
| 3235 | PEPDTYWQ | -0.85 | Non-Toxin | -0.24 | 0.32 | 1035.18 |
| 3236 | PEPDTYYS | -0.81 | Non-Toxin | -0.23 | 0.16 | 971.08 |
| 3237 | PEPDTYYT | -0.77 | Non-Toxin | -0.22 | 0.16 | 985.11 |
| 3238 | PEPDTYYC | -0.53 | Non-Toxin | -0.2 | 0.16 | 987.14 |
| 3239 | PEPDTYYN | -0.78 | Non-Toxin | -0.28 | 0.16 | 998.11 |
| 3240 | PEPDTYYQ | -0.56 | Non-Toxin | -0.29 | 0.32 | 1012.14 |
| 3241 | PEPDCFFS | -0.6 | Non-Toxin | -0.06 | 0.16 | 941.11 |
| 3242 | PEPDCFFT | -0.52 | Non-Toxin | -0.05 | 0.16 | 955.14 |
| 3243 | PEPDCFFC | -0.07 | Non-Toxin | -0.02 | 0.16 | 957.17 |
| 3244 | PEPDCFFN | -0.53 | Non-Toxin | -0.11 | 0.16 | 968.14 |
| 3245 | PEPDCFFQ | -0.58 | Non-Toxin | -0.11 | 0.32 | 982.17 |
| 3246 | PEPDCFWS | -0.83 | Non-Toxin | -0.09 | 0.16 | 980.15 |
| 3247 | PEPDCFWT | -0.8 | Non-Toxin | -0.08 | 0.16 | 994.18 |
| 3248 | PEPDCFWC | -0.27 | Non-Toxin | -0.05 | 0.16 | 996.21 |
| 3249 | PEPDCFWN | -0.9 | Non-Toxin | -0.14 | 0.16 | 1007.18 |
| 3250 | PEPDCFWQ | -0.79 | Non-Toxin | -0.14 | 0.32 | 1021.21 |
| 3251 | PEPDCFYS | -0.87 | Non-Toxin | -0.13 | 0.16 | 957.11 |
| 3252 | PEPDCFYT | -0.89 | Non-Toxin | -0.12 | 0.16 | 971.14 |
| 3253 | PEPDCFYC | -0.79 | Non-Toxin | -0.1 | 0.16 | 973.17 |
| 3254 | PEPDCFYN | -0.86 | Non-Toxin | -0.18 | 0.16 | 984.14 |
| 3255 | PEPDCFYQ | -0.66 | Non-Toxin | -0.19 | 0.32 | 998.17 |
| 3256 | PEPDCWFS | -0.59 | Non-Toxin | -0.09 | 0.16 | 980.15 |
| 3257 | PEPDCWFT | -0.5 | Non-Toxin | -0.08 | 0.16 | 994.18 |
| 3258 | PEPDCWFC | -0.09 | Non-Toxin | -0.05 | 0.16 | 996.21 |
| 3259 | PEPDCWFN | -0.49 | Non-Toxin | -0.14 | 0.16 | 1007.18 |
| 3260 | PEPDCWFQ | -0.54 | Non-Toxin | -0.14 | 0.32 | 1021.21 |
| 3261 | PEPDCWWS | -0.52 | Non-Toxin | -0.12 | 0.16 | 1019.19 |
| 3262 | PEPDCWWT | -0.51 | Non-Toxin | -0.11 | 0.16 | 1033.22 |
| 3263 | PEPDCWWC | 0.05 | Toxin | -0.08 | 0.16 | 1035.25 |
| 3264 | PEPDCWWN | -0.6 | Non-Toxin | -0.17 | 0.16 | 1046.22 |
| 3265 | PEPDCWWQ | -0.49 | Non-Toxin | -0.17 | 0.32 | 1060.25 |
| 3266 | PEPDCWYS | -0.4 | Non-Toxin | -0.16 | 0.16 | 996.15 |
| 3267 | PEPDCWYT | -0.39 | Non-Toxin | -0.15 | 0.16 | 1010.18 |
| 3268 | PEPDCWYC | -0.19 | Non-Toxin | -0.13 | 0.16 | 1012.21 |
| 3269 | PEPDCWYN | -0.38 | Non-Toxin | -0.21 | 0.16 | 1023.18 |
| 3270 | PEPDCWYQ | -0.16 | Non-Toxin | -0.22 | 0.32 | 1037.21 |
| 3271 | PEPDCYFS | -0.47 | Non-Toxin | -0.13 | 0.16 | 957.11 |
| 3272 | PEPDCYFT | -0.36 | Non-Toxin | -0.12 | 0.16 | 971.14 |
| 3273 | PEPDCYFC | 0.01 | Toxin | -0.1 | 0.16 | 973.17 |
| 3274 | PEPDCYFN | -0.39 | Non-Toxin | -0.18 | 0.16 | 984.14 |
| 3275 | PEPDCYFQ | -0.44 | Non-Toxin | -0.19 | 0.32 | 998.17 |
| 3276 | PEPDCYWS | -0.38 | Non-Toxin | -0.16 | 0.16 | 996.15 |
| 3277 | PEPDCYWT | -0.36 | Non-Toxin | -0.15 | 0.16 | 1010.18 |
| 3278 | PEPDCYWC | 0.15 | Toxin | -0.13 | 0.16 | 1012.21 |
| 3279 | PEPDCYWN | -0.48 | Non-Toxin | -0.21 | 0.16 | 1023.18 |
| 3280 | PEPDCYWQ | -0.35 | Non-Toxin | -0.22 | 0.32 | 1037.21 |
| 3281 | PEPDCYYS | -0.32 | Non-Toxin | -0.21 | 0.16 | 973.11 |
| 3282 | PEPDCYYT | -0.37 | Non-Toxin | -0.2 | 0.16 | 987.14 |
| 3283 | PEPDCYYC | -0.11 | Non-Toxin | -0.17 | 0.16 | 989.17 |
| 3284 | PEPDCYYN | -0.29 | Non-Toxin | -0.26 | 0.16 | 1000.14 |
| 3285 | PEPDCYYQ | -0.1 | Non-Toxin | -0.26 | 0.32 | 1014.17 |
| 3286 | PEPDNFFS | -1.12 | Non-Toxin | -0.15 | 0.16 | 952.08 |
| 3287 | PEPDNFFT | -1.03 | Non-Toxin | -0.14 | 0.16 | 966.11 |
| 3288 | PEPDNFFC | -0.65 | Non-Toxin | -0.11 | 0.16 | 968.14 |
| 3289 | PEPDNFFN | -1.04 | Non-Toxin | -0.19 | 0.16 | 979.11 |
| 3290 | PEPDNFFQ | -1.08 | Non-Toxin | -0.2 | 0.32 | 993.14 |
| 3291 | PEPDNFWS | -1.23 | Non-Toxin | -0.18 | 0.16 | 991.12 |
| 3292 | PEPDNFWT | -1.19 | Non-Toxin | -0.17 | 0.16 | 1005.15 |
| 3293 | PEPDNFWC | -0.67 | Non-Toxin | -0.14 | 0.16 | 1007.18 |
| 3294 | PEPDNFWN | -1.29 | Non-Toxin | -0.22 | 0.16 | 1018.15 |
| 3295 | PEPDNFWQ | -1.18 | Non-Toxin | -0.23 | 0.32 | 1032.18 |
| 3296 | PEPDNFYS | -1.35 | Non-Toxin | -0.22 | 0.16 | 968.08 |
| 3297 | PEPDNFYT | -1.32 | Non-Toxin | -0.21 | 0.16 | 982.11 |
| 3298 | PEPDNFYC | -1.16 | Non-Toxin | -0.18 | 0.16 | 984.14 |
| 3299 | PEPDNFYN | -1.29 | Non-Toxin | -0.27 | 0.16 | 995.11 |
| 3300 | PEPDNFYQ | -1.12 | Non-Toxin | -0.27 | 0.32 | 1009.14 |
| 3301 | PEPDNWFS | -1.02 | Non-Toxin | -0.18 | 0.16 | 991.12 |
| 3302 | PEPDNWFT | -0.95 | Non-Toxin | -0.17 | 0.16 | 1005.15 |
| 3303 | PEPDNWFC | -0.57 | Non-Toxin | -0.14 | 0.16 | 1007.18 |
| 3304 | PEPDNWFN | -0.96 | Non-Toxin | -0.22 | 0.16 | 1018.15 |
| 3305 | PEPDNWFQ | -0.98 | Non-Toxin | -0.23 | 0.32 | 1032.18 |
| 3306 | PEPDNWWS | -0.96 | Non-Toxin | -0.2 | 0.16 | 1030.16 |
| 3307 | PEPDNWWT | -0.93 | Non-Toxin | -0.19 | 0.16 | 1044.19 |
| 3308 | PEPDNWWC | -0.4 | Non-Toxin | -0.17 | 0.16 | 1046.22 |
| 3309 | PEPDNWWN | -1.03 | Non-Toxin | -0.25 | 0.16 | 1057.19 |
| 3310 | PEPDNWWQ | -0.92 | Non-Toxin | -0.26 | 0.32 | 1071.22 |
| 3311 | PEPDNWYS | -0.85 | Non-Toxin | -0.25 | 0.16 | 1007.12 |
| 3312 | PEPDNWYT | -0.84 | Non-Toxin | -0.24 | 0.16 | 1021.15 |
| 3313 | PEPDNWYC | -0.61 | Non-Toxin | -0.21 | 0.16 | 1023.18 |
| 3314 | PEPDNWYN | -0.79 | Non-Toxin | -0.3 | 0.16 | 1034.15 |
| 3315 | PEPDNWYQ | -0.59 | Non-Toxin | -0.3 | 0.32 | 1048.18 |
| 3316 | PEPDNYFS | -0.89 | Non-Toxin | -0.22 | 0.16 | 968.08 |
| 3317 | PEPDNYFT | -0.83 | Non-Toxin | -0.21 | 0.16 | 982.11 |
| 3318 | PEPDNYFC | -0.46 | Non-Toxin | -0.18 | 0.16 | 984.14 |
| 3319 | PEPDNYFN | -0.87 | Non-Toxin | -0.27 | 0.16 | 995.11 |
| 3320 | PEPDNYFQ | -0.88 | Non-Toxin | -0.27 | 0.32 | 1009.14 |
| 3321 | PEPDNYWS | -0.94 | Non-Toxin | -0.25 | 0.16 | 1007.12 |
| 3322 | PEPDNYWT | -0.91 | Non-Toxin | -0.24 | 0.16 | 1021.15 |
| 3323 | PEPDNYWC | -0.38 | Non-Toxin | -0.21 | 0.16 | 1023.18 |
| 3324 | PEPDNYWN | -1.02 | Non-Toxin | -0.3 | 0.16 | 1034.15 |
| 3325 | PEPDNYWQ | -0.9 | Non-Toxin | -0.3 | 0.32 | 1048.18 |
| 3326 | PEPDNYYS | -0.86 | Non-Toxin | -0.29 | 0.16 | 984.08 |
| 3327 | PEPDNYYT | -0.81 | Non-Toxin | -0.28 | 0.16 | 998.11 |
| 3328 | PEPDNYYC | -0.5 | Non-Toxin | -0.26 | 0.16 | 1000.14 |
| 3329 | PEPDNYYN | -0.81 | Non-Toxin | -0.34 | 0.16 | 1011.11 |
| 3330 | PEPDNYYQ | -0.6 | Non-Toxin | -0.35 | 0.32 | 1025.14 |
| 3331 | PEPDQFFS | -0.8 | Non-Toxin | -0.15 | 0.32 | 966.11 |
| 3332 | PEPDQFFT | -0.7 | Non-Toxin | -0.14 | 0.32 | 980.14 |
| 3333 | PEPDQFFC | -0.32 | Non-Toxin | -0.11 | 0.32 | 982.17 |
| 3334 | PEPDQFFN | -0.7 | Non-Toxin | -0.2 | 0.32 | 993.14 |
| 3335 | PEPDQFFQ | -0.75 | Non-Toxin | -0.21 | 0.47 | 1007.17 |
| 3336 | PEPDQFWS | -0.88 | Non-Toxin | -0.18 | 0.32 | 1005.15 |
| 3337 | PEPDQFWT | -0.83 | Non-Toxin | -0.17 | 0.32 | 1019.18 |
| 3338 | PEPDQFWC | -0.31 | Non-Toxin | -0.14 | 0.32 | 1021.21 |
| 3339 | PEPDQFWN | -0.94 | Non-Toxin | -0.23 | 0.32 | 1032.18 |
| 3340 | PEPDQFWQ | -0.84 | Non-Toxin | -0.23 | 0.47 | 1046.21 |
| 3341 | PEPDQFYS | -0.96 | Non-Toxin | -0.23 | 0.32 | 982.11 |
| 3342 | PEPDQFYT | -0.94 | Non-Toxin | -0.21 | 0.32 | 996.14 |
| 3343 | PEPDQFYC | -0.76 | Non-Toxin | -0.19 | 0.32 | 998.17 |
| 3344 | PEPDQFYN | -0.95 | Non-Toxin | -0.27 | 0.32 | 1009.14 |
| 3345 | PEPDQFYQ | -0.72 | Non-Toxin | -0.28 | 0.47 | 1023.17 |
| 3346 | PEPDQWFS | -0.88 | Non-Toxin | -0.18 | 0.32 | 1005.15 |
| 3347 | PEPDQWFT | -0.81 | Non-Toxin | -0.17 | 0.32 | 1019.18 |
| 3348 | PEPDQWFC | -0.43 | Non-Toxin | -0.14 | 0.32 | 1021.21 |
| 3349 | PEPDQWFN | -0.81 | Non-Toxin | -0.23 | 0.32 | 1032.18 |
| 3350 | PEPDQWFQ | -0.83 | Non-Toxin | -0.23 | 0.47 | 1046.21 |
| 3351 | PEPDQWWS | -0.83 | Non-Toxin | -0.21 | 0.32 | 1044.19 |
| 3352 | PEPDQWWT | -0.79 | Non-Toxin | -0.2 | 0.32 | 1058.22 |
| 3353 | PEPDQWWC | -0.25 | Non-Toxin | -0.17 | 0.32 | 1060.25 |
| 3354 | PEPDQWWN | -0.89 | Non-Toxin | -0.26 | 0.32 | 1071.22 |
| 3355 | PEPDQWWQ | -0.78 | Non-Toxin | -0.26 | 0.47 | 1085.25 |
| 3356 | PEPDQWYS | -0.66 | Non-Toxin | -0.26 | 0.32 | 1021.15 |
| 3357 | PEPDQWYT | -0.67 | Non-Toxin | -0.24 | 0.32 | 1035.18 |
| 3358 | PEPDQWYC | -0.43 | Non-Toxin | -0.22 | 0.32 | 1037.21 |
| 3359 | PEPDQWYN | -0.65 | Non-Toxin | -0.3 | 0.32 | 1048.18 |
| 3360 | PEPDQWYQ | -0.44 | Non-Toxin | -0.31 | 0.47 | 1062.21 |
| 3361 | PEPDQYFS | -0.99 | Non-Toxin | -0.23 | 0.32 | 982.11 |
| 3362 | PEPDQYFT | -0.89 | Non-Toxin | -0.21 | 0.32 | 996.14 |
| 3363 | PEPDQYFC | -0.55 | Non-Toxin | -0.19 | 0.32 | 998.17 |
| 3364 | PEPDQYFN | -0.94 | Non-Toxin | -0.27 | 0.32 | 1009.14 |
| 3365 | PEPDQYFQ | -0.96 | Non-Toxin | -0.28 | 0.47 | 1023.17 |
| 3366 | PEPDQYWS | -1.01 | Non-Toxin | -0.26 | 0.32 | 1021.15 |
| 3367 | PEPDQYWT | -0.96 | Non-Toxin | -0.24 | 0.32 | 1035.18 |
| 3368 | PEPDQYWC | -0.43 | Non-Toxin | -0.22 | 0.32 | 1037.21 |
| 3369 | PEPDQYWN | -1.07 | Non-Toxin | -0.3 | 0.32 | 1048.18 |
| 3370 | PEPDQYWQ | -0.95 | Non-Toxin | -0.31 | 0.47 | 1062.21 |
| 3371 | PEPDQYYS | -0.9 | Non-Toxin | -0.3 | 0.32 | 998.11 |
| 3372 | PEPDQYYT | -0.88 | Non-Toxin | -0.29 | 0.32 | 1012.14 |
| 3373 | PEPDQYYC | -0.61 | Non-Toxin | -0.26 | 0.32 | 1014.17 |
| 3374 | PEPDQYYN | -0.87 | Non-Toxin | -0.35 | 0.32 | 1025.14 |
| 3375 | PEPDQYYQ | -0.67 | Non-Toxin | -0.35 | 0.47 | 1039.17 |
| 3376 | PEPESFFS | -0.87 | Non-Toxin | -0.08 | 0.32 | 939.08 |
| 3377 | PEPESFFT | -0.83 | Non-Toxin | -0.07 | 0.32 | 953.11 |
| 3378 | PEPESFFC | -0.57 | Non-Toxin | -0.05 | 0.32 | 955.14 |
| 3379 | PEPESFFN | -0.85 | Non-Toxin | -0.13 | 0.32 | 966.11 |
| 3380 | PEPESFFQ | -0.78 | Non-Toxin | -0.14 | 0.47 | 980.14 |
| 3381 | PEPESFWS | -0.97 | Non-Toxin | -0.11 | 0.32 | 978.12 |
| 3382 | PEPESFWT | -0.89 | Non-Toxin | -0.1 | 0.32 | 992.15 |
| 3383 | PEPESFWC | -0.54 | Non-Toxin | -0.08 | 0.32 | 994.18 |
| 3384 | PEPESFWN | -0.98 | Non-Toxin | -0.16 | 0.32 | 1005.15 |
| 3385 | PEPESFWQ | -0.9 | Non-Toxin | -0.17 | 0.47 | 1019.18 |
| 3386 | PEPESFYS | -1.05 | Non-Toxin | -0.16 | 0.32 | 955.08 |
| 3387 | PEPESFYT | -1.02 | Non-Toxin | -0.15 | 0.32 | 969.11 |
| 3388 | PEPESFYC | -0.89 | Non-Toxin | -0.12 | 0.32 | 971.14 |
| 3389 | PEPESFYN | -1 | Non-Toxin | -0.21 | 0.32 | 982.11 |
| 3390 | PEPESFYQ | -0.86 | Non-Toxin | -0.21 | 0.47 | 996.14 |
| 3391 | PEPESWFS | -1.09 | Non-Toxin | -0.12 | 0.32 | 978.12 |
| 3392 | PEPESWFT | -1.05 | Non-Toxin | -0.11 | 0.32 | 992.15 |
| 3393 | PEPESWFC | -0.77 | Non-Toxin | -0.08 | 0.32 | 994.18 |
| 3394 | PEPESWFN | -1.05 | Non-Toxin | -0.16 | 0.32 | 1005.15 |
| 3395 | PEPESWFQ | -1.01 | Non-Toxin | -0.17 | 0.47 | 1019.18 |
| 3396 | PEPESWWS | -1.05 | Non-Toxin | -0.15 | 0.32 | 1017.16 |
| 3397 | PEPESWWT | -1.01 | Non-Toxin | -0.14 | 0.32 | 1031.19 |
| 3398 | PEPESWWC | -0.67 | Non-Toxin | -0.11 | 0.32 | 1033.22 |
| 3399 | PEPESWWN | -1.08 | Non-Toxin | -0.19 | 0.32 | 1044.19 |
| 3400 | PEPESWWQ | -1.01 | Non-Toxin | -0.2 | 0.47 | 1058.22 |
| 3401 | PEPESWYS | -0.96 | Non-Toxin | -0.19 | 0.32 | 994.12 |
| 3402 | PEPESWYT | -0.95 | Non-Toxin | -0.18 | 0.32 | 1008.15 |
| 3403 | PEPESWYC | -0.77 | Non-Toxin | -0.15 | 0.32 | 1010.18 |
| 3404 | PEPESWYN | -0.92 | Non-Toxin | -0.24 | 0.32 | 1021.15 |
| 3405 | PEPESWYQ | -0.76 | Non-Toxin | -0.24 | 0.47 | 1035.18 |
| 3406 | PEPESYFS | -0.98 | Non-Toxin | -0.16 | 0.32 | 955.08 |
| 3407 | PEPESYFT | -0.93 | Non-Toxin | -0.15 | 0.32 | 969.11 |
| 3408 | PEPESYFC | -0.67 | Non-Toxin | -0.12 | 0.32 | 971.14 |
| 3409 | PEPESYFN | -0.97 | Non-Toxin | -0.21 | 0.32 | 982.11 |
| 3410 | PEPESYFQ | -0.92 | Non-Toxin | -0.21 | 0.47 | 996.14 |
| 3411 | PEPESYWS | -1.02 | Non-Toxin | -0.19 | 0.32 | 994.12 |
| 3412 | PEPESYWT | -0.99 | Non-Toxin | -0.18 | 0.32 | 1008.15 |
| 3413 | PEPESYWC | -0.63 | Non-Toxin | -0.15 | 0.32 | 1010.18 |
| 3414 | PEPESYWN | -1.05 | Non-Toxin | -0.24 | 0.32 | 1021.15 |
| 3415 | PEPESYWQ | -0.97 | Non-Toxin | -0.24 | 0.47 | 1035.18 |
| 3416 | PEPESYYS | -0.93 | Non-Toxin | -0.23 | 0.32 | 971.08 |
| 3417 | PEPESYYT | -0.92 | Non-Toxin | -0.22 | 0.32 | 985.11 |
| 3418 | PEPESYYC | -0.69 | Non-Toxin | -0.19 | 0.32 | 987.14 |
| 3419 | PEPESYYN | -0.89 | Non-Toxin | -0.28 | 0.32 | 998.11 |
| 3420 | PEPESYYQ | -0.72 | Non-Toxin | -0.29 | 0.47 | 1012.14 |
| 3421 | PEPETFFS | -1.09 | Non-Toxin | -0.07 | 0.32 | 953.11 |
| 3422 | PEPETFFT | -1.06 | Non-Toxin | -0.06 | 0.32 | 967.14 |
| 3423 | PEPETFFC | -0.78 | Non-Toxin | -0.04 | 0.32 | 969.17 |
| 3424 | PEPETFFN | -1.02 | Non-Toxin | -0.12 | 0.32 | 980.14 |
| 3425 | PEPETFFQ | -1.04 | Non-Toxin | -0.13 | 0.47 | 994.17 |
| 3426 | PEPETFWS | -1.14 | Non-Toxin | -0.1 | 0.32 | 992.15 |
| 3427 | PEPETFWT | -1.08 | Non-Toxin | -0.09 | 0.32 | 1006.18 |
| 3428 | PEPETFWC | -0.75 | Non-Toxin | -0.07 | 0.32 | 1008.21 |
| 3429 | PEPETFWN | -1.17 | Non-Toxin | -0.15 | 0.32 | 1019.18 |
| 3430 | PEPETFWQ | -1.09 | Non-Toxin | -0.16 | 0.47 | 1033.21 |
| 3431 | PEPETFYS | -1.22 | Non-Toxin | -0.15 | 0.32 | 969.11 |
| 3432 | PEPETFYT | -1.19 | Non-Toxin | -0.14 | 0.32 | 983.14 |
| 3433 | PEPETFYC | -1.07 | Non-Toxin | -0.11 | 0.32 | 985.17 |
| 3434 | PEPETFYN | -1.18 | Non-Toxin | -0.2 | 0.32 | 996.14 |
| 3435 | PEPETFYQ | -1.05 | Non-Toxin | -0.2 | 0.47 | 1010.17 |
| 3436 | PEPETWFS | -1.09 | Non-Toxin | -0.1 | 0.32 | 992.15 |
| 3437 | PEPETWFT | -1.06 | Non-Toxin | -0.1 | 0.32 | 1006.18 |
| 3438 | PEPETWFC | -0.79 | Non-Toxin | -0.07 | 0.32 | 1008.21 |
| 3439 | PEPETWFN | -1.04 | Non-Toxin | -0.15 | 0.32 | 1019.18 |
| 3440 | PEPETWFQ | -1.04 | Non-Toxin | -0.16 | 0.47 | 1033.21 |
| 3441 | PEPETWWS | -1.06 | Non-Toxin | -0.14 | 0.32 | 1031.19 |
| 3442 | PEPETWWT | -1.03 | Non-Toxin | -0.12 | 0.32 | 1045.22 |
| 3443 | PEPETWWC | -0.66 | Non-Toxin | -0.1 | 0.32 | 1047.25 |
| 3444 | PEPETWWN | -1.1 | Non-Toxin | -0.18 | 0.32 | 1058.22 |
| 3445 | PEPETWWQ | -1.02 | Non-Toxin | -0.19 | 0.47 | 1072.25 |
| 3446 | PEPETWYS | -0.99 | Non-Toxin | -0.18 | 0.32 | 1008.15 |
| 3447 | PEPETWYT | -0.95 | Non-Toxin | -0.17 | 0.32 | 1022.18 |
| 3448 | PEPETWYC | -0.79 | Non-Toxin | -0.14 | 0.32 | 1024.21 |
| 3449 | PEPETWYN | -0.94 | Non-Toxin | -0.23 | 0.32 | 1035.18 |
| 3450 | PEPETWYQ | -0.79 | Non-Toxin | -0.23 | 0.47 | 1049.21 |
| 3451 | PEPETYFS | -1 | Non-Toxin | -0.15 | 0.32 | 969.11 |
| 3452 | PEPETYFT | -0.96 | Non-Toxin | -0.14 | 0.32 | 983.14 |
| 3453 | PEPETYFC | -0.71 | Non-Toxin | -0.11 | 0.32 | 985.17 |
| 3454 | PEPETYFN | -0.99 | Non-Toxin | -0.2 | 0.32 | 996.14 |
| 3455 | PEPETYFQ | -0.96 | Non-Toxin | -0.2 | 0.47 | 1010.17 |
| 3456 | PEPETYWS | -1.04 | Non-Toxin | -0.18 | 0.32 | 1008.15 |
| 3457 | PEPETYWT | -1.01 | Non-Toxin | -0.17 | 0.32 | 1022.18 |
| 3458 | PEPETYWC | -0.65 | Non-Toxin | -0.14 | 0.32 | 1024.21 |
| 3459 | PEPETYWN | -1.08 | Non-Toxin | -0.23 | 0.32 | 1035.18 |
| 3460 | PEPETYWQ | -1 | Non-Toxin | -0.23 | 0.47 | 1049.21 |
| 3461 | PEPETYYS | -0.99 | Non-Toxin | -0.22 | 0.32 | 985.11 |
| 3462 | PEPETYYT | -0.94 | Non-Toxin | -0.21 | 0.32 | 999.14 |
| 3463 | PEPETYYC | -0.76 | Non-Toxin | -0.18 | 0.32 | 1001.17 |
| 3464 | PEPETYYN | -0.94 | Non-Toxin | -0.27 | 0.32 | 1012.14 |
| 3465 | PEPETYYQ | -0.79 | Non-Toxin | -0.28 | 0.47 | 1026.17 |
| 3466 | PEPECFFS | -0.73 | Non-Toxin | -0.05 | 0.32 | 955.14 |
| 3467 | PEPECFFT | -0.67 | Non-Toxin | -0.04 | 0.32 | 969.17 |
| 3468 | PEPECFFC | -0.37 | Non-Toxin | -0.01 | 0.32 | 971.2 |
| 3469 | PEPECFFN | -0.68 | Non-Toxin | -0.09 | 0.32 | 982.17 |
| 3470 | PEPECFFQ | -0.68 | Non-Toxin | -0.1 | 0.47 | 996.2 |
| 3471 | PEPECFWS | -0.88 | Non-Toxin | -0.08 | 0.32 | 994.18 |
| 3472 | PEPECFWT | -0.85 | Non-Toxin | -0.07 | 0.32 | 1008.21 |
| 3473 | PEPECFWC | -0.49 | Non-Toxin | -0.04 | 0.32 | 1010.24 |
| 3474 | PEPECFWN | -0.91 | Non-Toxin | -0.12 | 0.32 | 1021.21 |
| 3475 | PEPECFWQ | -0.84 | Non-Toxin | -0.13 | 0.47 | 1035.24 |
| 3476 | PEPECFYS | -0.9 | Non-Toxin | -0.12 | 0.32 | 971.14 |
| 3477 | PEPECFYT | -0.86 | Non-Toxin | -0.11 | 0.32 | 985.17 |
| 3478 | PEPECFYC | -0.8 | Non-Toxin | -0.08 | 0.32 | 987.2 |
| 3479 | PEPECFYN | -0.85 | Non-Toxin | -0.17 | 0.32 | 998.17 |
| 3480 | PEPECFYQ | -0.72 | Non-Toxin | -0.17 | 0.47 | 1012.2 |
| 3481 | PEPECWFS | -0.73 | Non-Toxin | -0.08 | 0.32 | 994.18 |
| 3482 | PEPECWFT | -0.66 | Non-Toxin | -0.07 | 0.32 | 1008.21 |
| 3483 | PEPECWFC | -0.39 | Non-Toxin | -0.04 | 0.32 | 1010.24 |
| 3484 | PEPECWFN | -0.66 | Non-Toxin | -0.12 | 0.32 | 1021.21 |
| 3485 | PEPECWFQ | -0.66 | Non-Toxin | -0.13 | 0.47 | 1035.24 |
| 3486 | PEPECWWS | -0.67 | Non-Toxin | -0.11 | 0.32 | 1033.22 |
| 3487 | PEPECWWT | -0.65 | Non-Toxin | -0.1 | 0.32 | 1047.25 |
| 3488 | PEPECWWC | -0.28 | Non-Toxin | -0.07 | 0.32 | 1049.28 |
| 3489 | PEPECWWN | -0.71 | Non-Toxin | -0.15 | 0.32 | 1060.25 |
| 3490 | PEPECWWQ | -0.64 | Non-Toxin | -0.16 | 0.47 | 1074.28 |
| 3491 | PEPECWYS | -0.6 | Non-Toxin | -0.15 | 0.32 | 1010.18 |
| 3492 | PEPECWYT | -0.55 | Non-Toxin | -0.14 | 0.32 | 1024.21 |
| 3493 | PEPECWYC | -0.41 | Non-Toxin | -0.11 | 0.32 | 1026.24 |
| 3494 | PEPECWYN | -0.55 | Non-Toxin | -0.2 | 0.32 | 1037.21 |
| 3495 | PEPECWYQ | -0.4 | Non-Toxin | -0.2 | 0.47 | 1051.24 |
| 3496 | PEPECYFS | -0.67 | Non-Toxin | -0.12 | 0.32 | 971.14 |
| 3497 | PEPECYFT | -0.59 | Non-Toxin | -0.11 | 0.32 | 985.17 |
| 3498 | PEPECYFC | -0.34 | Non-Toxin | -0.08 | 0.32 | 987.2 |
| 3499 | PEPECYFN | -0.61 | Non-Toxin | -0.17 | 0.32 | 998.17 |
| 3500 | PEPECYFQ | -0.61 | Non-Toxin | -0.17 | 0.47 | 1012.2 |
| 3501 | PEPECYWS | -0.62 | Non-Toxin | -0.15 | 0.32 | 1010.18 |
| 3502 | PEPECYWT | -0.6 | Non-Toxin | -0.14 | 0.32 | 1024.21 |
| 3503 | PEPECYWC | -0.27 | Non-Toxin | -0.11 | 0.32 | 1026.24 |
| 3504 | PEPECYWN | -0.68 | Non-Toxin | -0.2 | 0.32 | 1037.21 |
| 3505 | PEPECYWQ | -0.6 | Non-Toxin | -0.2 | 0.47 | 1051.24 |
| 3506 | PEPECYYS | -0.57 | Non-Toxin | -0.19 | 0.32 | 987.14 |
| 3507 | PEPECYYT | -0.55 | Non-Toxin | -0.18 | 0.32 | 1001.17 |
| 3508 | PEPECYYC | -0.37 | Non-Toxin | -0.16 | 0.32 | 1003.2 |
| 3509 | PEPECYYN | -0.51 | Non-Toxin | -0.24 | 0.32 | 1014.17 |
| 3510 | PEPECYYQ | -0.38 | Non-Toxin | -0.25 | 0.47 | 1028.2 |
| 3511 | PEPENFFS | -1.11 | Non-Toxin | -0.13 | 0.32 | 966.11 |
| 3512 | PEPENFFT | -1.04 | Non-Toxin | -0.12 | 0.32 | 980.14 |
| 3513 | PEPENFFC | -0.78 | Non-Toxin | -0.1 | 0.32 | 982.17 |
| 3514 | PEPENFFN | -1.04 | Non-Toxin | -0.18 | 0.32 | 993.14 |
| 3515 | PEPENFFQ | -1.03 | Non-Toxin | -0.19 | 0.47 | 1007.17 |
| 3516 | PEPENFWS | -1.16 | Non-Toxin | -0.16 | 0.32 | 1005.15 |
| 3517 | PEPENFWT | -1.12 | Non-Toxin | -0.15 | 0.32 | 1019.18 |
| 3518 | PEPENFWC | -0.79 | Non-Toxin | -0.12 | 0.32 | 1021.21 |
| 3519 | PEPENFWN | -1.19 | Non-Toxin | -0.21 | 0.32 | 1032.18 |
| 3520 | PEPENFWQ | -1.12 | Non-Toxin | -0.22 | 0.47 | 1046.21 |
| 3521 | PEPENFYS | -1.22 | Non-Toxin | -0.21 | 0.32 | 982.11 |
| 3522 | PEPENFYT | -1.18 | Non-Toxin | -0.2 | 0.32 | 996.14 |
| 3523 | PEPENFYC | -1.06 | Non-Toxin | -0.17 | 0.32 | 998.17 |
| 3524 | PEPENFYN | -1.18 | Non-Toxin | -0.25 | 0.32 | 1009.14 |
| 3525 | PEPENFYQ | -1.05 | Non-Toxin | -0.26 | 0.47 | 1023.17 |
| 3526 | PEPENWFS | -1.03 | Non-Toxin | -0.16 | 0.32 | 1005.15 |
| 3527 | PEPENWFT | -0.97 | Non-Toxin | -0.15 | 0.32 | 1019.18 |
| 3528 | PEPENWFC | -0.71 | Non-Toxin | -0.12 | 0.32 | 1021.21 |
| 3529 | PEPENWFN | -0.97 | Non-Toxin | -0.21 | 0.32 | 1032.18 |
| 3530 | PEPENWFQ | -0.95 | Non-Toxin | -0.22 | 0.47 | 1046.21 |
| 3531 | PEPENWWS | -0.98 | Non-Toxin | -0.19 | 0.32 | 1044.19 |
| 3532 | PEPENWWT | -0.95 | Non-Toxin | -0.18 | 0.32 | 1058.22 |
| 3533 | PEPENWWC | -0.61 | Non-Toxin | -0.15 | 0.32 | 1060.25 |
| 3534 | PEPENWWN | -1.02 | Non-Toxin | -0.24 | 0.32 | 1071.22 |
| 3535 | PEPENWWQ | -0.95 | Non-Toxin | -0.25 | 0.47 | 1085.25 |
| 3536 | PEPENWYS | -0.9 | Non-Toxin | -0.24 | 0.32 | 1021.15 |
| 3537 | PEPENWYT | -0.87 | Non-Toxin | -0.23 | 0.32 | 1035.18 |
| 3538 | PEPENWYC | -0.71 | Non-Toxin | -0.2 | 0.32 | 1037.21 |
| 3539 | PEPENWYN | -0.86 | Non-Toxin | -0.28 | 0.32 | 1048.18 |
| 3540 | PEPENWYQ | -0.72 | Non-Toxin | -0.29 | 0.47 | 1062.21 |
| 3541 | PEPENYFS | -0.93 | Non-Toxin | -0.21 | 0.32 | 982.11 |
| 3542 | PEPENYFT | -0.88 | Non-Toxin | -0.2 | 0.32 | 996.14 |
| 3543 | PEPENYFC | -0.62 | Non-Toxin | -0.17 | 0.32 | 998.17 |
| 3544 | PEPENYFN | -0.9 | Non-Toxin | -0.25 | 0.32 | 1009.14 |
| 3545 | PEPENYFQ | -0.86 | Non-Toxin | -0.26 | 0.47 | 1023.17 |
| 3546 | PEPENYWS | -0.96 | Non-Toxin | -0.24 | 0.32 | 1021.15 |
| 3547 | PEPENYWT | -0.93 | Non-Toxin | -0.23 | 0.32 | 1035.18 |
| 3548 | PEPENYWC | -0.59 | Non-Toxin | -0.2 | 0.32 | 1037.21 |
| 3549 | PEPENYWN | -1.01 | Non-Toxin | -0.28 | 0.32 | 1048.18 |
| 3550 | PEPENYWQ | -0.93 | Non-Toxin | -0.29 | 0.47 | 1062.21 |
| 3551 | PEPENYYS | -0.88 | Non-Toxin | -0.28 | 0.32 | 998.11 |
| 3552 | PEPENYYT | -0.83 | Non-Toxin | -0.27 | 0.32 | 1012.14 |
| 3553 | PEPENYYC | -0.61 | Non-Toxin | -0.24 | 0.32 | 1014.17 |
| 3554 | PEPENYYN | -0.85 | Non-Toxin | -0.33 | 0.32 | 1025.14 |
| 3555 | PEPENYYQ | -0.7 | Non-Toxin | -0.33 | 0.47 | 1039.17 |
| 3556 | PEPEQFFS | -1 | Non-Toxin | -0.14 | 0.47 | 980.14 |
| 3557 | PEPEQFFT | -0.92 | Non-Toxin | -0.13 | 0.47 | 994.17 |
| 3558 | PEPEQFFC | -0.66 | Non-Toxin | -0.1 | 0.47 | 996.2 |
| 3559 | PEPEQFFN | -0.92 | Non-Toxin | -0.19 | 0.47 | 1007.17 |
| 3560 | PEPEQFFQ | -0.93 | Non-Toxin | -0.19 | 0.63 | 1021.2 |
| 3561 | PEPEQFWS | -1.05 | Non-Toxin | -0.17 | 0.47 | 1019.18 |
| 3562 | PEPEQFWT | -1 | Non-Toxin | -0.16 | 0.47 | 1033.21 |
| 3563 | PEPEQFWC | -0.67 | Non-Toxin | -0.13 | 0.47 | 1035.24 |
| 3564 | PEPEQFWN | -1.08 | Non-Toxin | -0.22 | 0.47 | 1046.21 |
| 3565 | PEPEQFWQ | -1.01 | Non-Toxin | -0.22 | 0.63 | 1060.24 |
| 3566 | PEPEQFYS | -1.13 | Non-Toxin | -0.21 | 0.47 | 996.14 |
| 3567 | PEPEQFYT | -1.08 | Non-Toxin | -0.2 | 0.47 | 1010.17 |
| 3568 | PEPEQFYC | -0.95 | Non-Toxin | -0.17 | 0.47 | 1012.2 |
| 3569 | PEPEQFYN | -1.08 | Non-Toxin | -0.26 | 0.47 | 1023.17 |
| 3570 | PEPEQFYQ | -0.94 | Non-Toxin | -0.27 | 0.63 | 1037.2 |
| 3571 | PEPEQWFS | -1.07 | Non-Toxin | -0.17 | 0.47 | 1019.18 |
| 3572 | PEPEQWFT | -1.01 | Non-Toxin | -0.16 | 0.47 | 1033.21 |
| 3573 | PEPEQWFC | -0.75 | Non-Toxin | -0.13 | 0.47 | 1035.24 |
| 3574 | PEPEQWFN | -1.01 | Non-Toxin | -0.22 | 0.47 | 1046.21 |
| 3575 | PEPEQWFQ | -1 | Non-Toxin | -0.22 | 0.63 | 1060.24 |
| 3576 | PEPEQWWS | -1.04 | Non-Toxin | -0.2 | 0.47 | 1058.22 |
| 3577 | PEPEQWWT | -1 | Non-Toxin | -0.19 | 0.47 | 1072.25 |
| 3578 | PEPEQWWC | -0.65 | Non-Toxin | -0.16 | 0.47 | 1074.28 |
| 3579 | PEPEQWWN | -1.07 | Non-Toxin | -0.25 | 0.47 | 1085.25 |
| 3580 | PEPEQWWQ | -0.99 | Non-Toxin | -0.25 | 0.63 | 1099.28 |
| 3581 | PEPEQWYS | -0.94 | Non-Toxin | -0.24 | 0.47 | 1035.18 |
| 3582 | PEPEQWYT | -0.91 | Non-Toxin | -0.23 | 0.47 | 1049.21 |
| 3583 | PEPEQWYC | -0.75 | Non-Toxin | -0.2 | 0.47 | 1051.24 |
| 3584 | PEPEQWYN | -0.89 | Non-Toxin | -0.29 | 0.47 | 1062.21 |
| 3585 | PEPEQWYQ | -0.76 | Non-Toxin | -0.3 | 0.63 | 1076.24 |
| 3586 | PEPEQYFS | -1.14 | Non-Toxin | -0.21 | 0.47 | 996.14 |
| 3587 | PEPEQYFT | -1.06 | Non-Toxin | -0.2 | 0.47 | 1010.17 |
| 3588 | PEPEQYFC | -0.82 | Non-Toxin | -0.17 | 0.47 | 1012.2 |
| 3589 | PEPEQYFN | -1.09 | Non-Toxin | -0.26 | 0.47 | 1023.17 |
| 3590 | PEPEQYFQ | -1.08 | Non-Toxin | -0.27 | 0.63 | 1037.2 |
| 3591 | PEPEQYWS | -1.16 | Non-Toxin | -0.24 | 0.47 | 1035.18 |
| 3592 | PEPEQYWT | -1.11 | Non-Toxin | -0.23 | 0.47 | 1049.21 |
| 3593 | PEPEQYWC | -0.77 | Non-Toxin | -0.2 | 0.47 | 1051.24 |
| 3594 | PEPEQYWN | -1.19 | Non-Toxin | -0.29 | 0.47 | 1062.21 |
| 3595 | PEPEQYWQ | -1.11 | Non-Toxin | -0.3 | 0.63 | 1076.24 |
| 3596 | PEPEQYYS | -1.09 | Non-Toxin | -0.29 | 0.47 | 1012.14 |
| 3597 | PEPEQYYT | -1.05 | Non-Toxin | -0.28 | 0.47 | 1026.17 |
| 3598 | PEPEQYYC | -0.86 | Non-Toxin | -0.25 | 0.47 | 1028.2 |
| 3599 | PEPEQYYN | -1.04 | Non-Toxin | -0.33 | 0.47 | 1039.17 |
| 3600 | PEPEQYYQ | -0.91 | Non-Toxin | -0.34 | 0.63 | 1053.2 |
